# OncoSimulR: genetic simulation with arbitrary epistasis and mutator genes in asexual populations

**DOI:** 10.1093/bioinformatics/btx077

**Published:** 2017-02-10

**Authors:** Ramon Diaz-Uriarte

**Affiliations:** Department of Biochemistry, Universidad Autónoma de Madrid, Instituto de Investigaciones Biomédicas ‘Alberto Sols’ (UAM-CSIC), Madrid, Spain

## Abstract

**Summary:**

OncoSimulR implements forward-time genetic simulations of biallelic loci in asexual populations with special focus on cancer progression. Fitness can be defined as an arbitrary function of genetic interactions between multiple genes or modules of genes, including epistasis, restrictions in the order of accumulation of mutations, and order effects. Mutation rates can differ among genes, and can be affected by (anti)mutator genes. Also available are sampling from simulations (including single-cell sampling), plotting the genealogical relationships of clones and generating and plotting fitness landscapes.

**Availability and Implementation:**

Implemented in R and C ++, freely available from BioConductor for Linux, Mac and Windows under the GNU GPL license. Version 2.5.9 or higher available from: http://www.bioconductor.org/packages/devel/bioc/html/OncoSimulR.html. GitHub repository at: https://github.com/rdiaz02/OncoSimul

**Supplementary information:**

Supplementary data are available at *Bioinformatics* online.

## Introduction

Forward-time genetic simulations are used in population genetics and cancer research to verify analytic results, to generate data to assess the performance of statistical methods, and to examine complex models that are mathematically intractable (Thornton, 2014). Often, we will want to use a range of populations sizes, large genomes and flexible mechanisms to specify the effects of mutations on both fitness and mutation rates (to model mutator/antimutator genes; Gerrish *et al.*, 2007). If the effects of sampling are relevant (e.g. Diaz-Uriarte, 2015), we will want to use different sampling schemes and if understanding dynamics matters, we will want to track the history of the clones. Many forward-time simulators are available (see Peng *et al.*, 2012; Thornton, 2014, and the Genetic Simulation Resources page https://popmodels.cancercontrol.cancer.gov/gsr/). Some of the tools closest to fulfill the above needs are simuPOP (Peng *et al.*, 2012), fwdpp (Thornton, 2014), FFPopSim (Zanini and Neher, 2012) and TTP (Reiter *et al.*, 2013); these programs, however, miss some of the above mentioned features, especially flexible ways to specify fitness and mutator effects, order effects or gene-specific mutation rates.

## 2 Functionality

OncoSimulR is an individual-based forward-time genetic simulator for biallelic markers (wildtype versus mutated) in asexually reproducing populations without spatial structure (perfect mixing). Its design emphasizes flexible specification of fitness and mutator effects.

OncoSimulR uses a standard continuous time model, where individual cells divide, die and mutate with rates that can depend on genotype and population size; over time the abundance of the different genotypes changes by the action of selection (due to differences in net growth rates among genotypes), drift and mutation. As a result of a mutation in a preexisting clone new clones arise, and the birth rate of a newly arisen clone is determined at the time of its emergence as a function of its genotype. Simulations can use an exponential growth model or a model with carrying capacity (following McFarland *et al.*, 2013). For the exponential growth model, the death rate is fixed at one whereas in the model with carrying capacity death rate increases with population size. In both cases, therefore, fitness differences among genotypes in a given population at a given time are due to differences in the mapping between genotype and birth rate. A key feature of OncoSimulR is the flexibility to specify the dependence of birth rates on genotype and, thus, the flexibility to specify fitness. With OncoSimulR we can:

- Specify the fitness of each genotype.
- Use a system of blocks (that might share elements) to combine:
  - Effects on fitness of individual genes and epistatic effects of any order that involve an arbitrary number of genes.
  - Order effects on fitness involving arbitrary numbers of genes. With order effects (Ortmann *et al.*, 2015) the fitness of a genotype with genes A and B mutated depends on whether A or B mutated first.
  - Directed acyclic graphs (DAGs), as used in cancer progression networks such as Oncogenetic Trees and Conjunctive Bayesian Networks (Beerenwinkel *et al.*, 2014), to specify restrictions in the order of accumulation of mutations.

Mutator/antimutator genes can be specified similar to fitness effects. Genes with mutator effects can also have direct effects on fitness. Mutation rates can be gene-specific or common to all genes. In addition to genes, we can specify fitness and mutator effects using ‘modules’ (pathways).

Typical use cases involve tens to thousands of genes on population sizes up to 10^5^ to 10^7^ (see Supplementary documentation). OncoSimulR uses the state-of-the-art BNB algorithm of Mather *et al.* (2012). Simulations return the population size of every genotype/clone at each of the sampling periods. We can take samples from those data with single-cell or whole-tumor resolution. Additional functionality includes storing and plotting the parent-child (genealogical) relationships of clones, generating random fitness landscapes and plotting them (inspired by MAGELLAN: Brouillet *et al.*, 2015), statistics of evolutionary predictability, or generating random DAGs of restrictions in the order of mutations.

## 3 Using OncoSimulR: examples

The next are some research questions where OncoSimulR could be of help; full code is provided in the Supplementary documentation.

- **Recovering restrictions in the order of accumulation of mutations (Diaz-Uriarte, **2015**)**. Run simulations on random DAGs to obtain data to input to cancer progression network methods; compare inferred versus true DAGs.
- **Sign epistasis and crossing fitness valleys (Ochs and Desai, **2015**)**. Specify epistatic interactions and run simulations until fixation; examine proportion of genotypes fixed under different scenarios.
- **Predictability of evolution in complex fitness landscapes (**Szendro et al., 2013a**)**. Run simulations under random fitness landscapes and compare evolutionary predictability of trajectories.
- **Mutator genes (Gerrish *et al.*, **2007**)**. Specify different numbers/effects of mutator genes and examine how they affect cancer progression.
- **Epistatic interactions between drivers and passengers in cancer (Bauer *et al.*, **2014**) and consequences of order effects (Ortmann *et al.*,**^2015^**)**. Run simulations under different epistatic interactions between drivers and passengers or under different strengths of order effects and examine how often populations reach a certain size.

## 4 Conclusion

Salient features of OncoSimulR compared to other simulators are the unparalleled flexibility to specify fitness and mutator effects, with modules and order effects as particularly unique, and the options for sampling and stopping the simulations, especially convenient in cancer evolution models. Also unique in this type of software is the addition of functions for simulating fitness landscapes and assessing evolutionary predictability. OncoSimulR can thus be used to address questions that span from the effect of mutator genes in cancer, to the interplay between fitness landscapes and mutation rates. OncoSimulR can therefore be of interest to computational oncologists and evolutionary geneticists working on problems specific to asexual populations.

## Supplementary Material

Supplementary Data
