# Supplementary material for: OncoSimulR: genetic simulation with arbitrary epistasis and mutator genes in asexual populations
Source: Bioinformatics. 2017 Feb 10;33(12):1898–9. doi: 10.1093/bioinformatics/btx077 (PMC5946943; doi:10.1093/bioinformatics/btx077)
Supplement: Supplementary Data [file btx077_supp.pdf]

OncoSimulR: forward genetic simulation in asexual populations with arbitrary epistatic interactions and a focus on modeling tumor progression.

Ramon Diaz-Uriarte
Dept. Biochemistry, Universidad Autónoma de Madrid, Instituto de Investigaciones Biomédicas 'Alberto Sols' (UAM-CSIC), Madrid, Spain.
rdiaz02@gmail.com, http://ligarto.org/rdiaz

2017-01-09. OncoSimulR version 2.5.9. Revision: 5b63a1c

Contents

1 Introduction **6**
1.1 Key features of OncoSimulR 7
1.2 What kinds of questions is OncoSimulR suited for? 10
1.3 Examples of questions that can be addressed with OncoSimulR . . . 13
1.3.1 Recovering restrictions in the order of accumulation of mutations 13
1.3.2 Sign epistasis and probability of crossing fitness valleys 14
1.3.3 Predictability of evolution in complex fitness landscapes . . . 16
1.3.4 Mutator and antimutator genes 17
1.3.5 Epistatic interactions between drivers and passengers in cancer and the consequences of order effects 19
1.3.5.1 Epistatic interactions between drivers and passengers 19
1.3.5.2 Consequences of order effects for cancer initiation . . 21
1.4 Trade-offs and what is OncoSimulR not well suited for 23
1.5 Steps for using OncoSimulR 23
1.6 Two quick examples of fitness specifications 24
1.7 Citing OncoSimulR and other documentation 27
1.7.1 HTML and PDF versions of the vignette 29
1.8 Testing, code coverage, and other examples 29
1.9 Versions 29

2 Running time and space consumption of OncoSimulR **30**
2.1 Exp and McFL with "detectionProb" and pancreas example 31
2.1.1 Changing fitness: $s = 0.1$ and $s = 0.05$ 43
2.2 Several "common use cases" runs 45
2.2.1 Common use cases, set 1. 47
2.2.2 Common use cases, set 2. 51

2.3 Can we use a large number of genes? 53
2.3.1 Exponential model with 10,000 and 50,000 genes 53
2.3.1.1 Exponential, 10,000 genes, example 1 53
2.3.1.2 Exponential, 10,000 genes, example 2 54
2.3.1.3 Exponential, 50,000 genes, example 1 55
2.3.1.4 Exponential, 50,000 genes, example 2 56
2.3.1.5 Exponential, 50,000 genes, example 3 57
2.3.1.6 Interlude: where is that 1 GB coming from? 58
2.3.2 McFarland model with 50,000 genes; the effect of `keepEvery` . 58
2.3.2.1 McFarland, 50,000 genes, example 1 59
2.3.2.2 McFarland, 50,000 genes, example 2 59
2.3.2.3 McFarland, 50,000 genes, example 3 60
2.3.2.4 McFarland, 50,000 genes, example 4 61
2.3.2.5 McFarland, 50,000 genes, example 5 62
2.3.2.6 McFarland, 50,000 genes, example 6 63
2.3.3 Examples with $s = 0.05$ 64
2.3.4 The different consequences of `keepEvery = NA` in the Exp and McFL models 66
2.3.5 Are we keeping the complete history (genealogy) of the clones? 66
2.4 Population sizes $\geq 10^{10}$ 67
2.5 A summary of some determinants of running time and space consumption 70

3 Specifying fitness effects 72
3.1 Introduction to the specification of fitness effects 72
3.1.1 Explicit mapping of genotypes to fitness 74
3.1.2 How to specify fitness effects with the lego system 77
3.2 Numeric values of fitness effects 78
3.2.1 McFarland parameterization 78
3.2.2 No viability of clones and types of models 79
3.3 Genes without interactions 80
3.4 Using DAGs: Restrictions in the order of mutations as extended posets 81
3.4.1 AND, OR, XOR relationships 81
3.4.2 Fitness effects 82
3.4.3 Extended posets 83
3.4.4 DAGs: A first conjunction (AND) example 83
3.4.5 DAGs: A second conjunction example 86
3.4.6 DAGs: A semimonotone or “OR” example 88
3.4.7 An “XMPN” or “XOR” example 89
3.4.8 Posets: the three types of relationships 91
3.5 Modules 93
3.5.1 What does a module provide 93
3.5.2 Specifying modules 94
3.5.3 Modules and posets again: the three types of relationships and modules 95
3.6 Order effects 98
3.6.1 Order effects: three-gene orders 98

3.6.2 Order effects and modules with multiple genes 100
3.6.3 Order and modules with 325 genotypes 101
3.6.4 Order effects and genes without interactions 102
3.7 Epistasis 103
3.7.1 Epistasis: two alternative specifications 103
3.7.2 Epistasis with three genes and two alternative specifications . 105
3.7.3 Why can we specify some effects with a "-"? 107
3.7.4 Epistasis: modules 107
3.8 I do not want epistasis, but I want modules! 109
3.9 Synthetic viability 111
3.9.1 A simple synthetic viability example 111
3.9.2 Synthetic viability, non-zero fitness, and modules 112
3.10 Synthetic mortality or synthetic lethality 113
3.11 Possible issues with Bozic model 114
3.11.1 Synthetic viability using Bozic model 114
3.11.2 Numerical issues with death rates of 0 in Bozic model 115
3.12 A longer example: Poset, epistasis, synthetic mortality and viability, order effects and genes without interactions, with some modules . . . 117
3.13 Homozygosity, heterozygosity, oncogenes, tumor suppressors 123
3.14 Gene-specific mutation rates 124
3.15 Mutator genes 125

4 Plotting fitness landscapes 129

5 Specifying fitness effects: some examples from the literature 133
5.1 Bauer et al., 2014 133
5.1.1 Using a DAG 133
5.1.2 Specifying fitness of genotypes directly 136
5.2 Misra et al., 2014 138
5.2.1 Example 1.a 138
5.2.2 Example 1.b 139
5.2.3 Example 1.c 140
5.3 Ochs and Desai, 2015 141
5.4 Weissman et al., 2009 144
5.4.1 Figure 1.a 144
5.4.2 Figure 1.b 144
5.5 Gerstung et al., 2011, pancreatic cancer poset 147
5.6 Raphael and Vandin's 2014 modules 148

6 Running and plotting the simulations: starting, ending, and examples 151
6.1 Starting and ending 151
6.2 Can I start the simulation from a specific mutant? 151
6.3 Ending the simulations 153
6.3.1 Ending the simulations: conditions 154
6.3.2 Stochastic detection mechanism: "detectionProb" 155

6.3.2.1 Stochastic detection mechanism and minimum number of drivers 156
6.3.3 Fixation of genes/gene combinations 157
6.4 Plotting genotype/driver abundance over time; plotting the simulated trajectories 158
6.5 Several examples of simulations and plotting simulation trajectories . 159
6.5.1 Bauer's example again 159
6.5.2 McFarland model with 5000 passengers and 70 drivers 161
6.5.3 McFarland model with 50,000 passengers and 70 drivers: clonal competition 164
6.5.4 Simulation with a conjunction example 166
6.5.5 Simulation with order effects and McFL model 171
6.6 Interactive graphics 178

7 Sampling multiple simulations 179
7.1 Whole-tumor and single-cell sampling, and do we always want to sample? 183
7.2 Differences between "samplePop" and "oncoSimulSample" 184

8 Showing the genealogical relationships of clones 185
8.1 Parent-child relationships from multiple runs 191

9 Generating random fitness landscapes 194

10 Measures of evolutionary predictability and genotype diversity 197

11 Generating random DAGs for restrictions 200

12 FAQ, odds and ends 202
12.1 What we mean by "clone"; and "I want clones disregarding passengers" 202
12.2 Does OncoSimulR keep track of individuals or of clones? And how can it keep track of such large populations? 203
12.2.1 `sampleEvery`, `keepPhylog`, and pruning 203
12.3 Dealing with errors in "oncoSimulPop" 204
12.4 Whole tumor sampling, genotypes, and allele counts: what gives? And what about order? 205
12.5 Doesn't the BNB algorithm require small mutation rates for it to be advantageous? 205
12.6 Can we use the BNB algorithm with state-dependent birth or death rates? 206
12.7 Sometimes I get exceptions when running with mutator genes 206
12.8 What are good values of `sampleEvery`? 207
12.9 What can you do with the simulations? 208

13 Using v.1 posets and simulations 209
13.1 Specifying restrictions: posets 209
13.1.1 Simulating progression in several subjects 216
13.2 Sampling from a set of simulated subjects 216

14 Session info and packages used **219**

15 Funding **220**

16 References **220**

1 Introduction

OncoSimulR is an individual- or clone-based forward-time genetic simulator for biallelic markers (wildtype vs. mutated) in asexually reproducing populations without spatial structure (perfect mixing). Its design emphasizes flexible specification of fitness and mutator effects.

OncoSimulR was originally developed to simulate tumor progression with emphasis on allowing users to set restrictions in the accumulation of mutations as specified, for example, by Oncogenetic Trees (OT: Desper et al. 1999; Szabo and Boucher 2008) or Conjunctive Bayesian Networks (CBN: Beerenwinkel, Eriksson, and Sturmfels 2007; Gerstung et al. 2009; Gerstung et al. 2011), with the possibility of adding passenger mutations to the simulations and allowing for several types of sampling.

Since then, OncoSimulR has been vastly extended to allow you to specify other types of restrictions in the accumulation of genes, such as the XOR models of Korsunsky et al. (2014) or the "semimonotone" model of Farahani and Lagergren (2013). Moreover, different fitness effects related to the order in which mutations appear can also be incorporated, involving arbitrary numbers of genes. This is *very* different from "restrictions in the order of accumulation of mutations". With order effects, described in a recent cancer paper by Ortmann and collaborators (Ortmann et al. 2015), the effect of having both mutations "A" and "B" differs depending on whether "A" appeared before or after "B" (the actual case involves genes JAK2 and TET2).

More generally, OncoSimulR now also allows you to specify arbitrary epistatic interactions between arbitrary collections of genes and to model, for example, synthetic mortality or synthetic viability (again, involving an arbitrary number of genes, some of which might also depend on other genes, or show order effects with other genes). Moreover, it is possible to specify the above interactions in terms of modules, not genes. This idea is discussed in, for example, Raphael and Vandin (2015) and Gerstung et al. (2011): the restrictions encoded in, say, CBNs or OT can be considered to apply not to genes, but to modules, where each module is a set of genes (and the intersection between modules is the empty set) that performs a specific biological function. Modules, then, play the role of a "union operation" over the set of genes in a module. In addition, arbitrary numbers of genes without interactions (and with fitness effects coming from any distribution you might want) are also possible.

Mutator/antimutator genes, genes that alter the mutation rate of other genes (Gerrish et al. 2007; Tomlinson, Novelli, and Bodmer 1996), can also be simulated with OncoSimulR and specified with most of the mechanisms above (you can have, for instance, interactions between mutator genes). And, regardless of the presence or not of other mutator/antimutator genes, different genes can have different mutation rates.

Simulations can be stopped as a function of total population size, number of mutated driver genes, or number of time periods. Simulations can also be stopped with a stochastic detection mechanism where the probability of detecting a tumor increases with total population size. Simulations return the number of cells of every geno-

type/clone at each of the sampling periods and we can take samples from the former with single-cell or whole- tumor resolution, adding noise if we want. If we ask for them, simulations also store and return the genealogical relationships of all clones generated during the simulation.

The models so far implemented are all continuous time models, which are simulated using the BNB algorithm of Mather, Hasty, and Tsimring (2012). The core of the code is implemented in C++, providing for fast execution. To help with simulation studies, code to simulate random graphs of the kind often seen in CBNs, OTs, etc, is also available. Finally, OncoSimulR also allows for the generation of random fitness landscapes and the representation of fitness landscapes and provides statistics of evolutionary predictability.

1.1 Key features of OncoSimulR

As mentioned above, OncoSimulR is now a very general package for forward genetic simulation, with applicability well beyond tumor progression. This is a summary of some of its key features:

- You can specify arbitrary interactions between genes, with arbitrary fitness effects, with explicit support for:
  - Restrictions in the accumulations of mutations, as specified by Oncogenetic Trees (OTs), Conjunctive Bayesian Networks (CBNs), semimonotone progression networks, and XOR relationships.
  - Epistatic interactions including, but not limited to, synthetic viability and synthetic lethality.
  - Order effects.
- You can add passenger mutations.
- You can add mutator/antimutator effects.
- Fitness and mutation rates can be gene-specific.
- You can add arbitrary numbers of non-interacting genes with arbitrary fitness effects.
- you can allow for deviations from the OT, CBN, semimonotone, and XOR models, specifying a penalty for such deviations (the s_h parameter).
- You can conduct multiple simulations, and sample from them with different temporal schemes and using both whole tumor or single cell sampling.
- You can stop the simulations using a flexible combination of conditions: final time, number of drivers, population size, fixation of certain genotypes, and a stochastic stopping mechanism that depends on population size.

- Right now, three different models are available, two that lead to exponential growth, one of them loosely based on Bozic et al. (2010), and another that leads to logistic-like growth, based on McFarland et al. (2013).
- You can use large numbers of genes (e.g., see an example of 50000 in section 6.5.3).
- Simulations are generally very fast: I use C++ to implement the BNB algorithm (see sections 12.5 and 12.6 for more detailed comments on the usage of this algorithm).
- You can obtain the true sequence of events and the phylogenetic relationships between clones (see section 12.1 for the details of what we mean by "clone").
- You can generate random fitness landscapes (under the House of Cards, Rough Mount Fuji, or additive models, or combinations of the former) and use those landscapes as input to the simulation functions.
- You can plot fitness landscapes.
- You can obtain statistics of evolutionary predictability from the simulations.

The table below, modified from the table at the Genetics Simulation Resources (GSR) page, provides a summary of the key features of OncoSimulR. (An explanation of the meaning of terms specific to the GSR table is available from https://popmodels.cancercontrol.cancer.gov/gsr/search/ or from the Genetics Simulation Resources table itself, by moving the mouse over each term).

Table 1: Key features of OncoSimulR. Modified from the original table from https://popmodels.cancercontrol.cancer.gov/gsr/packages/oncosimulr/#detailed .

Attribute Category	Attribute
**Target**	
Type of Simulated Data	Haploid DNA Sequence
Variations	Biallelic Marker, Genotype or Sequencing Error
**Simulation Method**	Forward-time
Type of Dynamical Model	Continuous time
Entities Tracked	Clones (see 12.2)
**Input**	Program specific (R data frames and matrices specifying genotypes' fitness, gene effects, and starting genotype)
**Output**	
Data Type	Genotype or Sequence, Individual Relationship (complete parent-child relationships between clones), Demographic (populations sizes of all clones at sampling times), Diversity Measures (LOD, POM, diversity of genotypes), Fitness
Sample Type	Random or Independent, Longitudinal, Other (proportional to population size)
**Evolutionary Features**	
Mating Scheme	Asexual Reproduction
Demographic	
Population Size Changes	Exponential (two models), Logistic (McFarland et al., 2013)
Fitness Components	
Birth Rate	Individually Determined from Genotype (models "Exp" and "McFL")
Death Rate	Individually Determined from Genotype (model "Bozic"), Influenced by Environment —population size (model "McFL")
Natural Selection	
Determinant	Single and Multi-locus, Fitness of Offspring, Environmental Factors (population size)
Models	Directional Selection, Multi-locus models, Epistasis, Random Fitness Effects
Mutation Models	Two-allele Mutation Model (wildtype, mutant), without back mutation
Events Allowed	Varying Genetic Features: change of individual mutation rates (mutator/antimutator genes)
Spatial Structure	No Spatial Structure (perfectly mixed and no migration)

Further details about the original motivation for wanting to simulate data this way in the context of tumor progression can be found in Diaz-Uriarte (2015), where additional comments about model parameters and caveats are discussed.

Are there similar programs? The Java program by Reiter et al. (2013), TTP, offers somewhat similar functionality to the previous version of OncoSimulR, but it is restricted to at most four drivers (whereas v.1 of OncoSimulR allowed for up to 64), you cannot use arbitrary CBNs or OTs (or XORs or semimonotone graphs) to specify restrictions, there is no allowance for passengers, and a single type of model (a discrete time Galton-Watson process) is implemented. The current functionality of OncoSimulR goes well beyond the the previous version (and, thus, also the TPT of Reiter et al. (2013)). We now allow you to specify all types of fitness effects in other general forward genetic simulators such as FFPopSim (Zanini and Neher 2012), and some that, to our knowledge (e.g., order effects) are not available from any genetics simulator. In addition, the "Lego system" to flexibly combine different fitness specifications is also unique; by "Lego system" I mean that we can combine different pieces and blocks, similarly to what we do with Lego bricks. (I find this an intuitive and very graphical analogy, which I have copied from Hothorn et al. (2006) and Hothorn et al. (2008)). In a nutshell, salient features of OncoSimulR compared to other simulators are the unparalleled flexibility to specify fitness and mutator effects, with modules and order effects as particularly unique, and the options for sampling and stopping the simulations, particularly convenient in cancer evolution models. Also unique in this type of software is the addition of functions for simulating fitness landscapes and assessing evolutionary predictability.

1.2 What kinds of questions is OncoSimulR suited for?

OncoSimulR can be used to address questions that span from the effect of mutator genes in cancer to the interplay between fitness landscapes and mutation rates. The main types of questions that OncoSimulR can help address involve combinations of:

- Simulating asexual evolution (the `oncoSimul*` functions) where:
  - Fitness is:
    - A function of specific epistatic effects between genes
    - A function of order effects
    - A function of epistatic effects specified using DAGs/posets where these DAGs/posets:
      - Are user-specified
      - Generated randomly (`simOGraph`)
    - Any mapping between genotypes and fitness where this mapping is:
      - User-specified
      - Generated randomly from families of random fitness landscapes (`rfitness`)
  - Mutation rates can:
    - Vary between genes

        * Be affected by other genes

- Examining times to evolutionarily or biomedically relevant events (fixation of genotypes, reaching a minimal size, acquiring a minimal number of driver genes, etc —specified with the stopping conditions to the `oncoSimul*` functions).
- Using different sampling schemes (`samplePop`) that are related to:
    - Assessing genotypes from single-cell vs. whole tumor (or whole population) with the `typeSample` argument
    - Genotyping error (`propError` argument)
    - Timing of samples (`timeSample` argument)
    - ... and assessing the consequences of those on the observed genotypes and their diversity (`sampledGenotypes`) and any other inferences that depend on the observational process.
    - (OncoSimulR returns the abundances of all genotypes at each of the sampling points, so you are not restricted by what the `samplePop` function provides.)
- Tracking the genealogical relationships of clones (`plotClonePhylog`) and assessing evolutionary predictability (`LOD`, `POM`).

Some specific questions that you can address with the help of OncoSimulR are discussed in section 1.3.

A quick overview of the main functions and their relationships is shown in Figure 1, where we use italics for the type/class of R object and courier font for the name of the functions.

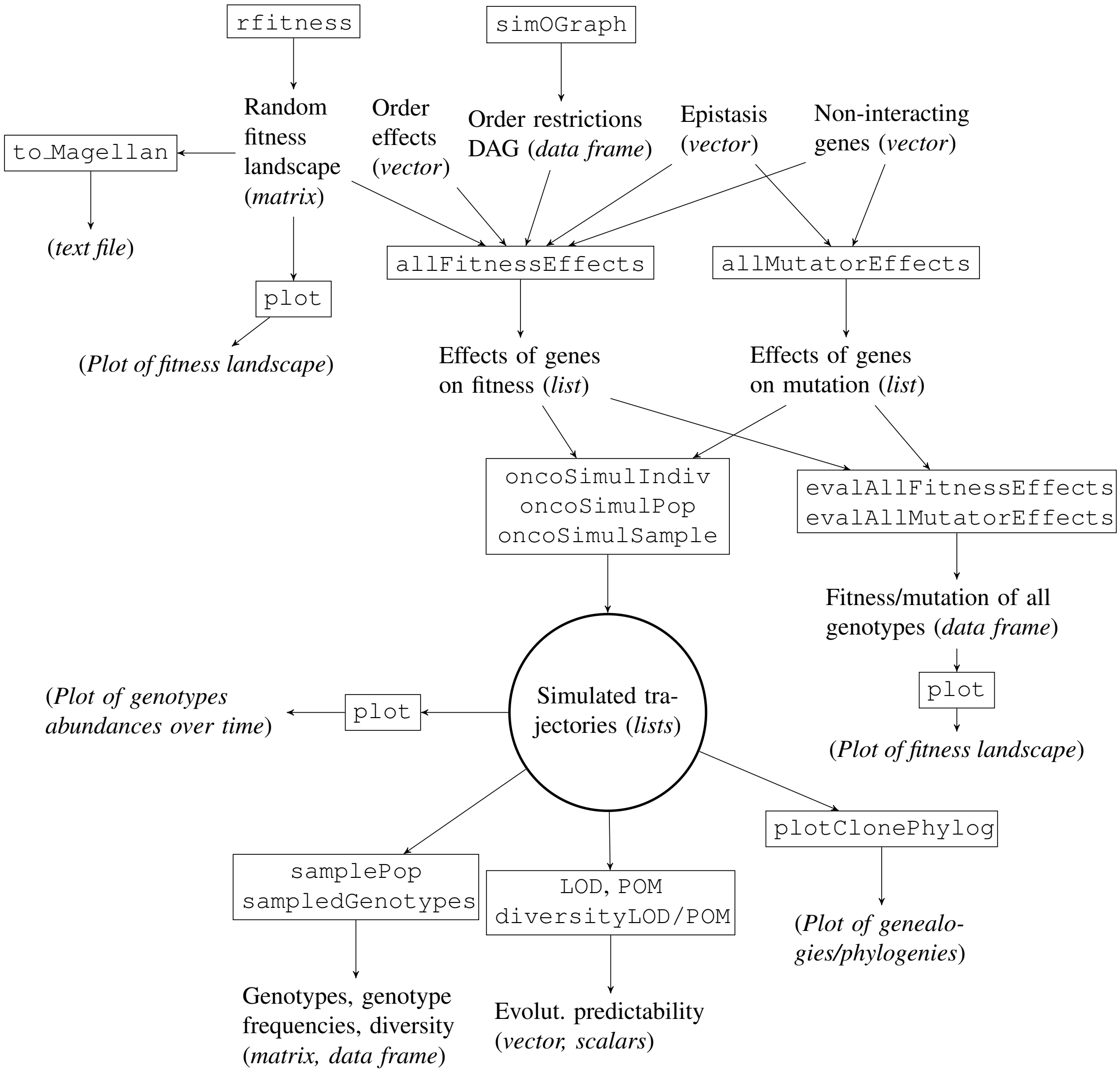

Figure 1: Relationships between the main functions in OncoSimulR.

1.3 Examples of questions that can be addressed with OncoSimulR

Most of the examples in the rest of this vignette, starting with those in 1.6, focus on the mechanics. Here, we will illustrate some problems in cancer genomics and evolutionary genetics where OncoSimulR could be of help. This section does not try to provide an answer to any of these questions (those would be full papers by themselves). Instead, this section simply tries to illustrate some kinds of questions where you can use OncoSimulR; of course, the possible uses of OncoSimulR are only limited by your ingenuity. Here, I will only use short snippets of working code as we are limited by time of execution; for real work you would want to use many more scenarios and many more simulations, you would use appropriate statistical methods to compare the output of runs, etc, etc, etc.

```
## Load the package
library(OncoSimulR)
```

1.3.1 Recovering restrictions in the order of accumulation of mutations

This is a question that was addressed, for instance, in Diaz-Uriarte (2015): do methods that try to infer restrictions in the order of accumulation of mutations (e.g., Szabo and Boucher 2008; Gerstung et al. 2009; Ramazzotti et al. 2015) work well under different evolutionary models and with different sampling schemes? (This issue is also touched upon in section 13.2).

A possible way to examine that question would involve:

- generating random DAGs that encode restrictions;
- simulating cancer evolution using those DAGs;
- sampling the data and adding different levels of noise to the sampled data;
- running the inferential method;
- comparing the inferred DAG with the original, true, one.

```
## For reproducibility
set.seed(2)
RNGkind("L'Ecuyer-CMRG")

## Simulate a DAG
g1 <- simOGraph(4, out = "rT")

## Simulate 10 evolutionary trajectories
s1 <- oncoSimulPop(10, allFitnessEffects(g1, drvNames = 1:4),
                   mc.cores = 2, ## adapt to your hardware
                   seed = NULL) ## for reproducibility of vignette

## Sample those data uniformly, and add noise
d1 <- samplePop(s1, timeSample = "unif", propError = 0.1)
```

```
##
##  Subjects by Genes matrix of 10 subjects and 4 genes.

## You would now run the appropriate inferential method and
## compare observed and true. For example

## require(Oncotree)
## fit1 <- oncotree.fit(d1)

## Now, you'd compare fitted and original. This is well beyond
## the scope of this document (and OncoSimulR itself).
```

1.3.2 Sign epistasis and probability of crossing fitness valleys

This question, and the question in the next section (1.3.3), encompass a wide range of issues that have been addressed in evolutionary genetics studies and which include from detailed analysis of simple models with a few uphill paths and valleys as in Weissman et al. (2009) or Ochs and Desai (2015), to questions that refer to larger, more complex fitness landscapes as in Szendro, Franke, et al. (2013) or Franke et al. (2011) (see below).

Using as an example Ochs and Desai (2015) (we will see this example again in section 5.3, where we cover different ways of specifying fitness), we could specify the fitness landscape and run simulations until fixation (with argument `fixation` to `oncoSimulPop` —see more details in section 6.3.3, again with this example). We would then examine the proportion of genotypes fixed under different scenarios. And we can extend this example by adding mutator genes:

```
## For reproducibility
set.seed(2)
RNGkind("L'Ecuyer-CMRG")

## Specify fitness effects.

## Numeric values arbitrary, but set the intermediate genotype en
## route to ui as mildly deleterious so there is a valley.

## As in Ochs and Desai, the ui and uv genotypes
## can never appear.

u <- 0.2; i <- -0.02; vi <- 0.6; ui <- uv <- -Inf

od <- allFitnessEffects(
    epistasis = c("u" = u,  "u:i" = ui,
                  "u:v" = uv, "i" = i,
                  "v:-i" = -Inf, "v:i" = vi))
```

```r
## For the sake of extending this example, also turn i into a
## mutator gene

odm <- allMutatorEffects(noIntGenes = c("i" = 50))

## How do mutation and fitness look like for each genotype?
evalAllGenotypesFitAndMut(od, odm, addwt = TRUE)
##   Genotype Fitness MutatorFactor
## 1       WT   1.000             1
## 2        i   0.980            50
## 3        u   1.200             1
## 4        v   0.000             1
## 5     i, u   0.000            50
## 6     i, v   1.568            50
## 7     u, v   0.000             1
## 8  i, u, v   0.000            50
```

Ochs and Desai explicitly say "Each simulated population was evolved until either the uphill genotype or valley-crossing genotype fixed." So we will use `fixation`.

```r
## Set a small initSize, as o.w. unlikely to pass the valley
initS <- 10
## The number of replicates is tiny, 10, for the sake of speed
## of creation of the vignette
od_sim <- oncoSimulPop(10, od, muEF = odm,
                       fixation = c("u", "i, v"), initSize = initS,
                       model = "McFL",
                       mu = 1e-4, detectionDrivers = NA,
                       finalTime = NA,
                       detectionSize = NA, detectionProb = NA,
                       onlyCancer = TRUE,
                       mc.cores = 2, ## adapt to your hardware
                       seed = NULL) ## for reproducibility
## What is the frequency of each final genotype?
sampledGenotypes(samplePop(od_sim))
##
##  Subjects by Genes matrix of 10 subjects and 3 genes.
##   Genotype Freq
## 1     i, v    5
## 2        u    5
##
##  Shannon's diversity (entropy) of sampled genotypes: 0.6931
```

1.3.3 Predictability of evolution in complex fitness landscapes

Focusing now on predictability in more general fitness landscapes, we would run simulations under random fitness landscapes with varied ruggedness, and would then examine the evolutionary predictability of the trajectories with measures such as "Lines of Descent" and "Path of the Maximum" (Szendro, Franke, et al. 2013) and the diversity of the sampled genotypes under different sampling regimes (see details in section 10).

```
## For reproducibility
set.seed(7)
RNGkind("L'Ecuyer-CMRG")



## Repeat the following loop for different combinations of whatever
## interests you, such as number of genes, or distribution of the
## c and sd (which affect how rugged the landscape is), or
## reference genotype, or evolutionary model, or stopping criterion,
## or sampling procedure, or ...

##  Generate a random fitness landscape, from the Rough Mount
##  Fuji model, with g genes, and c ("slope" constant) and
##  reference chosen randomly (reference is random by default and
##  thus not specified below). Require a minimal number of
##  accessible genotypes

g <- 6
c <- runif(1, 1/5, 5)
rl <- rfitness(g, c = c, min_accessible_genotypes = g)

## Plot it if you want; commented here as it takes long for a
## vignette

## plot(rl)

## Obtain landscape measures from Magellan. Export to Magellan
to_Magellan(rl, file = "rl1.txt")

## (Getting the statistics from Magellan requires either
## calling the web app (http://wwwabi.snv.jussieu.fr/public/Magellan/)
## or asking Magellan's authors for the software. This is of course
## beyond the scope of the example and the package.)

## Simulate evolution in that landscape many times (here just 10)
simulrl <- oncoSimulPop(10, allFitnessEffects(genotFitness = rl),
                        keepPhylog = TRUE, keepEvery = 1,
                        initSize = 4000,
```

```
                        seed = NULL, ## for reproducibility
                        mc.cores = 2) ## adapt to your hardware

## Obtain measures of evolutionary predictability
diversityLOD(LOD(simulrl))
## [1] 1.418
diversityPOM(POM(simulrl))
## [1] 1.643
sampledGenotypes(samplePop(simulrl, typeSample = "whole"))
##
##  Subjects by Genes matrix of 10 subjects and 6 genes.
##   Genotype Freq
## 1        A    3
## 2     A, E    1
## 3     A, F    2
## 4     E, F    4
##
##  Shannon's diversity (entropy) of sampled genotypes: 1.28
```

1.3.4 Mutator and antimutator genes

The effects of mutator and antimutator genes have been examined both in cancer genetics (Nowak 2006; Tomlinson, Novelli, and Bodmer 1996) and in evolutionary genetics (Gerrish et al. 2007), and are related to wider issues such as Muller's ratchet and the evolution of sex. There are, thus, a large range of questions related to mutator and antimutator genes.

One question addressed in Tomlinson, Novelli, and Bodmer (1996) concerns under what circumstances mutator genes are likely to play a role in cancer progression. For instance, Tomlinson, Novelli, and Bodmer (1996) find that an increased mutation rate is more likely to matter if the number of required mutations in driver genes needed to reach cancer is large and if the mutator effect is large.

We might want to ask, then, how long it takes before to reach cancer under different scenarios. Time to reach cancer is stored in the component `FinalTime` of the output. We would specify different numbers and effects of mutator genes (argument `muEF`). We would also change the criteria for reaching cancer and in our case we can easily do that by specifying different numbers in `detectionDrivers`. Of course, we would also want to examine the effects of varying numbers of mutators, drivers, and possibly fitness consequences of mutators. Below we assume mutators are neutral and we assume there are no additional genes with deleterious mutations, but this need not be so, of course (see also Tomlinson, Novelli, and Bodmer 1996; Gerrish et al. 2007; McFarland, Mirny, and Korolev 2014).

Let us run an example. For the sake of simplicity, we assume no epistatic interactions.

```r
sd <- 0.1 ## fitness effect of drivers
sm <- 0 ## fitness effect of mutator
nd <- 20 ## number of drivers
nm <- 5  ## number of mutators
mut <- 10 ## mutator effect

fitnessGenesVector <- c(rep(sd, nd), rep(sm, nm))
names(fitnessGenesVector) <- 1:(nd + nm)
mutatorGenesVector <- rep(mut, nm)
names(mutatorGenesVector) <- (nd + 1):(nd + nm)

ft <- allFitnessEffects(noIntGenes = fitnessGenesVector,
                        drvNames = 1:nd)
mt <- allMutatorEffects(noIntGenes = mutatorGenesVector)
```

Now, simulate using the fitness and mutator specification. We fix the number of drivers to cancer, and we stop when those numbers of drivers are reached. Since we only care about the time it takes to reach cancer, not the actual trajectories, we set `keepEvery = NA`:

```r
## For reproducibility
set.seed(2)
RNGkind("L'Ecuyer-CMRG")


ddr <- 4
st <- oncoSimulPop(4, ft, muEF = mt,
                   detectionDrivers = ddr,
                   finalTime = NA,
                   detectionSize = NA,
                   detectionProb = NA,
                   onlyCancer = TRUE,
                   keepEvery = NA,
                   mc.cores = 2, ## adapt to your hardware
                   seed = NULL) ## for reproducibility

## How long did it take to reach cancer?
unlist(lapply(st, function(x) x$FinalTime))
## [1]  370  141 1793  282
```

(Incidentally, notice that it is easy to get OncoSimulR to throw an exception if you accidentally specify a huge mutation rate when all mutator genes are mutated: see section 12.7.)

1.3.5 Epistatic interactions between drivers and passengers in cancer and the consequences of order effects

1.3.5.1 Epistatic interactions between drivers and passengers

Bauer, Siebert, and Traulsen (2014) have examined the effects of epistatic relationships between drivers and passengers in cancer initiation. We could use their model as a starting point, and examine how likely cancer is to develop under different variations of their model and different evolutionary scenarios (e.g., initial sample size, mutation rates, evolutionary model, etc).

There are several ways to specify their model, as we discuss in section 5.1. We will use one based on DAGs here:

```
K <- 4
sp <- 1e-5
sdp <- 0.015
sdplus <- 0.05
sdminus <- 0.1

cnt <- (1 + sdplus)/(1 + sdminus)
prod_cnt <- cnt - 1
bauer <- data.frame(parent = c("Root", rep("D", K)),
                    child = c("D", paste0("s", 1:K)),
                    s = c(prod_cnt, rep(sdp, K)),
                    sh = c(0, rep(sp, K)),
                    typeDep = "MN")
fbauer <- allFitnessEffects(bauer)
(b1 <- evalAllGenotypes(fbauer, order = FALSE, addwt = TRUE))
##              Genotype Fitness
## 1                  WT  1.0000
## 2                   D  0.9545
## 3                  s1  1.0000
## 4                  s2  1.0000
## 5                  s3  1.0000
## 6                  s4  1.0000
## 7               D, s1  0.9689
## 8               D, s2  0.9689
## 9               D, s3  0.9689
## 10              D, s4  0.9689
## 11             s1, s2  1.0000
## 12             s1, s3  1.0000
## 13             s1, s4  1.0000
## 14             s2, s3  1.0000
## 15             s2, s4  1.0000
## 16             s3, s4  1.0000
## 17         D, s1, s2  0.9834
## 18         D, s1, s3  0.9834
```

```
## 19         D, s1, s4  0.9834
## 20         D, s2, s3  0.9834
## 21         D, s2, s4  0.9834
## 22         D, s3, s4  0.9834
## 23        s1, s2, s3  1.0000
## 24        s1, s2, s4  1.0000
## 25        s1, s3, s4  1.0000
## 26        s2, s3, s4  1.0000
## 27     D, s1, s2, s3  0.9981
## 28     D, s1, s2, s4  0.9981
## 29     D, s1, s3, s4  0.9981
## 30     D, s2, s3, s4  0.9981
## 31    s1, s2, s3, s4  1.0000
## 32 D, s1, s2, s3, s4  1.0131

## How does the fitness landscape look like?
plot(b1, use_ggrepel = TRUE) ## avoid overlapping labels
```

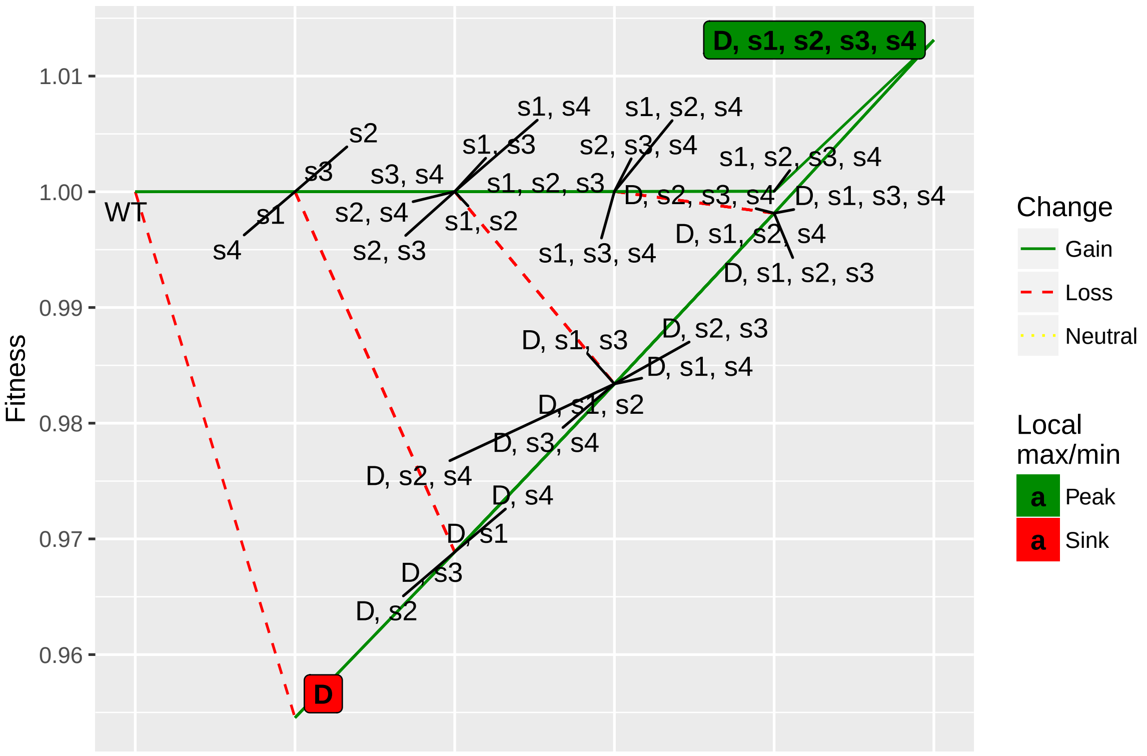

Now run simulations and examine how frequently the runs end up with population sizes larger than a pre-specified threshold; for instance, below we look at increasing population size 4x in the default maximum number of 2281 time periods (for real, you would of course increase the number of total populations, the range of initial population sizes, model, mutation rate, required population size or number of drivers, etc):

```
## For reproducibility
set.seed(2)
RNGkind("L'Ecuyer-CMRG")

totalpops <- 5
initSize <- 100
sb1 <- oncoSimulPop(totalpops, fbauer, model = "Exp",
                    initSize = initSize,
                    onlyCancer = FALSE,
                    mc.cores = 2, ## adapt to your hardware
                    seed = NULL) ## for reproducibility

## What proportion of the simulations reach 4x initSize?
sum(summary(sb1)[, "TotalPopSize"] > (4 * initSize))/totalpops
## [1] 0.2
```

Alternatively, to examine how long it takes to reach cancer for a pre-specified size, you could look at the value of `FinalTime` as we did above (section 1.3.4) after running simulations with `onlyCancer = TRUE` and `detectionSize` set to some reasonable value:

```
totalpops <- 5
initSize <- 100
sb2 <- oncoSimulPop(totalpops, fbauer, model = "Exp",
                    initSize = initSize,
                    onlyCancer = TRUE,
                    detectionSize = 10 * initSize,
                    mc.cores = 2, ## adapt to your hardware
                    seed = NULL) ## for reproducibility

## How long did it take to reach cancer?
unlist(lapply(sb2, function(x) x$FinalTime))
## [1] 416 354 339 445 215
```

1.3.5.2 Consequences of order effects for cancer initiation

Instead of focusing on different models for epistatic interactions, you might want to examine the consequences of order effects (Ortmann et al. 2015). You would proceed as above, but using models that differ by, say, the presence or absence of order effects. Details on their specification are provided in section 3.6. Here is one particular model (you would, of course, want to compare this to models without order effects or with other magnitudes and types of order effects):

```
## Order effects involving three genes.

## Genotype "D, M" has different fitness effects
```

```
## depending on whether M or D mutated first.
## Ditto for genotype "F, D, M".

## Meaning of specification: X > Y means
## that X is mutated before Y.


o3 <- allFitnessEffects(orderEffects = c(
                            "F > D > M" = -0.3,
                            "D > F > M" = 0.4,
                            "D > M > F" = 0.2,
                            "D > M"     = 0.1,
                            "M > D"     = 0.5))

## With the above specification, let's double check
## the fitness of the possible genotypes

(oeag <- evalAllGenotypes(o3, addwt = TRUE, order = TRUE))
##     Genotype Fitness
## 1          WT    1.00
## 2           D    1.00
## 3           F    1.00
## 4           M    1.00
## 5       D > F    1.00
## 6       D > M    1.10
## 7       F > D    1.00
## 8       F > M    1.00
## 9       M > D    1.50
## 10      M > F    1.00
## 11 D > F > M    1.54
## 12 D > M > F    1.32
## 13 F > D > M    0.77
## 14 F > M > D    1.50
## 15 M > D > F    1.50
## 16 M > F > D    1.50
```

Now, run simulations and examine how frequently the runs do not end up in extinction. As above, for real, you would of course increase the number of total populations, the range of initial population sizes, mutation rate, etc:

```
## For reproducibility
set.seed(2)
RNGkind("L'Ecuyer-CMRG")

totalpops <- 5
soe1 <- oncoSimulPop(totalpops, o3, model = "Exp",
                     initSize = 500,
```

```
                          onlyCancer = FALSE,
                          mc.cores = 2, ## adapt to your hardware
                          seed = NULL) ## for reproducibility

## What proportion of the simulations do not end up extinct?
sum(summary(soe1)[, "TotalPopSize"] > 0)/totalpops
## [1] 0.4
```

As we just said, alternatively, to examine how long it takes to reach cancer you could run simulations with `onlyCancer = TRUE` and look at the value of `FinalTime` as we did above (section 1.3.4).

1.4 Trade-offs and what is OncoSimulR not well suited for

OncoSimulR is designed for complex fitness specifications and selection scenarios and uses forward-time simulations; the types of questions where OncoSimulR can be of help are discussed in sections 1.2 and 1.3 and running time and space consumption of OncoSimulR are addressed in section 2. You should be aware that **coalescent simulations**, sometimes also called backward-time simulations, are much more efficient for simulating neutral data as well as some special selection scenarios (Yuan et al. 2012; Carvajal-Rodriguez 2010; Hoban, Bertorelle, and Gaggiotti 2011).

In addition, since OncoSimulR allows you to specify fitness with arbitrary epistatic and order effects, as well as mutator effects, you need to learn the syntax of how to specify those effects and you might be paying a performance penalty if your scenario does not require this complexity. For instance, in the model of Beerenwinkel et al. (2007), the fitness of a genotype depends only on the total number of drivers mutated, but not on which drivers are mutated (and, thus, not on the epistatic interactions nor the order of accumulation of the drivers). This means that the syntax for specifying that model could probably be a lot simpler (e.g., specify s per driver).

But it also means that code written for just that case could probably run much faster. First, because fitness evaluation is easier. Second, and possibly much more important, because what we need to keep track of leads to much simpler and economic structures: we do not need to keep track of clones (where two cells are regarded as different clones if they differ anywhere in their genotype), but only of clone types or clone classes as defined by the number of mutated drivers, and keeping track of clones can be expensive —see sections 2 and 12.2.

So for those cases where you do not need the full flexibility of OncoSimulR, special purpose software might be easier to use and faster to run. Of course, for some types of problems this special purpose software might not be available, though.

1.5 Steps for using OncoSimulR

Using this package will often involve the following steps:

1. Specify fitness effects: sections 3 and 5.
2. Simulate cancer progression: section 6. You can simulate for a single individual or subject or for a set of subjects. You will need to:
   - Decide on a model. This basically amounts to choosing a model with exponential growth ("Exp" or "Bozic") or a model with carrying capacity ("McFL"). If exponential growth, you can choose whether the the effects of mutations operate on the death rate ("Bozic") or the birth rate ("Exp")[1].
   - Specify other parameters of the simulation. In particular, decide when to stop the simulation, mutation rates, etc.

   Of course, at least for initial playing around, you can use the defaults.
3. Sample from the simulated data and do something with those simulated data (e.g., fit an OT model to them, examine diversity or time until cancer, etc). Most of what you do with the data, however, is outside the scope of this package and this vignette.

Before anything else, let us load the package in case it was not yet loaded. We also explicitly load *graph* and *igraph* for the vignette to work (you do not need that for your usual interactive work). And I set the default color for vertices in igraph.

```
library(OncoSimulR)
library(graph)
library(igraph)
igraph_options(vertex.color = "SkyBlue2")
```

To be explicit, what version are we running?

```
packageVersion("OncoSimulR")
## [1] '2.5.9'
```

1.6 Two quick examples of fitness specifications

Following 1.5 we will run two very minimal examples. First a model with a few genes and **epistasis**:

```
## 1. Fitness effects: here we specify an
##    epistatic model with modules.
sa <- 0.1
sb <- -0.2
sab <- 0.25
sac <- -0.1
sbc <- 0.25
```

[1] It is of course possible to do this with the carrying capacity (or gompertz-like) models, but there probably is little reason to do it. McFarland et al. (2013) discuss this has little effect on their results, for example. In addition, decreasing the death rate will more easily lead to numerical problems as shown in section 3.11.2.

```r
sv2 <- allFitnessEffects(epistasis = c("-A : B" = sb,
                                       "A : -B" = sa,
                                       "A : C" = sac,
                                       "A:B" = sab,
                                       "-A:B:C" = sbc),
                         geneToModule = c(
                             "A" = "a1, a2",
                             "B" = "b",
                             "C" = "c"),
                         drvNames = c("a1", "a2", "b", "c"))
evalAllGenotypes(sv2, addwt = TRUE)
##        Genotype Fitness
## 1            WT   1.000
## 2            a1   1.100
## 3            a2   1.100
## 4             b   0.800
## 5             c   1.000
## 6        a1, a2   1.100
## 7         a1, b   1.250
## 8         a1, c   0.990
## 9         a2, b   1.250
## 10        a2, c   0.990
## 11         b, c   1.000
## 12    a1, a2, b   1.250
## 13    a1, a2, c   0.990
## 14     a1, b, c   1.125
## 15     a2, b, c   1.125
## 16 a1, a2, b, c   1.125

## 2. Simulate the data. Here we use the "McFL" model and set
##    explicitly parameters for mutation rate, initial size, size
##    of the population that will end the simulations, etc

RNGkind("Mersenne-Twister")
set.seed(983)
ep1 <- oncoSimulIndiv(sv2, model = "McFL",
                      mu = 5e-6,
                      sampleEvery = 0.025,
                      keepEvery = 0.5,
                      initSize = 2000,
                      finalTime = 3000,
                      onlyCancer = FALSE)
```

```r
## 3. We will not analyze those data any further. We will only plot
## them.  For the sake of a small plot, we thin the data.
plot(ep1, show = "drivers", xlim = c(0, 1500),
```

```
      thinData = TRUE, thinData.keep = 0.5)
```

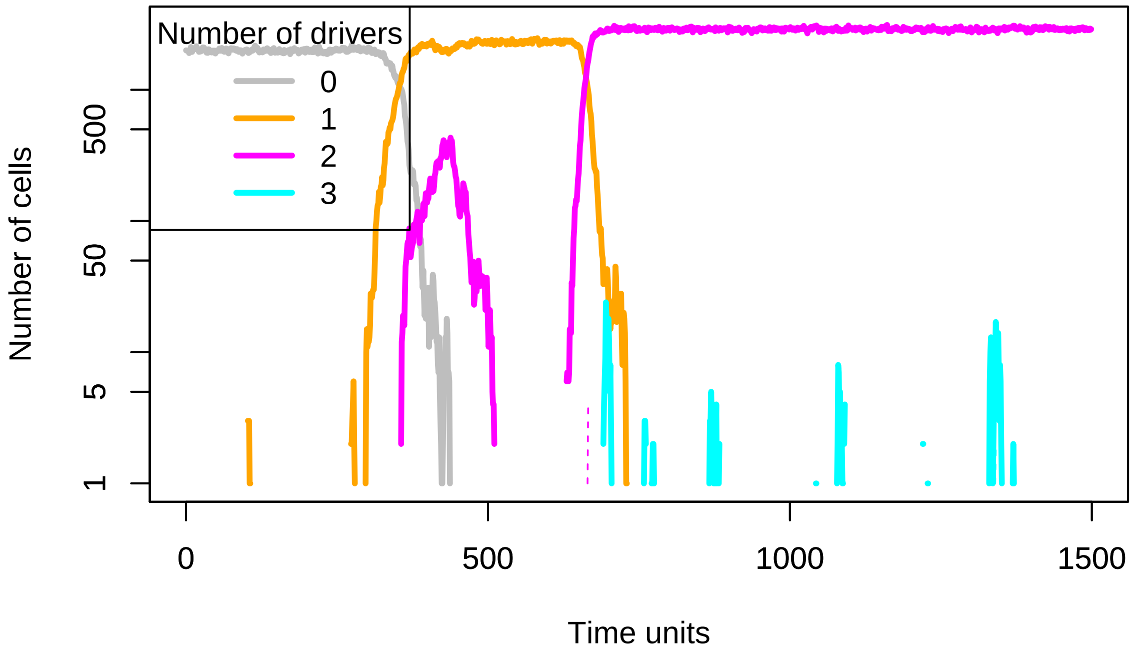

Figure 2: Plot of drivers of an epistasis simulation.

As a second example, we will use a model where we specify **restrictions in the order of accumulation of mutations using a DAG** with the pancreatic cancer poset in Gerstung et al. (2011) (see more details in section 5.5):

```
## 1. Fitness effects:
pancr <- allFitnessEffects(
    data.frame(parent = c("Root", rep("KRAS", 4),
                          "SMAD4", "CDNK2A",
                          "TP53", "TP53", "MLL3"),
                  child = c("KRAS","SMAD4", "CDNK2A",
                          "TP53", "MLL3",
                          rep("PXDN", 3), rep("TGFBR2", 2)),
                  s = 0.1,
                  sh = -0.9,
                  typeDep = "MN"),
    drvNames = c("KRAS", "SMAD4", "CDNK2A", "TP53",
                 "MLL3", "TGFBR2", "PXDN"))
```

```
## Plot the DAG of the fitnessEffects object
plot(pancr)
```

```
## 2. Simulate from it. We change several possible options.

set.seed(4) ## Fix the seed, so we can repeat it
ep2 <- oncoSimulIndiv(pancr, model = "McFL",
                      mu = 1e-6,
```

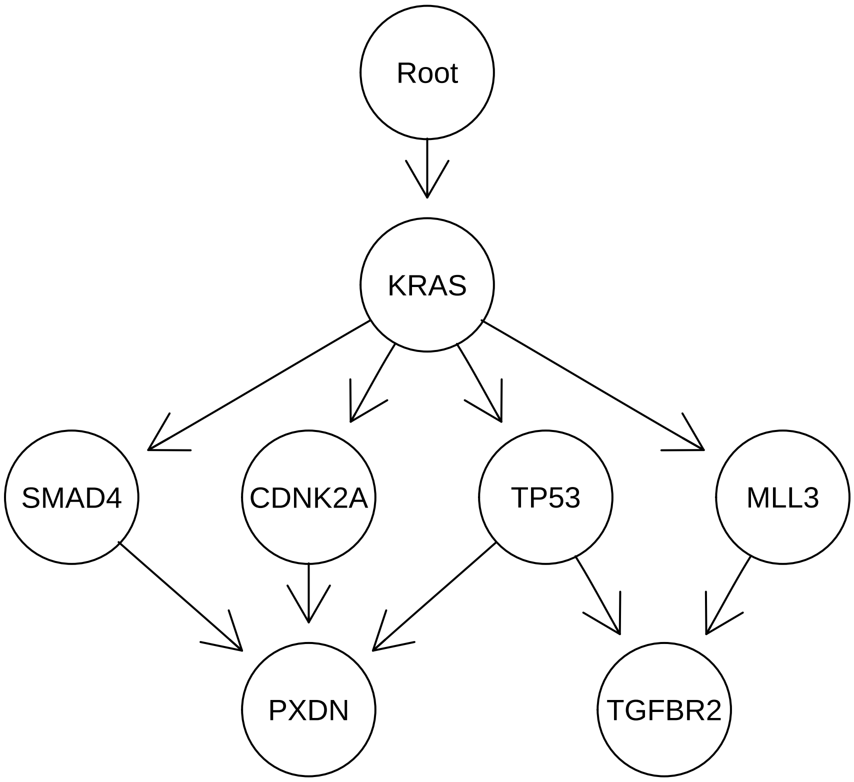

Figure 3: Plot of DAG corresponding to fitnessEffects object.

```
                      sampleEvery = 0.02,
                      keepEvery = 1,
                      initSize = 1000,
                      finalTime = 10000,
                      onlyCancer = FALSE)
```

```
## 3. What genotypes and drivers we get? And play with limits
##    to show only parts of the data. We also aggressively thin
##    the data.
par(cex = 0.7)
plot(ep2, show = "genotypes", xlim = c(1000, 8000),
     ylim = c(0, 2400),
     thinData = TRUE, thinData.keep = 0.03)
```

The rest of this vignette explores all of those functions and arguments in much more detail.

1.7 Citing OncoSimulR and other documentation

In R, you can do

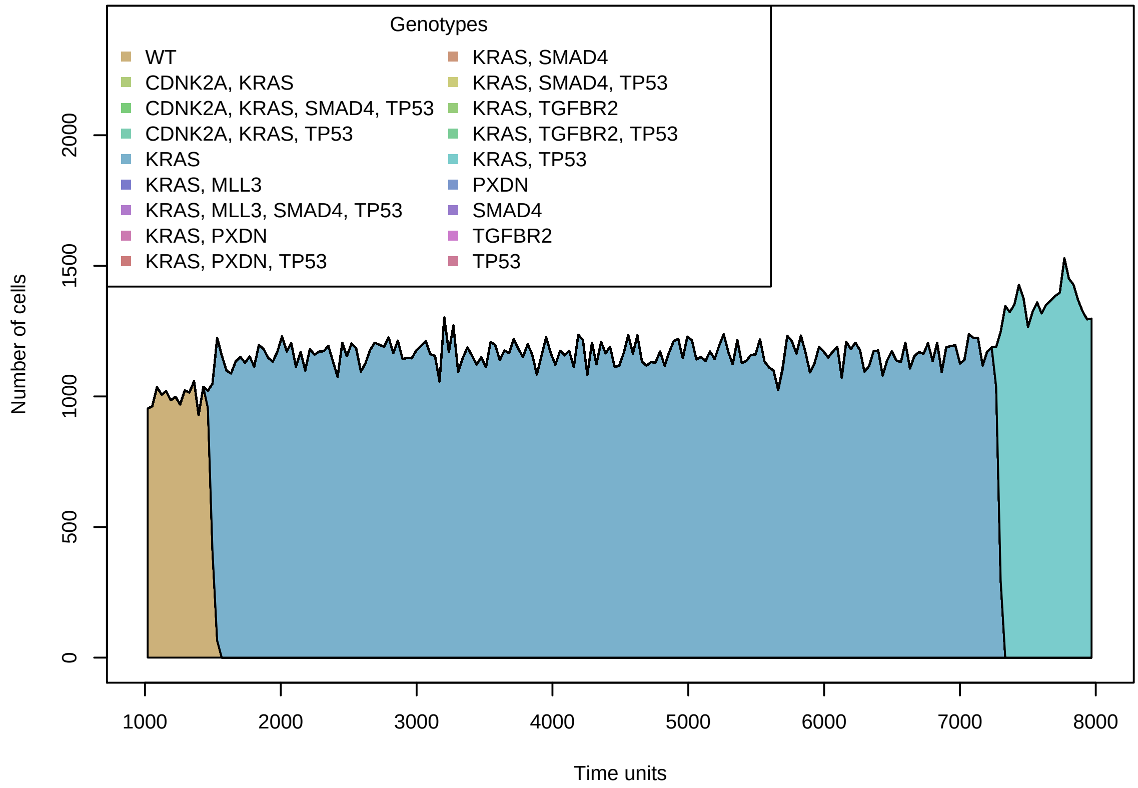

Figure 4: Plot of genotypes of a simulation from a DAG.

```
citation("OncoSimulR")
##
## If you use OncoSimulR, please cite the OncoSimulR bioRxiv
## paper.  A former version of OncoSimulR has been used in a
## large comparative study of methods to infer restrictions,
## published in BMC Bioinformatics; you might want to cite that
## too, if appropriate, such as when referring to using
## evolutionary simulations to assess oncogenetic tree methods
## performance.
##
##   R Diaz-Uriarte. OncoSimulR: genetic simulation of cancer
##   progression with arbitrary epistasis and mutator genes.
##   2016. bioRxiv, http://dx.doi.org/10.1101/069500.
##
##   R Diaz-Uriarte. Identifying restrictions in the order of
##   accumulation of mutations during tumor progression:
##   effects of passengers, evolutionary models, and sampling
##   BMC Bioinformatics, 16(41), 2015.
```

which will tell you how to cite the package.

This is the URL for the bioRxiv paper: http://biorxiv.org/content/early/2016/08/

14/069500.

1.7.1 HTML and PDF versions of the vignette

A PDF version of this vignette is available from https://rdiaz02.github.io/OncoSimul/pdfs/OncoSimulR.pdf. And an HTML version from https://rdiaz02.github.io/OncoSimul/OncoSimulR.html. These files should correspond to the most recent, GitHub version, of the package (i.e., they might include changes not yet available from the BioConductor package).

1.8 Testing, code coverage, and other examples

OncoSimulR includes more than 2000 tests that are run at every check cycle. These tests provide a code coverage of more than 90% including both the C++ and R code. Another set of over 500 long-running (several hours) tests can be run on demand (see directory '/tests/manual'). In addition to serving as test cases, some of that code also provides further examples of usage.

1.9 Versions

In this vignette and the documentation I often refer to version 1 (v.1) and version 2 of OncoSimulR. Version 1 is the version available up to, and including, BioConductor v. 3.1. Version 2 of OncoSimulR is available starting from BioConductor 3.2 (and, of course, available too from development versions of BioC). So, if you are using the current stable or development version of BioConductor, or you grab the sources from GitHub (https://github.com/rdiaz02/OncoSimul) you are using what we call version 2. Please note that **the functionality of version 1 will soon be removed.**

2 Running time and space consumption of OncoSimulR

Time to complete the simulations and size of returned objects (space consumption) depend on several, interacting factors. The usual rule of "experiment before launching a large number of simulations" applies, but here we will walk through several cases to get a feeling for the major factors that affect speed and size. Many of the comments on this section need to use ideas discussed in other places of this document; if you read this section first, you might want to come back after reading the relevant parts.

Speed will depend on:

- Your hardware, of course.
- The evolutionary model.
- The granularity of how often you keep data (`keepEvery` argument). Note that the default, which is to keep as often as you sample (so that we preserve all history) can lead to slow execution times.
- The mutation rate, because higher mutation rates lead to more clones, and more clones means we need to iterate over, well, more clones, and keep larger data structures.
- The fitness specification: more complex fitness specifications tend to be slightly slower but specially different fitness specifications can have radically different effects on the evolutionary trajectories, accessibility of fast growing genotypes and, generally, the evolutionary dynamics.
- The stopping conditions (`detectionProb`, `detectionDrivers`, `detectionSize` arguments) and whether or not simulations are run until cancer is reached (`onlyCancer` argument).
- Most of the above factors can interact in complex ways.

Size of returned objects will depend on:

- Any factor that affects the number of clones tracked/returned, in particular: initial sizes and stopping conditions, mutation rate, and how often you keep data (the `keepEvery` argument can make a huge difference here).
- Whether or not you keep the complete genealogy of clones (this affects slightly the size of returned object, not speed).

In the sections that follow, we go over several cases to understand some of the main settings that affect running time (or execution time) and space consumption (the size of returned objects). It should be understood, however, that many of the examples shown below do not represent typical use cases of OncoSimulR and are used only to identify what and how affects running time and space consumption. As we will see in most examples in this vignette, typical use cases of OncoSimulR involve hundreds to thousands of genes on population sizes up to 10^5 to 10^7.

Note that most of the code in this section is not executed during the building of the vignette to keep vignette build time reasonable and prevent using huge amounts of RAM. All of the code, ready to be sourced and run, is available from the 'inst/miscell'

directory (and the summary output from some of the benchmarks is available from the 'miscell-files/vignette_bench_Rout' directory of the main OncoSimul repository at https://github.com/rdiaz02/OncoSimul).

2.1 Exp and McFL with "detectionProb" and pancreas example

To get familiar with some of they factors that affect time and size, we will use the fitness specification from section 1.6, with the `detectionProb` stopping mechanism (see 6.3.2). We will use the two main growth models (exponential and McFarland). Each model will be run with two settings of `keepEvery`. With `keepEvery = 1` (runs `exp1` and `mc1`), population samples are stored at time intervals of 1 (even if most of the clones in those samples later become extinct). With `keepEvery = NA` (runs `exp2` and `mc2`) no intermediate population samples are stored, so clones that become extinct at any sampling period are pruned and only the existing clones at the end of the simulation are returned (see details in 12.2.1).

Will run 100 simulations. The results I show are for a laptop with an 8-core Intel Xeon E3-1505M CPU, running Debian GNU/Linux (the results from these benchmarks are available as `data(benchmark_1)`).

```
## Specify fitness
pancr <- allFitnessEffects(
    data.frame(parent = c("Root", rep("KRAS", 4),
                          "SMAD4", "CDNK2A",
                          "TP53", "TP53", "MLL3"),
               child = c("KRAS","SMAD4", "CDNK2A",
                          "TP53", "MLL3",
                          rep("PXDN", 3), rep("TGFBR2", 2)),
               s = 0.1,
               sh = -0.9,
               typeDep = "MN"),
    drvNames = c("KRAS", "SMAD4", "CDNK2A", "TP53",
                 "MLL3", "TGFBR2", "PXDN"))

Nindiv <- 100 ## Number of simulations run.
              ## Increase this number to decrease sampling variation

## keepEvery = 1
t_exp1 <- system.time(
    exp1 <- oncoSimulPop(Nindiv, pancr,
                         detectionProb = "default",
                         detectionSize = NA,
                         detectionDrivers = NA,
                         finalTime = NA,
                         keepEvery = 1,
```

```
                            model = "Exp",
                            mc.cores = 1))["elapsed"]/Nindiv


t_mc1 <- system.time(
    mc1 <- oncoSimulPop(Nindiv, pancr,
                           detectionProb = "default",
                           detectionSize = NA,
                           detectionDrivers = NA,
                           finalTime = NA,
                           keepEvery = 1,
                           model = "McFL",
                           mc.cores = 1))["elapsed"]/Nindiv

## keepEvery = NA
t_exp2 <- system.time(
    exp2 <- oncoSimulPop(Nindiv, pancr,
                            detectionProb = "default",
                            detectionSize = NA,
                            detectionDrivers = NA,
                            finalTime = NA,
                            keepEvery = NA,
                            model = "Exp",
                            mc.cores = 1))["elapsed"]/Nindiv


t_mc2 <- system.time(
    mc2 <- oncoSimulPop(Nindiv, pancr,
                           detectionProb = "default",
                           detectionSize = NA,
                           detectionDrivers = NA,
                           finalTime = NA,
                           keepEvery = NA,
                           model = "McFL",
                           mc.cores = 1))["elapsed"]/Nindiv
```

We can obtain times, sizes of objects, and summaries of numbers of clones, iterations, and final times doing, for instance:

```
cat("\n\n\n t_exp1 = ", t_exp1, "\n")
object.size(exp1)/(Nindiv * 1024^2)
cat("\n\n")
summary(unlist(lapply(exp1, "[[", "NumClones")))
summary(unlist(lapply(exp1, "[[", "NumIter")))
summary(unlist(lapply(exp1, "[[", "FinalTime")))
summary(unlist(lapply(exp1, "[[", "TotalPopSize")))
```

The above runs yield the following:

Table 2: Benchmarks of Exp and McFL models using the default `detectionProb` with two settings of `keepEvery`.

	Elapsed Time, average per simulation (s)	Object Size, average per simulation (MB)	Number of Clones, median	Number of Iterations, median	Final Time, median	Total Population Size, median	Total Population Size, max.	keepEvery
**exp1**	0	0.04	2	254	252	1,058	11,046	1
**mc1**	0.74	3.9	12	816,331	20,406	696	979	1
**exp2**	0	0.01	1	296	294	1,021	21,884	NA
**mc2**	0.7	0.01	1	694,716	17,366	692	888	NA

The above table shows that a naive comparison (looking simply at execution time) might conclude that the McFL model is much, much slower than the Exp model. But that is not the complete story: using the `detectionProb` stopping mechanism (see 6.3.2) will lead to stopping the simulations very quickly in the exponential model because as soon as a clone with fitness > 1 appears it starts growing exponentially. In fact, we can see that the number of iterations and the final time are much smaller in the Exp than in the McFL model. We will elaborate on this point below (section 2.2.1), when we discuss the setting for `checkSizePEvery` (here left at its default value of 20): checking the exiting condition more often (smaller `checkSizePEvery`) would probably be justified here (notice also the very large final times) and would lead to a sharp decrease in number of iterations and, thus, running time.

This table also shows that the `keepEvery = NA` setting, which was in effect in simulations `exp2` and `mc2`, can make a difference especially for the McFL models, as seen by the median number of clones and the size of the returned object. Models `exp2` and `mc2` do not store any intermediate population samples so clones that become extinct at any sampling period are pruned and only the existing clones at the end of the simulation are returned. In contrast, models `exp1` and `mc1` store population samples at time intervals of 1 (`keepEvery = 1`), even if many of those clones eventually become extinct. We will return to this issue below as execution time and object size depend strongly on the number of clones tracked.

We can run the exponential model again modifying the arguments of the `detectionProb` mechanism; in two of the models below (`exp3` and `exp4`) no detection can take place unless populations are at least 100 times larger than the initial population size, and probability of detection is 0.1 with a population size 1,000 times larger than the initial one (`PDBaseline = 5e4`, `n2 = 5e5`). In the other two models (`exp5` and `exp6`), no detection can take place unless populations are at least 1,000 times larger than the initial population size, and probability of detection is 0.1 with a population size 100,000 times larger than the initial one (`PDBaseline = 5e5`, `n2 = 5e7`)[2]. In runs `exp3` and `exp5` we set `keepEvery = 1` and in runs `exp4` and `exp6` we set `keepEvery = NA`.

```
t_exp3 <- system.time(
    exp3 <- oncoSimulPop(Nindiv, pancr,
                         detectionProb = c(PDBaseline = 5e4,
                                           p2 = 0.1, n2 = 5e5,
                                           checkSizePEvery = 20),
                         detectionSize = NA,
                         detectionDrivers = NA,
                         finalTime = NA,
                         keepEvery = 1,
                         model = "Exp",
                         mc.cores = 1))["elapsed"]/Nindiv
```

[2]Again, these are not necessarily reasonable or common settings. We are using them to understand what and how affects running time and space consumption.

```
t_exp4 <- system.time(
    exp4 <- oncoSimulPop(Nindiv, pancr,
                            detectionProb = c(PDBaseline = 5e4,
                                              p2 = 0.1, n2 = 5e5,
                                              checkSizePEvery = 20),
                            detectionSize = NA,
                            detectionDrivers = NA,
                            finalTime = NA,
                            keepEvery = NA,
                            model = "Exp",
                            mc.cores = 1))["elapsed"]/Nindiv



t_exp5 <- system.time(
    exp5 <- oncoSimulPop(Nindiv, pancr,
                            detectionProb = c(PDBaseline = 5e5,
                                              p2 = 0.1, n2 = 5e7),
                            detectionSize = NA,
                            detectionDrivers = NA,
                            finalTime = NA,
                            keepEvery = 1,
                            model = "Exp",
                            mc.cores = 1))["elapsed"]/Nindiv

t_exp6 <- system.time(
    exp6 <- oncoSimulPop(Nindiv, pancr,
                            detectionProb = c(PDBaseline = 5e5,
                                              p2 = 0.1, n2 = 5e7),
                            detectionSize = NA,
                            detectionDrivers = NA,
                            finalTime = NA,
                            keepEvery = NA,
                            model = "Exp",
                            mc.cores = 1))["elapsed"]/Nindiv
```

Table 3: Benchmarks of Exp and McFL models modifying the default `detectionProb` with two settings of `keepEvery`.

	Elapsed Time, average per simulation (s)	Object Size, average per simulation (MB)	Number of Clones, median	Number of Iterations, median	Final Time, median	Total Population Size, median	Total Population Size, max.	keepEvery	PDBaseline	n2
**exp3**	0.01	0.41	14	2,754	1,890	6,798,358	2.7e+08	1	50,000	5e+05
**exp4**	0.01	0.02	8	2,730	2,090	7,443,812	1.7e+08	NA	50,000	5e+05
**exp5**	0.84	0.91	34	54,332	2,026	1.4e+09	4.2e+10	1	5e+05	5e+07
**exp6**	0.54	0.02	27	44,288	2,026	1.2e+09	3.3e+10	NA	5e+05	5e+07

As above, `keepEvery = NA` (in `exp4` and `exp6`) leads to much smaller object sizes and slightly smaller numbers of clones and execution times. Changing the exiting conditions (by changing `detectionProb` arguments) leads to large increases in number of iterations (in this case by factors of about 15x to 25x) and a corresponding increase in execution time as well as much larger population sizes (in some cases $> 10^{10}$).

In some of the runs of `exp5` and `exp6` we get the (recoverable) exception message from the C++ code: `Recoverable exception ti set to DBL_MIN. Rerunning`, which is related to those simulations reaching total population sizes $> 10^{10}$; we return to this below (section 2.4). You might also wonder why total and median population sizes are so large in these two runs, given the exiting conditions. One of the reasons is that we are using the default `checkSizePEvery = 20`, so the interval between successive checks of the exiting condition is large; this is discussed at greater length in section 2.2.1.

All the runs above used the default value `onlyCancer = TRUE`. This means that simulations will be repeated until the exiting conditions are reached (see details in section 6.3) and, therefore, any simulation that ends up in extinction will be repeated. This setting can thus have a large effect on the exponential models, because when the initial population size is not very large and we start from the wildtype, it is not uncommon for simulations to become extinct (when birth and death rates are equal and the population size is small, it is easy to reach extinction before a mutation in a gene that increases fitness occurs). But this is rarely the case in the McFarland model (unless we use really tiny initial population sizes) because of the dependency of death rate on total population size (see section 3.2.1).

The number of attempts until cancer was reached in the above models is shown in Table 4 (the values can be obtained from any of the above runs doing, for instance, `median(unlist(lapply(exp1, function(x) x$other$attemptsUsed)))`):

Table 4: Number of attempts until cancer.

	Attempts until Cancer, median	Attempts until Cancer, mean	Attempts until Cancer, max.	PDBaseline	n2
**exp1**	1	1.9	7	600	1,000
**mc1**	1	1	1	600	1,000
**exp2**	2	2.2	16	600	1,000
**mc2**	1	1	1	600	1,000
**exp3**	6	7.7	40	50,000	5e+05
**exp4**	6	8	39	50,000	5e+05
**exp5**	5	8.3	41	5e+05	5e+07
**exp6**	5	7.2	30	5e+05	5e+07

The McFL models finish in a single attempt. The exponential model simulations where we can exit with small population sizes (`exp1`, `exp2`) need many fewer attempts to reach cancer than those where large population sizes are required (`exp3` to `exp6`).

There is no relevant different among those last four, which is what we would expect: a population that has already reached a size of 50,000 cells from an initial population size of 500 is obviously a growing population where there is at least one mutant with positive fitness; thus, it unlikely to go extinct and therefore having to grow up to at least 500,000 will not significantly increase the risk of extinction.

We will now rerun all of the above models with argument `onlyCancer = FALSE`. The results are shown in Table 5 (note that the differences between this table and Table 2 for the McFL models are due only to sampling variation).

Table 5: Benchmarks of models in Table 2 and 3 when run with `onlyCancer = FALSE`.

	Elapsed Time, average per simulation (s)	Object Size, average per simulation (MB)	Number of Clones, median	Number of Iterations, median	Final Time, median	Total Population Size, median	Total Population Size, mean	Total Population Size, max.	keepEvery	PDBaseline	n2
**exp1__noc**	0.001	0.041	1.5	394	393	0	708	18,188	1	600	1,000
**mc1__noc**	0.69	3.9	12	673,910	16,846	692	700	983	1	600	1,000
**exp2__noc**	0.001	0.012	1	320	319	726	870	26,023	NA	600	1,000
**mc2__noc**	0.65	0.014	1	628,683	15,716	694	704	910	NA	600	1,000
**exp3__noc**	0.002	0.15	2	718	694	0	2,229,519	5.7e+07	1	50,000	5e+05
**exp4__noc**	0.002	0.013	0	600	599	0	3,122,765	1.3e+08	NA	50,000	5e+05
**exp5__noc**	0.17	0.22	3	848	777	0	5.9e+08	1.5e+10	1	5e+05	5e+07
**exp6__noc**	0.068	0.013	0	784	716	0	4.1e+08	1.3e+10	NA	5e+05	5e+07

Now most simulations under the exponential model end up in extinction, as seen by the median population size of 0 (but not all, as the mean and max. population size are clearly away from zero). Consequently, simulations under the exponential model are now faster (and the size of the average returned object is smaller). Of course, whether one should run simulations with `onlyCancer = TRUE` or `onlyCancer = FALSE` will depend on the question being asked (see, for example, section 1.3.5 for a question where we will naturally want to use `onlyCancer = FALSE`).

To make it easier to compare results with those of the next section, Table 6 shows all the runs so far.

Table 6: Benchmarks of all models in Tables 2, 3, and 5.

	Elapsed Time, average per simulation (s)	Object Size, average per simulation (MB)	Number of Clones, median	Number of Iterations, median	Final Time, median	Total Population Size, median	Total Population Size, mean	Total Population Size, max.	keepEvery	PDBaseline	n2	onlyCancer
**exp1**	0.001	0.037	2	254	252	1,058	1,277	11,046	1	600	1,000	TRUE
**mc1**	0.74	3.9	12	816,331	20,406	696	702	979	1	600	1,000	TRUE
**exp2**	0.001	0.012	1	296	294	1,021	1,392	21,884	NA	600	1,000	TRUE
**mc2**	0.7	0.014	1	694,716	17,366	692	698	888	NA	600	1,000	TRUE
**exp3**	0.01	0.41	14	2,754	1,890	6,798,358	1.7e+07	2.7e+08	1	50,000	5e+05	TRUE
**exp4**	0.009	0.016	8	2,730	2,090	7,443,812	1.5e+07	1.7e+08	NA	50,000	5e+05	TRUE
**exp5**	0.84	0.91	34	54,332	2,026	1.4e+09	3.5e+09	4.2e+10	1	5e+05	5e+07	TRUE
**exp6**	0.54	0.021	27	44,288	2,026	1.2e+09	3.2e+09	3.3e+10	NA	5e+05	5e+07	TRUE
**exp1_noc**	0.001	0.041	1.5	394	393	0	708	18,188	1	600	1,000	FALSE
**mc1_noc**	0.69	3.9	12	673,910	16,846	692	700	983	1	600	1,000	FALSE
**exp2_noc**	0.001	0.012	1	320	319	726	870	26,023	NA	600	1,000	FALSE
**mc2_noc**	0.65	0.014	1	628,683	15,716	694	704	910	NA	600	1,000	FALSE
**exp3_noc**	0.002	0.15	2	718	694	0	2,229,519	5.7e+07	1	50,000	5e+05	FALSE
**exp4_noc**	0.002	0.013	0	600	599	0	3,122,765	1.3e+08	NA	50,000	5e+05	FALSE
**exp5_noc**	0.17	0.22	3	848	777	0	5.9e+08	1.5e+10	1	5e+05	5e+07	FALSE
**exp6_noc**	0.068	0.013	0	784	716	0	4.1e+08	1.3e+10	NA	5e+05	5e+07	FALSE

2.1.1 Changing fitness: $s = 0.1$ and $s = 0.05$

In the above fitness specification the fitness effect of each gene (when its restrictions are satisfied) is $s = 0.1$ (see section 3.2 for details). Here we rerun all the above benchmarks using $s = 0.05$ (the results from these benchmarks are available as `data(benchmark_1_0.05)`) and results are shown below in Table 7.

Table 7: Benchmarks of all models in Table 6 using $s = 0.05$ (instead of $s = 0.1$).

	Elapsed Time, average per simulation (s)	Object Size, average per simulation (MB)	Number of Clones, median	Number of Iterations, median	Final Time, median	Total Population Size, median	Total Population Size, mean	Total Population Size, max.	keepEvery	PDBaseline	n2	onlyCancer
**exp1**	0.002	0.043	2	316	315	1,104	1,181	3,176	1	600	1,000	TRUE
**mc1**	1.7	11	17	2e+06	50,696	644	647	761	1	600	1,000	TRUE
**exp2**	0.001	0.012	1	274	273	1,129	1,281	7,608	NA	600	1,000	TRUE
**mc2**	1.6	0.016	1	1,615,197	40,376	644	651	772	NA	600	1,000	TRUE
**exp3**	0.012	0.63	15	3,995	2,919	3,798,540	5,892,376	4.5e+07	1	50,000	5e+05	TRUE
**exp4**	0.011	0.017	9	4,288	3,276	4,528,072	6,551,319	3.2e+07	NA	50,000	5e+05	TRUE
**exp5**	0.3	1.2	34	68,410	2,751	6.8e+08	1e+09	8.2e+09	1	5e+05	5e+07	TRUE
**exp6**	0.26	0.022	23	44,876	2,499	4.3e+08	8.9e+08	7.3e+09	NA	5e+05	5e+07	TRUE
**exp1_noc**	0.001	0.039	2	310	308	0	522	2,239	1	600	1,000	FALSE
**mc1_noc**	1.6	11	17	2e+06	50,776	638	643	757	1	600	1,000	FALSE
**exp2_noc**	0.001	0.012	0	340	336	0	599	3,994	NA	600	1,000	FALSE
**mc2_noc**	1.7	0.017	1	2,102,439	52,556	645	650	740	NA	600	1,000	FALSE
**exp3_noc**	0.002	0.11	2	618	615	0	150,978	6,093,498	1	50,000	5e+05	FALSE
**exp4_noc**	0.002	0.013	0	813	812	0	558,225	2.3e+07	NA	50,000	5e+05	FALSE
**exp5_noc**	0.031	0.23	3	917	914	0	1.1e+08	3.7e+09	1	5e+05	5e+07	FALSE
**exp6_noc**	0.046	0.013	0	628	610	0	1.7e+08	5.1e+09	NA	5e+05	5e+07	FALSE

As expected, having a smaller s leads to slower processes in most cases, since it takes longer to reach the exiting conditions sooner. Particularly noticeable are the runs for the McFL models (notice the increases in population size and number of iterations —see also below).

That is not the case, however, for `exp5` and `exp6` (and `exp5_noc` and `exp6_noc`). When running with $s = 0.05$ the simulations exit at a later time (see column "Final Time") but they exit with smaller population sizes. Here we have an interaction between sampling frequency, speed of growth of the population, mutation events and number of clones. In populations that grow much faster mutation events will happen more often (which will trigger further iterations of the algorithm); in addition, more new clones will be created, even if they only exist for short times and become extinct by the following sampling period (so they are not reflected in the `pops.by.time` matrix). These differences are proportionally larger the larger the rate of growth of the population. Thus, they are larger between, say, the `exp5` at $s = 0.1$ and $s = 0.05$ than between the `exp4` at the two different s: the `exp5` exit conditions can only be satisfied at much larger population sizes so at populations sizes when growth is much faster (recall we are dealing with exponential growth).

Recall also that with the default settings in `detectionProb`, we assess the exiting condition every 20 time periods (argument `checkSizePEvery`); this means that for fast growing populations, the increase in population size between successive checks of the exit conditions will be much larger (this phenomenon is also discussed in section 2.2.1).

Thus, what is happening in the `exp5` and `exp6` with $s = 0.1$ is that close to the time the exit conditions could be satisfied, they are growing very fast, accumulating mutants, and incurring in additional iterations. They exit sooner in terms of time periods, but they do much more work before arriving there.

The setting of `checkSizePEvery` is also having a huge effect on the McFL model simulations (the number of iterations is $> 10^6$). Even more than in the previous section, checking the exiting condition more often (smaller `checkSizePEvery`) would probably be justified here (notice also the very large final times) and would lead to a sharp decrease in number of iterations and, thus, running time.

The moral here is that in complex simulations like this (and most simulations are complex), the effects of some parameters (s in this case) might look counter-intuitive at first. Thus the need to "experiment before launching a large number of simulations".

2.2 Several "common use cases" runs

Let us now execute some simulations under more usual conditions. We will use seven different fitness specifications: the pancreas example, two random fitness landscapes, and four sets of independent genes (200 to 4000 genes) with fitness effects randomly drawn from exponential distributions:

```r
pancr <- allFitnessEffects(
    data.frame(parent = c("Root", rep("KRAS", 4),
                   "SMAD4", "CDNK2A",
                   "TP53", "TP53", "MLL3"),
               child = c("KRAS","SMAD4", "CDNK2A",
                   "TP53", "MLL3",
                   rep("PXDN", 3), rep("TGFBR2", 2)),
               s = 0.1,
               sh = -0.9,
               typeDep = "MN"),
    drvNames = c("KRAS", "SMAD4", "CDNK2A", "TP53",
                 "MLL3", "TGFBR2", "PXDN"))


## Random fitness landscape with 6 genes
## At least 50 accessible genotypes
rfl6 <- rfitness(6, min_accessible_genotypes = 50)
attributes(rfl6)$accessible_genotypes ## How many accessible
rf6 <- allFitnessEffects(genotFitness = rfl6)


## Random fitness landscape with 12 genes
## At least 200 accessible genotypes
rfl12 <- rfitness(12, min_accessible_genotypes = 200)
attributes(rfl12)$accessible_genotypes ## How many accessible
rf12 <- allFitnessEffects(genotFitness = rfl12)




## Independent genes; positive fitness from exponential distribution
## with mean around 0.1, and negative from exponential with mean
## around -0.02. Half of genes positive fitness effects, half
## negative.

ng <- 200 re_200 <- allFitnessEffects(noIntGenes = c(rexp(ng/2, 10),
-rexp(ng/2, 50)))

ng <- 500
re_500 <- allFitnessEffects(noIntGenes = c(rexp(ng/2, 10),
                                           -rexp(ng/2, 50)))

ng <- 2000
re_2000 <- allFitnessEffects(noIntGenes = c(rexp(ng/2, 10),
                                            -rexp(ng/2, 50)))
```

```
ng <- 4000
re_4000 <- allFitnessEffects(noIntGenes = c(rexp(ng/2, 10),
                                            -rexp(ng/2, 50)))
```

2.2.1 Common use cases, set 1.

We will use the Exp and the McFL models, run with different parameters. The script is provided as 'benchmark_2.R', under '/inst/miscell', with output in the 'miscell-files/vignette_bench_Rout' directory of the main OncoSimul repository at https://github.com/rdiaz02/OncoSimul. The data are available as `data(benchmark_2)`.

For the Exp model the call will be

```
oncoSimulPop(Nindiv,
             fitness,
             detectionProb = NA,
             detectionSize = 1e6,
             initSize = 500,
             detectionDrivers = NA,
             keepPhylog = TRUE,
             model = "Exp",
             errorHitWallTime = FALSE,
             errorHitMaxTries = FALSE,
             finalTime = 5000,
             onlyCancer = FALSE,
             mc.cores = 1,
             sampleEvery = 0.5,
             keepEvery = 1)
```

And for McFL:

```
initSize <- 1000
oncoSimulPop(Nindiv,
               fitness,
                detectionProb = c(
                    PDBaseline = 1.4 * initSize,
                    n2 = 2 * initSize,
                    p2 = 0.1,
                    checkSizePEvery = 4),
                initSize = initSize,
                detectionSize = NA,
                detectionDrivers = NA,
                keepPhylog = TRUE,
                model = "McFL",
                errorHitWallTime = FALSE,
                errorHitMaxTries = FALSE,
```

```
                 finalTime = 5000,
                 max.wall.time = 10,
                 onlyCancer = FALSE,
                 mc.cores = 1,
                 keepEvery = 1)
```

For the exponential model we will stop simulations when populations have $> 10^6$ cells (simulations start from 500 cells). For the McFarland model we will use the `detectionProb` mechanism (see section 6.3.2 for details); we could have used as stopping mechanism `detectionSize = 2 * initSize` (which would be basically equivalent to reaching cancer, as argued in (McFarland et al. 2013)) but we want to provide further examples under the `detectionProb` mechanism. We will start from 1000 cells, not 500 (starting from 1000 we almost always reach cancer in a single run).

Why not use the `detectionProb` mechanism with the `Exp` models? Because it can be hard to intuitively understand what are reasonable settings for the parameters of the `detectionProb` mechanism when used in a population that is growing exponentially, especially if different genes have very different effects on fitness. Moreover, we are using fitness specifications that are very different (compare the fitness landscape of six genes, the pancreas specification, and the fitness specification with 4000 genes with fitness effects drawn from an exponential distribution —`re_4000`). In contrast, the `detectionProb` mechanism might be simpler to reason about in a population that is growing under a model of carrying capacity with possibly large periods of stasis. Let us emphasize that it is not that the `detectionProb` mechanism does not make sense with the Exp model; it is simply that the parameters might need finer adjustment for them to make sense, and in these benchmarks we are dealing with widely different fitness specifications.

Note also that we specify `checkSizePEvery = 4` (instead of the default, which is 20). Why? Because the fitness specifications where fitness effects are drawn from exponential distributions (`re_200` to `re_4000` above) include many genes (well, up to 4000) some of them with possibly very large effects. In these conditions, simulations can run very fast in the sense of "units of time". If we check exiting conditions every 20 units the population could have increased its size several orders of magnitude in between checks (this is also discussed in sections 2.1.1 and 6.3.2). You can verify this by running the script with other settings for `checkSizePEvery` (and being aware that large settings might require you to wait for a long time). To ensure that populations have really grown, we have increased the setting of `PDBaseline` so that no simulation can be considered for stopping unless its size is 1.4 times larger than `initSize`.

In all cases we use `keepEvery = 1` and `keepPhylog = TRUE` (so we store the population sizes of all clones every 1 time unit and we keep the complete genealogy of clones). Finally, we run all models with `errorHitWallTime = FALSE` and `errorHitMaxTries = FALSE` so that we can see results even if stopping conditions are not met.

The results of the benchmarks, using 100 individual simulations, are shown in Table 8.

Table 8: Benchmarks under some common use cases, set 1.

Model	Fitness	Elapsed Time, average per simulation (s)	Object Size, average per simulation (MB)	Number of Clones, median	Number of Iterations, median	Final Time, median	Total Population Size, median	Total Population Size, mean	Total Population Size, max.
Exp	pancr	0.002	0.12	3	1,397	697	0	164,222	1,053,299
McFL	pancr	0.12	0.56	8	2e+05	5,000	1,037	1,144	1,938
Exp	rf6	0.002	0.064	6	783	391	1e+06	594,899	1,309,497
McFL	rf6	0.019	0.071	3	23,297	582	1,884	1,975	4,636
Exp	rf12	0.01	0.13	4	1,178	542	0	287,669	1,059,141
McFL	rf12	0.14	0.82	18	2e+05	5,000	1,252	1,295	1,695
Exp	re_200	0.013	0.67	230	1,185	223	1,060,944	859,606	1,536,242
McFL	re_200	0.018	0.22	47	9,679	240	2,166	2,973	29,301
Exp	re_500	0.09	2.7	771	2,732	152	1,068,732	959,026	1,285,522
McFL	re_500	0.024	0.44	91	7,056	172	2,148	2,578	8,234
Exp	re_2000	0.91	29	3,376	7,412	70	1,163,990	1,143,041	1,741,492
McFL	re_2000	0.031	1.9	186	3,546	80	2,870	3,704	13,248
Exp	re_4000	3.3	113	7,088	12,216	52	1,217,568	1,309,185	2,713,200
McFL	re_4000	0.063	6.5	326	2,731	52	4,592	13,601	729,611

In most cases, simulations run reasonably fast (under 0.1 seconds per individual simulation) and the returned objects are small. I will only focus on a few cases.

The McFL model with random fitness landscape `rf12` and with `pancr` does not satisfy the conditions of `detectionProb` in most cases: its median final time is 5000, which was the maximum final time specified. This suggests that the fitness landscape is such that it is unlikely that we will reach population sizes > 1400 (remember we the setting for `PDBaseline`) before 5000 time units. There is nothing particular about using a fitness landscape of 12 genes and other runs in other 12-gene random fitness landscapes do not show this pattern. However, complex fitness landscapes might be such that genotypes of high fitness (those that allow reaching a large population size quickly) are not easily accessible[3] so reaching them might take a long time. This does not affect the exponential model in the same way because, well, because there is exponential growth in that model: any genotype with fitness > 1 will grow exponentially (of course, at possibly very different rates). You might want to play with the script and modify the call to `rfitness` (using different values of `reference` and `c`, for instance) to have simpler paths to a maximum or modify the call to `oncoSimulPop` (with, say, `finalTime` to much larger values). Some of these issues are related to more general questions about fitness landscapes and accessibility (see section 1.3.2 and references therein).

You could also set `onlyCancer = TRUE`. This might make sense if you are interested in only seeing simulations that "reach cancer" (where "reach cancer" means reaching a state you define as a function of population size or drivers). However, if you are exploring fitness landscapes, `onlyCancer = TRUE` might not always be reasonable as reaching a particular population size, for instance, might just not be possible under some fitness landscapes (this phenomenon is of course not restricted to random fitness landscapes —see also section 2.3.3).

As we anticipated above, the `detectionProb` mechanism has to be used with care: some of the simulations run in very short "time units", such as those for the fitness specifications with 2000 and 4000 genes. Having used a `checkSizePEvery = 20` probably would not have made sense.

Finally, it is interesting that in the cases examined here, the two slowest running simulations are from "Exp", with fitnesses `re_2000` and `re_4000` (and the third slowest is also Exp, under `re_500`). These are also the cases with the largest number of clones. Why? In the "Exp" model there is no competition, and fitness specifications `re_2000` and `re_4000` have genomes with many genes with positive fitness contributions. It is thus very easy to obtain, from the wildtype ancestor, a large number of clones all of which have birth rates > 1 and, thus, clones that are unlikely to become extinct.

[3]By easily accessible I mean that there are many, preferably short, paths of non-decreasing fitness from the wildtype to this genotype. See definitions and discussion in, e.g., Franke et al. (2011).

2.2.2 Common use cases, set 2.

We will now rerun the simulations above changing the following:

- `finalTime` set to 25000.
- `onlyCancer` set to TRUE.
- The "Exp" models will stop when population size $> 10^5$.

This is in script 'benchmark_3.R', under '/inst/miscell', with output in the 'miscell-files/vignette_bench_Rout' directory of the main OncoSimul repository at https://github.com/rdiaz02/OncoSimul. The data are available as `data(benchmark_3)`.

Table 9: Benchmarks under some common use cases, set 2.

Model	Fitness	Elapsed Time, average per simulation (s)	Object Size, average per simulation (MB)	Number of Clones, median	Number of Iterations, median	Final Time, median	Total Population Size, median	Total Population Size, mean	Total Population Size, max.
Exp	pancr	0.012	0.32	10	3,480	1,718	1e+05	1e+05	108,805
McFL	pancr	0.41	1.7	14	4e+05	9,955	1,561	1,555	1,772
Exp	rf6	0.003	0.058	4	866	430	107,492	109,774	135,257
McFL	rf6	0.033	0.12	4	35,216	880	2,003	2,010	3,299
Exp	rf12	0.012	0.098	9	1,138	561	1e+05	1e+05	112,038
McFL	rf12	0.17	0.76	16	1e+05	2,511	1,486	1,512	1,732
Exp	re_200	0.004	0.39	106	723	252	1e+05	105,586	122,338
McFL	re_200	0.026	0.33	61	13,484	335	1,830	2,049	3,702
Exp	re_500	0.007	0.61	168	490	117	110,311	112,675	134,860
McFL	re_500	0.018	0.33	70	5,157	126	2,524	3,455	19,899
Exp	re_2000	0.046	5.7	651	1,078	68	106,340	109,081	153,146
McFL	re_2000	0.029	1.8	186	3,444	80	2,837	4,009	37,863
Exp	re_4000	0.1	19	1,140	1,722	51	111,256	113,499	168,958
McFL	re_4000	0.057	6.7	325	3,081	60	3,955	8,892	265,183

Since we increased the maximum final time and forced runs to "reach cancer" the McFL run with the pancreas fitness specification takes a bit longer because it also has to do a larger number of iterations. Interestingly, notice that the median final time is close to 10000, so the runs in 2.2.1 with maximum final time of 5000 would have had a hard time finishing with `onlyCancer = TRUE`.

Forcing simulations to "reach cancer" and just random differences between the random fitness landscape also affect the McFL run under `rf12`: final time is below 5000 and the median number of iterations is about half of what was above.

Finally, by stopping the Exp simulations at 10^5, simulations with `re_2000` and `re_4000` finish now in much shorter times (but they still take longer than their McFL counterparts) and the number of clones created is much smaller.

2.3 Can we use a large number of genes?

Yes. In fact, in OncoSimulR there is no pre-set limit on genome size. However, large numbers of genes can lead to unacceptably large returned object sizes and/or running time. We discuss several examples next that illustrate some of the major issues to consider. Another example with 50,000 genes is shown in section 6.5.3.

We have seen in 2.1 and 2.2.1 that for the Exp model, benchmark results using `detectionProb` require a lot of care and can be misleading. Here, we will fix initial population sizes (to 500) and all final population sizes will be set to $\geq 10^6$. In addition, to avoid the confounding factor of the `onlyCancer = TRUE` argument, we will set it to FALSE, so we measure directly the time of individual runs.

2.3.1 Exponential model with 10,000 and 50,000 genes

2.3.1.1 Exponential, 10,000 genes, example 1

We will start with 10000 genes and an exponential model, where we stop when the population grows over 10^6 individuals:

```
ng <- 10000
u <- allFitnessEffects(noIntGenes = c(rep(0.1, ng/2),
                                       rep(-0.1, ng/2)))

t_e_10000 <- system.time(
    e_10000 <- oncoSimulPop(5, u, model = "Exp", mu = 1e-7,
                            detectionSize = 1e6,
                            detectionDrivers = NA,
                            detectionProb = NA,
                            keepPhylog = TRUE,
                            onlyCancer = FALSE,
                            mutationPropGrowth = TRUE,
                            mc.cores = 1))
```

```r
t_e_10000
##    user  system elapsed
##   4.368   0.196   4.566

summary(e_10000)[, c(1:3, 8, 9)]
##   NumClones TotalPopSize LargestClone FinalTime NumIter
## 1      5017      1180528       415116       143    7547
## 2      3726      1052061       603612       131    5746
## 3      4532      1100721       259510       132    6674
## 4      4150      1283115       829728        99    6646
## 5      4430      1139185       545958       146    6748

print(object.size(e_10000), units = "MB")
## 863.9 Mb
```

Each simulation takes about 1 second but note that the number of clones for most simulations is already over 4000 and that the size of the returned object is close to 1 GB (a more detailed explanation of where this 1 GB comes from is deferred until section 2.3.1.6).

2.3.1.2 Exponential, 10,000 genes, example 2

We can decrease the size of the returned object if we use the `keepEvery = NA` argument (this setting was explained in detail in section 2.1):

```r
t_e_10000b <- system.time(
    e_10000b <- oncoSimulPop(5,
                             u,
                             model = "Exp",
                             mu = 1e-7,
                             detectionSize = 1e6,
                             detectionDrivers = NA,
                             detectionProb = NA,
                             keepPhylog = TRUE,
                             onlyCancer = FALSE,
                             keepEvery = NA,
                             mutationPropGrowth = TRUE,
                             mc.cores = 1
                             ))
```

```r
t_e_10000b
##    user  system elapsed
##   5.484   0.100   5.585

summary(e_10000b)[, c(1:3, 8, 9)]
##   NumClones TotalPopSize LargestClone FinalTime NumIter
## 1      2465      1305094       727989        91    6447
```

```
## 2      2362      1070225       400329       204     8345
## 3      2530      1121164       436721       135     8697
## 4      2593      1206293       664494       125     8149
## 5      2655      1186994       327835       191     8572

print(object.size(e_10000b), units = "MB")
## 488.3 Mb
```

2.3.1.3 Exponential, 50,000 genes, example 1

Let's use 50,000 genes. To keep object sizes reasonable we use `keepEvery = NA`. For now, we also set `mutationPropGrowth = FALSE` so that the mutation rate does not become really large in clones with many mutations but, of course, whether or not this is a reasonable decision depends on the problem; see also below.

```
ng <- 50000
u <- allFitnessEffects(noIntGenes = c(rep(0.1, ng/2),
                                      rep(-0.1, ng/2)))
t_e_50000 <- system.time(
    e_50000 <- oncoSimulPop(5,
                            u,
                            model = "Exp",
                            mu = 1e-7,
                            detectionSize = 1e6,
                            detectionDrivers = NA,
                            detectionProb = NA,
                            keepPhylog = TRUE,
                            onlyCancer = FALSE,
                            keepEvery = NA,
                            mutationPropGrowth = FALSE,
                            mc.cores = 1
                            ))


t_e_50000
##    user  system elapsed
##  44.192   1.684  45.891

summary(e_50000)[, c(1:3, 8, 9)]
##   NumClones TotalPopSize LargestClone FinalTime NumIter
## 1      7367      1009949       335455     75.00   18214
## 2      8123      1302324       488469     63.65   17379
## 3      8408      1127261       270690     72.57   21144
## 4      8274      1138513       318152     80.59   20994
## 5      7520      1073131       690814     70.00   18569
```

```r
print(object.size(e_50000), units = "MB")
## 7598.6 Mb
```

Of course, simulations now take longer and the size of the returned object is over 7 GB (we are keeping more than 7,000 clones, even if when we prune all those that went extinct).

2.3.1.4 Exponential, 50,000 genes, example 2

What if we had not pruned?

```r
ng <- 50000
u <- allFitnessEffects(noIntGenes = c(rep(0.1, ng/2),
                                      rep(-0.1, ng/2)))
t_e_50000np <- system.time(
    e_50000np <- oncoSimulPop(5,
                              u,
                              model = "Exp",
                              mu = 1e-7,
                              detectionSize = 1e6,
                              detectionDrivers = NA,
                              detectionProb = NA,
                              keepPhylog = TRUE,
                              onlyCancer = FALSE,
                              keepEvery = 1,
                              mutationPropGrowth = FALSE,
                              mc.cores = 1
                              ))

t_e_50000np
##   user  system elapsed
## 42.316   2.764  45.079

summary(e_50000np)[, c(1:3, 8, 9)]
##   NumClones TotalPopSize LargestClone FinalTime NumIter
## 1     13406      1027949       410074     71.97   19469
## 2     12469      1071325       291852     66.00   17834
## 3     11821      1089834       245720     90.00   16711
## 4     14008      1165168       505607     77.61   19675
## 5     14759      1074621       205954     87.68   20597

print(object.size(e_50000np), units = "MB")
## 12748.4 Mb
```

The main effect is not on execution time but on object size (it has grown by 5 GB). We are tracking more than 10,000 clones.

2.3.1.5 Exponential, 50,000 genes, example 3

What about the `mutationPropGrowth` setting? We will rerun the example in 2.3.1.3 leaving `keepEvery = NA` but with the default `mutationPropGrowth`:

```
ng <- 50000
u <- allFitnessEffects(noIntGenes = c(rep(0.1, ng/2),
                                       rep(-0.1, ng/2)))

t_e_50000c <- system.time(
    e_50000c <- oncoSimulPop(5,
                             u,
                             model = "Exp",
                             mu = 1e-7,
                             detectionSize = 1e6,
                             detectionDrivers = NA,
                             detectionProb = NA,
                             keepPhylog = TRUE,
                             onlyCancer = FALSE,
                             keepEvery = NA,
                             mutationPropGrowth = TRUE,
                             mc.cores = 1
                             ))

t_e_50000c
##    user  system elapsed
## 84.228   2.416  86.665

summary(e_50000c)[, c(1:3, 8, 9)]
##   NumClones TotalPopSize LargestClone FinalTime NumIter
## 1     11178      1241970       344479     84.74   27137
## 2     12820      1307086       203544     91.94   33448
## 3     10592      1126091       161057     83.81   26064
## 4     11883      1351114       148986     65.68   25396
## 5     10518      1101392       253523     99.79   26082

print(object.size(e_50000c), units = "MB")
## 10904.9 Mb
```

As expected (because the mutation rate per unit time is increasing in the fastest growing clones), we have many more clones, larger objects, and longer times of execution here: we almost double the time and the size of the object increases by almost 3 GB.

What about larger population sizes or larger mutation rates? The number of clones starts growing fast, which means much slower execution times and much larger returned objects (see also the examples below).

2.3.1.6 Interlude: where is that 1 GB coming from?

In section 2.3.1.1 we have seen an apparently innocuous simulation producing a returned object of almost 1 GB. Where is that coming from? It means that each simulation produced almost 200 MB of output.

Let us look at one simulation in more detail:

```
r1 <- oncoSimulIndiv(u,
                     model = "Exp",
                     mu = 1e-7,
                     detectionSize = 1e6,
                     detectionDrivers = NA,
                     detectionProb = NA,
                     keepPhylog = TRUE,
                     onlyCancer = FALSE,
                     mutationPropGrowth = TRUE
                     )
summary(r1)[c(1, 8)]
##   NumClones  FinalTime
## 1      3887        345

print(object.size(r1), units = "MB")
## 160 Mb

## Size of the two largest objects inside:
sizes <- lapply(r1, function(x) object.size(x)/(1024^2))
sort(unlist(sizes), decreasing = TRUE)[1:2]
## Genotypes pops.by.time
##       148.28       10.26

dim(r1$Genotypes)
## [1] 10000  3887
```

The above shows the reason: the `Genotypes` matrix is a 10,000 by 3,887 integer matrix (with a 0 and 1 indicating not-mutated/mutated for each gene in each genotype) and in R integers use 4 bytes each. The `pops.by.time` matrix is 346 by 3,888 (the 1 in $346 = 345 + 1$ comes from starting at 0 and going up to the final time, both included; the 1 in $3888 = 3887 + 1$ is from the column of time) double matrix and doubles use 8 bytes[4].

2.3.2 McFarland model with 50,000 genes; the effect of `keepEvery`

We show an example of McFarland's model with 50,000 genes in section 6.5.3. We will show here a few more examples with those many genes but with a different

[4] These matrices do not exist during most of the execution of the C++ code; they are generated right before returning from the C++ code.

fitness specification and changing several other settings.

2.3.2.1 McFarland, 50,000 genes, example 1

Let's start with `mutationPropGrowth = FALSE` and `keepEvery = NA`. Simulations end when population size $\geq 10^6$.

```
ng <- 50000
u <- allFitnessEffects(noIntGenes = c(rep(0.1, ng/2),
                                      rep(-0.1, ng/2)))

t_mc_50000_nmpg <- system.time(
    mc_50000_nmpg <- oncoSimulPop(5,
                                  u,
                                  model = "McFL",
                                  mu = 1e-7,
                                  detectionSize = 1e6,
                                  detectionDrivers = NA,
                                  detectionProb = NA,
                                  keepPhylog = TRUE,
                                  onlyCancer = FALSE,
                                  keepEvery = NA,
                                  mutationPropGrowth = FALSE,
                                  mc.cores = 1
                                  ))
t_mc_50000_nmpg
##   user  system elapsed
##  30.46    0.54   31.01


summary(mc_50000_nmpg)[, c(1:3, 8, 9)]
##   NumClones TotalPopSize LargestClone FinalTime NumIter
## 1      1902      1002528       582752     284.2   31137
## 2      2159      1002679       404858     274.8   36905
## 3      2247      1002722       185678     334.5   42429
## 4      2038      1009606       493574     218.4   32519
## 5      2222      1004661       162628     291.0   38470

print(object.size(mc_50000_nmpg), units = "MB")
## 2057.6 Mb
```

We are already dealing with 2000 clones.

2.3.2.2 McFarland, 50,000 genes, example 2

Setting `keepEvery = 1` (i.e., keeping track of clones with an interval of 1):

```r
t_mc_50000_nmpg_k <- system.time(
    mc_50000_nmpg_k <- oncoSimulPop(5,
                                    u,
                                    model = "McFL",
                                    mu = 1e-7,
                                    detectionSize = 1e6,
                                    detectionDrivers = NA,
                                    detectionProb = NA,
                                    keepPhylog = TRUE,
                                    onlyCancer = FALSE,
                                    keepEvery = 1,
                                    mutationPropGrowth = FALSE,
                                    mc.cores = 1
                                    ))

t_mc_50000_nmpg_k
##    user  system elapsed
##  30.000   1.712  31.714

summary(mc_50000_nmpg_k)[, c(1:3, 8, 9)]
##   NumClones TotalPopSize LargestClone FinalTime NumIter
## 1      8779      1000223       136453     306.7   38102
## 2      7442      1006563       428150     345.3   35139
## 3      8710      1003509       224543     252.3   35659
## 4      8554      1002537       103889     273.7   36783
## 5      8233      1003171       263005     301.8   35236

print(object.size(mc_50000_nmpg_k), units = "MB")
## 8101.4 Mb
```

Computing time increases slightly but the major effect is seen on the size of the returned object, that increases by a factor of about 4x, up to 8 GB, corresponding to the increase in about 4x in the number of clones being tracked (see details of where the size of this object comes from in section 2.3.1.6).

2.3.2.3 McFarland, 50,000 genes, example 3

We will set `keepEvery = NA` again, but we will now increase detection size by a factor of 3 (so we stop when total population size becomes $\geq 3 * 10^6$).

```r
ng <- 50000
u <- allFitnessEffects(noIntGenes = c(rep(0.1, ng/2),
                                      rep(-0.1, ng/2)))

t_mc_50000_nmpg_3e6 <- system.time(
    mc_50000_nmpg_3e6 <- oncoSimulPop(5,
```

```
                                              u,
                                              model = "McFL",
                                              mu = 1e-7,
                                              detectionSize = 3e6,
                                              detectionDrivers = NA,
                                              detectionProb = NA,
                                              keepPhylog = TRUE,
                                              onlyCancer = FALSE,
                                              keepEvery = NA,
                                              mutationPropGrowth = FALSE,
                                              mc.cores = 1
                                              ))
t_mc_50000_nmpg_3e6
##    user  system elapsed
##  77.240   1.064  78.308

summary(mc_50000_nmpg_3e6)[, c(1:3, 8, 9)]
##   NumClones TotalPopSize LargestClone FinalTime NumIter
## 1      5487      3019083       836793     304.5   65121
## 2      4812      3011816       789146     286.3   53087
## 3      4463      3016896      1970957     236.6   45918
## 4      5045      3028142       956026     360.3   63464
## 5      4791      3029720       916692     358.1   55012

print(object.size(mc_50000_nmpg_3e6), units = "MB")
## 4759.3 Mb
```

Compared with the first run (2.3.2.1) we have approximately doubled computing time, number of iterations, number of clones, and object size.

2.3.2.4 McFarland, 50,000 genes, example 4

Let us use the same `detectionSize = 1e6` as in the first example (2.3.2.1), but with 5x the mutation rate:

```
t_mc_50000_nmpg_5mu <- system.time(
    mc_50000_nmpg_5mu <- oncoSimulPop(5,
                                      u,
                                      model = "McFL",
                                      mu = 5e-7,
                                      detectionSize = 1e6,
                                      detectionDrivers = NA,
                                      detectionProb = NA,
                                      keepPhylog = TRUE,
                                      onlyCancer = FALSE,
```

```
                                                keepEvery = NA,
                                                mutationPropGrowth = FALSE,
                                                mc.cores = 1
                                                ))

t_mc_50000_nmpg_5mu
##    user  system elapsed
## 167.332   1.796 169.167

summary(mc_50000_nmpg_5mu)[, c(1:3, 8, 9)]
##   NumClones TotalPopSize LargestClone FinalTime NumIter
## 1      7963      1004415       408352     99.03   57548
## 2      8905      1010751       120155    130.30   74738
## 3      8194      1005465       274661     96.98   58546
## 4      9053      1014049       119943    112.23   75379
## 5      8982      1011817        95047     99.95   76757

print(object.size(mc_50000_nmpg_5mu), units = "MB")
## 8314.4 Mb
```

The number of clones we are tracking is about 4x the number of clones of the first example (2.3.2.1), and roughly similar to the number of clones of the second example (2.3.2.2), and size of the returned object is similar to that of the second example. But computing time has increased by a factor of about 5x and iterations have increased by a factor of about 2x. Iterations increase because mutation is more frequent; in addition, at each sampling period each iteration needs to do more work as it needs to loop over a larger number of clones and this larger number includes clones that are not shown here, because they are pruned (they are extinct by the time we exit the simulation —again, pruning is discussed with further details in 12.2.1).

2.3.2.5 McFarland, 50,000 genes, example 5

Now let's run the above example but with `keepEvery = 1`:

```
t_mc_50000_nmpg_5mu_k <- system.time(
    mc_50000_nmpg_5mu_k <- oncoSimulPop(5,
                                        u,
                                        model = "McFL",
                                        mu = 5e-7,
                                        detectionSize = 1e6,
                                        detectionDrivers = NA,
                                        detectionProb = NA,
                                        keepPhylog = TRUE,
                                        onlyCancer = FALSE,
                                        keepEvery = 1,
                                        mutationPropGrowth = FALSE,
```

```
                                        mc.cores = 1
                                        ))

t_mc_50000_nmpg_5mu_k
##    user  system elapsed
## 174.404   5.068 179.481

summary(mc_50000_nmpg_5mu_k)[, c(1:3, 8, 9)]
##   NumClones TotalPopSize LargestClone FinalTime NumIter
## 1     25294      1001597       102766     123.4   74524
## 2     23766      1006679       223010     124.3   71808
## 3     21755      1001379       203638     114.8   62609
## 4     24889      1012103       161003     119.3   75031
## 5     21844      1002927       255388     108.8   64556

print(object.size(mc_50000_nmpg_5mu_k), units = "MB")
## 22645.8 Mb
```

We have already seen these effects before in section 2.3.2.2: using `keepEvery = 1` leads to a slight increase in execution time. What is really affected is the size of the returned object which increases by a factor of about 3x (and is now over 20GB). That 3x corresponds, of course, to the increase in the number of clones being tracked (now over 20,000). This, by the way, also allows us to understand the comment above, where we said that in these two cases (where we have increased mutation rate) at each iteration we need to do more work as at every update of the population the algorithm needs to loop over a much larger number of clones (even if many of those are eventually pruned).

2.3.2.6 McFarland, 50,000 genes, example 6

Finally, we will run the example in section 2.3.2.1 with the default of `mutationPropGrowth = TRUE`:

```
t_mc_50000 <- system.time(
    mc_50000 <- oncoSimulPop(5,
                             u,
                             model = "McFL",
                             mu = 1e-7,
                             detectionSize = 1e6,
                             detectionDrivers = NA,
                             detectionProb = NA,
                             keepPhylog = TRUE,
                             onlyCancer = FALSE,
                             keepEvery = NA,
                             mutationPropGrowth = TRUE,
```

```
                             mc.cores = 1
                             ))

t_mc_50000
##    user  system elapsed
## 303.352   2.808 306.223

summary(mc_50000)[, c(1:3, 8, 9)]
##   NumClones TotalPopSize LargestClone FinalTime NumIter
## 1     13928      1010815       219814     210.9   91255
## 2     12243      1003267       214189     178.1   67673
## 3     13880      1014131       124354     161.4   88322
## 4     14104      1012941        75521     205.7   98583
## 5     12428      1005594       232603     167.4   70359

print(object.size(mc_50000), units = "MB")
## 12816.6 Mb
```

Note the huge increase in computing time (related of course to the huge increase in number of iterations) and in the size of the returned object: we have gone from having to track about 2000 clones to tracking over 12000 clones even when we prune all clones without descendants.

2.3.3 Examples with $s = 0.05$

A script with the above runs but using $s = 0.05$ instead of $s = 0.1$ is available from the repository ('miscell-files/vignette_bench_Rout/large_num_genes_0.05.Rout'). I will single out a couple of cases here.

First, we repeat the run shown in section 2.3.2.5:

```
t_mc_50000_nmpg_5mu_k <- system.time(
    mc_50000_nmpg_5mu_k <- oncoSimulPop(2,
                                        u,
                                        model = "McFL",
                                        mu = 5e-7,
                                        detectionSize = 1e6,
                                        detectionDrivers = NA,
                                        detectionProb = NA,
                                        keepPhylog = TRUE,
                                        onlyCancer = FALSE,
                                        keepEvery = 1,
                                        mutationPropGrowth = FALSE,
                                        mc.cores = 1
                                        ))
t_mc_50000_nmpg_5mu_k
```

```
##    user  system elapsed
## 305.512   5.164 310.711

summary(mc_50000_nmpg_5mu_k)[, c(1:3, 8, 9)]
##   NumClones TotalPopSize LargestClone FinalTime NumIter
## 1     61737      1003273       104460  295.8731  204214
## 2     65072      1000540       133068  296.6243  210231

print(object.size(mc_50000_nmpg_5mu_k), units = "MB")
## 24663.6 Mb
```

Note we use only two replicates, since those two already lead to a 24 GB returned object as we are tracking more than 60,000 clones, more than twice those with $s = 0.1$. The reason for the difference in number of clones and iterations is of course the change from $s = 0.1$ to $s = 0.05$: under the McFarland model to reach population sizes of 10^6 starting from an equilibrium population of 500 we need about 43 mutations (whereas only about 22 are needed if $s = 0.1$[5]).

Next, let us rerun 2.3.2.1:

```
t_mc_50000_nmpg <- system.time(
    mc_50000_nmpg <- oncoSimulPop(5,
                                  u,
                                  model = "McFL",
                                  mu = 1e-7,
                                  detectionSize = 1e6,
                                  detectionDrivers = NA,
                                  detectionProb = NA,
                                  keepPhylog = TRUE,
                                  onlyCancer = FALSE,
                                  keepEvery = NA,
                                  mutationPropGrowth = FALSE,
                                  mc.cores = 1
                                  ))
t_mc_50000_nmpg
##    user  system elapsed
## 111.236   0.596 111.834

summary(mc_50000_nmpg)[, c(1:3, 8, 9)]
##   NumClones TotalPopSize LargestClone FinalTime NumIter
## 1      2646      1000700       217188   734.475  108566
## 2      2581      1001626       209873   806.500  107296
```

[5]Given the dependence of death rates on population size in McFarland's model (section 3.2.1), if all mutations have the same fitness effects we can calculate the equilibrium population size (where birth and death rates are equal) for a given number of mutated genes as: $K * (e^{(1+s)^p} - 1)$, where K is the initial equilibrium size, s the fitness effect of each mutation, and p the number of mutated genes.

```
## 3      2903      1001409       125148   841.700  120859
## 4      2310      1000146       473948   906.300   91519
## 5      2704      1001290       448409   838.800  103556

print(object.size(mc_50000_nmpg), units = "MB")
## 2638.3 Mb
```

Using $s = 0.05$ leads to a large increase in final time and number of iterations. However, as we are using the `keepEvery = NA` setting, the increase in number of clones tracked and in size of returned object is relatively small.

2.3.4 The different consequences of `keepEvery = NA` in the Exp and McFL models

We have seen that `keepEvery = NA` often leads to much smaller returned objects when using the McFarland model than when using the Exp model. Why? Because in the McFarland model there is strong competition and there can be complete clonal sweeps so that in extreme cases a single clone might be all that is left after some time. This is not the case in the exponential models.

Of course, the details depend on the difference in fitness effects between different genotypes (or clones). In particular, we have seen several examples where even with `keepEvery=NA` there are a lot of clones in the McFL models. In those examples many clones had identical fitness (the fitness effects of all genes with positive fitness was the same, and ditto for the genes with negative fitness effects), so no clone ends up displacing all the others.

2.3.5 Are we keeping the complete history (genealogy) of the clones?

Yes we are if we run with `keepPhylog = TRUE`, regardless of the setting for `keepEvery`. As explained in section 12.2, OncoSimulR prunes clones that never had a population size larger than zero at any sampling period (so they are not reflected in the `pops.by.time` matrix in the output). And when we set `keepEvery = NA` we are telling OncoSimulR to discard all sampling periods except the very last one (i.e., the `pops.by.time` matrix contains only the clones with 1 or more cells at the end of the simulation).

`keepPhylog` operates differently: it records the exact time at which a clone appeared and the clone that gave rise to it. This information is kept regardless of whether or not those clones appear in the `pops.by.time` matrix.

Keeping the complete genealogy might be of limited use if the `pops.by.time` matrix only contains the very last period. However, you can use `plotClonePhylog` and ask to be shown only clones that exist in the very last period (while of course showing all of their ancestors, even if those are now extinct —i.e., regardless of their abundance).

For instance, in run 2.3.1.3 we could have looked at the information stored about the genealogy of clones by doing (we look at the first "individual" of the simulation, of the five "individuals" we simulated):

```
head(e_50000[[1]]$other$PhylogDF)
##   parent child   time
## 1          3679 0.8402
## 2          4754 1.1815
## 3         20617 1.4543
## 4         15482 2.3064
## 5          4431 3.7130
## 6         41915 4.0628

tail(e_50000[[1]]$other$PhylogDF)
##                            parent                                  child time
## 20672                 3679, 20282                    3679, 20282, 22359 75.0
## 20673          3679, 17922, 22346             3679, 17922, 22346, 35811 75.0
## 20674                  2142, 3679                     2142, 3679, 25838 75.0
## 20675          3679, 17922, 19561             3679, 17922, 19561, 43777 75.0
## 20676 3679, 15928, 19190, 20282 3679, 15928, 19190, 20282, 49686 75.0
## 20677           2142, 3679, 16275              2142, 3679, 16275, 24201 75.0
```

where each row corresponds to one event of appearance of a new clone, the column labeled "parent" are the mutated genes in the parent, and the column labeled "child" are the mutated genes in the child.

And we could plot the genealogical relationships of clones that have a population size of at least one in the last period (again, while of course showing all of their ancestors, even if those are now extinct —i.e., regardless of their current numbers) doing:

```
plotClonePhylog(e_50000[[1]]) ## plot not shown
```

What is the cost of keep the clone genealogies? In terms of time it is minor. In terms of space, and as shown in the example above, we can end up storing a data frame with tends of thousands of rows and three columns (two factors, one float). In the example above the size of that data frame is approximately 2 MB for a single simulation. This is much smaller than the `pops.by.time` or `Genotypes` matrices, but it can quickly build up if you routinely launch, say, 1000 simulations via `oncoSimulPop`. That is why the default is `keepPhylog = FALSE` as this information is not needed as often as that in the other two matrices (`pops.by.time` and `Genotypes`). However, if you plan to measure evolutionary predictability using Lines of Descent, or LOD (section 10) you should run simulations with `keepPhylog = TRUE`.

2.4 Population sizes $\geq 10^{10}$

We have already seen examples where population sizes reach 10^8 to 10^{10}, as in Tables 3, 5, 7. What about even larger population sizes?

The C++ code will unconditionally alert if population sizes exceed $4 * 10^{15}$ as in those cases loosing precision (as we are using doubles) would be unavoidable, and we would also run into problems with the generation of binomial random variates (code that illustrates and discusses this problem is available in file "example-binom-problems.cpp", in directory "/inst/miscell"). However, well before we reach $4 * 10^{15}$ we loose precision from other sources. One of the most noticeable ones is that when we reach population sizes around 10^{11} the C++ code will often alert us by throwing exceptions with the message `Recoverable exception ti set to DBL_MIN. Rerunning.` I throw this exception because t_i, the random variable for time to next mutation, is less than `DBL_MIN`, the minimum representable floating-point number. This happens because, unless we use really tiny mutation rates, the time to a mutation starts getting closer to zero as population sizes grow very large. It might be possible to ameliorate these problems somewhat by using long doubles (instead of doubles) or special purpose libraries that provide more precision. However, this would make it harder to run the same code in different operating systems and would likely decrease execution speed on the rest of the common scenarios for which OncoSimulR has been designed.

The following code shows some examples where we use population sizes of 10^{10} or larger. Since we do not want simulations in the exponential model to end because of extinction, I use a fitness specification where all genes have a positive fitness effect and we start all simulations from a large population (to make it unlikely that the population will become extinct before cells mutate and start increasing in numbers). We set the maximum running time to 10 minutes. We keep the genealogy of the clones and use `keepEvery = 1`.

```
ng <- 50
u <- allFitnessEffects(noIntGenes = c(rep(0.1, ng)))
```

```
t_mc_k_50_1e11 <- system.time(
    mc_k_50_1e11 <- oncoSimulPop(5,
                                 u,
                                 model = "McFL",
                                 mu = 1e-7,
                                 detectionSize = 1e11,
                                 initSize = 1e5,
                                 detectionDrivers = NA,
                                 detectionProb = NA,
                                 keepPhylog = TRUE,
                                 onlyCancer = FALSE,
                                 mutationPropGrowth = FALSE,
                                 keepEvery = 1,
                                 finalTime = 5000,
                                 mc.cores = 1,
                                 max.wall.time = 600
                                 ))
```

```
## Recoverable exception ti set to DBL_MIN. Rerunning.
## Recoverable exception ti set to DBL_MIN. Rerunning.

t_mc_k_50_1e11
## user  system elapsed
## 613.612   0.040 613.664

summary(mc_k_50_1e11)[, c(1:3, 8, 9)]
##   NumClones TotalPopSize LargestClone FinalTime NumIter
## 1      5491 100328847809  44397848771  1019.950  942764
## 2      3194 100048090441  34834178374   789.675  888819
## 3      5745 100054219162  24412502660   927.950  929231
## 4      4017 101641197799  60932177160   750.725  480938
## 5      5393 100168156804  41659212367   846.250  898245

## print(object.size(mc_k_50_1e11), units = "MB")
## 177.8 Mb
```

We get to 10^{11}. But notice the exception with the warning about t_i. Notice also that this takes a long time and we run a very large number of iterations (getting close to one million in some cases).

Now the exponential model with `detectionSize = 1e11`:

```
t_exp_k_50_1e11 <- system.time(
    exp_k_50_1e11 <- oncoSimulPop(5,
                                  u,
                                  model = "Exp",
                                  mu = 1e-7,
                                  detectionSize = 1e11,
                                  initSize = 1e5,
                                  detectionDrivers = NA,
                                  detectionProb = NA,
                                  keepPhylog = TRUE,
                                  onlyCancer = FALSE,
                                  mutationPropGrowth = FALSE,
                                  keepEvery = 1,
                                  finalTime = 5000,
                                  mc.cores = 1,
                                  max.wall.time = 600,
                                  errorHitWallTime = FALSE,
                                  errorHitMaxTries = FALSE
                                  ))

## Recoverable exception ti set to DBL_MIN. Rerunning.
## Hitted wall time. Exiting.
## Recoverable exception ti set to DBL_MIN. Rerunning.
```

```
## Recoverable exception ti set to DBL_MIN. Rerunning.
## Recoverable exception ti set to DBL_MIN. Rerunning.
## Hitted wall time. Exiting.
## Recoverable exception ti set to DBL_MIN. Rerunning.
## Recoverable exception ti set to DBL_MIN. Rerunning.
## Recoverable exception ti set to DBL_MIN. Rerunning.
## Recoverable exception ti set to DBL_MIN. Rerunning.
## Recoverable exception ti set to DBL_MIN. Rerunning.
## Hitted wall time. Exiting.
## Hitted wall time. Exiting.

t_exp_k_50_1e11
##     user   system  elapsed
## 2959.068    0.128 2959.556
try(summary(exp_k_50_1e11)[, c(1:3, 8, 9)])
##   NumClones TotalPopSize LargestClone FinalTime NumIter
## 1      6078  65172752616  16529682757  235.7590 1883438
## 2      5370 106476643712  24662446729  232.0000 2516675
## 3      2711  21911284363  17945303353  224.8608  543698
## 4      2838  13241462284   2944300245  216.8091  372298
## 5      7289  76166784312  10941729810  240.0217 1999489

print(object.size(exp_k_50_1e11), units = "MB")
## 53.5 Mb
```

Note that we almost reached `max.wall.time` (600 * 5 = 3000). What if we wanted to go up to 10^{12}? We would not be able to do it in 10 minutes. We could set `max.wall.time` to a value larger than 600 to allow us to reach larger sizes but then we would be waiting for a possibly unacceptable time for simulations to finish. Moreover, this would eventually fail as simulations would keep hitting the t_i exception without ever being able to complete. Finally, even if we were very patient, hitting that t_i exception should make us worry about possible biases in the samples.

2.5 A summary of some determinants of running time and space consumption

To summarize this section, we have seen:

- Both McFL and Exp can be run in short times over a range of sizes for the `detectionProb` and `detectionSize` mechanisms using a complex fitness specification with moderate numbers of genes. These are the typical or common use cases of OncoSimulR.
- The `keepEvery` argument can have a large effect on time in the McFL models and specially on object sizes. If only the end result of the simulation is to be used, you should set `keepEvery = NA`.

- The distribution of fitness effects and the fitness landscape can have large effects on running times. Sometimes these are intuitive and simple to reason about, sometimes they are not as they interact with other factors (e.g., stopping mechanism, numbers of clones, etc). In general, there can be complex interactions between different settings, from mutation rate to fitness effects to initial size. As usual, test before launching a massive simulation.
- Simulations start to slow down and lead to a very large object size when we keep track of around 6000 to 10000 clones. Anything that leads to these patterns will slow down the simulations.
- OncoSimulR needs to keep track of genotypes (or clones), not just numbers of drivers and passengers, because it allows you to use complex fitness and mutation specifications that depend on specific genotypes. The `keepEvery = NA` is an approach to store only the minimal information needed, but it is unavoidable that during the simulations we might be forced to deal with many thousands of different clones.

3 Specifying fitness effects

OncoSimulR uses a standard continuous time model, where individual cells divide, die, and mutate with rates that can depend on genotype and population size; over time the abundance of the different genotypes changes by the action of selection (due to differences in net growth rates among genotypes), drift, and mutation. As a result of a mutation in a pre-existing clone new clones arise, and the birth rate of a newly arisen clone is determined at the time of its emergence as a function of its genotype. Simulations can use an use exponential growth model or a model with carrying capacity that follows McFarland et al. (2013). For the exponential growth model, the death rate is fixed at one whereas in the model with carrying capacity death rate increases with population size. In both cases, therefore, fitness differences among genotypes in a given population at a given time are due to differences in the mapping between genotype and birth rate. There is second exponential model (called "Bozic") where birth rate is fixed at one, and genotype determines death rate instead of birth rate (see details in 3.2). So when we discuss specifying fitness effects or the effects of genes on fitness, we are actually referring to specifying effects on birth (or death) rates, which then translate into differences in fitness (since the other rate, death or birth, is either fixed, as in the Exp and Bozic models, or depends on the population size). This is also shown in Table 1, in the rows for "Fitness components", under "Evolutionary Features".

3.1 Introduction to the specification of fitness effects

With OncoSimulR you can specify different types of effects on fitness:

- A special type of epistatic effect that is particularly amenable to be represented as a graph (a DAG). In this graph having, say, "B" be a child of "A" means that a mutation in B can only accumulate if a mutation in A is already present. This is what OT (Desper et al. 1999; Szabo and Boucher 2008), CBN (Beerenwinkel, Eriksson, and Sturmfels 2007; Gerstung et al. 2009; Gerstung et al. 2011), progression networks (Farahani and Lagergren 2013), and other similar models (Korsunsky et al. 2014) generally mean. Details are provided in section 3.4. Note that this is not an order effect (discussed below): the fitness of a genotype from this DAGs is a function of whether or not the restrictions in the graph are satisfied, not the historical sequence of how they were satisfied.
- Effects where the order in which mutations are acquired matters, as illustrated in section 3.6. There is, in fact, empirical evidence of these effects (Ortmann et al. 2015). For instance, the fitness of genotype "A, B" would differ depending on whether A or B was acquired first (or, as in the actual example in (Ortmann et al. 2015), the fitness of the mutant with JAK2 and TET2 mutated will depend on which of the genes was mutated first).
- General epistatic effects (e.g., section 3.7), including synthetic viability (e.g., section 3.9) and synthetic lethality/mortality (e.g., section 3.10).

- Genes that have independent effects on fitness (section 3.3).
- Modules (see section 3.5) allow you to specify any of the above effects (except those for genes without interactions, as it would not make sense there) in terms of modules (sets of genes), not individual genes. We will introduce them right after 3.4, and we will continue using them thereafter.

A guiding design principle of OncoSimulR is to try to make the specification of those effects as simple as possible but also as flexible as possible. Thus, there are two main ways of specifying fitness effects:

- Combining different types of effects in a single specification. For instance, you can combine epistasis with order effects with no interaction genes with modules. What you would do here is specify the effects that different mutations (or their combinations) have on fitness (the fitness effects) and then have OncoSimulR take care of combining them as if each of these were lego pieces. We will refer to this as the **lego system of fitness effects**. (As explained above, I find this an intuitive and very graphical analogy, which I have copied from Hothorn et al. (2006) and Hothorn et al. (2008)).
- Explicitly passing to OncoSimulR a mapping of genotypes to fitness. Here you specify the fitness of each genotype. We will refer to this as the **explicit mapping of genotypes to fitness**.

Both approaches have advantages and disadvantages. Here I emphasize some relevant differences.

- With the lego system you can specify huge genomes with an enormous variety of interactions, since the possible genotypes are not constructed in advance. You would not be able to do this with the explicit mapping of genotypes to fitness if you wanted to, say, construct that mapping for a modest genotype of 500 genes (you'd have more genotypes than particles in the observable Universe).
- For many models/data you often intuitively start with the fitness of the genotypes, not the fitness consequences of the different mutations. In these cases, you'd need to do the math to specify the terms you want if you used the lego system so you'll probably use the specification with the direct mapping genotype $\rightarrow$ fitness.
- Likewise, sometimes you already have a moderate size genotype $\rightarrow$ fitness mapping and you certainly do not want to do the math by hand: here the lego system would be painful to use.
- But sometimes we do think in terms of "the effects on fitness of such and such mutations are" and that immediately calls for the lego system, where you focus on the effects, and let OncoSimulR take care of doing the math of combining.
- If you want to use order effects, you must use the lego system (at least for now).
- If you want to specify modules, you must use the lego system (the explicit mapping of genotypes is, by its very nature, ill-suited for this).

- The lego system might help you see what your model really means: in many cases, you can obtain fairly succinct specifications of complex fitness models with just a few terms. Similarly, depending on what your emphasis is, you can often specify the same fitness landscape in several different ways.

Regardless of the route, you need to get that information into OncoSimulR's functions. The main function we will use is `allFitnessEffects`: this is the function in charge of reading the fitness specifications. We also need to discuss how, what, and where you have to pass to `allFitnessEffects`.

3.1.1 Explicit mapping of genotypes to fitness

Conceptually, the simplest way to specify fitness is to specify the mapping of all genotypes to fitness explicitly. An example will make this clear. Let's suppose you have a simple two-gene scenario, so a total of four genotypes, and you have a data frame with genotypes and fitness, where genoytpes are specified as character vectors, with mutated genes separated by commas:

```
m4 <- data.frame(G = c("WT", "A", "B", "A, B"), F = c(1, 2, 3, 4))
```

Now, let's give that to the `allFitnessEffects` function:

```
fem4 <- allFitnessEffects(genotFitness = m4)
## Column names of object not Genotype and Fitness. Renaming them assuming that is
```

(The message is just telling you what the program guessed you wanted.)

That's it. You can plot that fitnessEffects object

```
plot(fem4)
```

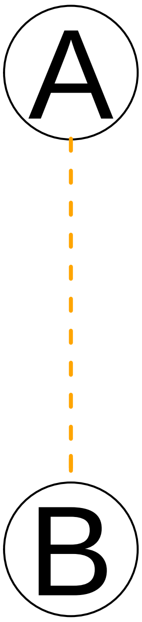

In this case, that plot is not very interesting (compare with the `plot(pancr)` we

saw in 1.6 or the plots in 3.4).

You can also check what OncoSimulR thinks the fitnesses are, with the `evalAllGenotypes` function that we will use repeatedly below (of course, here we should see the same fitnesses we entered):

```
evalAllGenotypes(fem4, addwt = TRUE)
##   Genotype Fitness
## 1       WT       1
## 2        A       2
## 3        B       3
## 4     A, B       4
```

And you can plot the fitness landscape:

```
plotFitnessLandscape(evalAllGenotypes(fem4))
```

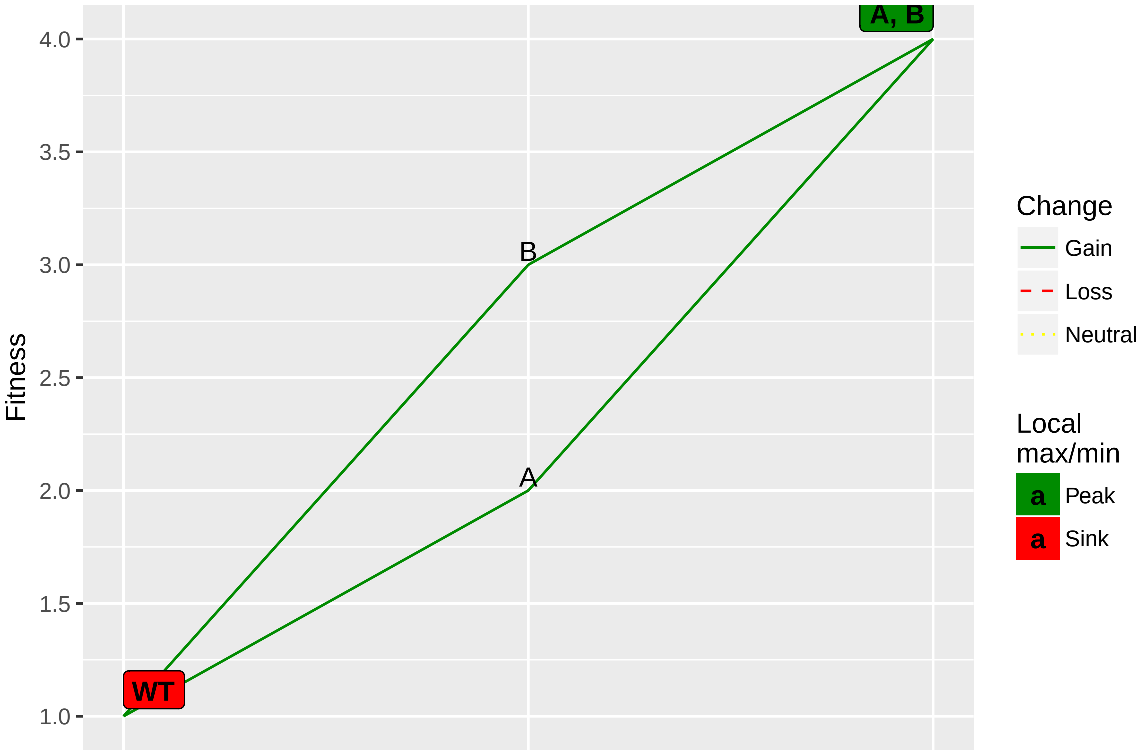

To specify the mapping you can also use a matrix (or data frame) with $g+1$ columns; each of the first g columns contains a 1 or a 0 indicating that the gene of that column is mutated or not. Column $g+1$ contains the fitness values. And you do not even need to specify all the genotypes (we will assume that the missing genotypes have fitness 1):

```
m6 <- cbind(c(1, 1), c(1, 0), c(2, 3))
fem6 <- allFitnessEffects(genotFitness = m6)
## Number of genotypes less than 2^L. Missing genotype will be set to 1
## Setting/resetting gene names because one or more are missing. If this is not wha
evalAllGenotypes(fem6, addwt = TRUE)
##   Genotype Fitness
```

```
## 1        WT        1
## 2         A        3
## 3         B        1
## 4      A, B        2
plot(fem6)
```

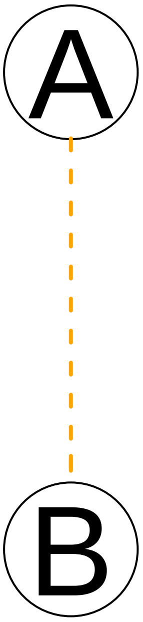


```
plotFitnessLandscape(evalAllGenotypes(fem6))
```

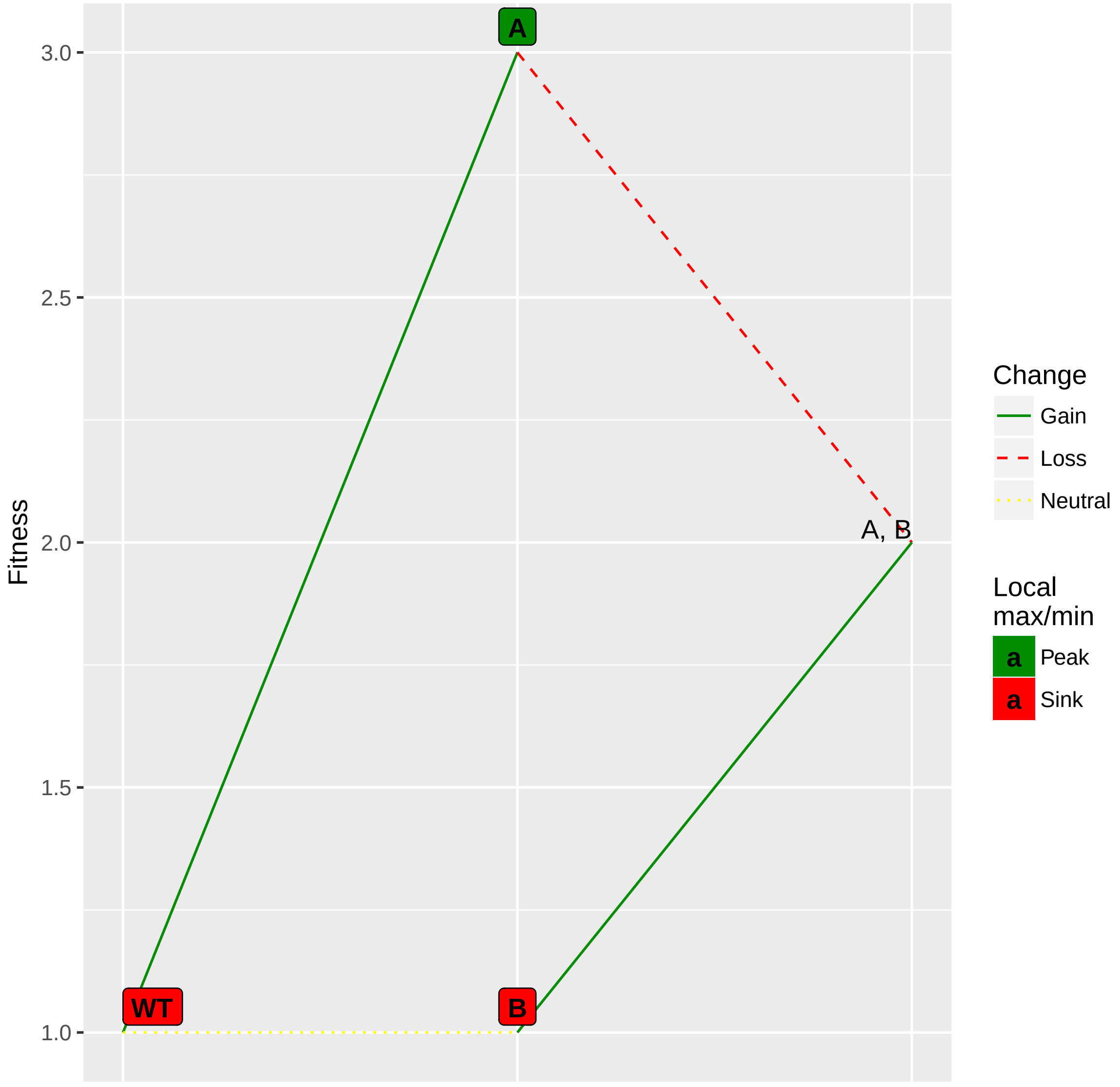

This way of giving a fitness specification to OncoSimulR might be ideal if you directly generate random mappings of genotypes to fitness (or random fitness landscapes), as we will do in section 9.

We will see an example of this way of passing fitness again in 5.1, where we will compare it with the lego system.

3.1.2 How to specify fitness effects with the lego system

An alternative general approach followed in many genetic simulators is to specify how particular combinations of alleles modify the wildtype genotype or the genotype that contains the individual effects of the interacting genes (e.g., see equation 1 in the supplementary material for FFPopSim (Zanini and Neher 2012)). For example, if we specify that a mutation in "A" contributes 0.04, a mutation in "B" contributes 0.03, and the double mutation "A:B" contributes 0.1, that means that the fitness of the "A, B" genotype (the genotype with A and B mutated) is that of the wildtype (1, by default), plus (actually, times —see section 3.2— but plus on the log scale)

the effects of having A mutated, plus (times) the effects of having B mutated, plus (times) the effects of "A:B" both being mutated.

We will see below that with the "lego system" it is possible to do something very similar to the explicit mapping of section 3.1.1. But this will sometimes require a more cumbersome notation (and sometimes also will require your doing some math). We will see examples in sections 3.7.1, 3.7.2 and 3.7.3 or the example in 5.4.2. But then, if we can be explicit about (at least some of) the mappings $genotype \rightarrow fitness$, how are these procedures different? When you use the "lego system" you can combine both a partial explicit mapping of genotypes to fitness with arbitrary fitness effects of other genes/modules. In other words, with the "lego system" OncoSimulR makes it simple to be explicit about the mapping of specific genotypes, while also using the "how this specific effects modifies previous effects" logic, leading to a flexible specification. This also means that in many cases the same fitness effects can be specified in several different ways.

Most of the rest of this section is devoted to explaining how to combine those pieces. Before that, however, we need to discuss the fitness model we use.

3.2 Numeric values of fitness effects

We evaluate fitness using the usual (Zanini and Neher 2012; Gillespie 1993; Beerenwinkel, Eriksson, and Sturmfels 2007; Datta et al. 2013) multiplicative model: fitness is $\prod(1 + s_i)$ where s_i is the fitness effect of gene (or gene interaction) i. In all models except Bozic, this fitness refers to the growth rate (the death rate being fixed to 1[6]). The original model of McFarland et al. (2013) has a slightly different parameterization, but you can go easily from one to the other (see section 3.2.1).

For the Bozic model (Bozic et al. 2010), however, the birth rate is set to 1, and the death rate then becomes $\prod(1 - s_i)$.

3.2.1 McFarland parameterization

In the original model of McFarland et al. (2013), the effects of drivers contribute to the numerator of the birth rate, and those of the (deleterious) passengers to the denominator as: $\frac{(1+s)^d}{(1+s_p)^p}$, where d and p are, respectively, the total number of drivers and passengers in a genotype, and here the fitness effects of all drivers is the same (s) and that of all passengers the same too (s_p). Note that, as written above, and as explicitly said in McFarland et al. (2013) (see p. 2911) and McFarland (2014) (see p. 9), "(...) s_p is the fitness disadvantage conferred by a passenger". In other words, the larger the s_p the more deleterious the passenger.

This is obvious, but I make it explicit because in our parameterization a positive s means fitness advantage, whereas fitness disadvantages are associated with negative

[6]You can change this if you really want to.

s. Of course, if you rewrite the above expression as $\frac{(1+s)^d}{(1-s_p)^p}$ then we are back to the "positive means fitness advantage and negative means fitness disadvantage".

As McFarland (2014) explains (see p. 9, bottom), we can rewrite the above expression so that there are no terms in the denominator. McFarland writes it as (I copy verbatim from the fourth and fifth lines from the bottom on his p. 9) $(1 + s_d)^{n_d}(1 - s_p^{'})^{n_p}$ where $s_p^{'} = s_p/(1 + s_p)$.

However, if we want to express everything as products (no ratios) and use the "positive s means advantage and negative s means disadvantage" rule, we want to write the above expression as $(1 + s_d)^{n_d}(1 + s_{pp})^{n_p}$ where $s_{pp} = -s_p/(1 + s_p)$. And this is actually what we do in v.2. There is an example, for instance, in section 6.5.2 where you will see:

```
sp <- 1e-3
spp <- -sp/(1 + sp)
```

so we are going from the "(...) s_p is the fitness disadvantage conferred by a passenger" in McFarland et al. (2013) (p. 2911) and McFarland (2014) (p. 9) to the expression where we have a product $\prod(1+s_i)$, with the "positive s means advantage and negative s means disadvantage" rule. This reparameterization applies to v.2. In v.1 we use the same parameterization as in the original one in McFarland et al. (2013), but with the "positive s means advantage and negative s means disadvantage" rule (so we are using expression $\frac{(1+s)^d}{(1-s_p)^p}$).

For death rate, we use the expression that McFarland et al. (2013) (see their p. 2911) use "(...) for large cancers (grown to 10^6 cells)": $D(N) = \log(1 + N/K)$ where K is the initial equilibrium population size. As the authors explain, for large N/K the above expression "(...) recapitulates Gompertzian dynamics observed experimentally for large tumors".

By default, OncoSimulR uses a value of $K = initSize/(e^1 - 1)$ so that the starting population is at equilibrium.

3.2.2 No viability of clones and types of models

For all models where fitness affects directly the birth rate (all except Bozic), if you specify that some event (say, mutating gene A) has $s_A \leq -1$, if that event happens then birth rate becomes zero. This is taken to indicate that the clone is not even viable and thus disappears immediately without any chance for mutation[7].

[7] This is a shortcut that we take because we think that it is what you mean. Note, however, that technically a clone with birth rate of 0 might have a non-zero probability of mutating before becoming extinct because in the continuous time model we use mutation is not linked to reproduction. In the present code, we are not allowing for any mutation when birth rate is 0. There are other options, but none which I find really better. An alternative implementation makes a clone immediately extinct if and only if any of the $s_i = -\infty$. However, we still need to handle the case with $s_i < -1$ as a special case. We either make it identical to the case with any $s_i = -\infty$ or for any $s_i > -\infty$ we set $(1 + s_i) = \max(0, 1 + s_i)$ (i.e., if $s_i < -1$ then $(1 + s_i) = 0$), to avoid obtaining negative birth rates (that make no sense) and the problem of multiplying an even number of negative numbers. I

Models based on Bozic, however, have a birth rate of 1 and mutations affect the death rate. In this case, a death rate larger than birth rate, *per se*, does not signal immediate extinction and, moreover, even for death rates that are a few times larger than birth rates, the clone could mutate before becoming extinct[8].

In general, if you want to identify some mutations or some combinations of mutations as leading to immediate extinction (i.e., no viability), of the affected clone, set it to $-\infty$ as this would work even if how birth rates of 0 are handled changes. Most examples below evaluate fitness by its effects on the birth rate. You can see one where we do it both ways in Section 3.11.1.

3.3 Genes without interactions

This is a simple scenario. Each gene i has a fitness effect s_i if mutated. The s_i can come from any distribution you want. As an example let's use three genes. We know there are no order effects, but we will also see what happens if we examine genotypes as ordered.

```
ai1 <- evalAllGenotypes(allFitnessEffects(
    noIntGenes = c(0.05, -.2, .1)), order = FALSE)
```

We can easily verify the first results:

```
ai1
##   Genotype Fitness
## 1        1   1.050
## 2        2   0.800
## 3        3   1.100
## 4     1, 2   0.840
## 5     1, 3   1.155
## 6     2, 3   0.880
## 7  1, 2, 3   0.924
```

```
all(ai1[, "Fitness"]  == c( (1 + .05), (1 - .2), (1 + .1),
       (1 + .05) * (1 - .2),
       (1 + .05) * (1 + .1),
       (1 - .2) * (1 + .1),
       (1 + .05) * (1 - .2) * (1 + .1)))
## [1] TRUE
```

And we can see that considering the order of mutations (see section 3.6) makes no difference:

think only the second would make sense as an alternative.

[8]We said "a few times". For a clone of population size 1 —which is the size at which all clones start from mutation—, if death rate is, say, 90 but birth rate is 1, the probability of mutating before becoming extinct is very, very close to zero for all reasonable values of mutation rate}. How do we signal immediate extinction or no viability in this case? You can set the value of $s = -\infty$.

```r
(ai2 <- evalAllGenotypes(allFitnessEffects(
    noIntGenes = c(0.05, -.2, .1)), order = TRUE,
    addwt = TRUE))
##      Genotype Fitness
## 1          WT   1.000
## 2           1   1.050
## 3           2   0.800
## 4           3   1.100
## 5       1 > 2   0.840
## 6       1 > 3   1.155
## 7       2 > 1   0.840
## 8       2 > 3   0.880
## 9       3 > 1   1.155
## 10      3 > 2   0.880
## 11  1 > 2 > 3   0.924
## 12  1 > 3 > 2   0.924
## 13  2 > 1 > 3   0.924
## 14  2 > 3 > 1   0.924
## 15  3 > 1 > 2   0.924
## 16  3 > 2 > 1   0.924
```

(The meaning of the notation in the output table is as follows: "WT" denotes the wild-type, or non-mutated clone. The notation $x > y$ means that a mutation in "x" happened before a mutation in "y". A genotype $x > y \_ z$ means that a mutation in "x" happened before a mutation in "y"; there is also a mutation in "z", but that is a gene for which order does not matter).

And what if I want genes without interactions but I want modules (see section 3.5)? Go to section 3.8.

3.4 Using DAGs: Restrictions in the order of mutations as extended posets

3.4.1 AND, OR, XOR relationships

The literature on Oncogenetic trees, CBNs, etc, has used graphs as a way of showing the restrictions in the order in which mutations can accumulate. The meaning of "convergent arrows" in these graphs, however, differs. In Figure 1 of Korsunsky et al. (2014) we are shown a simple diagram that illustrates the three basic different meanings of convergent arrows using two parental nodes. We will illustrate it here with three. Suppose we focus on node "g" in the following figure (we will create it shortly)

```r
data(examplesFitnessEffects)
plot(examplesFitnessEffects[["cbn1"]])
```

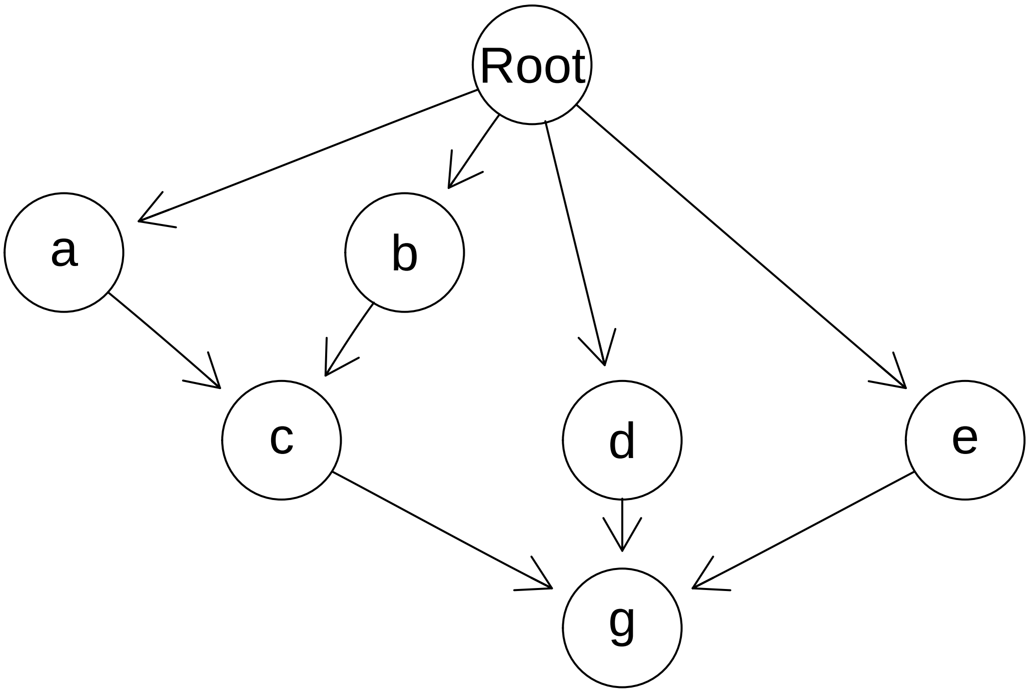

- In relationships of the type used in **Conjunctive Bayesian Networks (CBN)** (e.g., Gerstung et al. 2009), we are modeling an **AND** relationship, also called **CMPN** by Korsunsky et al. (2014) or **monotone** relationship by Farahani and Lagergren (2013). If the relationship in the graph is fully respected, then "g" will only appear if all of "c", "d", and "e" are already mutated.
- **Semimonotone** relationships *sensu* Farahani and Lagergren (2013) or **DMPN** *sensu* Korsunsky et al. (2014) are **OR** relationships: "g" will appear if one or more of "c", "d", or "e" are already mutated.
- **XMPN** relationships (Korsunsky et al. 2014) are **XOR** relationships: "g" will be present only if exactly one of "c", "d", or "e" is present.

Note that Oncogenetic trees (Desper et al. 1999; Szabo and Boucher 2008) need not deal with the above distinctions, since the DAGs are trees: no node has more than one incoming connection or more than one parent[9].

To have a flexible way of specifying all of these restrictions, we will want to be able to say what kind of dependency each child node has on its parents.

3.4.2 Fitness effects

Those DAGs specify dependencies and, as explained in Diaz-Uriarte (2015), it is simple to map them to a simple evolutionary model: any set of mutations that does not conform to the restrictions encoded in the graph will have a fitness of 0. However, we might not want to require absolute compliance with the DAG. This means we

[9]OTs and CBNs have some other technical differences about the underlying model they assume, such as the exponential waiting time in CBNs. We will not discuss them here.

might want to allow deviations from the DAG with a corresponding penalization that is, however, not identical to setting fitness to 0 (again, see Diaz-Uriarte 2015). This we can do by being explicit about the fitness effects of the deviations from the restrictions encoded in the DAG. We will use below a column of `s` for the fitness effect when the restrictions are satisfied and a column of `sh` when they are not. (See also 3.2 for the details about the meaning of the fitness effects).

That way of specifying fitness effects makes it also trivial to use the model in Hjelm, Höglund, and Lagergren (2006) where all mutations might be allowed to occur, but the presence of some mutations increases the probability of occurrence of other mutations. For example, the values of `sh` could be all small positive ones (or for mildly deleterious effects, small negative numbers), while the values of `s` are much larger positive numbers.

3.4.3 Extended posets

In version 1 of this package we used posets in the sense of Beerenwinkel, Eriksson, and Sturmfels (2007) and Gerstung et al. (2009), as explained in section 13.1 and in the help for `poset`. Here, we continue using two columns, that specify parents and children, but we add columns for the specific values of fitness effects (both s and sh —i.e., fitness effects for what happens when restrictions are and are not satisfied) and for the type of dependency as explained in section 3.4.1.

We can now illustrate the specification of different fitness effects using DAGs.

3.4.4 DAGs: A first conjunction (AND) example

```
cs <-  data.frame(parent = c(rep("Root", 4), "a", "b", "d", "e", "c"),
                  child = c("a", "b", "d", "e", "c", "c", rep("g", 3)),
                  s = 0.1,
                  sh = -0.9,
                  typeDep = "MN")

cbn1 <- allFitnessEffects(cs)
```

(We skip one letter, just to show that names need not be consecutive or have any particular order.)

We can get a graphical representation using the default "graphNEL"

```
plot(cbn1)
```

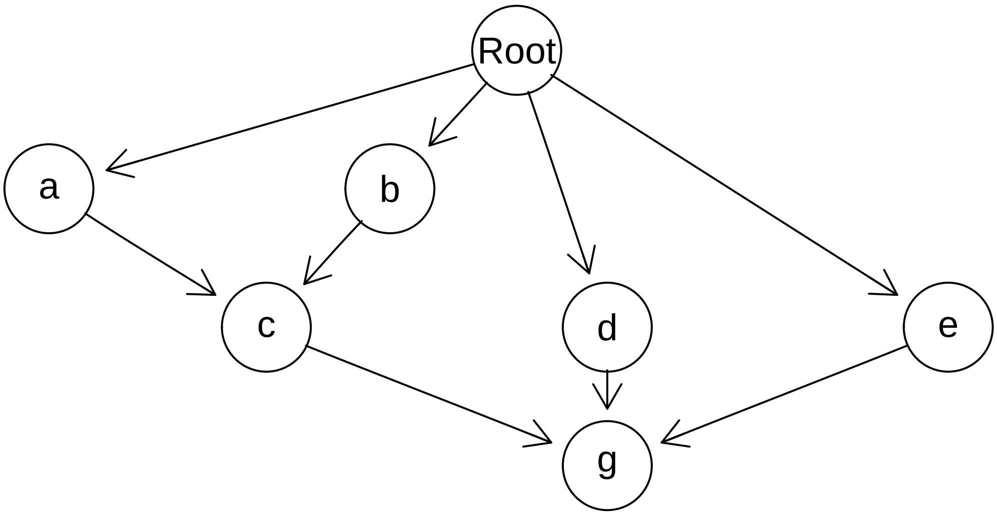

or one using “igraph”:

```
plot(cbn1, "igraph")
```

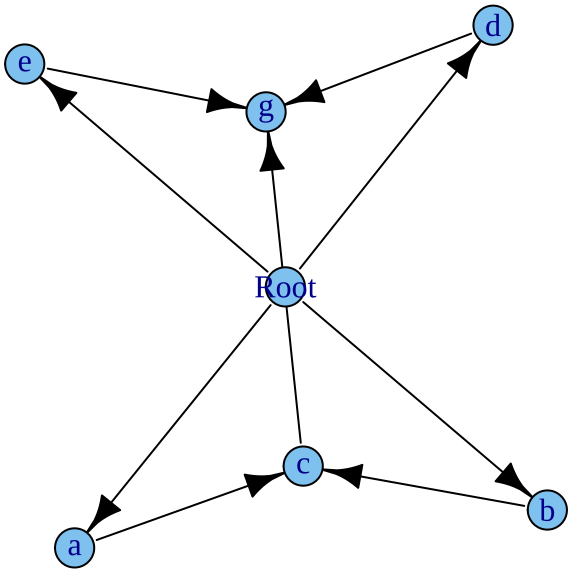

Since we have a parent and children, the reingold.tilford layout is probably the best here, so you might want to use that:

```
library(igraph) ## to make the reingold.tilford layout available
plot(cbn1, "igraph", layout = layout.reingold.tilford)
```

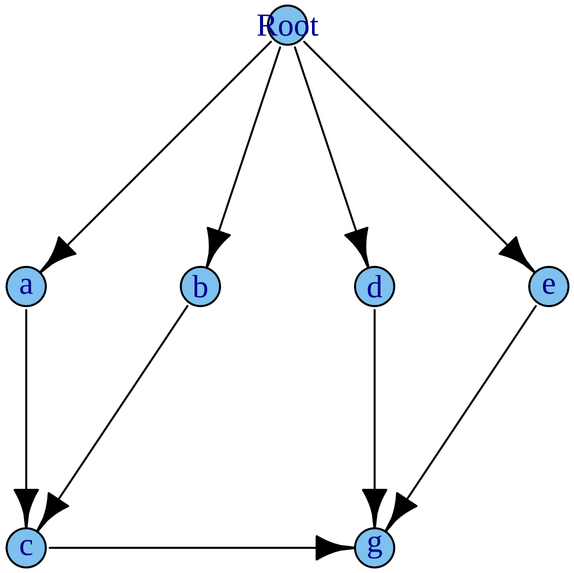

And what is the fitness of all genotypes?

```
gfs <- evalAllGenotypes(cbn1, order = FALSE, addwt = TRUE)

gfs[1:15, ]
##    Genotype Fitness
## 1        WT    1.00
## 2         a    1.10
## 3         b    1.10
## 4         c    0.10
## 5         d    1.10
## 6         e    1.10
## 7         g    0.10
## 8      a, b    1.21
## 9      a, c    0.11
## 10     a, d    1.21
## 11     a, e    1.21
## 12     a, g    0.11
## 13     b, c    0.11
## 14     b, d    1.21
## 15     b, e    1.21
```

You can verify that for each genotype, if a mutation is present without all of its dependencies present, you get a $(1-0.9)$ multiplier, and you get a $(1+0.1)$ multiplier for all the rest with its direct parents satisfied. For example, genotypes "a", or "b", or "d", or "e" have fitness $(1+0.1)$, genotype "a, b, c" has fitness $(1+0.1)^3$, but genotype "a, c" has fitness $(1+0.1)(1-0.9) = 0.11$.

3.4.5 DAGs: A second conjunction example

Let's try a first attempt at a somewhat more complex example, where the fitness consequences of different genes differ.

```
c1 <- data.frame(parent = c(rep("Root", 4), "a", "b", "d", "e", "c"),
                 child = c("a", "b", "d", "e", "c", "c", rep("g", 3)),
                 s = c(0.01, 0.02, 0.03, 0.04, 0.1, 0.1, rep(0.2, 3)),
                 sh = c(rep(0, 4), c(-.1, -.2), c(-.05, -.06, -.07)),
                 typeDep = "MN")

try(fc1 <- allFitnessEffects(c1))
```

If you try this, you'll get an error. There is an error because the "sh" varies within a child, and we do not allow that for a poset-type specification, as it is ambiguous. If you need arbitrary fitness values for arbitrary combinations of genotypes, you can specify them using epistatic effects as in section 3.7 and order effects as in section 3.6.

Why do we need to specify as many "s" and "sh" as there are rows (or a single one, that gets expanded to those many) when the "s" and "sh" are properties of the child node, not of the edges? Because, for ease, we use a data.frame.

We fix the error in our specification. Notice that the "sh" is not set to -1 in these examples. If you want strict compliance with the poset restrictions, you should set $sh = -1$ or, better yet, $sh = -\infty$ (see section 3.2.2), but having an $sh > -1$ will lead to fitnesses that are > 0 and, thus, is a way of modeling small deviations from the poset (see discussion in Diaz-Uriarte 2015).

Note that for those nodes that depend only on "Root" the type of dependency is irrelevant.

```
c1 <- data.frame(parent = c(rep("Root", 4), "a", "b", "d", "e", "c"),
                 child = c("a", "b", "d", "e", "c", "c", rep("g", 3)),
                 s = c(0.01, 0.02, 0.03, 0.04, 0.1, 0.1, rep(0.2, 3)),
                 sh = c(rep(0, 4), c(-.9, -.9), rep(-.95, 3)),
                 typeDep = "MN")

cbn2 <- allFitnessEffects(c1)
```

We could get graphical representations but the figures would be the same as in the example in section 3.4.4, since the structure has not changed, only the numeric values.

What is the fitness of all possible genotypes? Here, order of events *per se* does not matter, beyond that considered in the poset. In other words, the fitness of genotype "a, b, c" is the same no matter how we got to "a, b, c". What matters is whether or not the genes on which each of "a", "b", and "c" depend are present or not (I only show the first 10 genotypes)

```r
gcbn2 <- evalAllGenotypes(cbn2, order = FALSE)
gcbn2[1:10, ]
##    Genotype Fitness
## 1         a   1.010
## 2         b   1.020
## 3         c   0.100
## 4         d   1.030
## 5         e   1.040
## 6         g   0.050
## 7      a, b   1.030
## 8      a, c   0.101
## 9      a, d   1.040
## 10     a, e   1.050
```

Of course, if we were to look at genotypes but taking into account order of occurrence of mutations, we would see no differences

```r
gcbn2o <- evalAllGenotypes(cbn2, order = TRUE, max = 1956)
gcbn2o[1:10, ]
##    Genotype Fitness
## 1         a   1.010
## 2         b   1.020
## 3         c   0.100
## 4         d   1.030
## 5         e   1.040
## 6         g   0.050
## 7     a > b   1.030
## 8     a > c   0.101
## 9     a > d   1.040
## 10    a > e   1.050
```

(The $max = 1956$ is there so that we show all the genotypes, even if they are more than 256, the default.)

You can check the output and verify things are as they should. For instance:

```r
all.equal(
        gcbn2[c(1:21, 22, 28, 41, 44, 56, 63 ) , "Fitness"],
        c(1.01, 1.02, 0.1, 1.03, 1.04, 0.05,
          1.01 * c(1.02, 0.1, 1.03, 1.04, 0.05),
          1.02 * c(0.10, 1.03, 1.04, 0.05),
          0.1 * c(1.03, 1.04, 0.05),
          1.03 * c(1.04, 0.05),
          1.04 * 0.05,
          1.01 * 1.02 * 1.1,
          1.01 * 0.1 * 0.05,
          1.03 * 1.04 * 0.05,
          1.01 * 1.02 * 1.1 * 0.05,
```

```
           1.03 * 1.04 * 1.2 * 0.1, ## notice this
           1.01 * 1.02 * 1.03 * 1.04 * 1.1 * 1.2
           ))
## [1] TRUE
```

A particular one that is important to understand is genotype with mutated genes “c, d, e, g”:

```
gcbn2[56, ]
##      Genotype Fitness
## 56 c, d, e, g  0.1285
all.equal(gcbn2[56, "Fitness"], 1.03 * 1.04 * 1.2 * 0.10)
## [1] TRUE
```

where “g” is taken as if its dependencies are satisfied (as “c”, “d“, and”e" are present) even when the dependencies of “c” are not satisfied (and that is why the term for “c” is 0.9).

3.4.6 DAGs: A semimonotone or “OR” example

We will reuse the above example, changing the type of relationship:

```
s1 <- data.frame(parent = c(rep("Root", 4), "a", "b", "d", "e", "c"),
                 child = c("a", "b", "d", "e", "c", "c", rep("g", 3)),
                 s = c(0.01, 0.02, 0.03, 0.04, 0.1, 0.1, rep(0.2, 3)),
                 sh = c(rep(0, 4), c(-.9, -.9), rep(-.95, 3)),
                 typeDep = "SM")

smn1 <- allFitnessEffects(s1)
```

It looks like this (where edges are shown in blue to denote the semimonotone relationship):

```
plot(smn1)
```

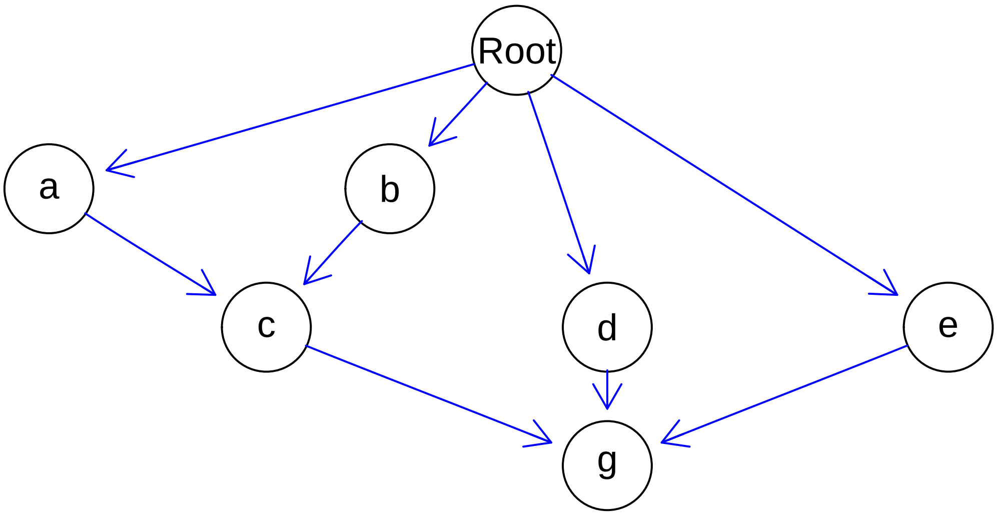


```
gsmn1 <- evalAllGenotypes(smn1, order = FALSE)
```

Having just one parental dependency satisfied is now enough, in contrast to what happened before. For instance:

```
gcbn2[c(8, 12, 22), ]
##    Genotype Fitness
## 8      a, c   0.101
## 12     b, c   0.102
## 22  a, b, c   1.133
gsmn1[c(8, 12, 22), ]
##    Genotype Fitness
## 8      a, c   1.111
## 12     b, c   1.122
## 22  a, b, c   1.133

gcbn2[c(20:21, 28), ]
##    Genotype Fitness
## 20     d, g 0.05150
## 21     e, g 0.05200
## 28  a, c, g 0.00505
gsmn1[c(20:21, 28), ]
##    Genotype Fitness
## 20     d, g   1.236
## 21     e, g   1.248
## 28  a, c, g   1.333
```

3.4.7 An "XMPN" or "XOR" example

Again, we reuse the example above, changing the type of relationship:

```
x1 <- data.frame(parent = c(rep("Root", 4), "a", "b", "d", "e", "c"),
```

```r
                  child = c("a", "b", "d", "e", "c", "c", rep("g", 3)),
                  s = c(0.01, 0.02, 0.03, 0.04, 0.1, 0.1, rep(0.2, 3)),
                  sh = c(rep(0, 4), c(-.9, -.9), rep(-.95, 3)),
                  typeDep = "XMPN")

xor1 <- allFitnessEffects(x1)
```

It looks like this (edges in red to denote the "XOR" relationship):

```r
plot(xor1)
```

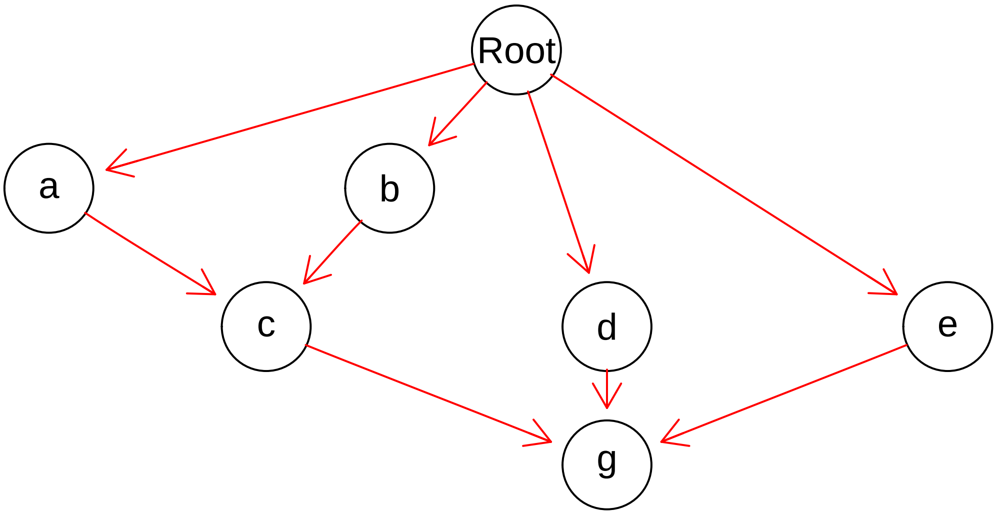


```r
gxor1 <- evalAllGenotypes(xor1, order = FALSE)
```

Whenever "c" is present with both "a" and "b", the fitness component for "c" will be $(1 - 0.1)$. Similarly for "g" (if more than one of "d","e", or"c" is present, it will show as $(1 - 0.05)$). For example:

```r
gxor1[c(22, 41), ]
##    Genotype Fitness
## 22  a, b, c 0.10302
## 41  d, e, g 0.05356
c(1.01 * 1.02 * 0.1, 1.03 * 1.04 * 0.05)
## [1] 0.10302 0.05356
```

However, having just both "a" and "b" is identical to the case with CBN and the monotone relationship (see sections 3.4.5 and 3.4.6). If you want the joint presence of "a" and "b" to result in different fitness than the product of the individual terms, without considering the presence of "c", you can specify that using general epistatic effects (section 3.7).

We also see a very different pattern compared to CBN (section 3.4.5) here:

```r
gxor1[28, ]
##    Genotype Fitness
## 28  a, c, g   1.333
```

```
1.01 * 1.1 * 1.2
## [1] 1.333
```

as exactly one of the dependencies for both "c" and "g" are satisfied.

But

```
gxor1[44, ]
##      Genotype Fitness
## 44 a, b, c, g  0.1236
1.01 * 1.02 * 0.1 * 1.2
## [1] 0.1236
```

is the result of a 0.1 for "c" (and a 1.2 for "g" that has exactly one of its dependencies satisfied).

3.4.8 Posets: the three types of relationships

```
p3 <- data.frame(
    parent = c(rep("Root", 4), "a", "b", "d", "e", "c", "f"),
    child = c("a", "b", "d", "e", "c", "c", "f", "f", "g", "g"),
    s = c(0.01, 0.02, 0.03, 0.04, 0.1, 0.1, 0.2, 0.2, 0.3, 0.3),
    sh = c(rep(0, 4), c(-.9, -.9), c(-.95, -.95), c(-.99, -.99)),
    typeDep = c(rep("--", 4),
                "XMPN", "XMPN", "MN", "MN", "SM", "SM"))
fp3 <- allFitnessEffects(p3)
```

This is how it looks like:

```
plot(fp3)
```

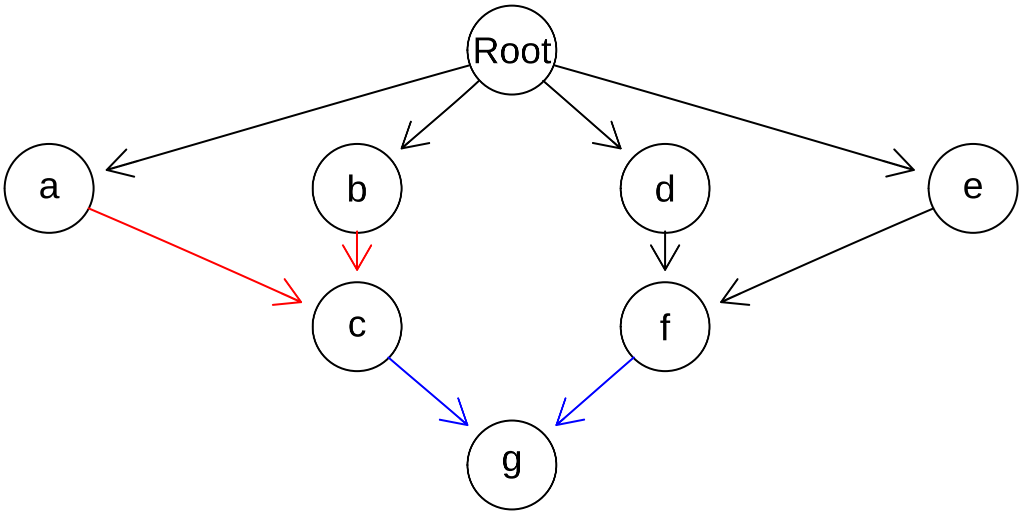

We can also use "igraph":

```r
plot(fp3, "igraph", layout.reingold.tilford)
```

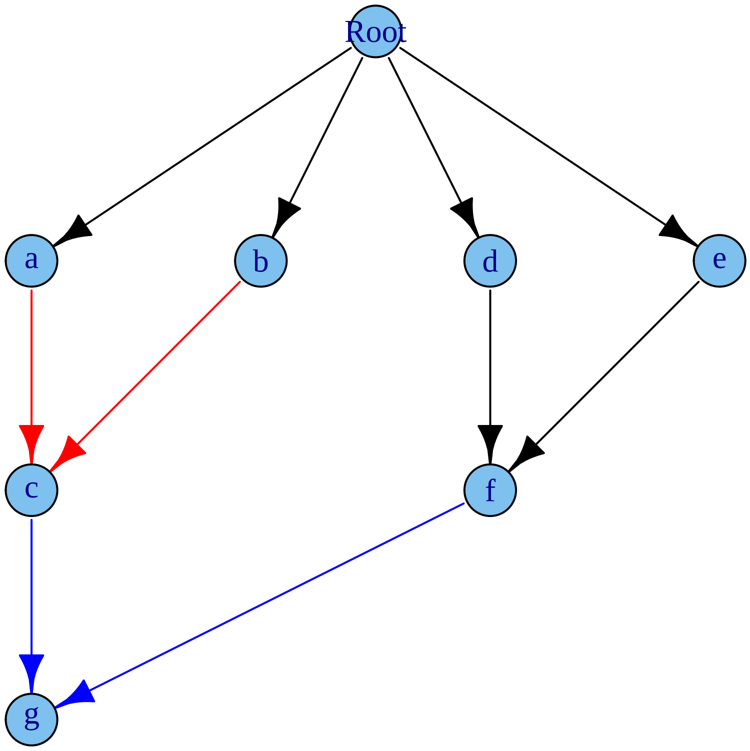


```r
gfp3 <- evalAllGenotypes(fp3, order = FALSE)
```

Let's look at a few:

```r
gfp3[c(9, 24, 29, 59, 60, 66, 119, 120, 126, 127), ]
##                  Genotype  Fitness
## 9                    a, c 1.111000
## 24                   d, f 0.051500
## 29                a, b, c 0.103020
## 59                c, f, g 0.006500
## 60                d, e, f 1.285440
## 66             a, b, c, f 0.005151
## 119         c, d, e, f, g 0.167107
## 120         a, b, c, d, e, f 0.132426
## 126      b, c, d, e, f, g 1.874943
## 127 a, b, c, d, e, f, g 0.172154

c(1.01 * 1.1, 1.03 * .05, 1.01 * 1.02 * 0.1, 0.1 * 0.05 * 1.3,
  1.03 * 1.04 * 1.2, 1.01 * 1.02 * 0.1 * 0.05,
  0.1 * 1.03 * 1.04 * 1.2 * 1.3,
  1.01 * 1.02 * 0.1 * 1.03 * 1.04 * 1.2,
  1.02 * 1.1 * 1.03 * 1.04 * 1.2 * 1.3,
  1.01 * 1.02 * 1.03 * 1.04 * 0.1 * 1.2 * 1.3)
##  [1] 1.111000 0.051500 0.103020 0.006500 1.285440 0.005151 0.167107
```

```
##  [8] 0.132426 1.874943 0.172154
```

As before, looking at the order of mutations makes no difference (look at the test directory to see a test that verifies this assertion).

3.5 Modules

As already mentioned, we can think of all the effects of fitness in terms not of individual genes but, rather, modules. This idea is discussed in, for example, Raphael and Vandin (2015), Gerstung et al. (2011): the restrictions encoded in, say, the DAGs can be considered to apply not to genes, but to modules, where each module is a set of genes (and the intersection between modules is the empty set). Modules, then, play the role of a "union operation" over sets of genes. Of course, if we can use modules for the restrictions in the DAGs we should also be able to use them for epistasis and order effects, as we will see later (e.g., 3.6.2).

3.5.1 What does a module provide

Modules can provide very compact ways of specifying relationships when you want to, well, model the existence of modules. For simplicity suppose there is a module, "A", made of genes "a1" and "a2", and a module "B", made of a single gene "b1". Module "B" can mutate if module "A" is mutated, but mutating both "a1" and "a2" provides no additional fitness advantage compared to mutating only a single one of them. We can specify this as:

```
s <- 0.2
sboth <- (1/(1 + s)) - 1
m0 <- allFitnessEffects(data.frame(
    parent = c("Root", "Root", "a1", "a2"),
    child = c("a1", "a2", "b", "b"),
    s = s,
    sh = -1,
    typeDep = "OR"),
                                epistasis = c("a1:a2" = sboth))
evalAllGenotypes(m0, order = FALSE, addwt = TRUE)
##    Genotype Fitness
## 1        WT    1.00
## 2        a1    1.20
## 3        a2    1.20
## 4         b    0.00
## 5    a1, a2    1.20
## 6     a1, b    1.44
## 7     a2, b    1.44
## 8 a1, a2, b    1.44
```

Note that we need to add an epistasis term, with value "sboth" to capture the idea of "mutating both"a1" and "a2" provides no additional fitness advantage compared to mutating only a single one of them"; see details in section 3.7.

Now, specify it using modules:

```
s <- 0.2
m1 <- allFitnessEffects(data.frame(
    parent = c("Root", "A"),
    child = c("A", "B"),
    s = s,
    sh = -1,
    typeDep = "OR"),
                        geneToModule = c("Root" = "Root",
                                         "A" = "a1, a2",
                                         "B" = "b1"))
evalAllGenotypes(m1, order = FALSE, addwt = TRUE)
##     Genotype Fitness
## 1         WT    1.00
## 2         a1    1.20
## 3         a2    1.20
## 4         b1    0.00
## 5     a1, a2    1.20
## 6     a1, b1    1.44
## 7     a2, b1    1.44
## 8 a1, a2, b1    1.44
```

This captures the ideas directly. The typing savings here are small, but they can be large with modules with many genes.

3.5.2 Specifying modules

How do you specify modules? The general procedure is simple: you pass a vector that makes explicit the mapping from modules to sets of genes. We just saw an example. There are several additional examples such as 3.5.3, 3.6.2, 3.7.4.

It is important to note that, once you specify modules, we expect all of the relationships (except those that involve the non interacting genes) to be specified as modules. Thus, all elements of the epistasis, posets (the DAGs) and order effects components should be specified in terms of modules. But you can, of course, specify a module as containing a single gene (and a single gene with the same name as the module).

What about the "Root" node? If you use a "restriction table", that restriction table (that DAG) must have a node named "Root" and in the mapping of genes to module there **must** be a first entry that has a module and gene named "Root", as we saw above with `geneToModule = c("Root" = "Root", ...`. We force you to do this to be explicit about the "Root" node. This is not needed (thought it does not hurt) with other fitness specifications. For instance, if we have a model with two modules,

one of them with two genes (see details in section 3.8) we do not need to pass a “Root” as in

```
fnme <- allFitnessEffects(epistasis = c("A" = 0.1,
                                        "B" = 0.2),
                          geneToModule = c("A" = "a1, a2",
                                           "B" = "b1"))
evalAllGenotypes(fnme, order = FALSE, addwt = TRUE)
##     Genotype Fitness
## 1         WT    1.00
## 2         a1    1.10
## 3         a2    1.10
## 4         b1    1.20
## 5     a1, a2    1.10
## 6     a1, b1    1.32
## 7     a2, b1    1.32
## 8 a1, a2, b1    1.32
```

but it is also OK to have a “Root” in the `geneToModule`:

```
fnme2 <- allFitnessEffects(epistasis = c("A" = 0.1,
                                         "B" = 0.2),
                           geneToModule = c(
                               "Root" = "Root",
                               "A" = "a1, a2",
                               "B" = "b1"))
evalAllGenotypes(fnme, order = FALSE, addwt = TRUE)
##     Genotype Fitness
## 1         WT    1.00
## 2         a1    1.10
## 3         a2    1.10
## 4         b1    1.20
## 5     a1, a2    1.10
## 6     a1, b1    1.32
## 7     a2, b1    1.32
## 8 a1, a2, b1    1.32
```

3.5.3 Modules and posets again: the three types of relationships and modules

We use the same specification of poset, but add modules. To keep it manageable, we only add a few genes for some modules, and have some modules with a single gene. Beware that the number of genotypes is starting to grow quite fast, though. We capitalize to differentiate modules (capital letters) from genes (lowercase with a number), but this is not needed.

```r
p4 <- data.frame(
    parent = c(rep("Root", 4), "A", "B", "D", "E", "C", "F"),
    child = c("A", "B", "D", "E", "C", "C", "F", "F", "G", "G"),
    s = c(0.01, 0.02, 0.03, 0.04, 0.1, 0.1, 0.2, 0.2, 0.3, 0.3),
    sh = c(rep(0, 4), c(-.9, -.9), c(-.95, -.95), c(-.99, -.99)),
    typeDep = c(rep("--", 4),
                "XMPN", "XMPN", "MN", "MN", "SM", "SM"))

fp4m <- allFitnessEffects(
    p4,
    geneToModule = c("Root" = "Root", "A" = "a1",
                     "B" = "b1, b2", "C" = "c1",
                     "D" = "d1, d2", "E" = "e1",
                     "F" = "f1, f2", "G" = "g1"))
```

By default, plotting shows the modules:

```r
plot(fp4m)
```

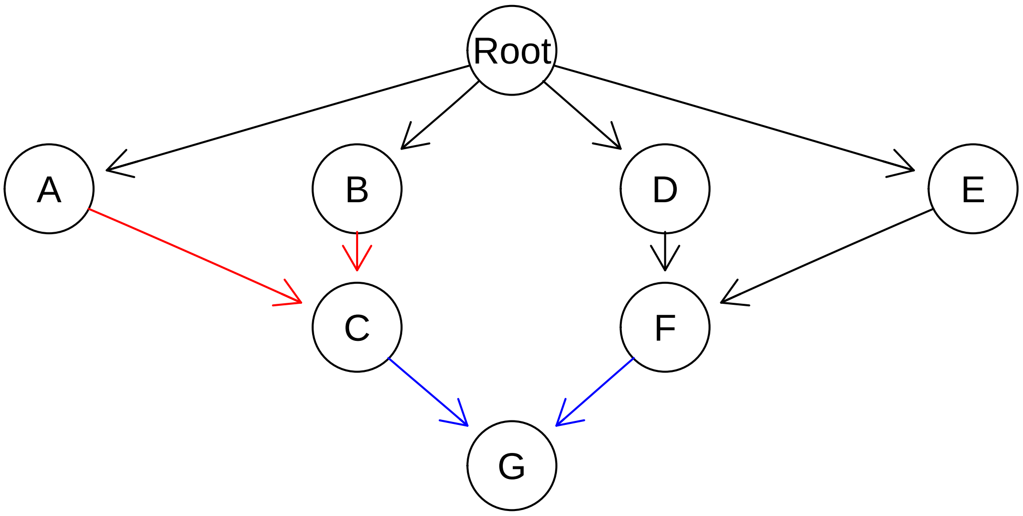

but we can show the gene names instead of the module names:

```r
plot(fp4m, expandModules = TRUE)
```

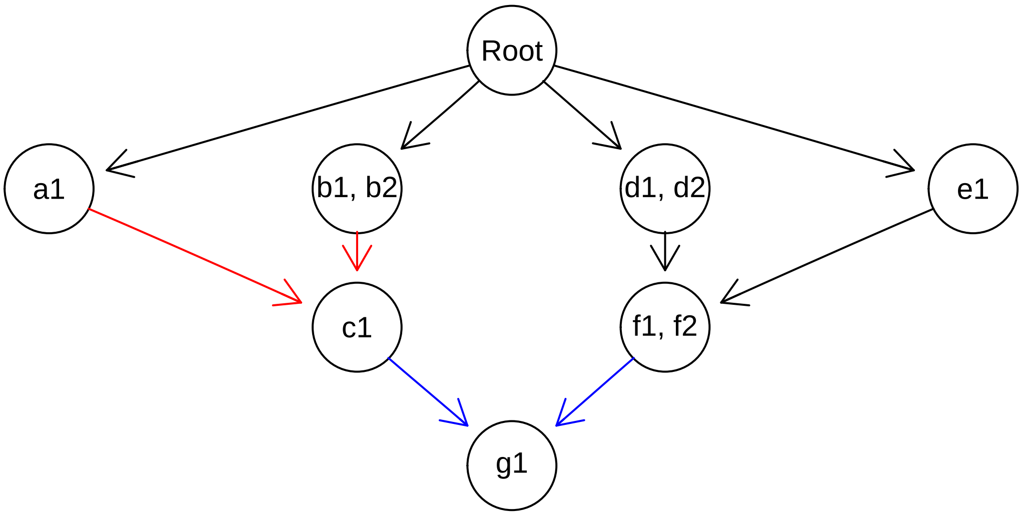

or

```
plot(fp4m, "igraph", layout = layout.reingold.tilford,
     expandModules = TRUE)
```

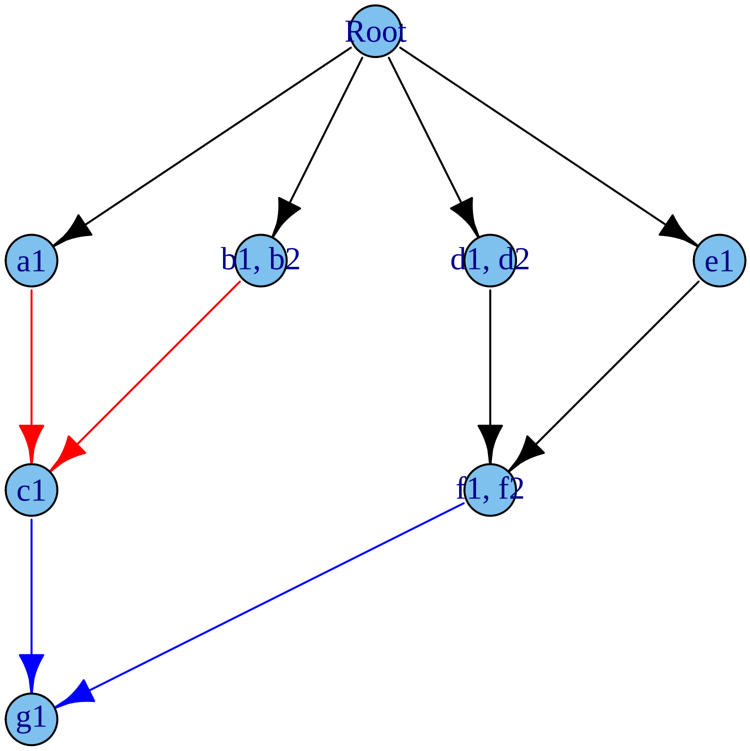

We obtain the fitness of all genotypes in the usual way:

```
gfp4 <- evalAllGenotypes(fp4m, order = FALSE, max = 1024)
```

Let's look at a few of those:

```
gfp4[c(12, 20, 21, 40, 41, 46, 50, 55, 64, 92,
       155, 157, 163, 372, 632, 828), ]
##                    Genotype Fitness
```

```
## 12                    a1, b2  1.0302
## 20                    b1, b2  1.0200
## 21                    b1, c1  1.1220
## 40                    c1, g1  0.1300
## 41                    d1, d2  1.0300
## 46                    d2, e1  1.0712
## 50                    e1, f1  0.0520
## 55                    f2, g1  0.0650
## 64                a1, b2, c1  0.1030
## 92                b1, b2, c1  1.1220
## 155               c1, f2, g1  0.0065
## 157               d1, d2, f1  0.0515
## 163               d1, f1, f2  0.0515
## 372           d1, d2, e1, f2  1.2854
## 632       d1, d2, e1, f1, f2  1.2854
## 828 b2, c1, d1, e1, f2, g1  1.8749

c(1.01 * 1.02, 1.02, 1.02 * 1.1, 0.1 * 1.3, 1.03,
  1.03 * 1.04, 1.04 * 0.05, 0.05 * 1.3,
  1.01 * 1.02 * 0.1, 1.02 * 1.1, 0.1 * 0.05 * 1.3,
  1.03 * 0.05, 1.03 * 0.05, 1.03 * 1.04 * 1.2, 1.03 * 1.04 * 1.2,
  1.02 * 1.1 * 1.03 * 1.04 * 1.2 * 1.3)
##  [1] 1.0302 1.0200 1.1220 0.1300 1.0300 1.0712 0.0520 0.0650 0.1030
## [10] 1.1220 0.0065 0.0515 0.0515 1.2854 1.2854 1.8749
```

3.6 Order effects

As explained in the introduction (section 1), by order effects we mean a phenomenon such as the one shown empirically by Ortmann et al. (2015): the fitness of a double mutant "A", "B" is different depending on whether "A" was acquired before "B" or "B" before "A". This, of course, can be generalized to more than two genes.

Note that order effects are different from the restrictions in the order of accumulation of mutations discussed in section 3.4. With restrictions in the order of accumulation of mutations we might say that acquiring "B" depends or is facilitated by having "A" mutated (and, unless we allowed for multiple mutations, having "A" mutated means having "A" mutated before "B"). However, once you have the genotype "A, B", its fitness does not depend on the order in which "A" and "B" appeared.

3.6.1 Order effects: three-gene orders

Consider this case, where three specific three-gene orders and two two-gene orders (one of them a subset of one of the three) lead to different fitness compared to the wild-type. We add also modules, to show its usage (but just limit ourselves to using one gene per module here).

Order effects are specified using a $x > y$, which means that that order effect is satisfied when module x is mutated before module y.

```
o3 <- allFitnessEffects(orderEffects = c(
                             "F > D > M" = -0.3,
                             "D > F > M" = 0.4,
                             "D > M > F" = 0.2,
                             "D > M"     = 0.1,
                             "M > D"     = 0.5),
                         geneToModule =
                             c("M" = "m",
                               "F" = "f",
                               "D" = "d") )


(ag <- evalAllGenotypes(o3, addwt = TRUE, order = TRUE))
##     Genotype Fitness
## 1         WT    1.00
## 2          d    1.00
## 3          f    1.00
## 4          m    1.00
## 5      d > f    1.00
## 6      d > m    1.10
## 7      f > d    1.00
## 8      f > m    1.00
## 9      m > d    1.50
## 10     m > f    1.00
## 11 d > f > m    1.54
## 12 d > m > f    1.32
## 13 f > d > m    0.77
## 14 f > m > d    1.50
## 15 m > d > f    1.50
## 16 m > f > d    1.50
```

(The meaning of the notation in the output table is as follows: “WT” denotes the wild-type, or non-mutated clone. The notation $x > y$ means that a mutation in “x” happened before a mutation in “y”. A genotype $x > y \_ z$ means that a mutation in “x” happened before a mutation in “y”; there is also a mutation in “z”, but that is a gene for which order does not matter).

The values for the first nine genotypes come directly from the fitness specifications. The 10th genotype matches $D > F > M$ ($= (1 + 0.4)$) but also $D > M$ ($(1 + 0.1)$). The 11th matches $D > M > F$ and $D > M$. The 12th matches $F > D > M$ but also $D > M$. Etc.

3.6.2 Order effects and modules with multiple genes

Consider the following case:

```
ofe1 <- allFitnessEffects(
    orderEffects = c("F > D" = -0.3, "D > F" = 0.4),
    geneToModule =
        c("F" = "f1, f2",
          "D" = "d1, d2") )

ag <- evalAllGenotypes(ofe1, order = TRUE)
```

There are four genes, $d1, d2, f1, f2$, where each d belongs to module D and each f belongs to module F.

What to expect for cases such as $d1 > f1$ or $f1 > d1$ is clear, as shown in

```
ag[5:16,]
##    Genotype Fitness
## 5   d1 > d2     1.0
## 6   d1 > f1     1.4
## 7   d1 > f2     1.4
## 8   d2 > d1     1.0
## 9   d2 > f1     1.4
## 10  d2 > f2     1.4
## 11  f1 > d1     0.7
## 12  f1 > d2     0.7
## 13  f1 > f2     1.0
## 14  f2 > d1     0.7
## 15  f2 > d2     0.7
## 16  f2 > f1     1.0
```

Likewise, cases such as $d1 > d2 > f1$ or $f2 > f1 > d1$ are clear, because in terms of modules they map to $ D > F$ or $F > D$: the observed order of mutation $d1 > d2 > f1$ means that module D was mutated first and module F was mutated second. Similar for $d1 > f1 > f2$ or $f1 > d1 > d2$: those map to $D > F$ and $F > D$. We can see the fitness of those four case in:

```
ag[c(17, 39, 19, 29), ]
##         Genotype Fitness
## 17 d1 > d2 > f1     1.4
## 39 f2 > f1 > d1     0.7
## 19 d1 > f1 > d2     1.4
## 29 f1 > d1 > d2     0.7
```

and they correspond to the values of those order effects, where $F > D = (1 - 0.3)$ and $D > F = (1 + 0.4)$:

```r
ag[c(17, 39, 19, 29), "Fitness"] == c(1.4, 0.7, 1.4, 0.7)
## [1] TRUE TRUE TRUE TRUE
```

What if we match several patterns? For example, $d1 > f1 > d2 > f2$ and $d1 > f1 > f2 > d2$? The first maps to $D > F > D > F$ and the second to $D > F > D$. But since we are concerned with which one happened first and which happened second we should expect those two to correspond to the same fitness, that of pattern $D > F$, as is the case:

```r
ag[c(43, 44),]
##              Genotype Fitness
## 43 d1 > f1 > d2 > f2     1.4
## 44 d1 > f1 > f2 > d2     1.4
ag[c(43, 44), "Fitness"] == c(1.4, 1.4)
## [1] TRUE TRUE
```

More generally, that applies to all the patterns that start with one of the "d" genes:

```r
all(ag[41:52, "Fitness"] == 1.4)
## [1] TRUE
```

Similar arguments apply to the opposite pattern, $F > D$, which apply to all the possible gene mutation orders that start with one of the "f" genes. For example:

```r
all(ag[53:64, "Fitness"] == 0.7)
## [1] TRUE
```

3.6.3 Order and modules with 325 genotypes

We can of course have more than two genes per module. This just repeats the above, with five genes (there are 325 genotypes, and that is why we pass the "max" argument to `evalAllGenotypes`, to allow for more than the default 256).

```r
ofe2 <- allFitnessEffects(
    orderEffects = c("F > D" = -0.3, "D > F" = 0.4),
    geneToModule =
        c("F" = "f1, f2, f3",
          "D" = "d1, d2") )
ag2 <- evalAllGenotypes(ofe2, max = 325, order = TRUE)
```

We can verify that any combination that starts with a "d" gene and then contains at least one "f" gene will have a fitness of $1 + 0.4$. And any combination that starts with an "f" gene and contains at least one "d" genes will have a fitness of $1 - 0.3$. All other genotypes have a fitness of 1:

```r
all(ag2[grep("^d.*f.*", ag2[, 1]), "Fitness"] == 1.4)
## [1] TRUE
all(ag2[grep("^f.*d.*", ag2[, 1]), "Fitness"] == 0.7)
```

```
## [1] TRUE
oe <- c(grep("^f.*d.*", ag2[, 1]), grep("^d.*f.*", ag2[, 1]))
all(ag2[-oe, "Fitness"] == 1)
## [1] TRUE
```

3.6.4 Order effects and genes without interactions

We will now look at both order effects and interactions. To make things more interesting, we name genes so that the ordered names do split nicely between those with and those without order effects (this, thus, also serves as a test of messy orders of names).

```
foi1 <- allFitnessEffects(
    orderEffects = c("D>B" = -0.2, "B > D" = 0.3),
    noIntGenes = c("A" = 0.05, "C" = -.2, "E" = .1))
```

You can get a verbose view of what the gene names and modules are (and their automatically created numeric codes) by:

```
foi1[c("geneModule", "long.geneNoInt")]
## $geneModule
##   Gene Module GeneNumID ModuleNumID
## 1 Root   Root         0           0
## 2    B      B         1           1
## 3    D      D         2           2
##
## $long.geneNoInt
##   Gene GeneNumID     s
## A    A         3  0.05
## C    C         4 -0.20
## E    E         5  0.10
```

We can get the fitness of all genotypes (we set $max = 325$ because that is the number of possible genotypes):

```
agoi1 <- evalAllGenotypes(foi1,  max = 325, order = TRUE)
head(agoi1)
##   Genotype Fitness
## 1        B    1.00
## 2        D    1.00
## 3        A    1.05
## 4        C    0.80
## 5        E    1.10
## 6    B > D    1.30
```

Now:

```r
rn <- 1:nrow(agoi1)
names(rn) <- agoi1[, 1]

agoi1[rn[LETTERS[1:5]], "Fitness"] == c(1.05, 1, 0.8, 1, 1.1)
## [1] TRUE TRUE TRUE TRUE TRUE
```

According to the fitness effects we have specified, we also know that any genotype with only two mutations, one of which is either "A", "C" "E" and the other is "B" or "D" will have the fitness corresponding to "A", "C" or "E", respectively:

```r
agoi1[grep("^A > [BD]$", names(rn)), "Fitness"] == 1.05
## [1] TRUE TRUE
agoi1[grep("^C > [BD]$", names(rn)), "Fitness"] == 0.8
## [1] TRUE TRUE
agoi1[grep("^E > [BD]$", names(rn)), "Fitness"] == 1.1
## [1] TRUE TRUE
agoi1[grep("^[BD] > A$", names(rn)), "Fitness"] == 1.05
## [1] TRUE TRUE
agoi1[grep("^[BD] > C$", names(rn)), "Fitness"] == 0.8
## [1] TRUE TRUE
agoi1[grep("^[BD] > E$", names(rn)), "Fitness"] == 1.1
## [1] TRUE TRUE
```

We will not be playing many additional games with regular expressions, but let us check those that start with "D" and have all the other mutations, which occupy rows 230 to 253; fitness should be equal (within numerical error, because of floating point arithmetic) to the order effect of "D" before "B" times the other effects $(1 - 0.3) * 1.05 * 0.8 * 1.1 = 0.7392$

```r
all.equal(agoi1[230:253, "Fitness"] ,
          rep((1 - 0.2) * 1.05 * 0.8 * 1.1, 24))
## [1] TRUE
```

and that will also be the value of any genotype with the five mutations where "D" comes before "B" such as those in rows 260 to 265, 277, or 322 and 323, but it will be equal to $(1 + 0.3) * 1.05 * 0.8 * 1.1 = 1.2012$ in those where "B" comes before "D". Analogous arguments apply to four, three, and two mutation genotypes.

3.7 Epistasis

3.7.1 Epistasis: two alternative specifications

We want the following mapping of genotypes to fitness:

A	B	Fitness
wt	wt	1
wt	M	$1 + s_b$

A	B	Fitness
M	wt	$1 + s_a$
M	M	$1 + s_{ab}$

Suppose that the actual numerical values are $s_a = 0.2, s_b = 0.3, s_{ab} = 0.7$.

We specify the above as follows:

```
sa <- 0.2
sb <- 0.3
sab <- 0.7

e2 <- allFitnessEffects(epistasis =
                            c("A: -B" = sa,
                              "-A:B" = sb,
                              "A : B" = sab))
evalAllGenotypes(e2, order = FALSE, addwt = TRUE)
##   Genotype Fitness
## 1       WT     1.0
## 2        A     1.2
## 3        B     1.3
## 4     A, B     1.7
```

That uses the "-" specification, so we explicitly exclude some patterns: with "A:-B" we say "A when there is no B".

But we can also use a specification where we do not use the "-". That requires a different numerical value of the interaction, because now, as we are rewriting the interaction term as genotype "A is mutant, B is mutant" the double mutant will incorporate the effects of "A mutant", "B mutant" and "both A and B mutants". We can define a new s_2 that satisfies $(1 + s_{ab}) = (1 + s_a)(1 + s_b)(1 + s_2)$ so $(1 + s_2) = (1 + s_{ab})/((1 + s_a)(1 + s_b))$ and therefore specify as:

```
s2 <- ((1 + sab)/((1 + sa) * (1 + sb))) - 1

e3 <- allFitnessEffects(epistasis =
                            c("A" = sa,
                              "B" = sb,
                              "A : B" = s2))
evalAllGenotypes(e3, order = FALSE, addwt = TRUE)
##   Genotype Fitness
## 1       WT     1.0
## 2        A     1.2
## 3        B     1.3
## 4     A, B     1.7
```

Note that this is the way you would specify effects with FFPopsim (Zanini and Neher 2012). Whether this specification or the previous one with "-" is simpler will depend

on the model. For synthetic mortality and viability, I think the one using "-" is simpler to map genotype tables to fitness effects. See also section 3.7.2 and 3.7.3 and the example in section 5.4.2.

Finally, note that we can also specify some of these effects by combining the graph and the epistasis, as shown in section 5.2.1 or 5.4.2.

3.7.2 Epistasis with three genes and two alternative specifications

Suppose we have

A	B	C	Fitness
M	wt	wt	$1 + s_a$
wt	M	wt	$1 + s_b$
wt	wt	M	$1 + s_c$
M	M	wt	$1 + s_{ab}$
wt	M	M	$1 + s_{bc}$
M	wt	M	$(1 + s_a)(1 + s_c)$
M	M	M	$1 + s_{abc}$

where missing rows have a fitness of 1 (they have been deleted for conciseness). Note that the mutant for exactly A and C has a fitness that is the product of the individual terms (so there is no epistasis in that case).

```
sa <- 0.1
sb <- 0.15
sc <- 0.2
sab <- 0.3
sbc <- -0.25
sabc <- 0.4

sac <- (1 + sa) * (1 + sc) - 1

E3A <- allFitnessEffects(epistasis =
                             c("A:-B:-C" = sa,
                               "-A:B:-C" = sb,
                               "-A:-B:C" = sc,
                               "A:B:-C" = sab,
                               "-A:B:C" = sbc,
                               "A:-B:C" = sac,
                               "A : B : C" = sabc)
                                                )

evalAllGenotypes(E3A, order = FALSE, addwt = FALSE)
##   Genotype Fitness
```

```
## 1         A    1.10
## 2         B    1.15
## 3         C    1.20
## 4      A, B    1.30
## 5      A, C    1.32
## 6      B, C    0.75
## 7   A, B, C    1.40
```

We needed to pass the s_{ac} coefficient explicitly, even if it that term was just the product. We can try to avoid using the “-”, however (but we will need to do other calculations). For simplicity, I use capital “S" in what follows where the letters differ from the previous specification:

```
sa <- 0.1
sb <- 0.15
sc <- 0.2
sab <- 0.3
Sab <- ( (1 + sab)/((1 + sa) * (1 + sb))) - 1
Sbc <- ( (1 + sbc)/((1 + sb) * (1 + sc))) - 1
Sabc <- ( (1 + sabc)/
          ( (1 + sa) * (1 + sb) * (1 + sc) *
            (1 + Sab) * (1 + Sbc) ) ) - 1

E3B <- allFitnessEffects(epistasis =
                             c("A" = sa,
                               "B" = sb,
                               "C" = sc,
                               "A:B" = Sab,
                               "B:C" = Sbc,
                               ## "A:C" = sac, ## not needed now
                               "A : B : C" = Sabc)
                                             )
evalAllGenotypes(E3B, order = FALSE, addwt = FALSE)
##   Genotype Fitness
## 1         A    1.10
## 2         B    1.15
## 3         C    1.20
## 4      A, B    1.30
## 5      A, C    1.32
## 6      B, C    0.75
## 7   A, B, C    1.40
```

The above two are, of course, identical:

```
all(evalAllGenotypes(E3A, order = FALSE, addwt = FALSE) ==
    evalAllGenotypes(E3B, order = FALSE, addwt = FALSE))
## [1] TRUE
```

We avoid specifying the "A:C", as it just follows from the individual "A" and "C" terms, but given a specified genotype table, we need to do a little bit of addition and multiplication to get the coefficients.

3.7.3 Why can we specify some effects with a "-"?

Let's suppose we want to specify the synthetic viability example seen before:

A	B	Fitness
wt	wt	1
wt	M	0
M	wt	0
M	M	(1 + s)

where "wt" denotes wild type and "M" denotes mutant.

If you want to directly map the above table to the fitness table for the program, to specify the genotype "A is wt, B is a mutant" you can specify it as "-A,B", not just as "B". Why? Because just the presence of a "B" is also compatible with genotype "A is mutant and B is mutant". If you use "-" you are explicitly saying what should not be there so that "-A,B" is NOT compatible with "A, B". Otherwise, you need to carefully add coefficients. Depending on what you are trying to model, different specifications might be simpler. See the examples in section 3.7.1 and 3.7.2. You have both options.

3.7.4 Epistasis: modules

There is nothing conceptually new, but we will show an example here:

```
sa <- 0.2
sb <- 0.3
sab <- 0.7

em <- allFitnessEffects(epistasis =
                            c("A: -B" = sa,
                              "-A:B" = sb,
                              "A : B" = sab),
                        geneToModule = c("A" = "a1, a2",
                                         "B" = "b1, b2"))
evalAllGenotypes(em, order = FALSE, addwt = TRUE)
##            Genotype Fitness
## 1                WT     1.0
## 2                a1     1.2
## 3                a2     1.2
```

```
## 4              b1      1.3
## 5              b2      1.3
## 6          a1, a2      1.2
## 7          a1, b1      1.7
## 8          a1, b2      1.7
## 9          a2, b1      1.7
## 10         a2, b2      1.7
## 11         b1, b2      1.3
## 12     a1, a2, b1      1.7
## 13     a1, a2, b2      1.7
## 14     a1, b1, b2      1.7
## 15     a2, b1, b2      1.7
## 16 a1, a2, b1, b2      1.7
```

Of course, we can do the same thing without using the "-", as in section 3.7.1:

```
s2 <- ((1 + sab)/((1 + sa) * (1 + sb))) - 1

em2 <- allFitnessEffects(epistasis =
                           c("A" = sa,
                             "B" = sb,
                             "A : B" = s2),
                        geneToModule = c("A" = "a1, a2",
                                         "B" = "b1, b2")
                        )
evalAllGenotypes(em2, order = FALSE, addwt = TRUE)
##          Genotype Fitness
## 1              WT      1.0
## 2              a1      1.2
## 3              a2      1.2
## 4              b1      1.3
## 5              b2      1.3
## 6          a1, a2      1.2
## 7          a1, b1      1.7
## 8          a1, b2      1.7
## 9          a2, b1      1.7
## 10         a2, b2      1.7
## 11         b1, b2      1.3
## 12     a1, a2, b1      1.7
## 13     a1, a2, b2      1.7
## 14     a1, b1, b2      1.7
## 15     a2, b1, b2      1.7
## 16 a1, a2, b1, b2      1.7
```

3.8 I do not want epistasis, but I want modules!

Sometimes you might want something like having several modules, say "A" and "B", each with a number of genes, but with "A" and "B" showing no interaction.

It is a terminological issue whether we should allow `noIntGenes` (no interaction genes), as explained in section 3.3 to actually be modules. The reasoning for not allowing them is that the situation depicted above (several genes in module A, for example) actually is one of interaction: the members of "A" are combined using an "OR" operator (i.e., the fitness consequences of having one or more genes of A mutated are the same), not just simply multiplying their fitness; similarly for "B". This is why no interaction genes also mean no modules allowed.

So how do you get what you want in this case? Enter the names of the modules in the `epistasis` component but have no term for ":" (the colon). Let's see an example:

```
fnme <- allFitnessEffects(epistasis = c("A" = 0.1,
                                        "B" = 0.2),
                          geneToModule = c("A" = "a1, a2",
                                           "B" = "b1, b2, b3"))

evalAllGenotypes(fnme, order = FALSE, addwt = TRUE)
##              Genotype Fitness
## 1                  WT    1.00
## 2                  a1    1.10
## 3                  a2    1.10
## 4                  b1    1.20
## 5                  b2    1.20
## 6                  b3    1.20
## 7              a1, a2    1.10
## 8              a1, b1    1.32
## 9              a1, b2    1.32
## 10             a1, b3    1.32
## 11             a2, b1    1.32
## 12             a2, b2    1.32
## 13             a2, b3    1.32
## 14             b1, b2    1.20
## 15             b1, b3    1.20
## 16             b2, b3    1.20
## 17         a1, a2, b1    1.32
## 18         a1, a2, b2    1.32
## 19         a1, a2, b3    1.32
## 20         a1, b1, b2    1.32
## 21         a1, b1, b3    1.32
## 22         a1, b2, b3    1.32
## 23         a2, b1, b2    1.32
```

```
## 24          a2, b1, b3    1.32
## 25          a2, b2, b3    1.32
## 26          b1, b2, b3    1.20
## 27      a1, a2, b1, b2    1.32
## 28      a1, a2, b1, b3    1.32
## 29      a1, a2, b2, b3    1.32
## 30      a1, b1, b2, b3    1.32
## 31      a2, b1, b2, b3    1.32
## 32 a1, a2, b1, b2, b3    1.32
```

In previous versions these was possible using the longer, still accepted way of specifying a : with a value of 0, but this is no longer needed:

```
fnme <- allFitnessEffects(epistasis = c("A" = 0.1,
                                        "B" = 0.2,
                                        "A : B" = 0.0),
                          geneToModule = c("A" = "a1, a2",
                                           "B" = "b1, b2, b3"))

evalAllGenotypes(fnme, order = FALSE, addwt = TRUE)
##              Genotype Fitness
## 1                  WT    1.00
## 2                  a1    1.10
## 3                  a2    1.10
## 4                  b1    1.20
## 5                  b2    1.20
## 6                  b3    1.20
## 7              a1, a2    1.10
## 8              a1, b1    1.32
## 9              a1, b2    1.32
## 10             a1, b3    1.32
## 11             a2, b1    1.32
## 12             a2, b2    1.32
## 13             a2, b3    1.32
## 14             b1, b2    1.20
## 15             b1, b3    1.20
## 16             b2, b3    1.20
## 17         a1, a2, b1    1.32
## 18         a1, a2, b2    1.32
## 19         a1, a2, b3    1.32
## 20         a1, b1, b2    1.32
## 21         a1, b1, b3    1.32
## 22         a1, b2, b3    1.32
## 23         a2, b1, b2    1.32
## 24         a2, b1, b3    1.32
## 25         a2, b2, b3    1.32
```

```
## 26         b1, b2, b3    1.20
## 27     a1, a2, b1, b2    1.32
## 28     a1, a2, b1, b3    1.32
## 29     a1, a2, b2, b3    1.32
## 30     a1, b1, b2, b3    1.32
## 31     a2, b1, b2, b3    1.32
## 32 a1, a2, b1, b2, b3    1.32
```

This can, of course, be extended to more modules.

3.9 Synthetic viability

Synthetic viability and synthetic lethality (e.g., Ashworth, Lord, and Reis-Filho 2011; Hartman, Garvik, and Hartwell 2001) are just special cases of epistasis (section 3.7) but we deal with them here separately.

3.9.1 A simple synthetic viability example

A simple and extreme example of synthetic viability is shown in the following table, where the joint mutant has fitness larger than the wild type, but each single mutant is lethal.

A	B	Fitness
wt	wt	1
wt	M	0
M	wt	0
M	M	(1 + s)

where “wt” denotes wild type and “M” denotes mutant.

We can specify this (setting $s = 0.2$) as (I play around with spaces, to show there is a certain flexibility with them):

```
s <- 0.2
sv <- allFitnessEffects(epistasis = c("-A : B" = -1,
                                      "A : -B" = -1,
                                      "A:B" = s))
```

Now, let’s look at all the genotypes (we use “addwt” to also get the wt, which by decree has fitness of 1), and disregard order:

```
(asv <- evalAllGenotypes(sv, order = FALSE, addwt = TRUE))
##   Genotype Fitness
## 1       WT     1.0
## 2        A     0.0
```

```
## 3         B     0.0
## 4      A, B     1.2
```

Asking the program to consider the order of mutations of course makes no difference:

```
evalAllGenotypes(sv, order = TRUE, addwt = TRUE)
##   Genotype Fitness
## 1       WT     1.0
## 2        A     0.0
## 3        B     0.0
## 4    A > B     1.2
## 5    B > A     1.2
```

Another example of synthetic viability is shown in section 5.2.2.

Of course, if multiple simultaneous mutations are not possible in the simulations, it is not possible to go from the wildtype to the double mutant in this model where the single mutants are not viable.

3.9.2 Synthetic viability, non-zero fitness, and modules

This is a slightly more elaborate case, where there is one module and the single mutants have different fitness between themselves, which is non-zero. Without the modules, this is the same as in Misra, Szczurek, and Vingron (2014), Figure 1b, which we go over in section 5.2.

A	B	Fitness
wt	wt	1
wt	M	$1 + s_b$
M	wt	$1 + s_a$
M	M	$1 + s_{ab}$

where $s_a, s_b < 0$ but $s_{ab} > 0$.

```
sa <- -0.1
sb <- -0.2
sab <- 0.25
sv2 <- allFitnessEffects(epistasis = c("-A : B" = sb,
                             "A : -B" = sa,
                             "A:B" = sab),
                         geneToModule = c(
                             "A" = "a1, a2",
                             "B" = "b"))
evalAllGenotypes(sv2, order = FALSE, addwt = TRUE)
##    Genotype Fitness
## 1        WT    1.00
```

```
## 2        a1    0.90
## 3        a2    0.90
## 4         b    0.80
## 5    a1, a2    0.90
## 6     a1, b    1.25
## 7     a2, b    1.25
## 8 a1, a2, b    1.25
```

And if we look at order, of course it makes no difference:

```
evalAllGenotypes(sv2, order = TRUE, addwt = TRUE)
##       Genotype Fitness
## 1           WT    1.00
## 2           a1    0.90
## 3           a2    0.90
## 4            b    0.80
## 5      a1 > a2    0.90
## 6       a1 > b    1.25
## 7      a2 > a1    0.90
## 8       a2 > b    1.25
## 9       b > a1    1.25
## 10      b > a2    1.25
## 11 a1 > a2 > b    1.25
## 12 a1 > b > a2    1.25
## 13 a2 > a1 > b    1.25
## 14 a2 > b > a1    1.25
## 15 b > a1 > a2    1.25
## 16 b > a2 > a1    1.25
```

3.10 Synthetic mortality or synthetic lethality

In contrast to section 3.9, here the joint mutant has decreased viability:

A	B	Fitness
wt	wt	1
wt	M	$1 + s_b$
M	wt	$1 + s_a$
M	M	$1 + s_{ab}$

where $s_a, s_b > 0$ but $s_{ab} < 0$.

```
sa <- 0.1
sb <- 0.2
sab <- -0.8
sm1 <- allFitnessEffects(epistasis = c("-A : B" = sb,
```

```
                                    "A : -B" = sa,
                                    "A:B" = sab))
evalAllGenotypes(sm1, order = FALSE, addwt = TRUE)
##   Genotype Fitness
## 1       WT     1.0
## 2        A     1.1
## 3        B     1.2
## 4     A, B     0.2
```

And if we look at order, of course it makes no difference:

```
evalAllGenotypes(sm1, order = TRUE, addwt = TRUE)
##   Genotype Fitness
## 1       WT     1.0
## 2        A     1.1
## 3        B     1.2
## 4    A > B     0.2
## 5    B > A     0.2
```

3.11 Possible issues with Bozic model

3.11.1 Synthetic viability using Bozic model

If we were to use the above specification with Bozic's models, we might not get what we think we should get:

```
evalAllGenotypes(sv, order = FALSE, addwt = TRUE, model = "Bozic")
##   Genotype Death_rate
## 1       WT        1.0
## 2        A        2.0
## 3        B        2.0
## 4     A, B        0.8
```

What gives here? The simulation code would alert you of this (see section 3.11.2) in this particular case because there are "-1", which might indicate that this is not what you want. The problem is that you probably want the Death rate to be infinity (the birth rate was 0, so no clone viability, when we used birth rates —section 3.2.2).

Let us say so explicitly:

```
s <- 0.2
svB <- allFitnessEffects(epistasis = c("-A : B" = -Inf,
                                       "A : -B" = -Inf,
                                       "A:B" = s))
evalAllGenotypes(svB, order = FALSE, addwt = TRUE, model = "Bozic")
##   Genotype Death_rate
## 1       WT        1.0
```

```
## 2         A         Inf
## 3         B         Inf
## 4      A, B         0.8
```

Likewise, values of s larger than one have no effect beyond setting $s = 1$ (a single term of $(1 - 1)$ will drive the product to 0, and as we cannot allow negative death rates negative values are set to 0):

```
s <- 1
svB1 <- allFitnessEffects(epistasis = c("-A : B" = -Inf,
                                        "A : -B" = -Inf,
                                        "A:B" = s))

evalAllGenotypes(svB1, order = FALSE, addwt = TRUE, model = "Bozic")
##   Genotype Death_rate
## 1       WT          1
## 2        A        Inf
## 3        B        Inf
## 4     A, B          0


s <- 3
svB3 <- allFitnessEffects(epistasis = c("-A : B" = -Inf,
                                        "A : -B" = -Inf,
                                        "A:B" = s))

evalAllGenotypes(svB3, order = FALSE, addwt = TRUE, model = "Bozic")
##   Genotype Death_rate
## 1       WT          1
## 2        A        Inf
## 3        B        Inf
## 4     A, B          0
```

Of course, death rates of 0.0 are likely to lead to trouble down the road, when we actually conduct simulations (see section 3.11.2).

3.11.2 Numerical issues with death rates of 0 in Bozic model

As we mentioned above (section 3.11.1) death rates of 0 can lead to trouble when using Bozic's model:

```
i1 <- allFitnessEffects(noIntGenes = c(1, 0.5))
evalAllGenotypes(i1, order = FALSE, addwt = TRUE,
                 model = "Bozic")
##   Genotype Death_rate
## 1       WT        1.0
```

```
## 2        1        0.0
## 3        2        0.5
## 4     1, 2        0.0

i1_b <- oncoSimulIndiv(i1, model = "Bozic")
## Warning in nr_oncoSimul.internal(rFE = fp, birth = birth, death
## = death, : You are using a Bozic model with the new restriction
## specification, and you have at least one s of 1. If that gene is
## mutated, this will lead to a death rate of 0 and the simulations
## will abort when you get a non finite value.
##
##  DEBUG2: Value of rnb = nan
##
##  DEBUG2: Value of m = 1
##
##  DEBUG2: Value of pe = 0
##
##  DEBUG2: Value of pm = 0.999999
##
##  this is spP
##
##  popSize = 1
##  birth = 1
##  death = 0
##  W = 1
##  R = 1
##  mutation = 1e-06
##  timeLastUpdate = 1096.35
##  absfitness = -inf
##  numMutablePos =1
##
##  Unrecoverable exception: Algo 2: retval not finite. Aborting.
```

Of course, there is no problem in using the above with other models:

```
evalAllGenotypes(i1, order = FALSE, addwt = TRUE,
                 model = "Exp")
##   Genotype Fitness
## 1       WT     1.0
## 2        1     2.0
## 3        2     1.5
## 4     1, 2     3.0
i1_e <- oncoSimulIndiv(i1, model = "Exp")
summary(i1_e)
##   NumClones TotalPopSize LargestClone MaxNumDrivers MaxDriversLast
## 1         3    181659523    181588631             0              0
```

```
##   NumDriversLargestPop TotalPresentDrivers FinalTime NumIter
## 1                    0                   0       204     551
##   HittedWallTime HittedMaxTries errorMF minDMratio minBMratio
## 1          FALSE          FALSE      NA      5e+05      5e+05
##   OccurringDrivers
## 1
```

3.12 A longer example: Poset, epistasis, synthetic mortality and viability, order effects and genes without interactions, with some modules

We will now put together a complex example. We will use the poset from section 3.5.3 but will also add:

- Order effects that involve genes in the poset. In this case, if C happens before F, fitness decreases by $1 - 0.1$. If it happens the other way around, there is no effect on fitness beyond their individual contributions.
- Order effects that involve two new modules, "H" and "I" (with genes "h1, h2" and "i1", respectively), so that if H happens before I fitness increases by $1 + 0.12$.
- Synthetic mortality between modules "I" (already present in the epistatic interaction) and "J" (with genes "j1" and "j2"): the joint presence of these modules leads to cell death (fitness of 0).
- Synthetic viability between modules "K" and "M" (with genes "k1", "k2" and "m1", respectively), so that their joint presence is viable but adds nothing to fitness (i.e., mutation of both has fitness 1), whereas each single mutant has a fitness of $1 - 0.5$.
- A set of 5 driver genes ($n1, \ldots, n5$) with fitness that comes from an exponential distribution with rate of 10.

As we are specifying many different things, we will start by writing each set of effects separately:

```
p4 <- data.frame(
    parent = c(rep("Root", 4), "A", "B", "D", "E", "C", "F"),
    child = c("A", "B", "D", "E", "C", "C", "F", "F", "G", "G"),
    s = c(0.01, 0.02, 0.03, 0.04, 0.1, 0.1, 0.2, 0.2, 0.3, 0.3),
    sh = c(rep(0, 4), c(-.9, -.9), c(-.95, -.95), c(-.99, -.99)),
    typeDep = c(rep("--", 4),
                "XMPN", "XMPN", "MN", "MN", "SM", "SM"))

oe <- c("C > F" = -0.1, "H > I" = 0.12)
sm <- c("I:J"  = -1)
sv <- c("-K:M" = -.5, "K:-M" = -.5)
epist <- c(sm, sv)
```

```r
modules <- c("Root" = "Root", "A" = "a1",
             "B" = "b1, b2", "C" = "c1",
             "D" = "d1, d2", "E" = "e1",
             "F" = "f1, f2", "G" = "g1",
             "H" = "h1, h2", "I" = "i1",
             "J" = "j1, j2", "K" = "k1, k2", "M" = "m1")

set.seed(1) ## for reproducibility
noint <- rexp(5, 10)
names(noint) <- paste0("n", 1:5)

fea <- allFitnessEffects(rT = p4, epistasis = epist,
                         orderEffects = oe,
                         noIntGenes = noint,
                         geneToModule = modules)
```

How does it look?

```r
plot(fea)
```

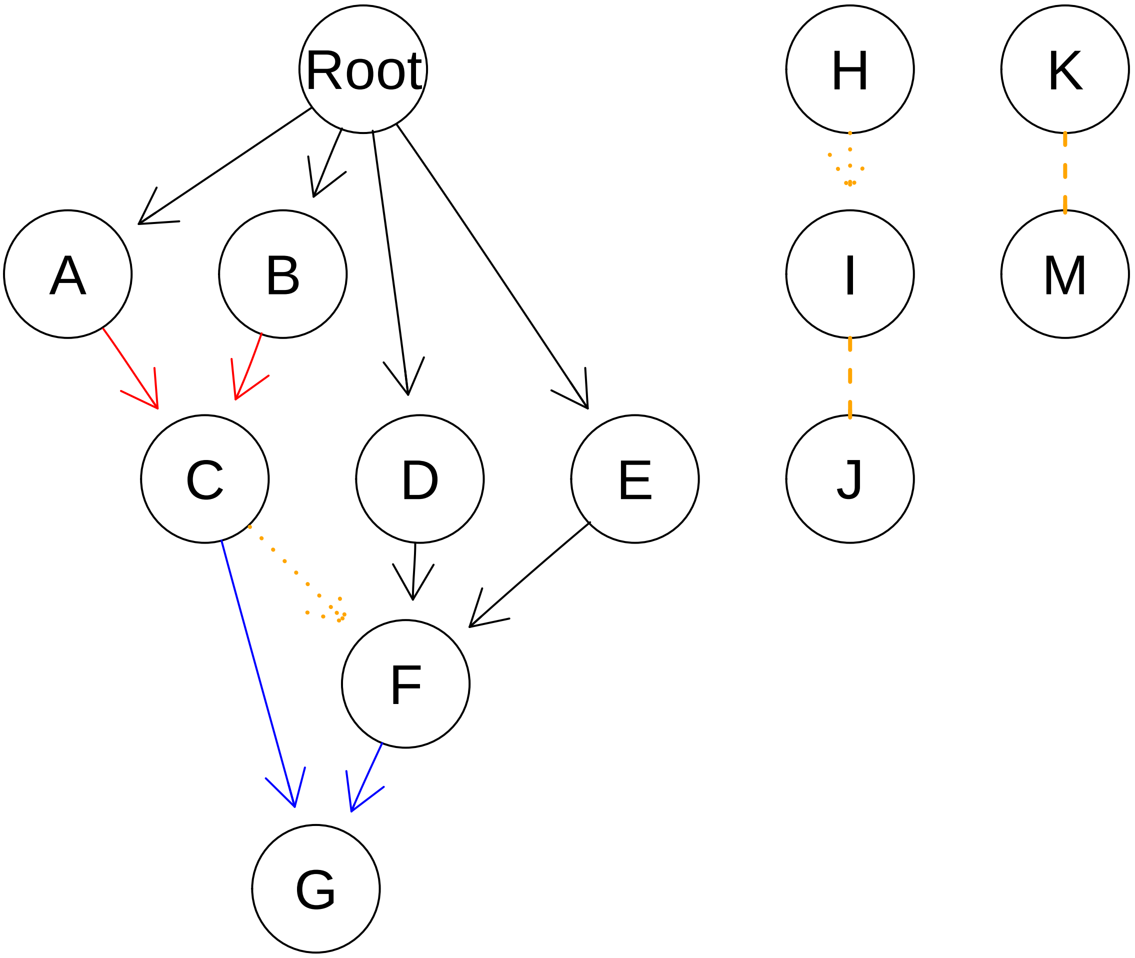

or

```r
plot(fea, "igraph")
```

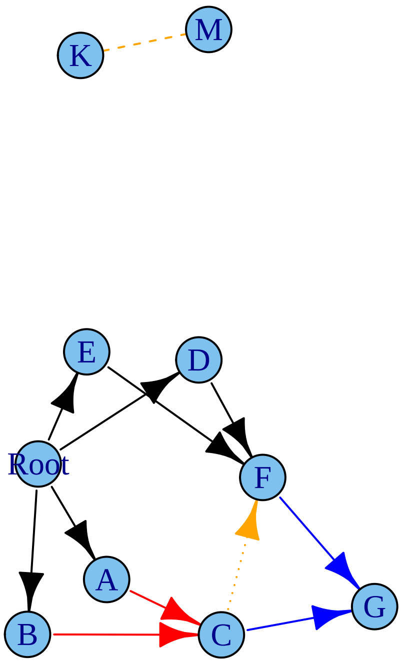

We can, if we want, expand the modules using a "graphNEL" graph

```r
plot(fea, expandModules = TRUE)
```

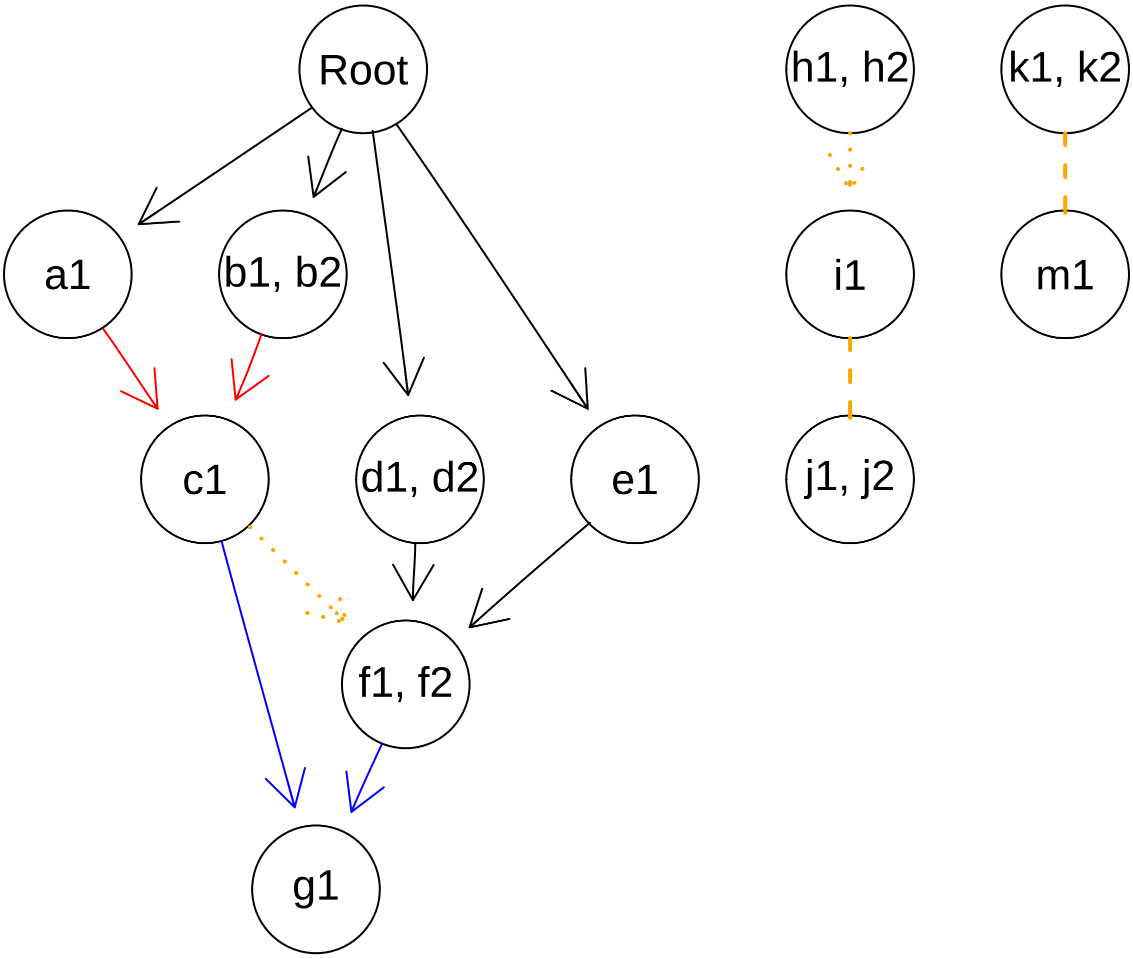

or an “igraph” one

```
plot(fea, "igraph", expandModules = TRUE)
```

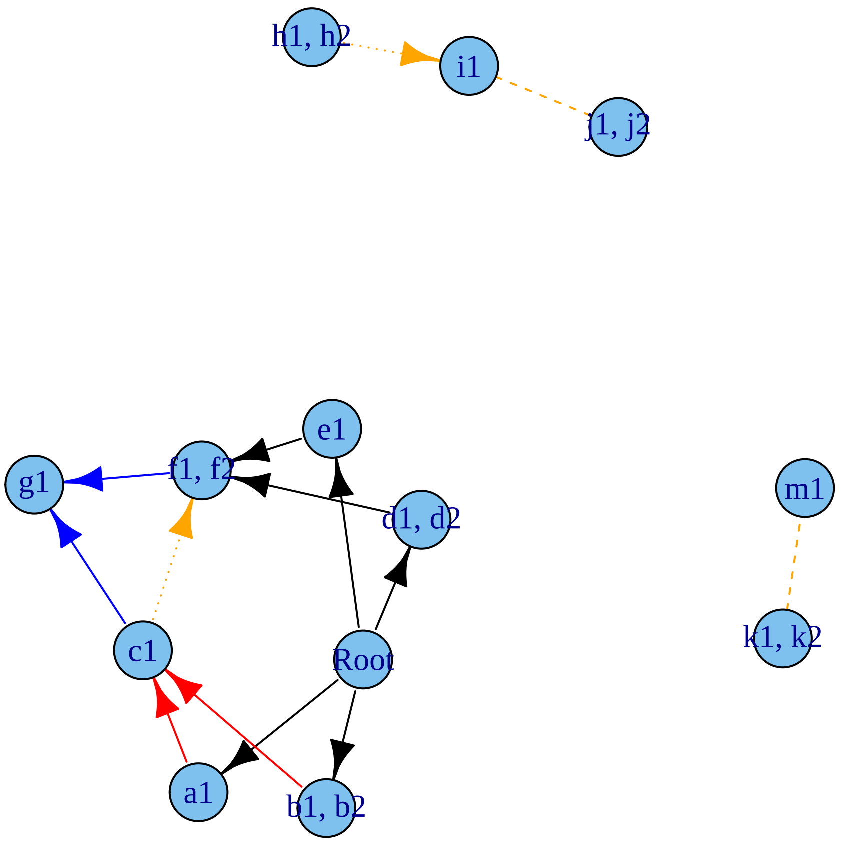

We will not evaluate the fitness of all genotypes, since the number of all ordered genotypes is $> 7 * 10^{22}$. We will look at some specific genotypes:

```
evalGenotype("k1 > i1 > h2", fea) ## 0.5
## [1] 0.5
evalGenotype("k1 > h1 > i1", fea) ## 0.5 * 1.12
## [1] 0.56

evalGenotype("k2 > m1 > h1 > i1", fea) ## 1.12
## [1] 1.12

evalGenotype("k2 > m1 > h1 > i1 > c1 > n3 > f2", fea)
## [1] 0.005113
## 1.12 * 0.1 * (1 + noint[3]) * 0.05 * 0.9
```

Finally, let's generate some ordered genotypes randomly:

```
randomGenotype <- function(fe, ns = NULL) {
    gn <- setdiff(c(fe$geneModule$Gene,
                    fe$long.geneNoInt$Gene), "Root")
    if(is.null(ns)) ns <- sample(length(gn), 1)
    return(paste(sample(gn, ns), collapse = " > "))
}
```

```r
set.seed(2) ## for reproducibility

evalGenotype(randomGenotype(fea), fea, echo = TRUE, verbose = TRUE)
## Genotype:  k2 > i1 > c1 > n1 > m1
##  Individual s terms are : 0.0755182 -0.9
##  Fitness:  0.1076
## [1] 0.1076
## Genotype:  k2 > i1 > c1 > n1 > m1
##  Individual s terms are : 0.0755182 -0.9
##  Fitness:  0.107552
evalGenotype(randomGenotype(fea), fea, echo = TRUE, verbose = TRUE)
## Genotype:  n2 > h1 > h2
##  Individual s terms are : 0.118164
##  Fitness:  1.118
## [1] 1.118
## Genotype:  n2 > h1 > h2
##  Individual s terms are : 0.118164
##  Fitness:  1.11816
evalGenotype(randomGenotype(fea), fea, echo = TRUE, verbose = TRUE)
## Genotype:  d2 > k2 > c1 > f2 > n4 > m1 > n3 > f1 > b1 > g1 > n5 > h1 > j2
##  Individual s terms are : 0.0145707 0.0139795 0.0436069 0.02 0.1 0.03 -0.95 0.3
##  Fitness:  0.07258
## [1] 0.07258
## Genotype:  d2 > k2 > c1 > f2 > n4 > m1 > n3 > f1 > b1 > g1 > n5 > h1 > j2
##  Individual s terms are : 0.0145707 0.0139795 0.0436069 0.02 0.1 0.03 -0.95 0.3
##  Fitness:  0.0725829
evalGenotype(randomGenotype(fea), fea, echo = TRUE, verbose = TRUE)
## Genotype:  h2 > c1 > f1 > n2 > b2 > a1 > n1 > i1
##  Individual s terms are : 0.0755182 0.118164 0.01 0.02 -0.9 -0.95 -0.1 0.12
##  Fitness:  0.006244
## [1] 0.006244
## Genotype:  h2 > c1 > f1 > n2 > b2 > a1 > n1 > i1
##  Individual s terms are : 0.0755182 0.118164 0.01 0.02 -0.9 -0.95 -0.1 0.12
##  Fitness:  0.00624418
evalGenotype(randomGenotype(fea), fea, echo = TRUE, verbose = TRUE)
## Genotype:  h2 > j1 > m1 > d2 > i1 > b2 > k2 > d1 > b1 > n3 > n1 > g1 > h1 > c1 >
##  Individual s terms are : 0.0755182 0.0145707 0.0436069 0.01 0.02 -0.9 0.03 0.04
##  Fitness:  0
## [1] 0
## Genotype:  h2 > j1 > m1 > d2 > i1 > b2 > k2 > d1 > b1 > n3 > n1 > g1 > h1 > c1 >
##  Individual s terms are : 0.0755182 0.0145707 0.0436069 0.01 0.02 -0.9 0.03 0.04
##  Fitness:  0
evalGenotype(randomGenotype(fea), fea, echo = TRUE, verbose = TRUE)
## Genotype:  n1 > m1 > n3 > i1 > j1 > n5 > k1
##  Individual s terms are : 0.0755182 0.0145707 0.0436069 -1
##  Fitness:  0
```

```
## [1] 0
## Genotype:  n1 > m1 > n3 > i1 > j1 > n5 > k1
##  Individual s terms are : 0.0755182 0.0145707 0.0436069 -1
##  Fitness:  0
evalGenotype(randomGenotype(fea), fea, echo = TRUE, verbose = TRUE)
## Genotype:  d2 > n1 > g1 > f1 > f2 > c1 > b1 > d1 > k1 > a1 > b2 > i1 > n4 > h2 >
##  Individual s terms are : 0.0755182 0.118164 0.0139795 0.01 0.02 -0.9 0.03 -0.95
##  Fitness:  0.004205
## [1] 0.004205
## Genotype:  d2 > n1 > g1 > f1 > f2 > c1 > b1 > d1 > k1 > a1 > b2 > i1 > n4 > h2 >
##  Individual s terms are : 0.0755182 0.118164 0.0139795 0.01 0.02 -0.9 0.03 -0.95
##  Fitness:  0.00420528
evalGenotype(randomGenotype(fea), fea, echo = TRUE, verbose = TRUE)
## Genotype:  j1 > f1 > j2 > a1 > n4 > c1 > n3 > k1 > d1 > h1
##  Individual s terms are : 0.0145707 0.0139795 0.01 0.1 0.03 -0.95 -0.5
##  Fitness:  0.02943
## [1] 0.02943
## Genotype:  j1 > f1 > j2 > a1 > n4 > c1 > n3 > k1 > d1 > h1
##  Individual s terms are : 0.0145707 0.0139795 0.01 0.1 0.03 -0.95 -0.5
##  Fitness:  0.0294308
evalGenotype(randomGenotype(fea), fea, echo = TRUE, verbose = TRUE)
## Genotype:  n5 > f2 > f1 > h2 > n4 > c1 > n3 > b1
##  Individual s terms are : 0.0145707 0.0139795 0.0436069 0.02 0.1 -0.95
##  Fitness:  0.06023
## [1] 0.06023
## Genotype:  n5 > f2 > f1 > h2 > n4 > c1 > n3 > b1
##  Individual s terms are : 0.0145707 0.0139795 0.0436069 0.02 0.1 -0.95
##  Fitness:  0.0602298
evalGenotype(randomGenotype(fea), fea, echo = TRUE, verbose = TRUE)
## Genotype:  h1 > d1 > f2
##  Individual s terms are : 0.03 -0.95
##  Fitness:  0.0515
## [1] 0.0515
## Genotype:  h1 > d1 > f2
##  Individual s terms are : 0.03 -0.95
##  Fitness:  0.0515
```

3.13 Homozygosity, heterozygosity, oncogenes, tumor suppressors

We are using what is conceptually a single linear chromosome. However, you can use it to model scenarios where the numbers of copies affected matter, by properly duplicating the genes.

Suppose we have a tumor suppressor gene, G, with two copies, one from Mom and

one from Dad. We can have a table like:

O_M	O_D	Fitness
wt	wt	1
wt	M	1
M	wt	1
M	M	$(1 + s)$

where $s > 0$, meaning that you need two hits, one in each copy, to trigger the clonal expansion.

What about oncogenes? A simple model is that one single hit leads to clonal expansion and additional hits lead to no additional changes, as in this table for gene O, where again the M or D subscript denotes the copy from Mom or from Dad:

O_M	O_D	Fitness
wt	wt	1
wt	M	$(1 + s)$
M	wt	$(1 + s)$
M	M	$(1 + s)$

If you have multiple copies you can proceed similarly. As you can see, these are nothing but special cases of synthetic mortality (3.10), synthetic viability (3.9) and epistasis (3.7).

3.14 Gene-specific mutation rates

You can specify gene-specific mutation rates. Instead of passing a scalar value for `mu`, you pass a named vector. (This does not work with the old v. 1 format, though; yet another reason to stop using that format). This is a simple example (many more are available in the tests, see file `./tests/testthat/test.per-gene-mutation-rates.R`).

```
muvar2 <- c("U" = 1e-6, "z" = 5e-5, "e" = 5e-4, "m" = 5e-3,
            "D" = 1e-4)
ni1 <- rep(0, 5)
names(ni1) <- names(muvar2) ## We use the same names, of course
fe1 <- allFitnessEffects(noIntGenes = ni1)
bb <- oncoSimulIndiv(fe1,
                     mu = muvar2, onlyCancer = FALSE,
                     initSize = 1e5,
                     finalTime = 25,
                     seed =NULL)
```

3.15 Mutator genes

You can specify mutator/antimutator genes (e.g. Gerrish et al. 2007; Tomlinson, Novelli, and Bodmer 1996). These are genes that, when mutated, lead to an increase/decrease in the mutation rate all over the genome (similar to what happens with, say, mutations in mismatch-repair genes or microsatellite instability in cancer).

The specification is very similar to that for fitness effects, except we do not (at least for now) allow the use of DAGs nor of order effects (we have seen no reference in the literature to suggest any of these would be relevant). You can, however, specify epistasis and use modules. Note that the mutator genes must be a subset of the genes in the fitness effects; if you want to have mutator genes that have no direct fitness effects, give them a fitness effect of 0.

This first is a very simple example with simple fitness effects and modules for mutators. We will specify the fitness and mutator effects and evaluate the fitness and mutator effects:

```
fe2 <- allFitnessEffects(noIntGenes =
                         c(a1 = 0.1, a2 = 0.2,
                           b1 = 0.01, b2 = 0.3, b3 = 0.2,
                           c1 = 0.3, c2 = -0.2))

fm2 <- allMutatorEffects(epistasis = c("A" = 5,
                                       "B" = 10,
                                       "C" = 3),
                         geneToModule = c("A" = "a1, a2",
                                          "B" = "b1, b2, b3",
                                          "C" = "c1, c2"))

## Show the fitness effect of a specific genotype
evalGenotype("a1, c2", fe2, verbose = TRUE)
##
##  Individual s terms are : 0.1 -0.2
## [1] 0.88

## Show the mutator effect of a specific genotype
evalGenotypeMut("a1, c2", fm2, verbose = TRUE)
##
##  Individual mutator product terms are : 5 3
## [1] 15

## Fitness and mutator of a specific genotype
evalGenotypeFitAndMut("a1, c2", fe2, fm2, verbose = TRUE)
##
##  Individual s terms are : 0.1 -0.2
##
##  Individual mutator product terms are : 5 3
```

```
## [1]  0.88 15.00
```

You can also use the evalAll functions. We do not show the output here to avoid cluttering the vignette:

```
## Show only all the fitness effects
evalAllGenotypes(fe2, order = FALSE)

## Show only all mutator effects
evalAllGenotypesMut(fm2)

## Show all fitness and mutator
evalAllGenotypesFitAndMut(fe2, fm2, order = FALSE)
```

Building upon the above, the next is an example where we have a bunch of no interaction genes that affect fitness, and a small set of genes that affect the mutation rate (but have no fitness effects).

```
set.seed(1) ## for reproducibility
## 17 genes, 7 with no direct fitness effects
ni <- c(rep(0, 7), runif(10, min = -0.01, max = 0.1))
names(ni) <- c("a1", "a2", "b1", "b2", "b3", "c1", "c2",
               paste0("g", 1:10))

fe3 <- allFitnessEffects(noIntGenes = ni)

fm3 <- allMutatorEffects(epistasis = c("A" = 5,
                                       "B" = 10,
                                       "C" = 3,
                                       "A:C" = 70),
                         geneToModule = c("A" = "a1, a2",
                                          "B" = "b1, b2, b3",
                                          "C" = "c1, c2"))
```

Let us check what the effects are of a few genotypes:

```
## These only affect mutation, not fitness
evalGenotypeFitAndMut("a1, a2", fe3, fm3, verbose = TRUE)
##
##  Individual s terms are : 0 0
##
##  Individual mutator product terms are : 5
## [1] 1 5
evalGenotypeFitAndMut("a1, b3", fe3, fm3, verbose = TRUE)
##
##  Individual s terms are : 0 0
##
##  Individual mutator product terms are : 5 10
```

```
## [1]  1 50

## These only affect fitness: the mutator multiplier is 1
evalGenotypeFitAndMut("g1", fe3, fm3, verbose = TRUE)
##
##  Individual s terms are : 0.019206
## [1] 1.019 1.000
evalGenotypeFitAndMut("g3, g9", fe3, fm3, verbose = TRUE)
##
##  Individual s terms are : 0.0530139 0.0592025
## [1] 1.115 1.000

## These affect both
evalGenotypeFitAndMut("g3, g9, a2, b3", fe3, fm3, verbose = TRUE)
##
##  Individual s terms are : 0 0 0.0530139 0.0592025
##
##  Individual mutator product terms are : 5 10
## [1]  1.115 50.000
```

Finally, we will do a simulation with those data

```
set.seed(1) ## so that it is easy to reproduce
mue1 <- oncoSimulIndiv(fe3, muEF = fm3,
                       mu = 1e-6,
                       initSize = 1e5,
                       model = "McFL",
                       detectionSize = 5e6,
                       finalTime = 500,
                       onlyCancer = FALSE)
```

```
## We do not show this in the vignette to avoid cluttering it
## with output
mue1
```

Of course, it is up to you to keep things reasonable: mutator effects are multiplicative, so if you specify, say, 20 genes (without modules), or 20 modules, each with a mutator effect of 50, the overall mutation rate can be increased by a factor of 50^{20} and that is unlikely to be what you really want (see also section 12.7).

You can play with the following case (an extension of the example above), where a clone with a mutator phenotype and some fitness enhancing mutations starts giving rise to many other clones, some with additional mutator effects, and thus leading to the number of clones blowing up (as some also accumulate additional fitness-enhancing mutations). Things start getting out of hand shortly after time 250. The code below takes a few minutes to run and is not executed here, but you can run it to get an idea of the increase in the number of clones and their relationships (the usage of `plotClonePhylog` is explained in section 8).

```r
set.seed(1) ## for reproducibility
## 17 genes, 7 with no direct fitness effects
ni <- c(rep(0, 7), runif(10, min = -0.01, max = 0.1))
names(ni) <- c("a1", "a2", "b1", "b2", "b3", "c1", "c2",
               paste0("g", 1:10))

## Next is for nicer figure labeling.
## Consider as drivers genes with s >0
gp <- which(ni > 0)

fe3 <- allFitnessEffects(noIntGenes = ni,
                         drvNames = names(ni)[gp])


set.seed(12)
mue1 <- oncoSimulIndiv(fe3, muEF = fm3,
                       mu = 1e-6,
                       initSize = 1e5,
                       model = "McFL",
                       detectionSize = 5e6,
                       finalTime = 270,
                       keepPhylog = TRUE,
                       onlyCancer = FALSE)
mue1
## If you decrease N even further it gets even more cluttered
op <- par(ask = TRUE)
plotClonePhylog(mue1, N = 10, timeEvents = TRUE)
plot(mue1, plotDrivers = TRUE, addtot = TRUE,
     plotDiversity = TRUE)

## The stacked plot is slow; be patient
## Most clones have tiny population sizes, and their lines
## are piled on top of each other
plot(mue1, addtot = TRUE,
     plotDiversity = TRUE, type = "stacked")
par(op)
```

4 Plotting fitness landscapes

The `evalAllGenotypes` and related functions allow you to obtain tables of the genotype to fitness mappings. It might be more convenient to actually plot that, allowing us to quickly identify local minima and maxima and get an idea of how the fitness landscape looks.

In `plotFitnessLandscape` I have blatantly and shamelessly copied most of the looks of the plots of MAGELLAN (Brouillet et al. 2015) (see also http://wwwabi.snv.jussieu.fr/public/Magellan/), a very nice web-based tool for fitness landscape plotting and analysis (MAGELLAN provides some other extra functionality and epistasis statistics not provided here).

As an example, let us show the example of Weissman et al. we saw in 5.4:

```
d1 <- -0.05 ## single mutant fitness 0.95
d2 <- -0.08 ## double mutant fitness 0.92
d3 <- 0.2   ## triple mutant fitness 1.2
s2 <- ((1 + d2)/(1 + d1)^2) - 1
s3 <- ( (1 + d3)/((1 + d1)^3 * (1 + s2)^3) ) - 1

wb <- allFitnessEffects(
    epistasis = c(
        "A" = d1,
        "B" = d1,
        "C" = d1,
        "A:B" = s2,
        "A:C" = s2,
        "B:C" = s2,
        "A:B:C" = s3))
```

```
plotFitnessLandscape(wb, use_ggrepel = TRUE)
```

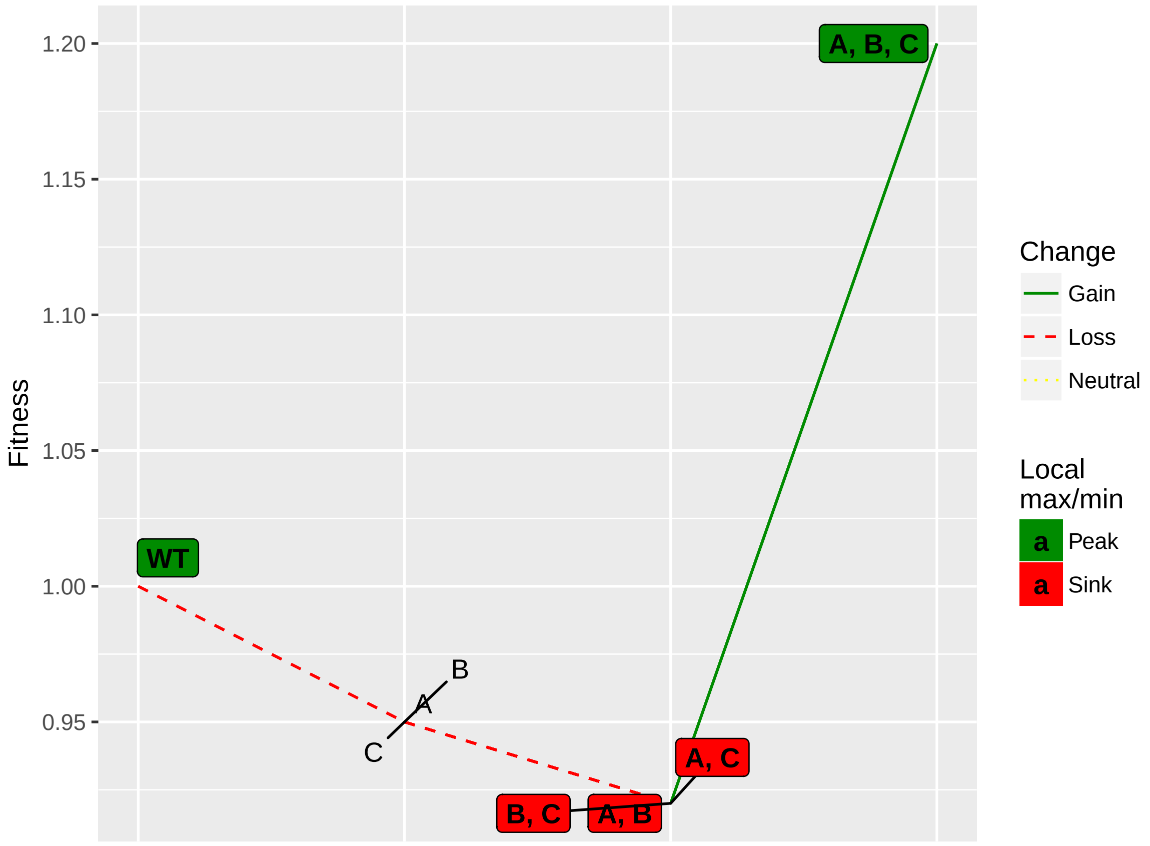

We have set `use_ggrepel = TRUE` to avoid overlap of labels.

For some types of objects, directly invoking plot will give you the fitness landscape plot:

```
(ewb <- evalAllGenotypes(wb, order = FALSE))
##   Genotype Fitness
## 1        A    0.95
## 2        B    0.95
## 3        C    0.95
## 4     A, B    0.92
## 5     A, C    0.92
## 6     B, C    0.92
## 7  A, B, C    1.20
plot(ewb, use_ggrepel = TRUE)
```

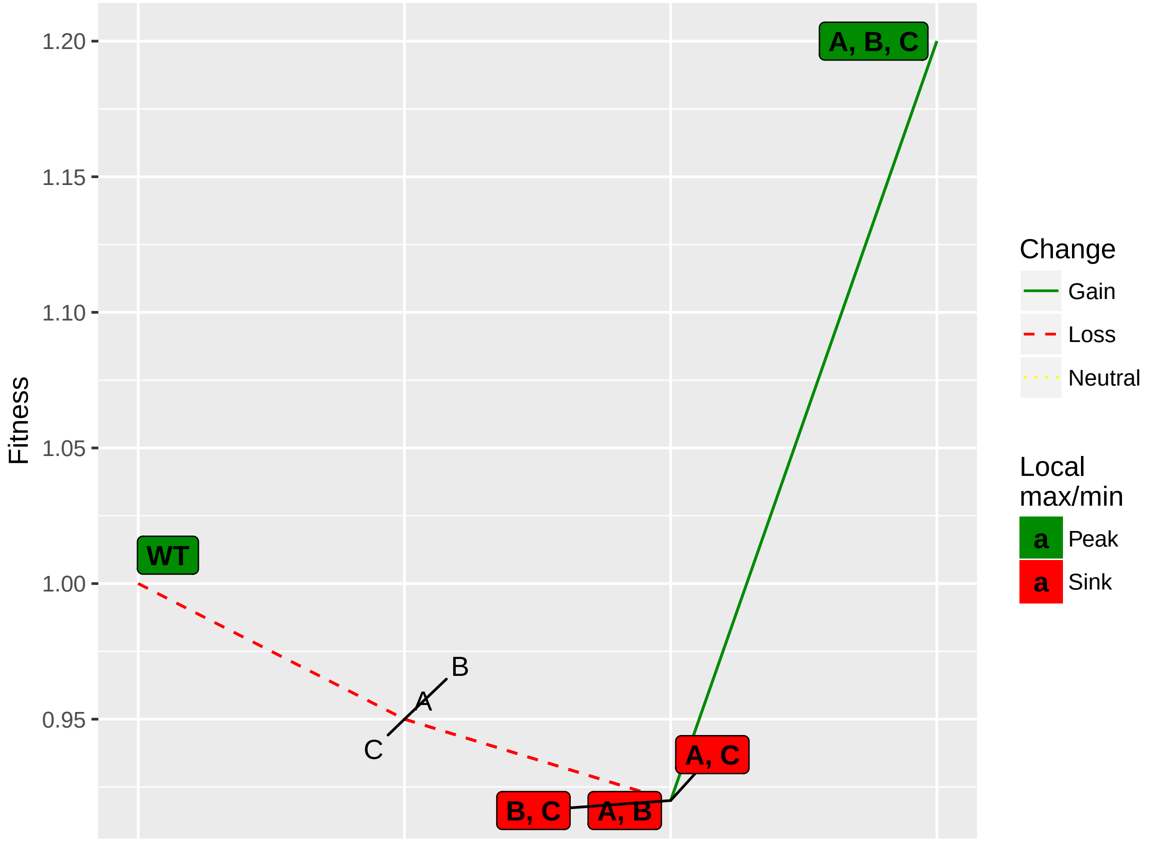

This is example (section 5.5) will give a very busy plot:

```
par(cex = 0.7)
pancr <- allFitnessEffects(
    data.frame(parent = c("Root", rep("KRAS", 4),
                   "SMAD4", "CDNK2A",
                   "TP53", "TP53", "MLL3"),
               child = c("KRAS","SMAD4", "CDNK2A",
                   "TP53", "MLL3",
                   rep("PXDN", 3), rep("TGFBR2", 2)),
               s = 0.1,
               sh = -0.9,
               typeDep = "MN"))
plot(evalAllGenotypes(pancr, order = FALSE), use_ggrepel = TRUE)
```

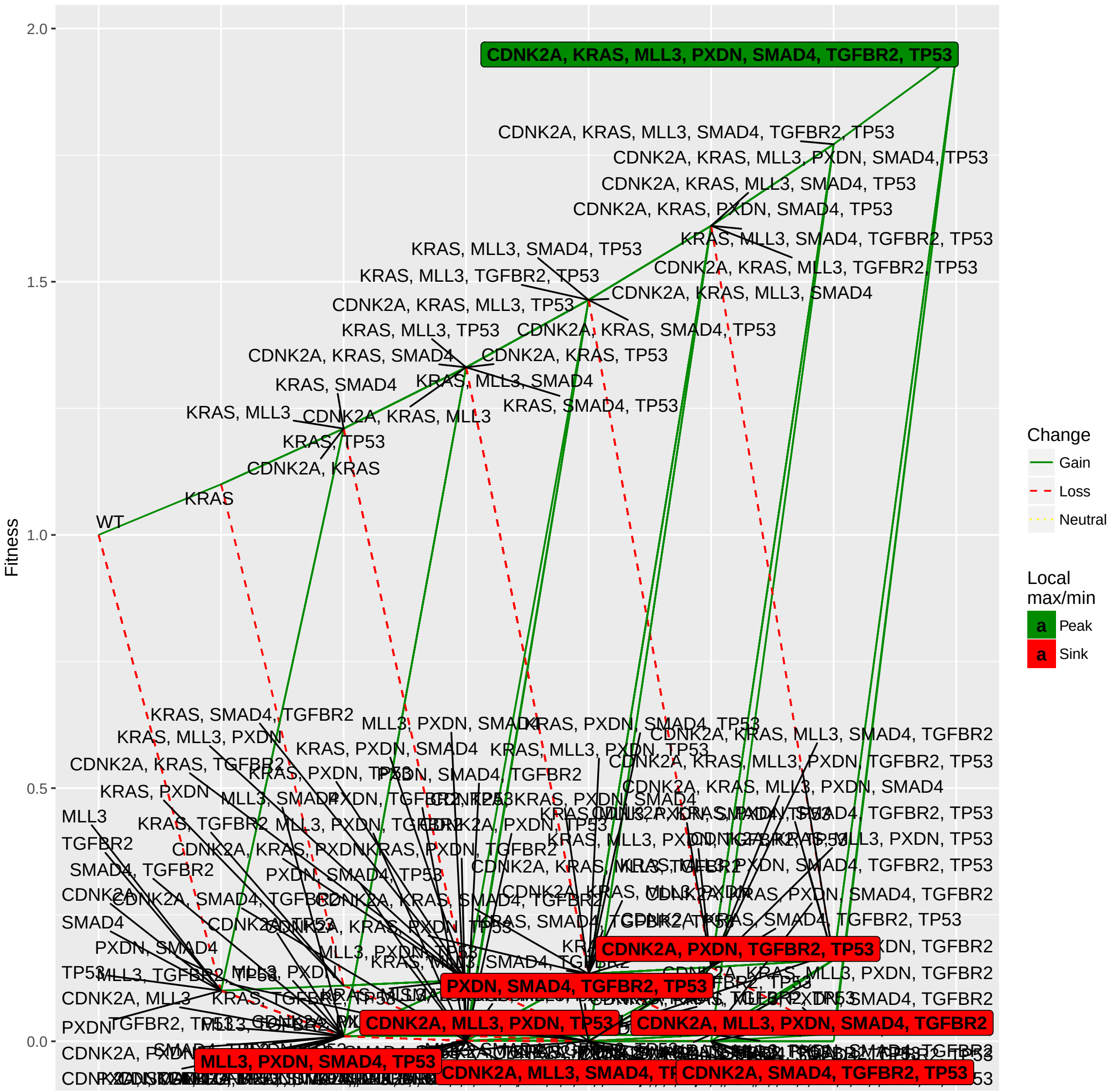

5 Specifying fitness effects: some examples from the literature

5.1 Bauer et al., 2014

In the model of Bauer and collaborators (Bauer, Siebert, and Traulsen 2014, 54) we have "For cells without the primary driver mutation, each secondary driver mutation leads to a change in the cell's fitness by s_P. For cells with the primary driver mutation, the fitness advantage obtained with each secondary driver mutation is s_{DP}."

The proliferation probability is given as:

- $\frac{1}{2}(1+s_p)^k$ when there are k secondary drivers mutated and no primary diver;
- $\frac{1}{2}\frac{1+S_D^+}{1+S_D^-}(1+S_{DP})^k$ when the primary driver is mutated;

apoptosis is one minus the proliferation rate.

5.1.1 Using a DAG

We cannot find a simple mapping from their expressions to our fitness parameterization, but we can get fairly close by using a DAG; in this one, note the unusual feature of having one of the "s" terms (that for the driver dependency on root) be negative. Using the parameters given in the legend of their Figure 3 for $s_p, S_D^+, S_D^-, S_{DP}$ and obtaining that negative value for the dependency of the driver on root we can do:

```
K <- 4
sp <- 1e-5
sdp <- 0.015
sdplus <- 0.05
sdminus <- 0.1
cnt <- (1 + sdplus)/(1 + sdminus)
prod_cnt <- cnt - 1
bauer <- data.frame(parent = c("Root", rep("D", K)),
                    child = c("D", paste0("s", 1:K)),
                    s = c(prod_cnt, rep(sdp, K)),
                    sh = c(0, rep(sp, K)),
                    typeDep = "MN")
fbauer <- allFitnessEffects(bauer)
(b1 <- evalAllGenotypes(fbauer, order = FALSE, addwt = TRUE))
##             Genotype Fitness
## 1                 WT  1.0000
## 2                  D  0.9545
## 3                 s1  1.0000
## 4                 s2  1.0000
## 5                 s3  1.0000
```

```
## 6                   s4  1.0000
## 7                D, s1  0.9689
## 8                D, s2  0.9689
## 9                D, s3  0.9689
## 10               D, s4  0.9689
## 11              s1, s2  1.0000
## 12              s1, s3  1.0000
## 13              s1, s4  1.0000
## 14              s2, s3  1.0000
## 15              s2, s4  1.0000
## 16              s3, s4  1.0000
## 17          D, s1, s2  0.9834
## 18          D, s1, s3  0.9834
## 19          D, s1, s4  0.9834
## 20          D, s2, s3  0.9834
## 21          D, s2, s4  0.9834
## 22          D, s3, s4  0.9834
## 23         s1, s2, s3  1.0000
## 24         s1, s2, s4  1.0000
## 25         s1, s3, s4  1.0000
## 26         s2, s3, s4  1.0000
## 27     D, s1, s2, s3  0.9981
## 28     D, s1, s2, s4  0.9981
## 29     D, s1, s3, s4  0.9981
## 30     D, s2, s3, s4  0.9981
## 31    s1, s2, s3, s4  1.0000
## 32 D, s1, s2, s3, s4  1.0131
```

(We use "D" for "driver" or "primary driver", as is it is called in the original paper, and "s" for secondary drivers, somewhat similar to passengers).

Note that what we specify as "typeDep" is irrelevant (MN, SMN, or XMPN make no difference).

This is the DAG:

```
plot(fbauer)
```

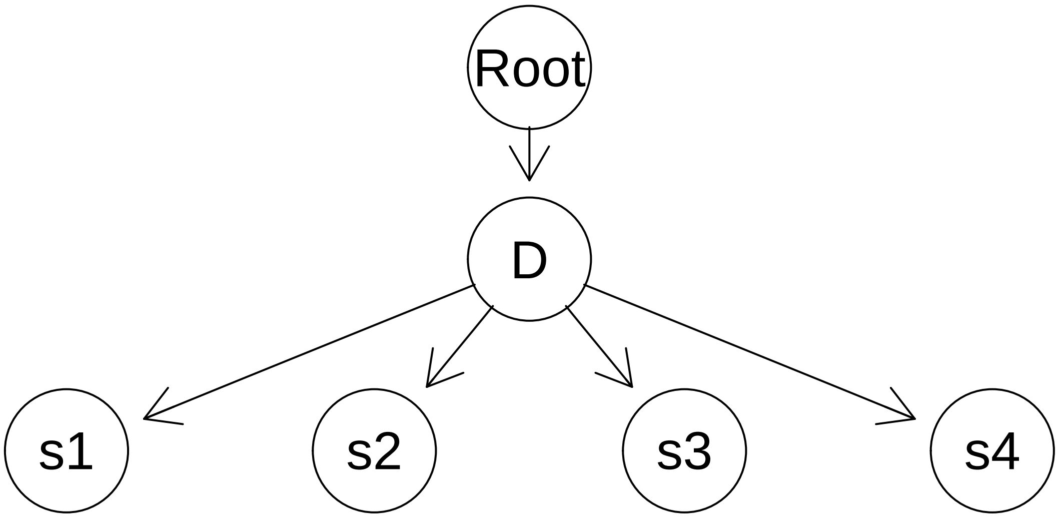

And if you compare the tabular output of `evalAllGenotypes` you can see that the values of fitness reproduces the fitness landscape that they show in their Figure 1. We can also use our plot for fitness landscapes:

```
plot(b1, use_ggrepel = TRUE)
```

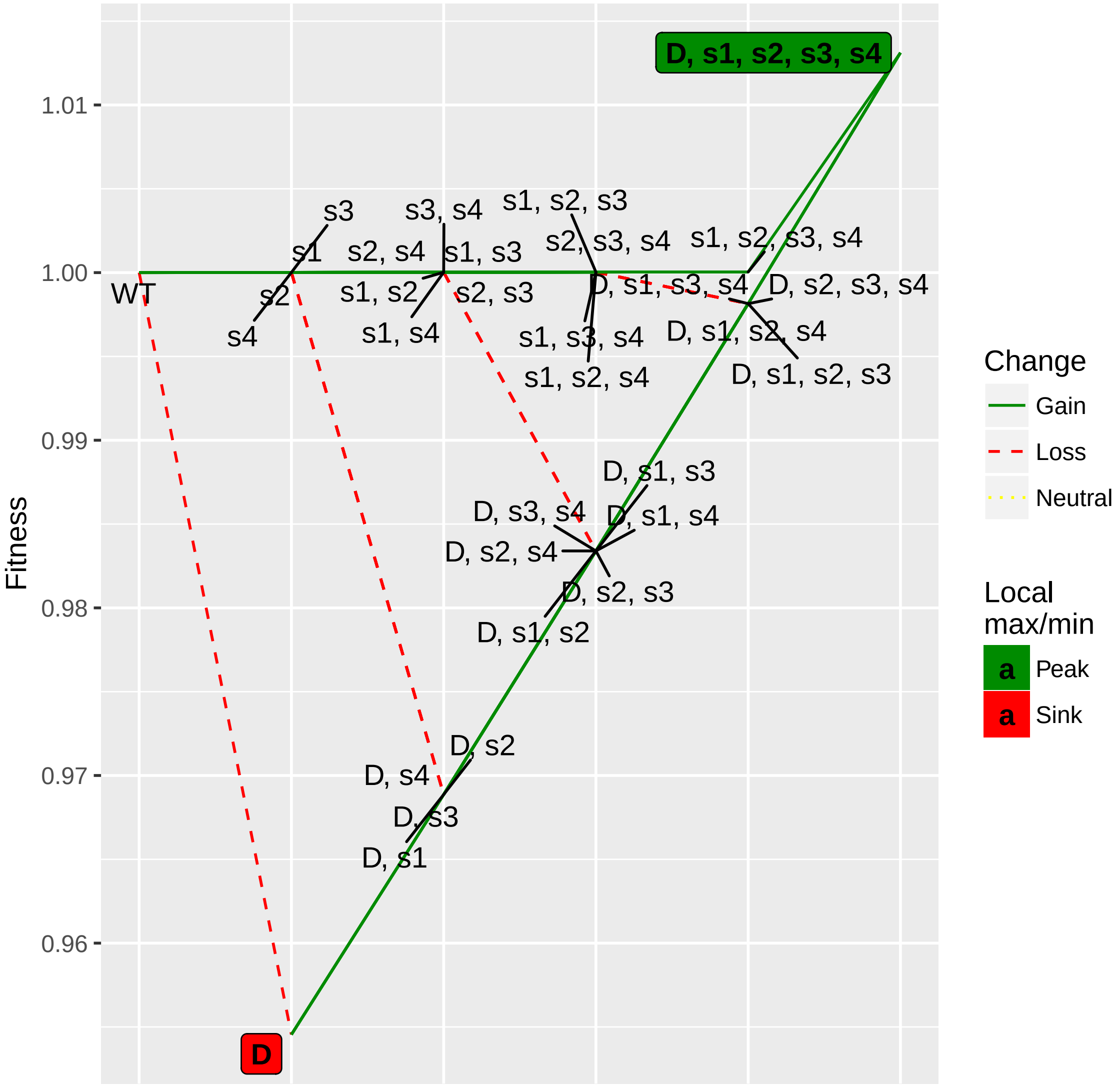

5.1.2 Specifying fitness of genotypes directly

An alternative approach to specify the fitness, if the number of genotypes is reasonably small, is to directly evaluate fitness as given by their expressions. Then, use the `genotFitness` argument to `allFitnessEffects`.

We will create all possible genotypes; then we will write a function that gives the fitness of each genotype according to their expression; finally, we will call this function on the data frame of genotypes, and pass this data frame to `allFitnessEffects`.

```
m1 <- expand.grid(D = c(1, 0), s1 = c(1, 0), s2 = c(1, 0),
                  s3 = c(1, 0), s4 = c(1, 0))

fitness_bauer <- function(D, s1, s2, s3, s4,
                          sp = 1e-5, sdp = 0.015, sdplus = 0.05,
                          sdminus = 0.1) {
    if(!D) {
```

```r
        b <- 0.5 * ( (1 + sp)^(sum(c(s1, s2, s3, s4))))
    } else {
        b <- 0.5 *
            (((1 + sdplus)/(1 + sdminus)  *
              (1 + sdp)^(sum(c(s1, s2, s3, s4)))))
    }
    fitness <- b - (1 - b)
    our_fitness <- 1 + fitness ## prevent negative fitness and
    ## make wt fitness = 1
    return(our_fitness)
}

m1$Fitness <-
    apply(m1, 1, function(x) do.call(fitness_bauer, as.list(x)))

bauer2 <- allFitnessEffects(genotFitness = m1)
```

Now, show the fitness of all genotypes:

```r
evalAllGenotypes(bauer2, order = FALSE, addwt = TRUE)
##              Genotype Fitness
## 1                  WT  1.0000
## 2                   D  0.9545
## 3                  s1  1.0000
## 4                  s2  1.0000
## 5                  s3  1.0000
## 6                  s4  1.0000
## 7              D, s1  0.9689
## 8              D, s2  0.9689
## 9              D, s3  0.9689
## 10             D, s4  0.9689
## 11            s1, s2  1.0000
## 12            s1, s3  1.0000
## 13            s1, s4  1.0000
## 14            s2, s3  1.0000
## 15            s2, s4  1.0000
## 16            s3, s4  1.0000
## 17        D, s1, s2  0.9834
## 18        D, s1, s3  0.9834
## 19        D, s1, s4  0.9834
## 20        D, s2, s3  0.9834
## 21        D, s2, s4  0.9834
## 22        D, s3, s4  0.9834
## 23       s1, s2, s3  1.0000
## 24       s1, s2, s4  1.0000
## 25       s1, s3, s4  1.0000
```

```
## 26         s2, s3, s4  1.0000
## 27      D, s1, s2, s3  0.9981
## 28      D, s1, s2, s4  0.9981
## 29      D, s1, s3, s4  0.9981
## 30      D, s2, s3, s4  0.9981
## 31     s1, s2, s3, s4  1.0000
## 32 D, s1, s2, s3, s4  1.0131
```

Can we use modules in this example, if we use the "lego system"? Sure, as in any other case.

5.2 Misra et al., 2014

Figure 1 of Misra, Szczurek, and Vingron (2014) presents three scenarios which are different types of epistasis.

5.2.1 Example 1.a

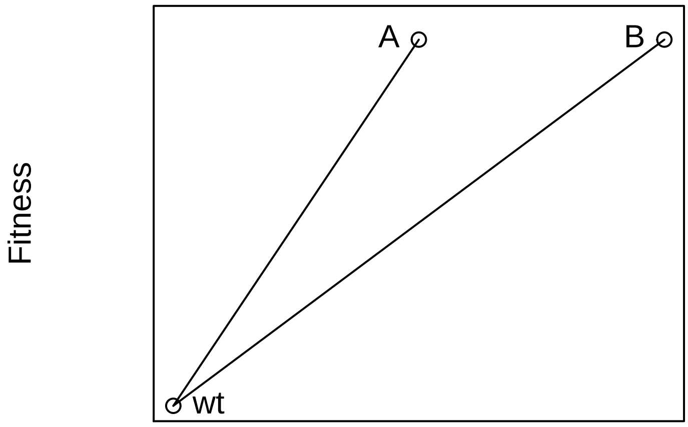

In that figure it is evident that the fitness effect of "A" and "B" are the same. There are two different models depending on whether "AB" is just the product of both, or there is epistasis. In the first case probably the simplest is:

```
s <- 0.1 ## or whatever number
m1a1 <- allFitnessEffects(data.frame(parent = c("Root", "Root"),
                                     child = c("A", "B"),
                                     s = s,
                                     sh = 0,
                                     typeDep = "MN"))
evalAllGenotypes(m1a1, order = FALSE, addwt = TRUE)
##   Genotype Fitness
## 1       WT    1.00
## 2        A    1.10
```

```
## 3          B     1.10
## 4       A, B     1.21
```

If the double mutant shows epistasis, as we saw before (section 3.7.1) we have a range of options. For example:

```
s <- 0.1
sab <- 0.3
m1a2 <- allFitnessEffects(epistasis = c("A:-B" = s,
                                        "-A:B" = s,
                                        "A:B" = sab))
evalAllGenotypes(m1a2, order = FALSE, addwt = TRUE)
##   Genotype Fitness
## 1       WT     1.0
## 2        A     1.1
## 3        B     1.1
## 4     A, B     1.3
```

But we could also modify the graph dependency structure, and we have to change the value of the coefficient, since that is what multiplies each of the terms for "A" and "B": $(1 + s_{AB}) = (1 + s)^2(1 + s_{AB3})$

```
sab3 <- ((1 + sab)/((1 + s)^2)) - 1
m1a3 <- allFitnessEffects(data.frame(parent = c("Root", "Root"),
                                     child = c("A", "B"),
                                     s = s,
                                     sh = 0,
                                     typeDep = "MN"),
                          epistasis = c("A:B" = sab3))
evalAllGenotypes(m1a3, order = FALSE, addwt = TRUE)
##   Genotype Fitness
## 1       WT     1.0
## 2        A     1.1
## 3        B     1.1
## 4     A, B     1.3
```

And, obviously

```
all.equal(evalAllGenotypes(m1a2, order = FALSE, addwt = TRUE),
          evalAllGenotypes(m1a3, order = FALSE, addwt = TRUE))
## [1] TRUE
```

5.2.2 Example 1.b

This is a specific case of synthetic viability (see also section 3.9):

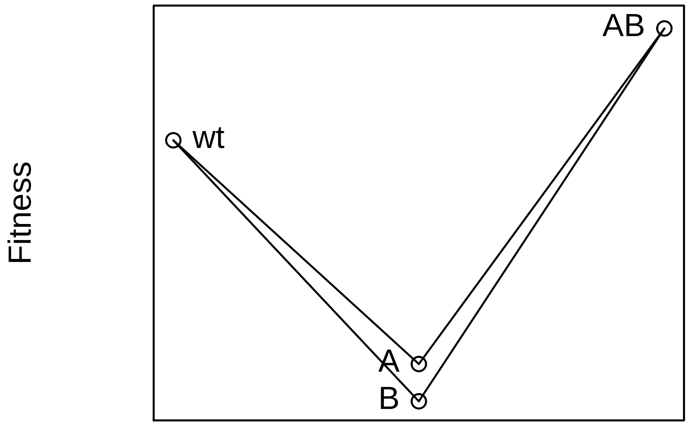

Here, $S_A, S_B < 0$, $S_B < 0$, $S_{AB} > 0$ and $(1 + S_{AB})(1 + S_A)(1 + S_B) > 1$.

As before, we can specify this in several different ways. The simplest is to specify all genotypes:

```
sa <- -0.6
sb <- -0.7
sab <- 0.3
m1b1 <- allFitnessEffects(epistasis = c("A:-B" = sa,
                                        "-A:B" = sb,
                                        "A:B" = sab))
evalAllGenotypes(m1b1, order = FALSE, addwt = TRUE)
##   Genotype Fitness
## 1       WT     1.0
## 2        A     0.4
## 3        B     0.3
## 4     A, B     1.3
```

We could also use a tree and modify the "sab" for the epistasis, as before (5.2.1).

5.2.3 Example 1.c

The final case, in figure 1.c of Misra et al., is just epistasis, where a mutation in one of the genes is deleterious (possibly only mildly), in the other is beneficial, and the double mutation has fitness larger than any of the other two.

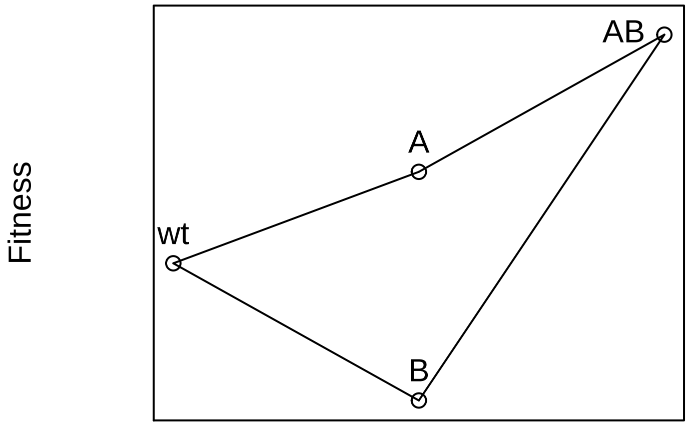

Here we have that $s_A > 0$, $s_B < 0$, $(1 + s_{AB})(1 + s_A)(1 + s_B) > (1 + s_{AB})$ so $s_{AB} > \frac{-s_B}{1+s_B}$

As before, we can specify this in several different ways. The simplest is to specify all genotypes:

```
sa <- 0.2
sb <- -0.3
sab <- 0.5
m1c1 <- allFitnessEffects(epistasis = c("A:-B" = sa,
                                        "-A:B" = sb,
                                        "A:B" = sab))
evalAllGenotypes(m1c1, order = FALSE, addwt = TRUE)
##   Genotype Fitness
## 1       WT     1.0
## 2        A     1.2
## 3        B     0.7
## 4     A, B     1.5
```

We could also use a tree and modify the "sab" for the epistasis, as before (5.2.1).

5.3 Ochs and Desai, 2015

In Ochs and Desai (2015) the authors present a model shown graphically as (the actual numerical values are arbitrarily set by me):

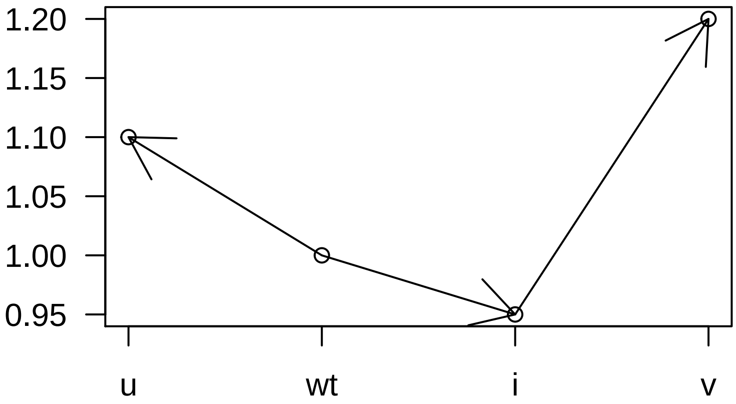

In their model, $s_u > 0$, $s_v > s_u$, $s_i < 0$, we can only arrive at v from i, and the mutants "ui" and "uv" can never appear as their fitness is 0, or $-\infty$, so $s_{ui} = s_{uv} = -1$ (or $-\infty$).

We can specify this combining a graph and epistasis specifications:

```
su <- 0.1
si <- -0.05
fvi <- 1.2 ## the fitness of the vi mutant
sv <- (fvi/(1 + si)) - 1
sui <- suv <- -1
od <- allFitnessEffects(
    data.frame(parent = c("Root", "Root", "i"),
               child = c("u", "i", "v"),
               s = c(su, si, sv),
               sh = -1,
               typeDep = "MN"),
    epistasis = c(
        "u:i" = sui,
        "u:v" = suv))
```

A figure showing that model is

```
plot(od)
```

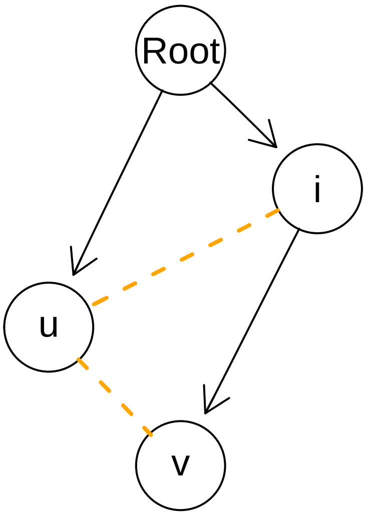

And the fitness of all genotype is

```
evalAllGenotypes(od, order = FALSE, addwt = TRUE)
##   Genotype Fitness
## 1       WT    1.00
## 2        i    0.95
## 3        u    1.10
## 4        v    0.00
## 5     i, u    0.00
## 6     i, v    1.20
## 7     u, v    0.00
## 8  i, u, v    0.00
```

We could alternatively have specified fitness either directly specifying the fitness of each genotype or specifying epistatic effects. Let us use the second approach:

```
u <- 0.2; i <- -0.02; vi <- 0.6; ui <- uv <- -Inf
od2 <- allFitnessEffects(
    epistasis = c("u" = u,  "u:i" = ui,
                  "u:v" = uv, "i" = i,
                  "v:-i" = -Inf, "v:i" = vi))
evalAllGenotypes(od, addwt = TRUE)
##   Genotype Fitness
## 1       WT    1.00
## 2        i    0.95
## 3        u    1.10
## 4        v    0.00
## 5     i, u    0.00
## 6     i, v    1.20
## 7     u, v    0.00
## 8  i, u, v    0.00
```

We will return to this model when we explain the usage of `fixation` for stopping the simulations (see 6.3.3).

5.4 Weissman et al., 2009

In their figure 1a, Weissman et al. (2009) present this model (actual numeric values are set arbitrarily)

5.4.1 Figure 1.a

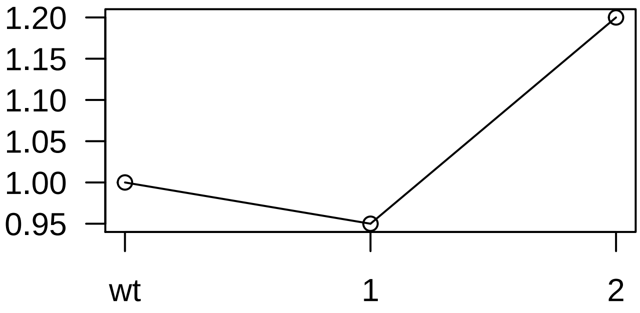

where the "1" and "2" in the figure refer to the total number of mutations in two different loci. This is, therefore, very similar to the example in section 5.2.2. Here we have, in their notation, $\delta_1 < 0$, fitness of single "A" or single "B" $= 1 + \delta_1$, $S_{AB} > 0$, $(1 + S_{AB})(1 + \delta_1)^2 > 1$.

5.4.2 Figure 1.b

In their figure 1b they show

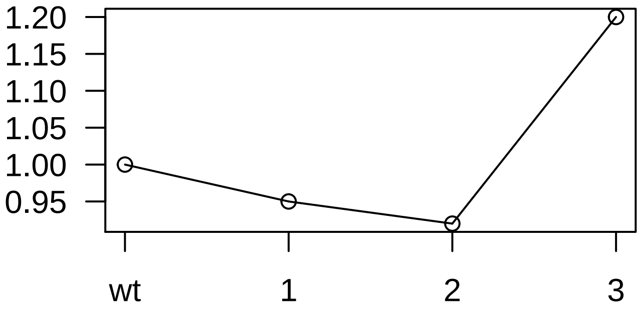

Where, as before, 1, 2, 3, denote the total number of mutations over three different loci and $\delta_1 < 0$, $\delta_2 < 0$, fitness of single mutant is $(1 + \delta_1)$, of double mutant is $(1 + \delta_2)$ so that $(1 + \delta_2) = (1 + \delta_1)^2(1 + s_2)$ and of triple mutant is $(1 + \delta_3)$, so that $(1 + \delta_3) = (1 + \delta_1)^3(1 + s_2)^3(1 + s_3)$.

We can specify this combining a graph with epistasis:

```
d1 <- -0.05 ## single mutant fitness 0.95
d2 <- -0.08 ## double mutant fitness 0.92
d3 <- 0.2   ## triple mutant fitness 1.2

s2 <- ((1 + d2)/(1 + d1)^2) - 1
s3 <- ( (1 + d3)/((1 + d1)^3 * (1 + s2)^3) ) - 1
```

```r
w <- allFitnessEffects(
    data.frame(parent = c("Root", "Root", "Root"),
               child = c("A", "B", "C"),
               s = d1,
               sh = -1,
               typeDep = "MN"),
    epistasis = c(
        "A:B" = s2,
        "A:C" = s2,
        "B:C" = s2,
        "A:B:C" = s3))
```

The model can be shown graphically as:

```r
plot(w)
```

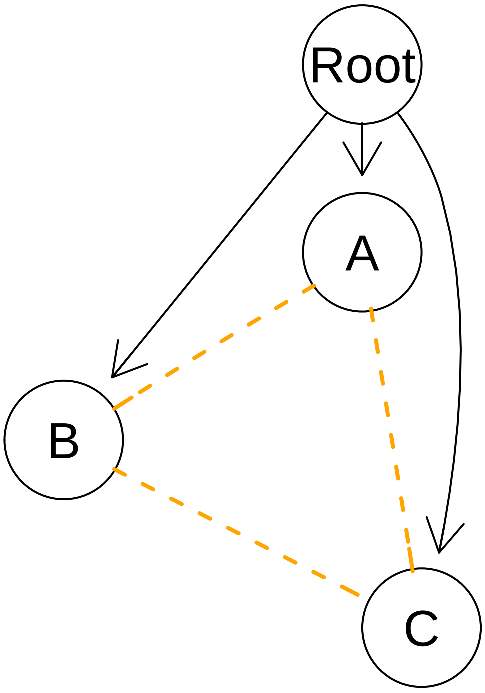

And fitness of all genotypes is:

```r
evalAllGenotypes(w, order = FALSE, addwt = TRUE)
##   Genotype Fitness
## 1       WT    1.00
## 2        A    0.95
## 3        B    0.95
## 4        C    0.95
## 5     A, B    0.92
## 6     A, C    0.92
## 7     B, C    0.92
## 8  A, B, C    1.20
```

Alternatively, we can directly specify what each genotype adds to the fitness, given the included genotype. This is basically replacing the graph by giving each of "A", "B", and "C" directly:

```
wb <- allFitnessEffects(
    epistasis = c(
        "A" = d1,
        "B" = d1,
        "C" = d1,
        "A:B" = s2,
        "A:C" = s2,
        "B:C" = s2,
        "A:B:C" = s3))

evalAllGenotypes(wb, order = FALSE, addwt = TRUE)
##   Genotype Fitness
## 1       WT    1.00
## 2        A    0.95
## 3        B    0.95
## 4        C    0.95
## 5     A, B    0.92
## 6     A, C    0.92
## 7     B, C    0.92
## 8  A, B, C    1.20
```

The plot, of course, is not very revealing and we cannot show that there is a three-way interaction (only all three two-way interactions):

```
plot(wb)
```

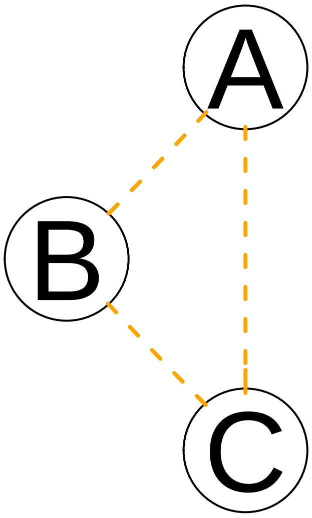

As we have seen several times already (sections 3.7.1, 3.7.2, 3.7.3) we can also give the genotypes directly and, consequently, the fitness of each genotype (not the added contribution):

```r
wc <- allFitnessEffects(
    epistasis = c(
        "A:-B:-C" = d1,
        "B:-C:-A" = d1,
        "C:-A:-B" = d1,
        "A:B:-C" = d2,
        "A:C:-B" = d2,
        "B:C:-A" = d2,
        "A:B:C" = d3))
evalAllGenotypes(wc, order = FALSE, addwt = TRUE)
##   Genotype Fitness
## 1       WT    1.00
## 2        A    0.95
## 3        B    0.95
## 4        C    0.95
## 5     A, B    0.92
## 6     A, C    0.92
## 7     B, C    0.92
## 8  A, B, C    1.20
```

5.5 Gerstung et al., 2011, pancreatic cancer poset

Similar to what we did in v.1 (see section 13.1) we can specify the pancreatic cancer poset in Gerstung et al. (2011) (their figure 2B, left). We use directly the names of the genes, since that is immediately supported by the new version.

```r
pancr <- allFitnessEffects(
    data.frame(parent = c("Root", rep("KRAS", 4),
                   "SMAD4", "CDNK2A",
                   "TP53", "TP53", "MLL3"),
               child = c("KRAS","SMAD4", "CDNK2A",
                   "TP53", "MLL3",
                   rep("PXDN", 3), rep("TGFBR2", 2)),
               s = 0.1,
               sh = -0.9,
               typeDep = "MN"))

plot(pancr)
```

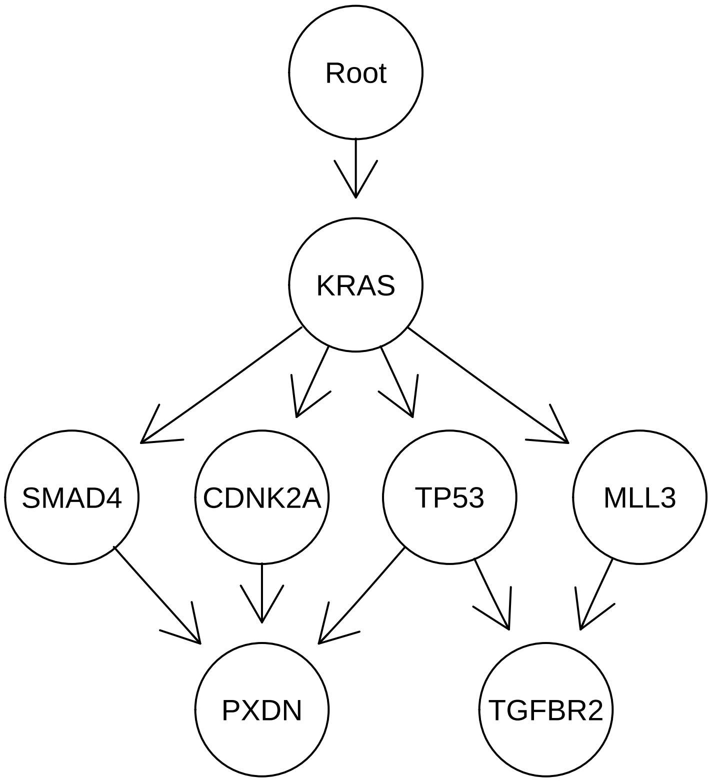

Of course the "s" and "sh" are set arbitrarily here.

5.6 Raphael and Vandin's 2014 modules

In Raphael and Vandin (2015), the authors show several progression models in terms of modules. We can code the extended poset for the colorectal cancer model in their Figure 4.a is (s and sh are arbitrary):

```
rv1 <- allFitnessEffects(data.frame(parent = c("Root", "A", "KRAS"),
                                    child = c("A", "KRAS", "FBXW7"),
                                    s = 0.1,
                                    sh = -0.01,
                                    typeDep = "MN"),
                         geneToModule = c("Root" = "Root",
                             "A" = "EVC2, PIK3CA, TP53",
                             "KRAS" = "KRAS",
                             "FBXW7" = "FBXW7"))

plot(rv1, expandModules = TRUE, autofit = TRUE)
```

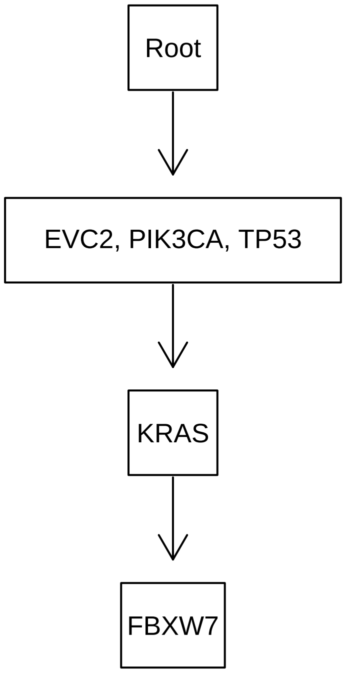

We have used the (experimental) `autofit` option to fit the labels to the edges. Note how we can use the same name for genes and modules, but we need to specify all the modules.

Their Figure 5b is

```r
rv2 <- allFitnessEffects(
    data.frame(parent = c("Root", "1", "2", "3", "4"),
               child = c("1", "2", "3", "4", "ELF3"),
               s = 0.1,
               sh = -0.01,
               typeDep = "MN"),
    geneToModule = c("Root" = "Root",
                     "1" = "APC, FBXW7",
                     "2" = "ATM, FAM123B, PIK3CA, TP53",
                     "3" = "BRAF, KRAS, NRAS",
                     "4" = "SMAD2, SMAD4, SOX9",
                     "ELF3" = "ELF3"))

plot(rv2, expandModules = TRUE,   autofit = TRUE)
```

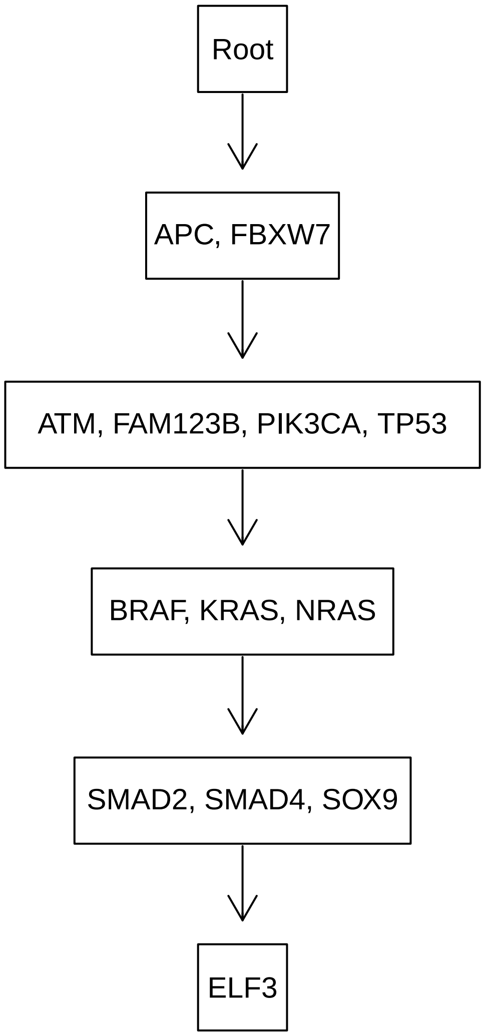
Root
APC, FBXW7
ATM, FAM123B, PIK3CA, TP53
BRAF, KRAS, NRAS
SMAD2, SMAD4, SOX9
ELF3

6 Running and plotting the simulations: starting, ending, and examples

6.1 Starting and ending

After you have decided the specifics of the fitness effects and the model, you need to decide:

- Where will you start your simulation from. This involves deciding the initial population size (argument `initSize`) and, possibly, the genotype of the initial population; the later is covered in section 6.2.
- When will you stop it: how long to run it, and whether or not to require simulations to reach cancer (under some definition of what it means to reach cancer). This is covered in 6.3.

6.2 Can I start the simulation from a specific mutant?

You bet. In version 2 you can specify the genotype for the initial mutant with the same flexibility as in `evalGenotype`. Here we show a couple of examples (we use the representation of the parent-child relationships —discussed in section 8— of the clones so that you can see which clones appear, and from which, and check that we are not making mistakes).

```
o3init <- allFitnessEffects(orderEffects = c(
                            "M > D > F" = 0.99,
                            "D > M > F" = 0.2,
                            "D > M"     = 0.1,
                            "M > D"     = 0.9),
                        noIntGenes = c("u" = 0.01,
                                       "v" = 0.01,
                                       "w" = 0.001,
                                       "x" = 0.0001,
                                       "y" = -0.0001,
                                       "z" = -0.001),
                        geneToModule =
                            c("M" = "m",
                              "F" = "f",
                              "D" = "d") )

oneI <- oncoSimulIndiv(o3init, model = "McFL",
                       mu = 5e-5, finalTime = 500,
                       detectionDrivers = 3,
                       onlyCancer = FALSE,
                       initSize = 1000,
```

```r
                           keepPhylog = TRUE,
                           initMutant = c("m > u > d")
                           )
plotClonePhylog(oneI, N = 0)
```

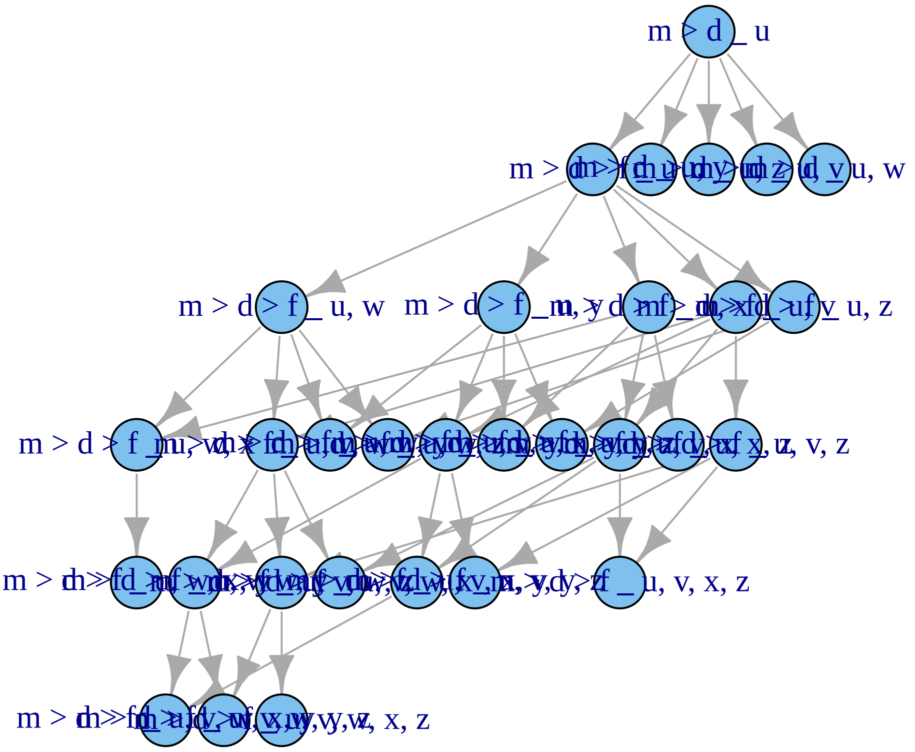

```r
## Note we also disable the stopping stochastically as a function of size
## to allow the population to grow large and generate may different
## clones.
ospI <- oncoSimulPop(2,
                     o3init, model = "Exp",
                     mu = 5e-5, finalTime = 500,
                     detectionDrivers = 3,
                     onlyCancer = TRUE,
                     initSize = 10,
                     keepPhylog = TRUE,
                     initMutant = c("d > m > z"),
                     mc.cores = 2
                     )
op <- par(mar = rep(0, 4), mfrow = c(1, 2))
plotClonePhylog(ospI[[1]])
plotClonePhylog(ospI[[2]])
```

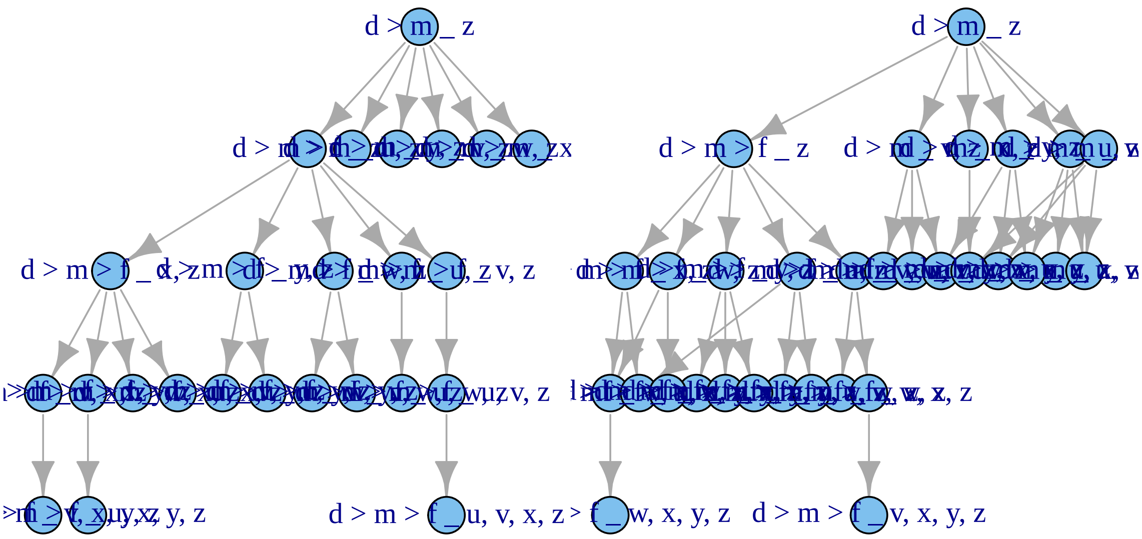


```
par(op)

ossI <- oncoSimulSample(2,
                        o3init, model = "Exp",
                        mu = 5e-5, finalTime = 500,
                        detectionDrivers = 2,
                        onlyCancer = TRUE,
                        initSize = 10,
                        initMutant = c("z > d"),
                        ## check presence of initMutant:
                        thresholdWhole = 1
                    )
## Successfully sampled 2 individuals
##
##  Subjects by Genes matrix of 2 subjects and 9 genes.

## No phylogeny is kept with oncoSimulSample, but look at the
## OcurringDrivers and the sample
ossI$popSample
##      d f m u v w x y z
## [1,] 1 0 0 0 0 0 0 0 1
## [2,] 1 0 0 0 0 0 0 0 1
ossI$popSummary[, "OccurringDrivers", drop = FALSE]
##   OccurringDrivers
## 1
## 2
```

6.3 Ending the simulations

OncoSimulR provides very flexible ways to decide when to stop a simulation. Here we focus on a single simulation; see further options with multiple simulations in 7.

6.3.1 Ending the simulations: conditions

- `onlyCancer = TRUE`. A simulation will be repeated until any one of the "reach cancer" conditions is met, if this happens before the simulation reaches `finalTime`[10]. These conditions are:
  - Total population size becomes larger than `detectionSize`.
  - The number of drivers in any one genotype or clone becomes equal to, or larger than, `detectionDrivers`; note that this allows you to stop the simulation as soon as a **specific genotype** is found, by using exactly and only the genes that make that genotype as the drivers.
  - A gene or gene combination among those listed in `fixation` becomes fixed in the population (i.e., has a frequency is 1) (see details in (6.3.3).
  - The tumor is detected according to a stochastic detection mechanism, where the probability of "detecting the tumor" increases with population size; this is explained below (6.3.2) and is controlled by argument `detectionProb`.

  As we exit as soon as any of the exiting conditions is reached, if you only care about one condition, set the other to `NA` (see also section 6.3.2.1).
- `onlyCancer = FALSE`. A simulation will run only once, and will exit as soon as any of the above conditions are met or as soon as the total population size becomes zero or we reach `finalTime`.

As an example of `onlyCancer = TRUE`, focusing on the first two mechanisms, suppose you give `detectionSize = 1e4` and `detectionDrivers =3` (and you have `detectionProb = NA`). A simulation will exit as soon as it reaches a total population size of 10^4 or any clone has four drivers, whichever comes first (if any of these happen before `finalTime`).

In the `onlyCancer = TRUE` case, what happens if we reach `finalTime` (or the population size becomes zero) before any of the "reach cancer" conditions have been fulfilled? The simulation will be repeated again, within the following limits:

- `max.wall.time`: the total wall time we allow an individual simulation to run;
- `max.num.tries`: the maximum number of times we allow a simulation to be repeated to reach cancer;
- `max.wall.time.total` and `max.num.tries.total`, similar to the above but over a set of simulations in function `oncoSimulSample`.

Incidentally, we keep track of the number of attempts used (the component `other$attemptsUsed$`) before we reach cancer, so you can estimate (as from a negative binomial sampling) the probability of reaching your desired end point under different scenarios.

The `onlyCancer = FALSE` case might be what you want to do when you examine general population genetics scenarios without focusing on possible sampling issues.

[10] Of course, the "reach cancer" idea and the `onlyCancer` argument are generic names; this could have been labeled "reach whatever interests me".

To do this, set `finalTime` to the value you want and set `onlyCancer = FALSE`; in addition, set `detectionProb` to "NA" and `detectionDrivers` and `detectionSize` to "NA" or to huge numbers[11]. In this scenario you simply collect the simulation output at the end of the run, regardless of what happened with the population (it became extinct, it did not reach a large size, it did not accumulate drivers, etc).

6.3.2 Stochastic detection mechanism: "detectionProb"

This is the process that is controlled by the argument `detectionProb`. Here the probability of tumor detection increases with the total population size. This is biologically a reasonable assumption: the larger the tumor, the more likely it is it will be detected.

At regularly spaced times during the simulation, we compute the probability of detection as a function of size and determine (by comparing against a random uniform number) if the simulation should finish. For simplicity, and to make sure the probability is bounded between 0 and 1, we use the function

$$P(N) = \begin{cases} 1 - e^{-cPDetect(N - PDBaseline)} & \text{if } N > PDBaseline \\ 0 & \text{if } N \leq PDBaseline \end{cases} \quad (1)$$

where $P(N)$ is the probability that a tumor with a population size N will be detected, and *cPDetect* controls the increase in $P(N)$ with population size; with $PDBaseline$ we both control the minimal population size at which this mechanism stats operating (because we will rarely want detection unless there is some meaningful increase of population size over `initSize`) and we model the increase in $P(N)$ as a function of differences with respect to $PDBaseline$.

The $P(N)$ refers to the probability of detection at each one of the occasions when we assess the probability of exiting. When, or how often, do we do that? When we assess probability of exiting is controlled by `checkSizePEvery`, which will often be much larger than `sampleEvery`[12]. Biologically, a way to think of `checkSizePEvery` is "time between doctor appointments".

An important **warning**, though: for populations that are growing very, very fast or where some genes might have very large effects on fitness even a moderate `checkSizePEvery` of, say, 10, might be inappropriate, since populations could have increased by several orders of magnitude between successive checks. This issue is also discussed in section 2.1.1 and 2.2.

[11]Setting `detectionDrivers` and `detectionSize` to "NA" is in fact equivalent to setting them to the largest possible numbers for these variables: $2^{32} - 1$ and ∞, respectively.

[12]We assess probability of exiting at every sampling time, as given by `sampleEvery`, that is the smallest possible sampling time that is separated from the previous time of assessment by at least `checkSizePEvery`. In other words, the interval between successive assessments will be the smallest multiple integer of `sampleEvery` that is larger than `checkSizePEvery`. For example, suppose `sampleEvery = 2` and `checkSizePEvery = 3`: we will assess exiting at times $4, 8, 12, 16, \ldots$. If `sampleEvery = 3` and `checkSizePEvery = 3`: we will assess exiting at times $6, 12, 18, \ldots$.

Finally, you can specify $cPDetect$ directly (you will need to set `n2` and `p2` to NA). However, it might be more intuitive to specify the pair `n2`, `p2`, such that $P(n2) = p2$ (and from that pair we solve for the value of $cPDetect$).

You can get a feeling for the effects of these arguments by playing with the following code, that we do not execute here for the sake of speed. Here no mutation has any effect, but there is a non-zero probability of exiting as soon as the total population size becomes larger than the initial population size. So, eventually, all simulations will exit and, as we are using the McFarland model, population size will vary slightly around the initial population size.

```
gi2 <- rep(0, 5)
names(gi2) <- letters[1:5]
oi2 <- allFitnessEffects(noIntGenes = gi2)
s5 <- oncoSimulPop(200,
                   oi2,
                   model = "McFL",
                   initSize = 1000,
                   detectionProb = c(p2 = 0.1,
                                     n2 = 2000,
                                     PDBaseline = 1000,
                                     checkSizePEvery = 2),
                   detectionSize = NA,
                   finalTime = NA,
                   keepEvery = NA,
                   detectionDrivers = NA)
s5
hist(unlist(lapply(s5, function(x) x$FinalTime)))
```

As you decrease `checkSizePEvery` the distribution of "FinalTime" will resemble more and more an exponential distribution.

In this vignette, there are some further examples of using this mechanism in 6.5.4 and 6.5.2, with the default arguments.

6.3.2.1 Stochastic detection mechanism and minimum number of drivers

We said above that we exit as soon as any of the conditions is reached (i.e., we use an OR operation over the exit conditions). There is a special exception to this procedure: if you set `AND_DrvProbExit = TRUE`, both the number of drivers and the `detectionProb` mechanism condition must fulfilled. This means that the `detectionProb` mechanism not assessed unless the `detectionDrivers` condition is. Using `AND_DrvProbExit = TRUE` allows to run simulations and ensure that all of the returned simulations will have at least some cells with the number of drivers as specified by `detectionDrivers`. Note, though, that this does not guarantee that when you sample the population, all those drivers will be detected (as this depends on the actual proportion of cells with the drivers and the settings of `samplePop`).

6.3.3 Fixation of genes/gene combinations

In some cases we might be interested in running simulations until a particular set of genes, or gene combinations, reaches fixation. This exit condition might be more relevant than some of the above in many non-cancer-related evolutionary genetics scenarios.

Simulations will stop as soon as any of the genes or gene combinations in the vector (or list) `fixation` reaches a frequency of 1. These gene combinations might have non-zero intersection (i.e., they might share genes), and those genes need not be drivers. If we want simulations to only stop when fixation of those genes/gene combinations is reached, we will set all other stopping conditions to `NA`. It is, of course, up to you to ensure that those stopping conditions are reasonable (that they can be reached) and to use, or not, `finalTime`; otherwise, simulations will eventually abort (e.g., when `max.wall.time` or `max.num.tries` are reached). Since we are asking for fixation, the `Exp` or `Bozic` models will often not be appropriate here; instead, models with competition such as `McFL` are more appropriate.

We return here to the example from section 5.3.

```
u <- 0.2; i <- -0.02; vi <- 0.6; ui <- uv <- -Inf
od2 <- allFitnessEffects(
    epistasis = c("u" = u,  "u:i" = ui,
                  "u:v" = uv, "i" = i,
                  "v:-i" = -Inf, "v:i" = vi))
```

Ochs and Desai explain that "Each simulated population was evolved until either the uphill genotype or valley-crossing genotype fixed." (see Ochs and Desai (2015), p.2, section "Simulations"). We will do the same here. We specify that we want to end the simulation when either the "u" or the "v, i" genotypes have reached fixation, by passing those genotype combinations as the `fixation` argument (in this example using `fixation = c("u", "v")` would have been equivalent, since the "v" genotype by itself has fitness of 0).

We want to be explicit that fixation will be the one and only condition for ending the simulations, and thus we set arguments `detectionDrivers`, `finalTime`, `detectionSize` and `detectionProb` explicitly to `NA`. (We set the number of repetitions only to 10 for the sake of speed when creating the vignette).

```
initS <- 20
## We use only a small number of repetitions for the sake
## of speed.
od100 <- oncoSimulPop(10, od2,
                      fixation = c("u", "v, i"),
                      model = "McFL",
                      mu = 1e-4,
                      detectionDrivers = NA,
                      finalTime = NA,
                      detectionSize = NA,
```

```
                               detectionProb = NA,
                               onlyCancer = TRUE,
                               initSize = initS,
                               mc.cores = 2)
```

What is the frequency of each genotype among the simulations? (or, what is the frequency of fixation of each genotype?)

```
sampledGenotypes(samplePop(od100))
##
##  Subjects by Genes matrix of 10 subjects and 3 genes.
##   Genotype Freq
## 1     i, v    5
## 2        u    5
##
##  Shannon's diversity (entropy) of sampled genotypes: 0.6931
```

Note the very large variability in reaching fixation

```
head(summary(od100)[, c(1:3, 8:9)])
##   NumClones TotalPopSize LargestClone FinalTime NumIter
## 1         3           11           11      3319  132788
## 2         4           31           31      2717  108713
## 3         4           39           39      6124  244973
## 4         3           23           23      5654  226180
## 5         4           36           36     11338  453584
## 6         3           15           15      2074   82961
```

6.4 Plotting genotype/driver abundance over time; plotting the simulated trajectories

We have seen many of these plots already, starting with Figure 2 and Figure 4 and we will see many more below, in the examples, starting with section 6.5.1 such as in figures 5 and 6. In a nutshell, what we are plotting is the information contained in the `pops.by.time` matrix, the matrix that contains the abundances of all the clones (or genotypes) at each of the sampling periods.

The functions that do the work are called `plot` and these are actually methods for objects of class "oncosimul" and "oncosimulpop". You can access the help by doing `?plot.oncosimul`, for example.

What entities are shown in the plot? You can show the trajectories of:

- numbers of drivers (e.g., 5);
- genotypes or clones (e.g., 6).

(Of course, showing "drivers" requires that you have specified certain genes as drivers.)

What types of plots are available?

- line plots;
- stacked plots;
- stream plots.

All those three are shown in both of Figure 5 and Figure 6.

If you run multiple simulations using `oncoSimulPop` you can plot the trajectories of all of the simulations.

6.5 Several examples of simulations and plotting simulation trajectories

6.5.1 Bauer's example again

We will use the model of Bauer, Siebert, and Traulsen (2014) that we saw in section 5.1.

```
K <- 5
sd <- 0.1
sdp <- 0.15
sp <- 0.05
bauer <- data.frame(parent = c("Root", rep("p", K)),
                    child = c("p", paste0("s", 1:K)),
                    s = c(sd, rep(sdp, K)),
                    sh = c(0, rep(sp, K)),
                    typeDep = "MN")
fbauer <- allFitnessEffects(bauer, drvNames = "p")
set.seed(1)
## Use fairly large mutation rate
b1 <- oncoSimulIndiv(fbauer, mu = 5e-5, initSize = 1000,
                     finalTime = NA,
                     onlyCancer = TRUE,
                     detectionProb = "default")
```

We will now use a variety of plots

```
par(mfrow = c(3, 1))
## First, drivers
plot(b1, type = "line", addtot = TRUE)
plot(b1, type = "stacked")
plot(b1, type = "stream")
```

```
par(mfrow = c(3, 1))
## Next, genotypes
plot(b1, show = "genotypes", type = "line")
```

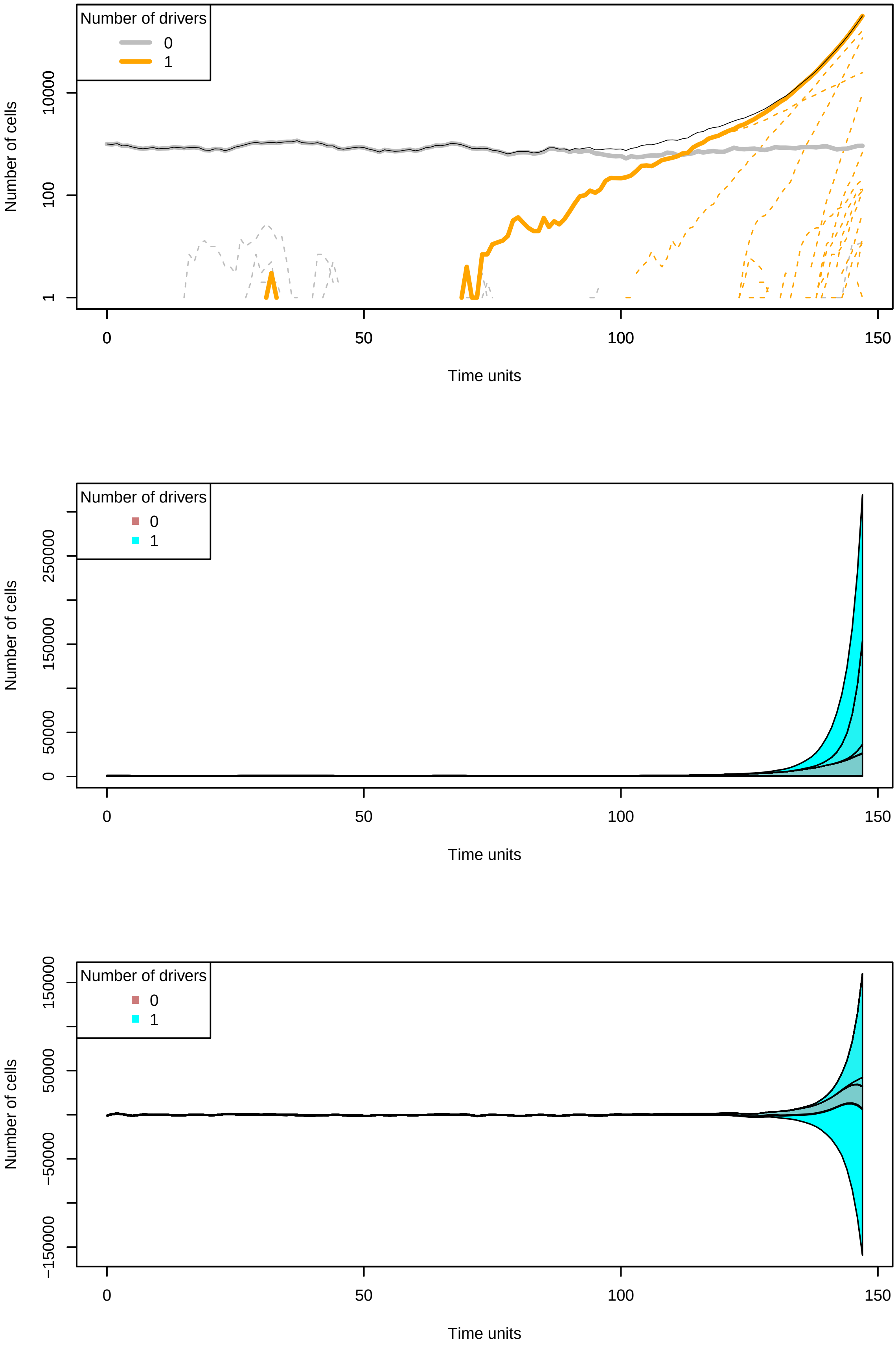

Figure 5: Three drivers' plots of a simulation of Bauer's model

```
plot(b1, show = "genotypes", type = "stacked")
plot(b1, show = "genotypes", type = "stream")
```

In this case, probably the stream plots are most helpful. Note, however, that (in contrast to some figures in the literature showing models of clonal expansion) the stream plot (or the stacked plot) does not try to explicitly show parent-descendant relationships, which would hardly be realistically possible in these plots (although the plots of phylogenies in section 8 could be of help).

6.5.2 McFarland model with 5000 passengers and 70 drivers

```
set.seed(678)
nd <- 70
np <- 5000
s <- 0.1
sp <- 1e-3
spp <- -sp/(1 + sp)
mcf1 <- allFitnessEffects(noIntGenes = c(rep(s, nd), rep(spp, np)),
                          drvNames = seq.int(nd))
mcf1s <-  oncoSimulIndiv(mcf1,
                         model = "McFL",
                         mu = 1e-7,
                         detectionProb = "default",
                         detectionSize = NA,
                         detectionDrivers = NA,
                         sampleEvery = 0.025,
                         keepEvery = 8,
                         initSize = 2000,
                         finalTime = 4000,
                         onlyCancer = FALSE)
summary(mcf1s)
##   NumClones TotalPopSize LargestClone MaxNumDrivers MaxDriversLast
## 1       618         3207         2841                3              3
##   NumDriversLargestPop TotalPresentDrivers FinalTime NumIter
## 1                    3                   5      1961   81003
##   HittedWallTime HittedMaxTries errorMF minDMratio minBMratio
## 1          FALSE          FALSE  0.0129       1585       1972
##    OccurringDrivers
## 1 5, 24, 30, 57, 67
```

```
par(mfrow  = c(2, 1))
## I use thinData to make figures smaller and faster
plot(mcf1s, addtot = TRUE, lwdClone = 0.9, log = "",
     thinData = TRUE, thinData.keep = 0.5)
plot(mcf1s, show = "drivers", type = "stacked",
```

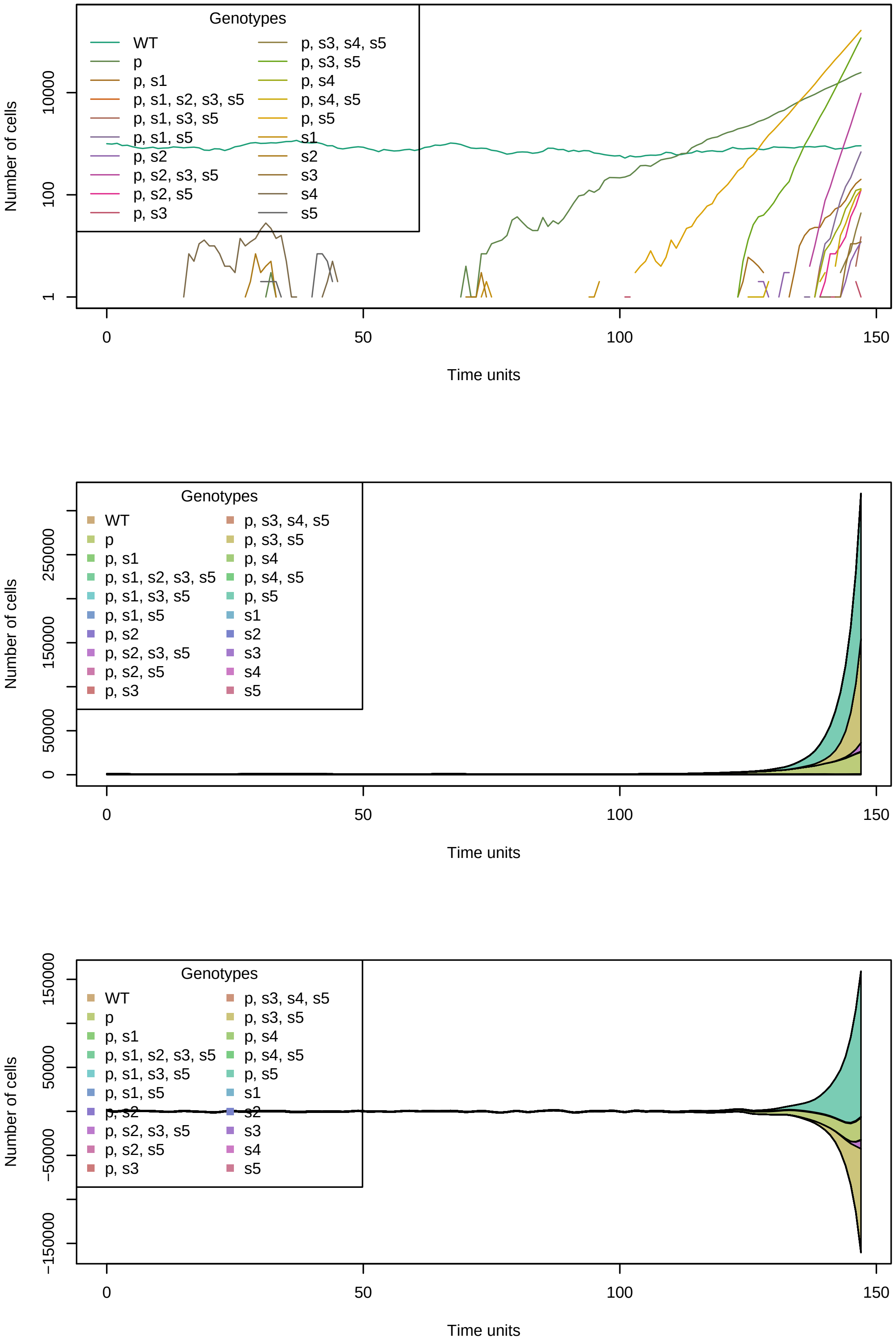

Figure 6: Three genotypes' plots of a simulation of Bauer's model

```
    thinData = TRUE, thinData.keep = 0.3,
    legend.ncols = 2)
```

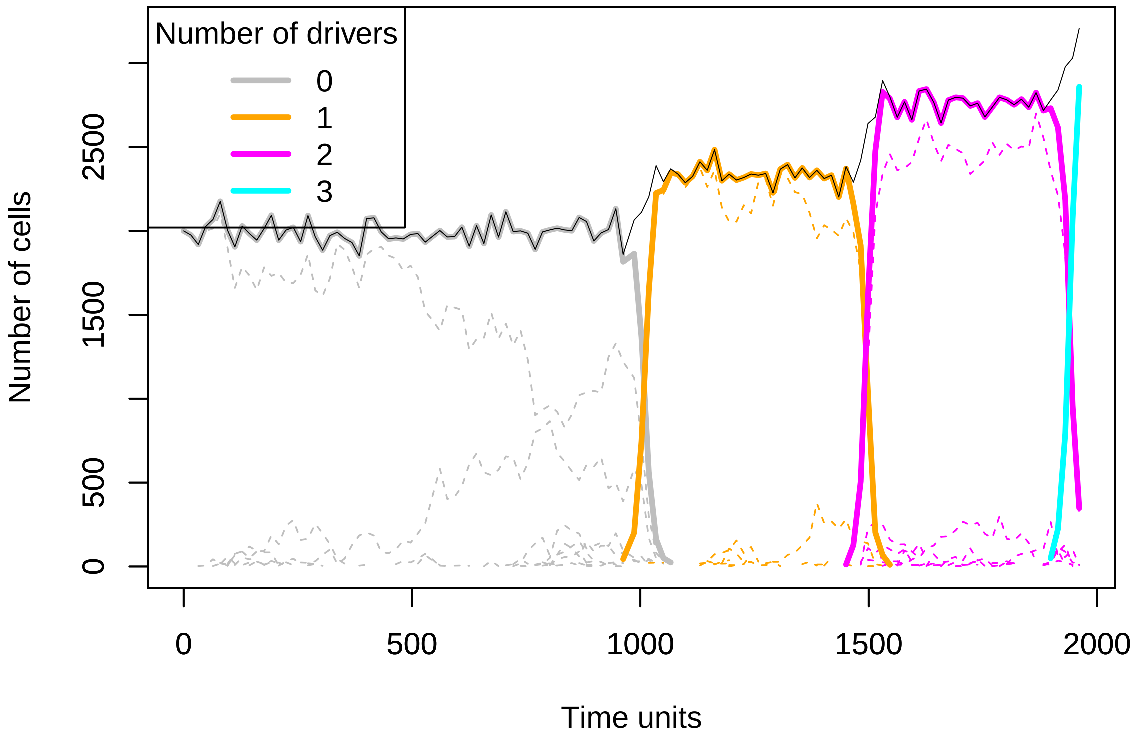

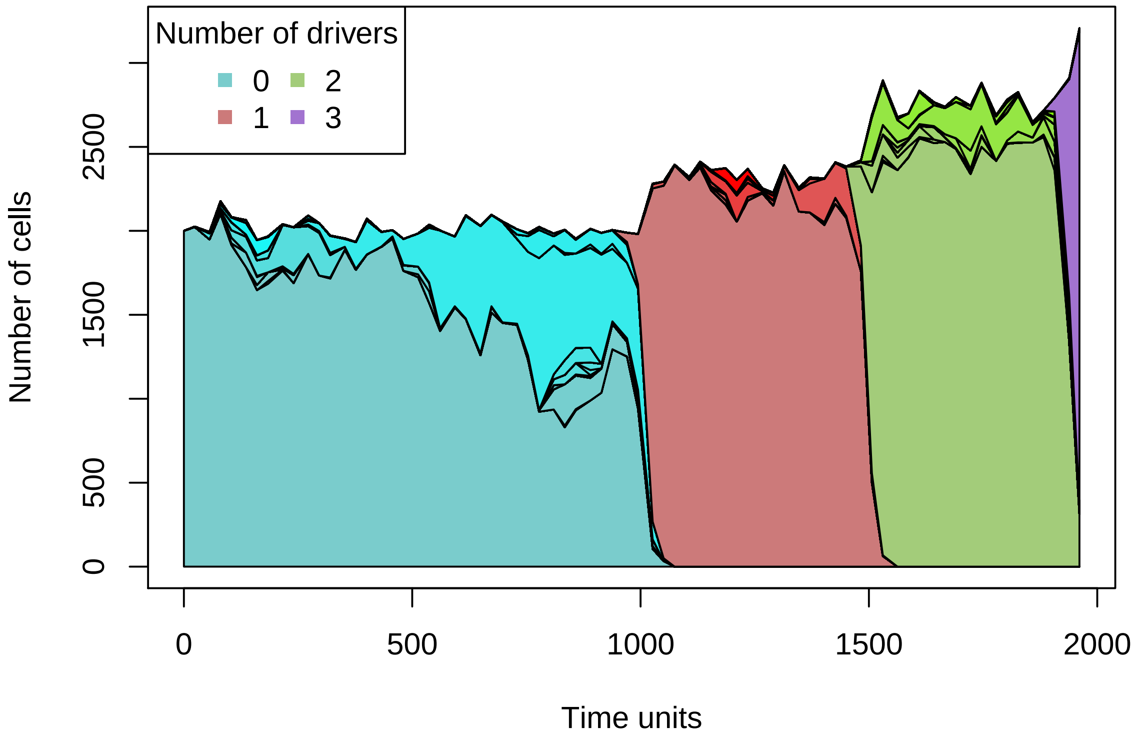

With the above output (where we see there are over 500 different genotypes) trying to represent the genotypes makes no sense.

6.5.3 McFarland model with 50,000 passengers and 70 drivers: clonal competition

The next is too slow (takes a couple of minutes in an i5 laptop) and too big to run in a vignette, because we keep track of over 4000 different clones (which leads to a result object of over 800 MB):

```r
set.seed(123)
nd <- 70
np <- 50000
s <- 0.1
sp <- 1e-4 ## as we have many more passengers
spp <- -sp/(1 + sp)
mcfL <- allFitnessEffects(noIntGenes = c(rep(s, nd), rep(spp, np)),
                          drvNames = seq.int(nd))
mcfLs <-  oncoSimulIndiv(mcfL,
                         model = "McFL",
                         mu = 1e-7,
                         detectionSize = 1e8,
                         detectionDrivers = 100,
                         sampleEvery = 0.02,
                         keepEvery = 2,
                         initSize = 1000,
                         finalTime = 2000,
                         onlyCancer = FALSE)
```

But you can access the pre-stored results and plot them (beware: this object has been trimmed by removing empty passenger rows in the Genotype matrix)

```r
data(mcfLs)
plot(mcfLs, addtot = TRUE, lwdClone = 0.9, log = "",
     thinData = TRUE, thinData.keep = 0.3,
     plotDiversity = TRUE)
```

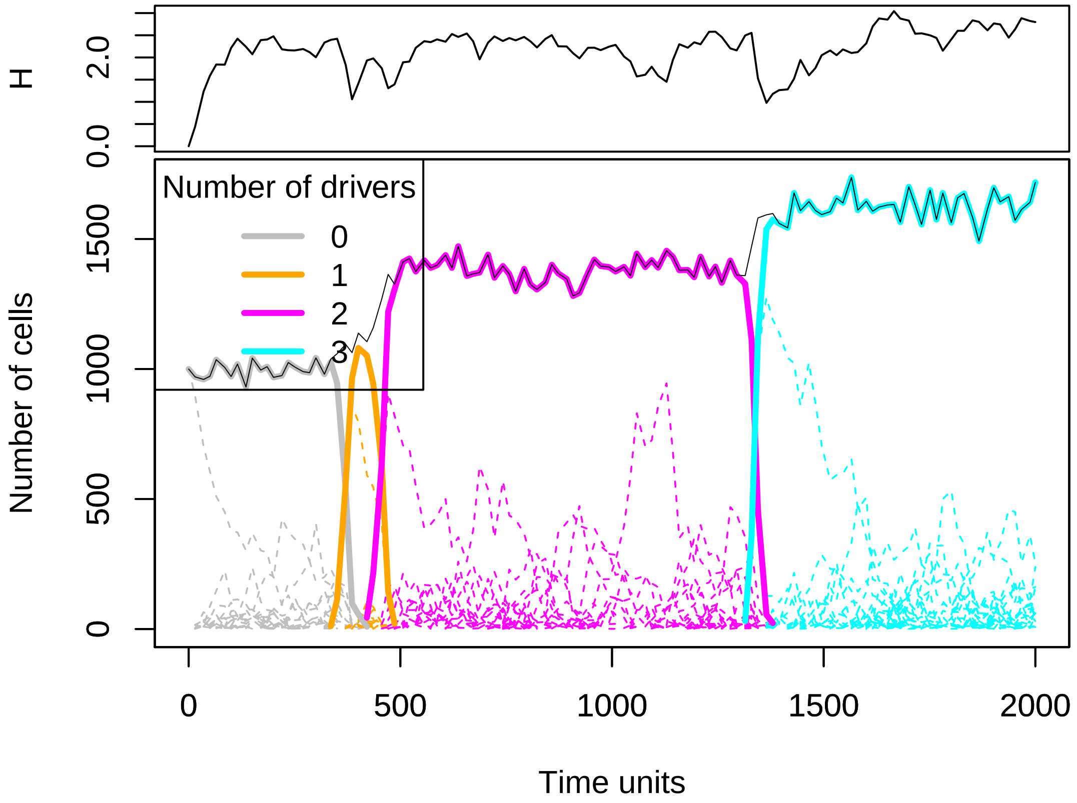

The argument `plotDiversity = TRUE` asks to show a small plot on top with Shannon's diversity index.

```
summary(mcfLs)
##   NumClones TotalPopSize LargestClone MaxNumDrivers MaxDriversLast
## 1      4458         1718          253             3              3
##   NumDriversLargestPop TotalPresentDrivers FinalTime NumIter
## 1                    3                  70      2000  113759
##   HittedWallTime HittedMaxTries errorMF minDMratio minBMratio
## 1          FALSE          FALSE 0.01922      184.1      199.6
##   OccurringDrivers
## 1   13, 38, 40, 69
## number of passengers per clone
summary(colSums(mcfLs$Genotypes[-(1:70), ]))
##    Min. 1st Qu.  Median    Mean 3rd Qu.    Max.
##    0.00    4.00    6.00    5.67    7.75   13.00
```

Note that we see clonal competition between clones with the same number of drivers (and with different drivers, of course). We will return to this (section 6.5.5).

A stacked plot might be better to show the extent of clonal competition (plotting takes some time —a stream plot reveals similar patterns and is also slower than the line plot). I will aggressively thin the data for this plot so it is faster and smaller (but we miss some of the fine grain, of course):

```
plot(mcfLs, type = "stacked", thinData = TRUE,
     thinData.keep = 0.2,
```

```
    plotDiversity = TRUE,
    xlim = c(0, 1000))
```

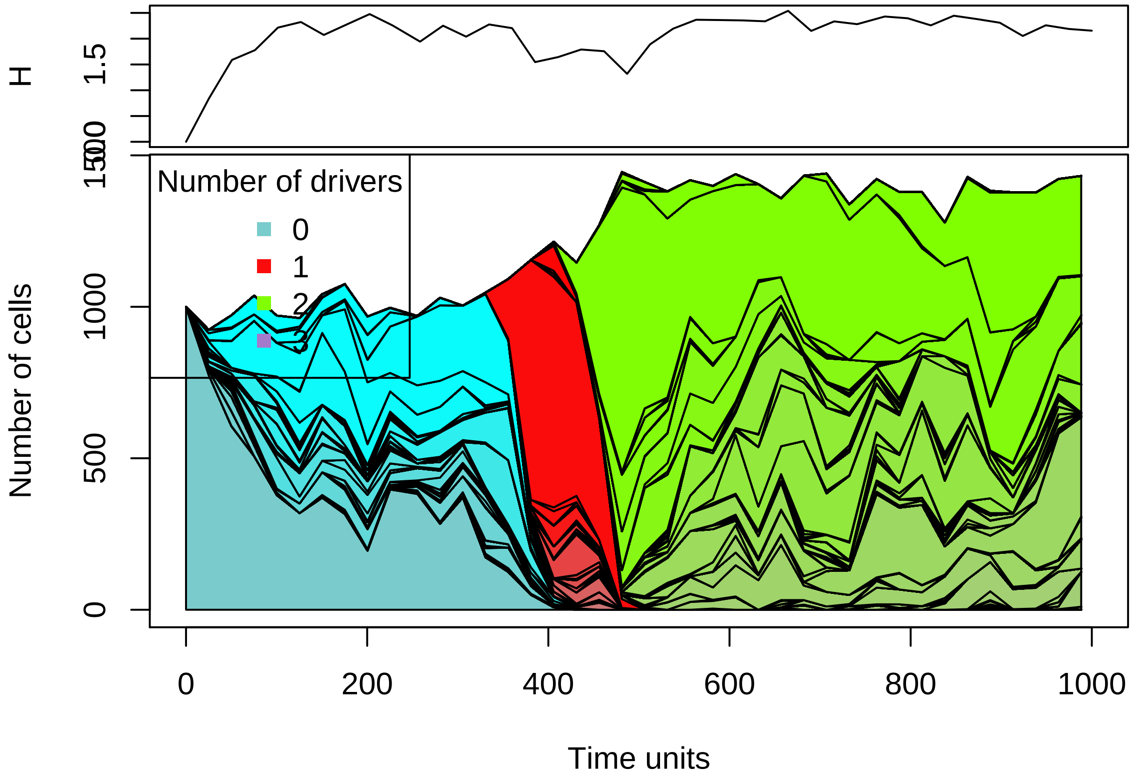

6.5.4 Simulation with a conjunction example

We will use several of the previous examples. Most of them are in file `examplesFitnessEffects`, where they are stored inside a list, with named components (names the same as in the examples above):

```
data(examplesFitnessEffects)
names(examplesFitnessEffects)
##  [1] "cbn1"    "cbn2"    "smn1"    "xor1"    "fp3"     "fp4m"    "o3"
##  [8] "ofe1"    "ofe2"    "foi1"    "sv"      "svB"     "svB1"    "sv2"
## [15] "sm1"     "e2"      "E3A"     "em"      "fea"     "fbauer"  "w"
## [22] "pancr"
```

We will simulate using the simple CBN-like restrictions of section 3.4.4 with two different models.

```
data(examplesFitnessEffects)
evalAllGenotypes(examplesFitnessEffects$cbn1, order = FALSE)[1:10, ]
##    Genotype Fitness
## 1         a    1.10
## 2         b    1.10
## 3         c    0.10
```

```
## 4         d    1.10
## 5         e    1.10
## 6         g    0.10
## 7      a, b    1.21
## 8      a, c    0.11
## 9      a, d    1.21
## 10     a, e    1.21
sm <-  oncoSimulIndiv(examplesFitnessEffects$cbn1,
                      model = "McFL",
                      mu = 5e-7,
                      detectionSize = 1e8,
                      detectionDrivers = 2,
                      detectionProb = "default",
                      sampleEvery = 0.025,
                      keepEvery = 5,
                      initSize = 2000,
                      onlyCancer = TRUE)
summary(sm)
##   NumClones TotalPopSize LargestClone MaxNumDrivers MaxDriversLast
## 1         6         2721         2005             2              2
##   NumDriversLargestPop TotalPresentDrivers FinalTime NumIter
## 1                    2                   4      2257   90282
##   HittedWallTime HittedMaxTries errorMF minDMratio minBMratio
## 1          FALSE          FALSE 0.01502     309424     333333
##   OccurringDrivers
## 1       a, b, c, e
```

```
set.seed(1234)
evalAllGenotypes(examplesFitnessEffects$cbn1, order = FALSE,
                 model = "Bozic")[1:10, ]
##    Genotype Death_rate
## 1         a       0.90
## 2         b       0.90
## 3         c       1.90
## 4         d       0.90
## 5         e       0.90
## 6         g       1.90
## 7      a, b       0.81
## 8      a, c       1.71
## 9      a, d       0.81
## 10     a, e       0.81
sb <-  oncoSimulIndiv(examplesFitnessEffects$cbn1,
                       model = "Bozic",
                       mu = 5e-6,
                      detectionProb = "default",
                       detectionSize = 1e8,
```

```
                               detectionDrivers = 4,
                               sampleEvery = 2,
                               initSize = 2000,
                               onlyCancer = TRUE)
summary(sb)
##   NumClones TotalPopSize LargestClone MaxNumDrivers MaxDriversLast
## 1         7        25636        23975             2              2
##   NumDriversLargestPop TotalPresentDrivers FinalTime NumIter
## 1                    1                   5        88      53
##   HittedWallTime HittedMaxTries errorMF minDMratio minBMratio
## 1          FALSE          FALSE      NA      33333      33333
##   OccurringDrivers
## 1    a, b, c, e, g
```

As usual, we will use several plots here.

```
## Show drivers, line plot
par(cex = 0.75, las = 1)
plot(sb,show = "drivers", type = "line", addtot = TRUE,
     plotDiversity = TRUE)
```

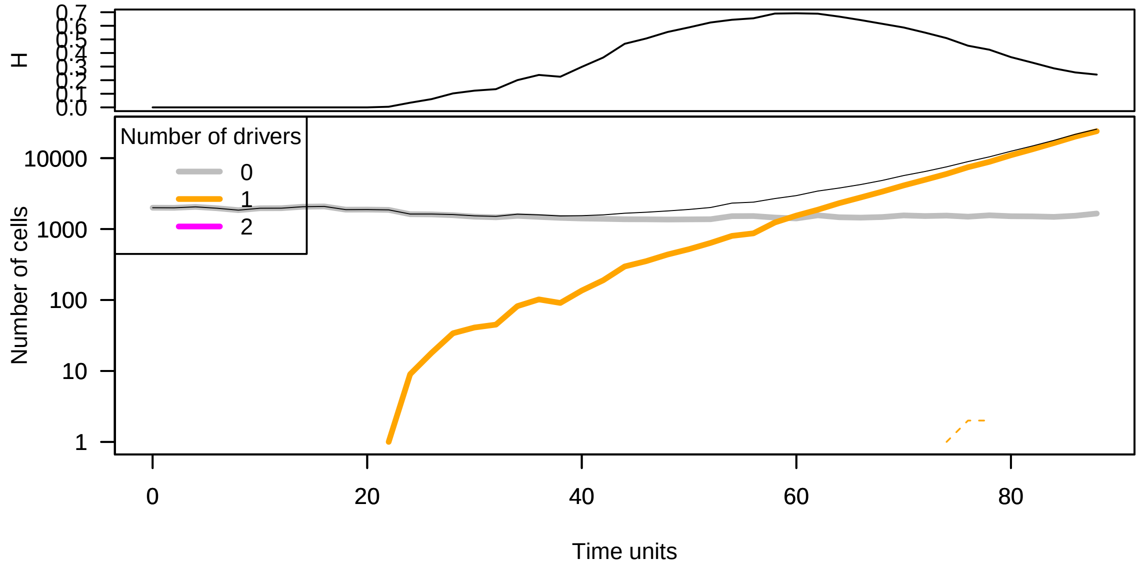


```
## Drivers, stacked
par(cex = 0.75, las = 1)
plot(sb,show = "drivers", type = "stacked", plotDiversity = TRUE)
```

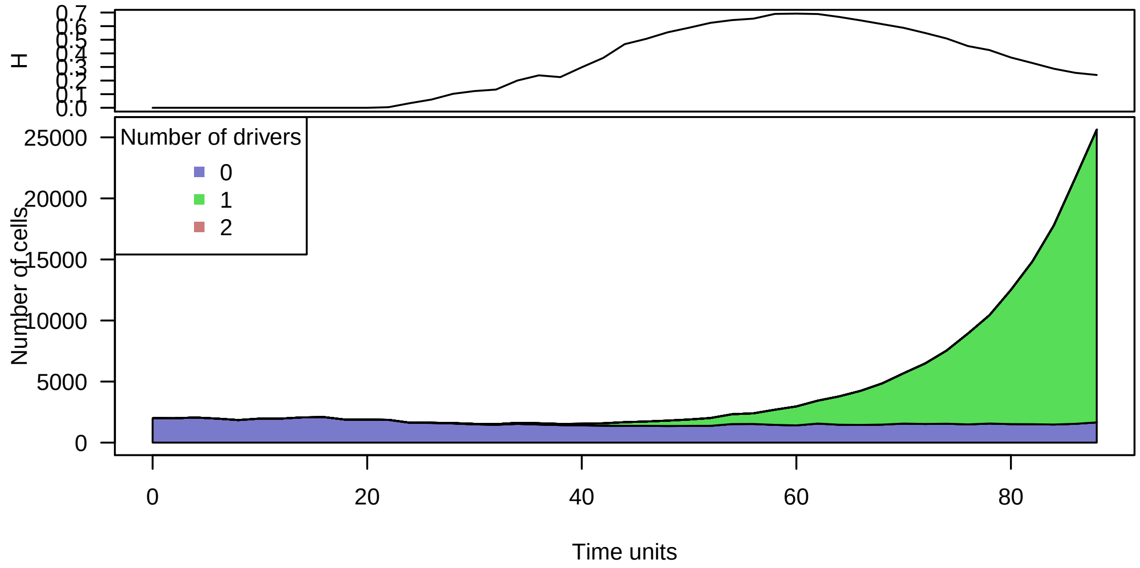


```
## Drivers, stream
par(cex = 0.75, las = 1)
plot(sb,show = "drivers", type = "stream", plotDiversity = TRUE)
```

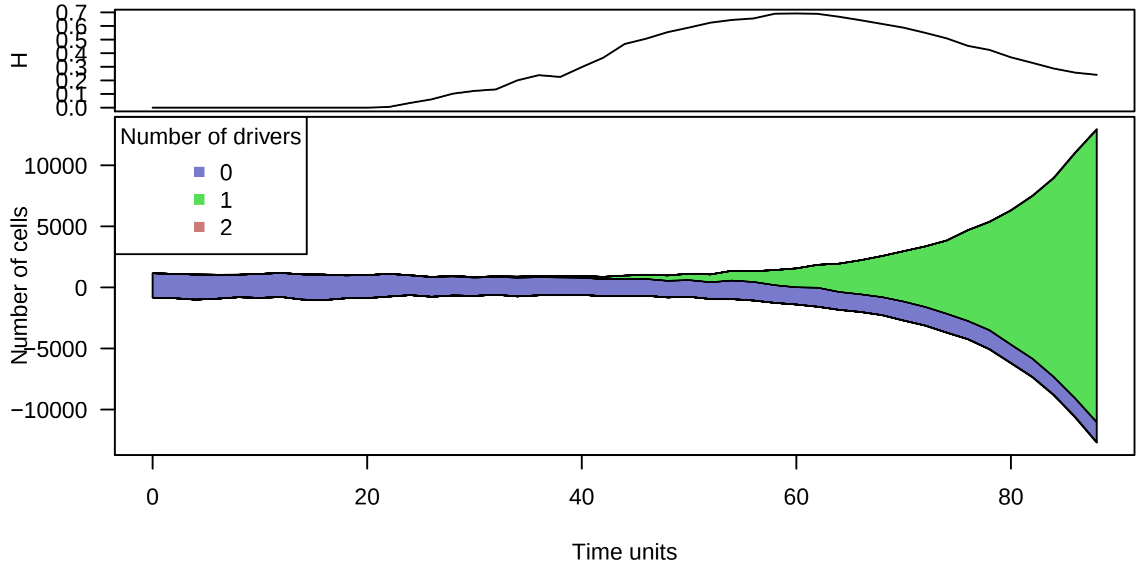


```
## Genotypes, line plot
par(cex = 0.75, las = 1)
plot(sb,show = "genotypes", type = "line", plotDiversity = TRUE)
```

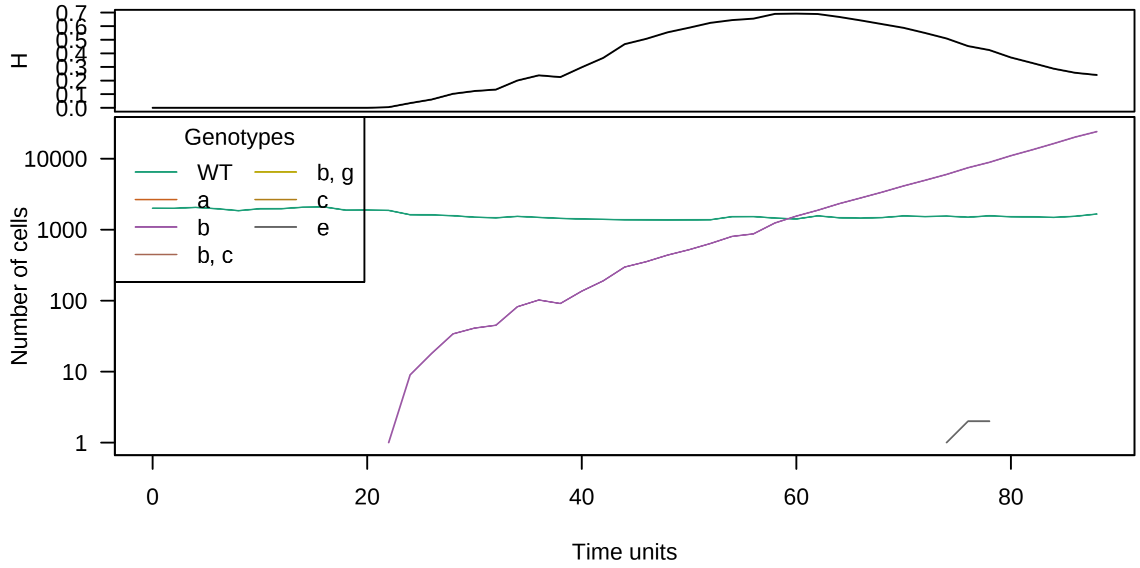


```
## Genotypes, stacked
par(cex = 0.75, las = 1)
plot(sb,show = "genotypes", type = "stacked", plotDiversity = TRUE)
```

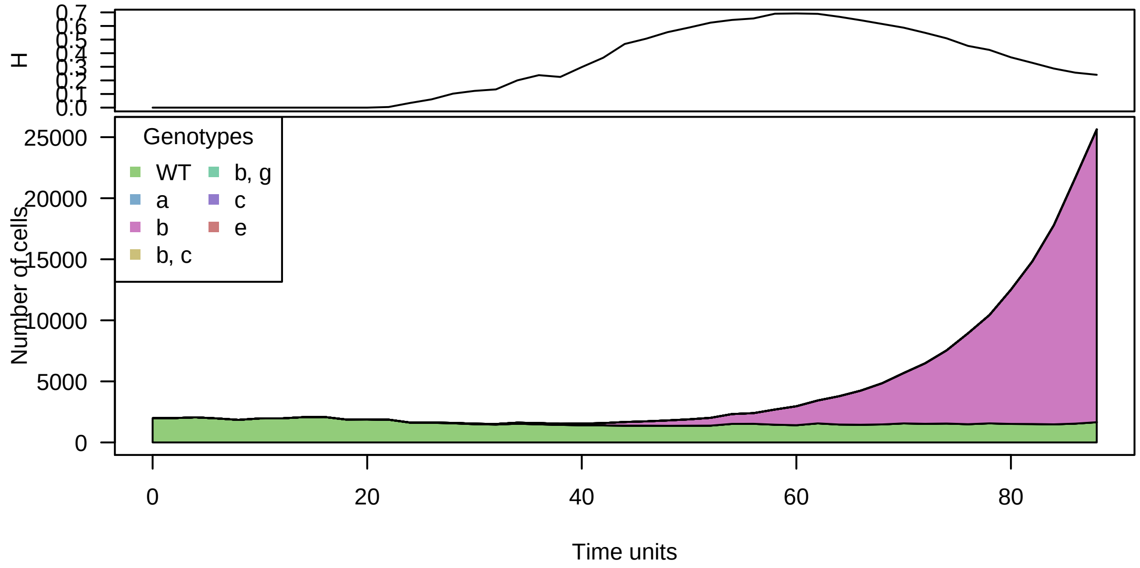


```
## Genotypes, stream
par(cex = 0.75, las = 1)
plot(sb,show = "genotypes", type = "stream", plotDiversity = TRUE)
```

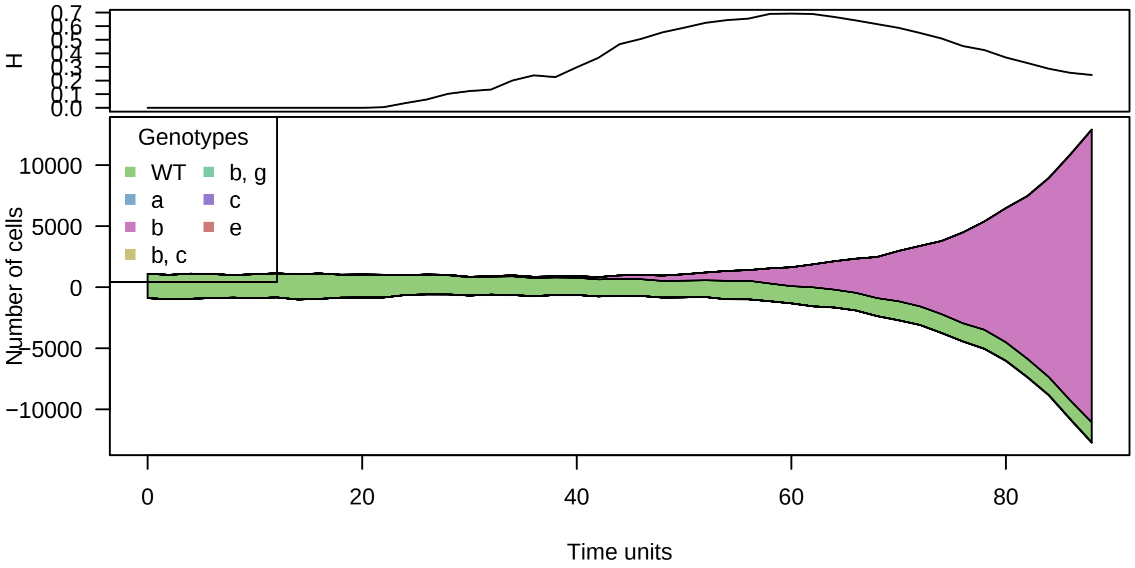

The above illustrates again that different types of plots can be useful to reveal different patterns in the data. For instance, here, because of the huge relative frequency of one of the clones/genotypes, the stacked and stream plots do not reveal the other clones/genotypes as we cannot use a log-transformed y-axis, even if there are other clones/genotypes present.

6.5.5 Simulation with order effects and McFL model

(We use a somewhat large mutation rate than usual, so that the simulation runs quickly.)

```
set.seed(4321)
tmp <-  oncoSimulIndiv(examplesFitnessEffects[["o3"]],
                        model = "McFL",
                        mu = 5e-5,
                        detectionSize = 1e8,
                        detectionDrivers = 3,
                        sampleEvery = 0.025,
                        max.num.tries = 10,
                        keepEvery = 5,
                        initSize = 2000,
                        finalTime = 6000,
                        onlyCancer = FALSE)
```

We show a stacked and a line plot of the drivers:

```
par(las = 1, cex = 0.85)
plot(tmp, addtot = TRUE, log = "", plotDiversity = TRUE,
     thinData = TRUE, thinData.keep = 0.2)
```

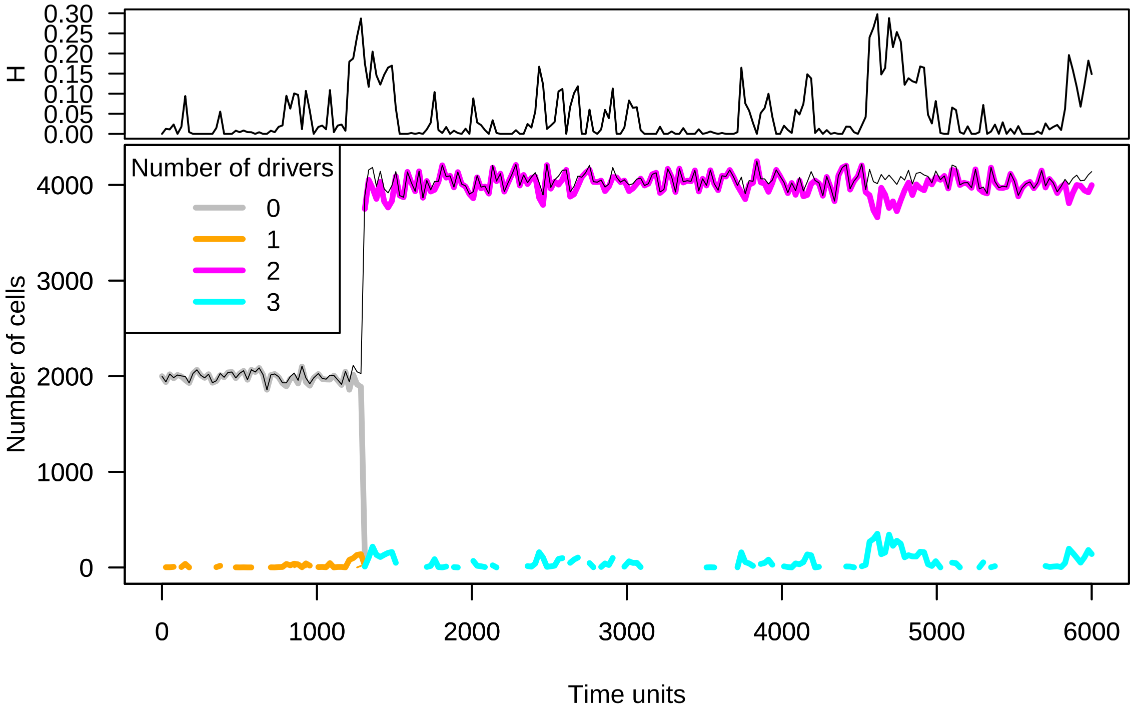


```
par(las = 1, cex = 0.85)
plot(tmp, type = "stacked", plotDiversity = TRUE,
     ylim = c(0, 5500), legend.ncols = 4,
     thinData = TRUE, thinData.keep = 0.2)
```

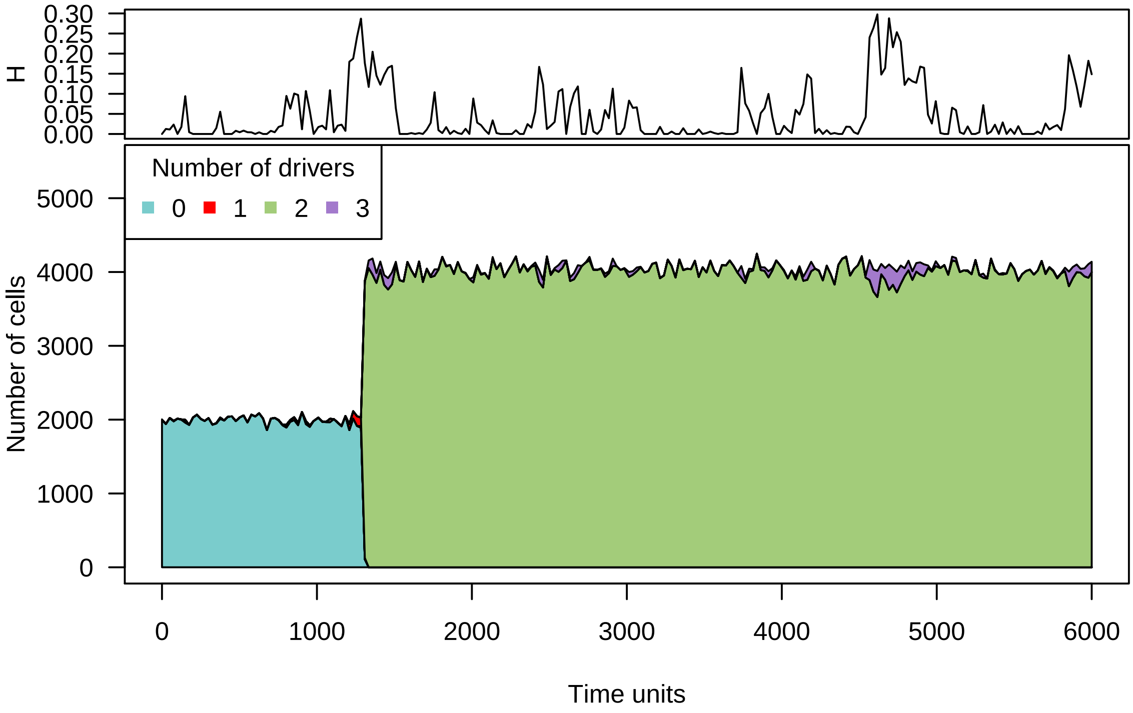

In this example (and at least under Linux, with both GCC and clang —random number streams in C++, and thus simulations, can differ between combinations of operating system and compiler), we can see that the mutants with three drivers do

not get established when we stop the simulation at time 6000. This is one case where the summary statistics about number of drivers says little of value, as fitness is very different for genotypes with the same number of mutations, and does not increase in a simple way with drivers:

```
evalAllGenotypes(examplesFitnessEffects[["o3"]], addwt = TRUE,
                 order = TRUE)
##      Genotype Fitness
## 1          WT    1.00
## 2           d    1.00
## 3           f    1.00
## 4           m    1.00
## 5       d > f    1.00
## 6       d > m    1.10
## 7       f > d    1.00
## 8       f > m    1.00
## 9       m > d    1.50
## 10      m > f    1.00
## 11 d > f > m    1.54
## 12 d > m > f    1.32
## 13 f > d > m    0.77
## 14 f > m > d    1.50
## 15 m > d > f    1.50
## 16 m > f > d    1.50
```

A few figures could help:

```
plot(tmp, show = "genotypes", ylim = c(0, 5500), legend.ncols = 3,
     thinData = TRUE, thinData.keep = 0.5)
```

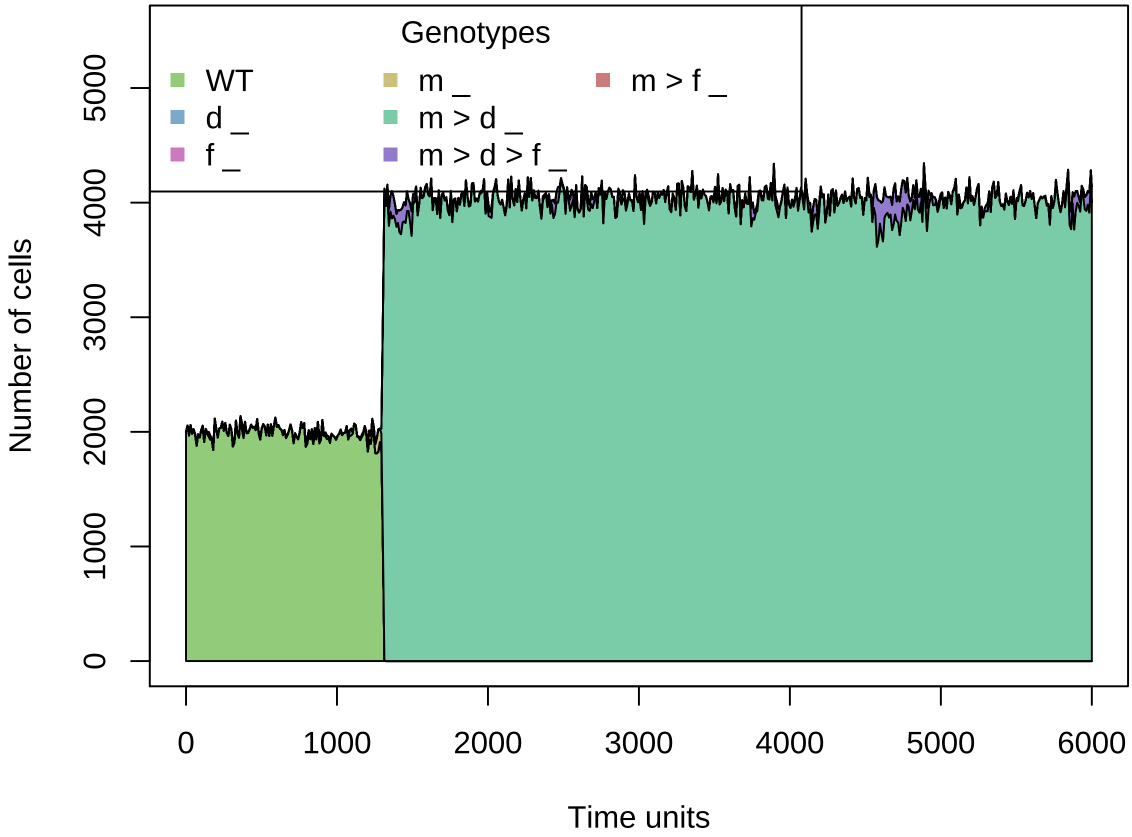

(When reading the figure legends, recall that genotype $x > y \_ z$ is one where a mutation in "x" happened before a mutation in "y", and there is also a mutation in "z" for which order does not matter. Here, there are no genes for which order does not matter and thus there is nothing after the "_").

In this case, the clones with three drivers end up displacing those with two by the time we stop; moreover, notice how those with one driver never really grow to a large population size, so we basically go from a population with clones with zero drivers to a population made of clones with two or three drivers:

```
set.seed(15)
tmp <-  oncoSimulIndiv(examplesFitnessEffects[["o3"]],
                       model = "McFL",
                       mu = 5e-5,
                       detectionSize = 1e8,
                       detectionDrivers = 3,
                       sampleEvery = 0.025,
                       max.num.tries = 10,
                       keepEvery = 5,
                       initSize = 2000,
                       finalTime = 20000,
                       onlyCancer = FALSE,
                       extraTime = 1500)
tmp
##
```

```
## Individual OncoSimul trajectory with call:
##  oncoSimulIndiv(fp = examplesFitnessEffects[["o3"]], model = "McFL",
##     mu = 5e-05, detectionSize = 1e+08, detectionDrivers = 3,
##     sampleEvery = 0.025, initSize = 2000, keepEvery = 5, extraTime = 1500,
##     finalTime = 20000, onlyCancer = FALSE, max.num.tries = 10)
##
##   NumClones TotalPopSize LargestClone MaxNumDrivers MaxDriversLast
## 1         8         4222         2758             3              3
##   NumDriversLargestPop TotalPresentDrivers FinalTime NumIter
## 1                    3                   3      6259  251948
##   HittedWallTime HittedMaxTries errorMF minDMratio minBMratio
## 1          FALSE          FALSE 0.01442       6161       6667
##   OccurringDrivers
## 1          d, f, m
##
## Final population composition:
##       Genotype    N
## 1            _    0
## 2          d _    0
## 3      d > f _    0
## 4          f _    0
## 5          m _    0
## 6      m > d _ 1464
## 7 m > d > f _ 2758
## 8      m > f _    0
```

use a drivers plot:

```
par(las = 1, cex = 0.85)
plot(tmp, addtot = TRUE, log = "", plotDiversity = TRUE,
     thinData = TRUE, thinData.keep = 0.5)
```

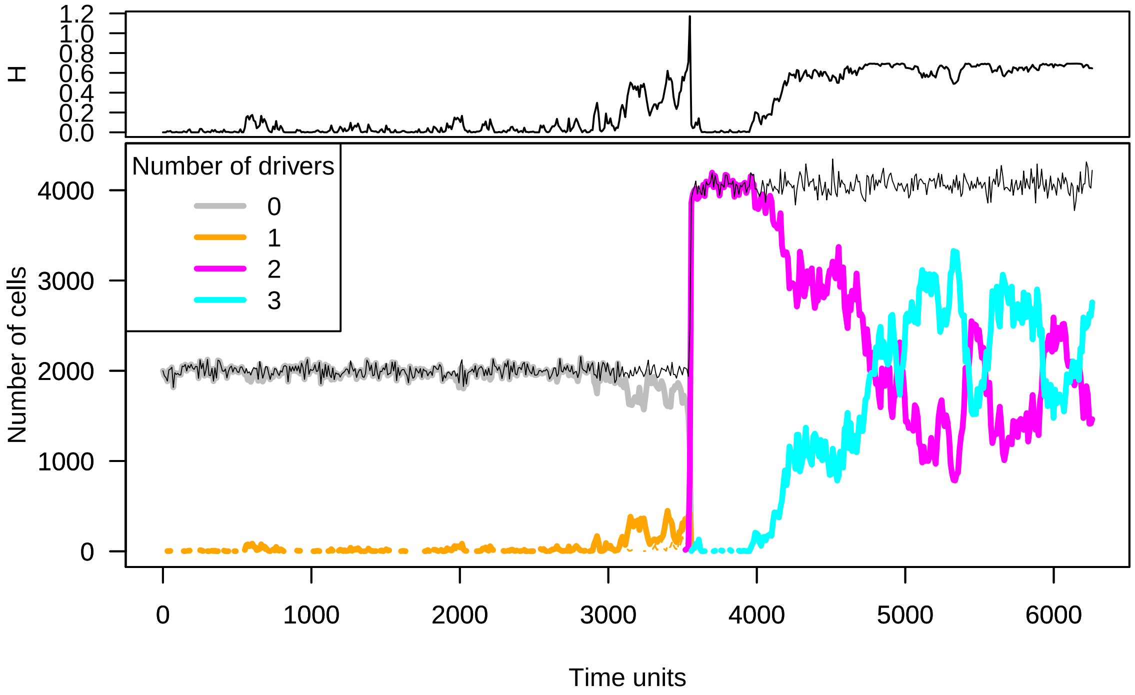


```r
par(las = 1, cex = 0.85)
plot(tmp, type = "stacked", plotDiversity = TRUE,
     legend.ncols = 4, ylim = c(0, 5200), xlim = c(3400, 5000),
     thinData = TRUE, thinData.keep = 0.5)
```

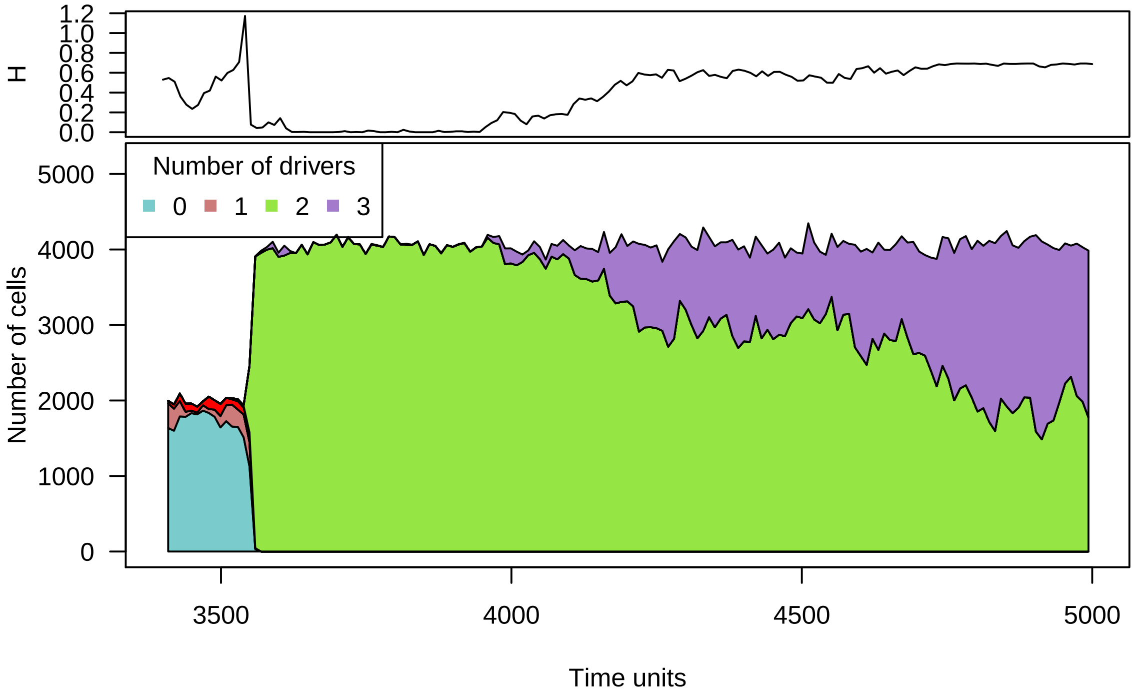

Now show the genotypes explicitly:

```r
## Improve telling apart the most abundant
## genotypes by sorting colors
## differently via breakSortColors
```

```r
## Modify ncols of legend, so it is legible by not overlapping
## with plot
par(las = 1, cex = 0.85)
plot(tmp, show = "genotypes", breakSortColors = "distave",
     plotDiversity = TRUE, legend.ncols = 4,
     ylim = c(0, 5300), xlim = c(3400, 5000),
     thinData = TRUE, thinData.keep = 0.5)
```

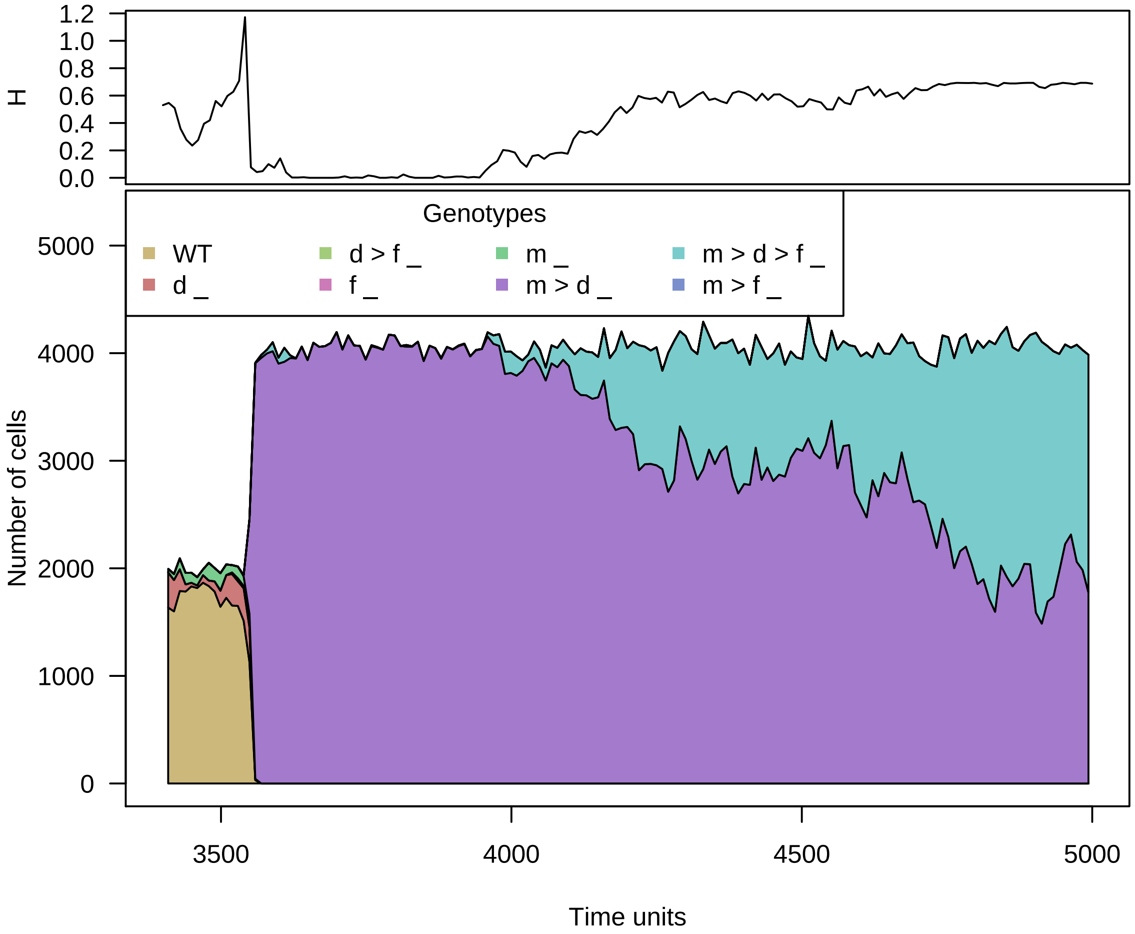

As before, the argument `plotDiversity = TRUE` asks to show a small plot on top with Shannon's diversity index. Here, as before, the quick clonal expansion of the clone with two drivers leads to a sudden drop in diversity (for a while, the population is made virtually of a single clone). Note, however, that compared to section 6.5.3, we are modeling here a scenario with very few genes, and correspondingly very few possible genotypes, and thus it is not strange that we observe very little diversity.

(We have used `extraTime` to continue the simulation well past the point of detection, here specified as three drivers. Instead of specifying `extraTime` we can set the `detectionDrivers` value to a number larger than the number of existing possible drivers, and the simulation will run until `finalTime` if `onlyCancer = FALSE`.)

6.6 Interactive graphics

It is possible to create interactive stacked area and stream plots using the *streamgraph* package, available from https://github.com/hrbrmstr/streamgraph. However, that package is not available as a CRAN or BioConductor package, and thus we cannot depend on it for this vignette (or this package). You can, however, paste the code below and make it run locally.

Before calling the `streamgraph` function, though, we need to convert the data from the original format in which it is stored into "long format". A simple convenience function is provided as `OncoSimulWide2Long` in *OncoSimulR*.

As an example, we will use the data we generated above for section 6.5.1.

```
## Convert the data
lb1 <- OncoSimulWide2Long(b1)

## Install the streamgraph package from GitHub and load
library(devtools)
devtools::install_github("hrbrmstr/streamgraph")
library(streamgraph)

## Stream plot for Genotypes
sg_legend(streamgraph(lb1, Genotype, Y, Time, scale = "continuous"),
              show=TRUE, label="Genotype: ")

## Staked area plot and we use the pipe
streamgraph(lb1, Genotype, Y, Time, scale = "continuous",
            offset = "zero") %>%
    sg_legend(show=TRUE, label="Genotype: ")
```

7 Sampling multiple simulations

Often, you will want to simulate multiple runs of the same scenario, and then obtain the matrix of runs by mutations (a matrix of individuals/samples by genes or, equivalently, a vector of “genotypes”), and do something with them. OncoSimulR offers several ways of doing this.

The key function here is `samplePop`, either called explicitly after `oncoSimulPop` (or `oncoSimulIndiv`), or implicitly as part of a call to `oncoSimulSample`. With `samplePop` you can use **single cell** or **whole tumor** sampling (for details see the help of `samplePop`). Depending on how the simulations were conducted, you might also sample at different times, or as a function of population sizes. A major difference between procedures has to do with whether or not you want to keep the complete history of the simulations.

You want to keep the complete history of population sizes of clones during the simulations. You will simulate using:

- `oncoSimulIndiv` repeatedly (maybe within `mclapply`, to parallelize the run).
- `oncoSimulPop`. `oncoSimulPop` is basically a thin wrapper around `oncoSimulIndiv` that uses `mclapply`.

In both cases, you specify the conditions for ending the simulations (as explained in 6.3). Then, you use function `samplePop` to obtain the matrix of samples by mutations.

You do not want to keep the complete history of population sizes of clones during the simulations. You will simulate using:

- `oncoSimulIndiv` repeatedly, with argument `keepEvery = NA`.
- `oncoSimulPop`, with argument `keepEvery = NA`.

  In both cases you specify the conditions for ending the simulations (as explained in 6.3). Then, you use function `samplePop`.
- `oncoSimulSample`, specifying the conditions for ending the simulations (as explained in 6.3). In this case, you will not use `samplePop`, as that is implicitly called by `oncoSimulSample`. The output is directly the matrix (and a little bit of summary from each run), and during the simulation it only stores one time point.

Why the difference between the above cases? If you keep the complete history of population sizes, you can take samples at any of the times between the beginning and the end of the simulations. If you do not keep the history, you can only sample at the time the simulation exited (see section 12.2). Why would you want to use the second route? If we are only interested in the final matrix of individuals by mutations, keeping the complete history above is wasteful because we store fully all of the simulations (for example in the call to `oncoSimulPop`) and then sample (in the call to `samplePop`).

Further criteria to use when choosing between sampling procedures is whether you need `detectionSize` and `detectionDrivers` do differ between simulations: if you use `oncoSimulPop` the arguments for `detectionSize` and `detectionDrivers` must be the same for all simulations but this is not the case for `oncoSimulSample`. See further comments in 7.2. Finally, parallelized execution is available for `oncoSimulPop` but, by design, not for `oncoSimulSample`.

The following are a few examples. First we run `oncoSimulPop` to obtain 4 simulations and in the last line we sample from them:

```
pancrPop <- oncoSimulPop(4, pancr,
                         detectionSize = 1e7,
                         keepEvery = 10,
                         mc.cores = 2)

summary(pancrPop)
##   NumClones TotalPopSize LargestClone MaxNumDrivers MaxDriversLast
## 1        16     10130909      8277602             0              0
## 2        10     10179827      9982470             0              0
## 3        11     10671391     10528644             0              0
## 4        14     10759927      9050270             0              0
##   NumDriversLargestPop TotalPresentDrivers FinalTime NumIter
## 1                    0                   0      1754    2376
## 2                    0                   0      1009    1683
## 3                    0                   0       564    1289
## 4                    0                   0      1048    1693
##   HittedWallTime HittedMaxTries errorMF minDMratio minBMratio
## 1          FALSE          FALSE      NA     142857     142857
## 2          FALSE          FALSE      NA     142857     142857
## 3          FALSE          FALSE      NA     142857     142857
## 4          FALSE          FALSE      NA     142857     142857
##   OccurringDrivers
## 1
## 2
## 3
## 4
samplePop(pancrPop)
##
##  Subjects by Genes matrix of 4 subjects and 7 genes.
##      CDNK2A KRAS MLL3 PXDN SMAD4 TGFBR2 TP53
## [1,]      0    1    0    0     0      0    0
## [2,]      0    1    0    0     0      0    0
## [3,]      0    1    0    0     0      0    0
## [4,]      0    1    0    0     0      0    0
```

Now a simple multiple call to `oncoSimulIndiv` wrapped inside `mclapply`; this is basically the same we just did above. We set the class of the object to allow direct

usage of `samplePop`. (Note: in Windows `mc.cores > 1` is not supported, so for the vignette to run in Windows, Linux, and Mac we explicitly set it here in the call to `mclapply`. For regular usage, you will not need to do this; just use whatever is appropriate for your operating system and number of cores. As well, we do not need any of this with `oncoSimulPop` because the code inside `oncoSimulPop` already takes care of setting `mc.cores` to 1 in Windows).

```
library(parallel)

if(.Platform$OS.type == "windows") {
    mc.cores <- 1
} else {
    mc.cores <- 2
}

p2 <- mclapply(1:4, function(x) oncoSimulIndiv(pancr,
                                               detectionSize = 1e7,
                                               keepEvery = 10),
                                               mc.cores = mc.cores)
class(p2) <- "oncosimulpop"
samplePop(p2)
##
##  Subjects by Genes matrix of 4 subjects and 7 genes.
##      CDNK2A KRAS MLL3 PXDN SMAD4 TGFBR2 TP53
## [1,]      1    1    0    0     0      0    0
## [2,]      0    1    0    0     0      0    0
## [3,]      0    1    0    0     0      0    0
## [4,]      0    1    0    0     0      0    0
```

Above, we have kept the complete history of the simulations as you can check by doing, for instance

```
tail(pancrPop[[1]]$pops.by.time)
##         [,1] [,2] [,3]    [,4] [,5] [,6] [,7] [,8] [,9]   [,10]
## [172,] 1710 2742    0     229    0    0    0    0    0  102031
## [173,] 1720 2643    0    1620    0    0    0    0    0  274348
## [174,] 1730 2568    0   11814    0    0    0    0    0  747110
## [175,] 1740 2521    0   95879    0    0    0    0    0 2037091
## [176,] 1750 2201    0  786377   54    1   10    2    5 5544042
## [177,] 1754 2193    0 1827735  168    3   17    2   48 8277602
##         [,11] [,12] [,13] [,14] [,15] [,16] [,17]
## [172,]      0     0     0     2     0     0     0
## [173,]      0     1     0     0     1    12     0
## [174,]      2     0     0    20     2    85     0
## [175,]    327     2     0   170     0   604     0
## [176,]   3398     2     0  1153     5  5669     0
## [177,]   8268    12     1  2426    10 12422     2
```

If we were not interested in the complete history of simulations we could have done instead (note the argument `keepEvery = NA`)

```
pancrPopNH <- oncoSimulPop(4, pancr,
                           detectionSize = 1e7,
                           keepEvery = NA,
                           mc.cores = 2)

summary(pancrPopNH)
##   NumClones TotalPopSize LargestClone MaxNumDrivers MaxDriversLast
## 1        13     10007213      8539916             0              0
## 2         9     10811718     10694739             0              0
## 3        13     10624537      7257362             0              0
## 4         8     10157712     10046377             0              0
##   NumDriversLargestPop TotalPresentDrivers FinalTime NumIter
## 1                    0                   0      2025    2651
## 2                    0                   0       421    1128
## 3                    0                   0      2041    2597
## 4                    0                   0       666    1327
##   HittedWallTime HittedMaxTries errorMF minDMratio minBMratio
## 1          FALSE          FALSE      NA     142857     142857
## 2          FALSE          FALSE      NA     142857     142857
## 3          FALSE          FALSE      NA     142857     142857
## 4          FALSE          FALSE      NA     142857     142857
##   OccurringDrivers
## 1
## 2
## 3
## 4
samplePop(pancrPopNH)
##
##  Subjects by Genes matrix of 4 subjects and 7 genes.
##      CDNK2A KRAS MLL3 PXDN SMAD4 TGFBR2 TP53
## [1,]      0    1    0    0     0      0    0
## [2,]      0    1    0    0     0      0    0
## [3,]      0    1    0    0     0      0    0
## [4,]      0    1    0    0     0      0    0
```

which only keeps the very last sample:

```
pancrPopNH[[1]]$pops.by.time
##      [,1] [,2]  [,3] [,4]    [,5]    [,6] [,7] [,8] [,9] [,10]
## [1,] 2025 4350 19398  172 8539916 1433826    4   17    1   105
##      [,11] [,12] [,13] [,14]
## [1,]     7  8060     6  1351
```

Or we could have used `oncoSimulSample`:

```
pancrSamp <- oncoSimulSample(4, pancr)
## Successfully sampled 4 individuals
##
##  Subjects by Genes matrix of 4 subjects and 7 genes.
pancrSamp$popSamp
##      CDNK2A KRAS MLL3 PXDN SMAD4 TGFBR2 TP53
## [1,]      0    1    0    0     0      0    0
## [2,]      0    1    0    0     0      0    0
## [3,]      0    1    0    0     0      0    0
## [4,]      0    1    0    0     0      0    0
```

Again, why the above differences? If we are only interested in the final matrix of populations by mutations, keeping the complete history the above is wasteful, because we store fully all of the simulations (in the call to `oncoSimulPop`) and then sample (in the call to `samplePop`).

7.1 Whole-tumor and single-cell sampling, and do we always want to sample?

`samplePop` is designed to emulate the process of obtaining a sample from a (set of) "patient(s)". But there is no need to sample. The history of the population, with a granularity that is controlled by argument `keepEvery`, is kept in the matrix `pops.by.time` which contains the number of cells of every clone at every sampling point (see further details in 12.2). This is the information used in the plots that show the trajectory of a simulation: the plots that show the change in genotype or driver abundance over time (see section 6.4 and examples mentioned there).

Regardless of whether and how you plot the information in `pops.by.time`, you can also sample one or multiple simulations using `samplePop`. In **whole-tumor** sampling the resolution is the whole tumor (or the whole population). Thus, a key argument is `thresholdWhole`, the threshold for detecting a mutation: a gene is considered mutated if it is altered in at least "thresholdWhole" proportion of the cells in that simulation (at a particular time point). This of course means that your "sampled genotype" might not correspond to any existing genotype because we are summing over all cells in the population. For instance, suppose that at the time we take the sample there are only two clones in the population, one clone with a frequency of 0.4 that has gene A mutated, and a second clone one with a frequency of 0.6 that has gene B mutated. If you set `thresholdWhole` to values ≤ 0.4 the sampled genotype will show both A and B mutated. **Single-cell** sampling is provided as an option in contrast to whole-tumor sampling. Here any sampled genotype will correspond to an existing genotype as you are sampling with single-cell resolution.

When `samplePop` is run on a set of simulated data of, say, 100 simulated trajectories (100 "subjects"), it will produce a matrix with 100 rows (100 "subjects"). But if it makes sense in the context of your problem (e.g., multiple samples per patient?) you can of course run `samplePop` repeatedly.

7.2 Differences between "samplePop" and "oncoSimulSample"

samplePop provides two sampling times: "last" and "uniform". It also allows you to sample at the first sample time(s) at which the population(s) reaches a given size, which can be either the same or different for each simulation (with argument popSizeSample). "last" means to sample each individual in the very last time period of the simulation. "uniform" means sampling each individual at a time chosen uniformly from all the times recorded in the simulation between the time when the first driver appeared and the final time period. "unif" means that it is almost sure that different individuals will be sampled at different times. "last" does not guarantee that different individuals will be sampled at the same time unit, only that all will be sampled in the last time unit of their simulation.

With oncoSimulSample we obtain samples that correspond to timeSample = "last" in samplePop by specifying a unique value for detectionSize and detectionDrivers. The data from each simulation will correspond to the time point at which those are reached (analogous to timeSample = "last"). How about uniform sampling? We pass a vector of detectionSize and detectionDrivers, where each value of the vector comes from a uniform distribution. This is not identical to the "uniform" sampling of oncoSimulSample, as we are not sampling uniformly over all time periods, but are stopping at uniformly distributed values over the stopping conditions. Arguably, however, the procedure in samplePop might be closer to what we mean with "uniformly sampled over the course of the disease" if that course is measured in terms of drivers or size of tumor.

An advantage of oncoSimulSample is that we can specify arbitrary sampling schemes, just by passing the appropriate vector detectionSize and detectionDrivers. A disadvantage is that if we change the stopping conditions we can not just resample the data, but we need to run it again.

There is no difference between oncoSimulSample and oncoSimulPop + samplePop in terms of the typeSample argument (whole tumor or single cell).

Finally, there are some additional differences between the two functions. oncoSimulPop can run parallelized (it uses mclapply). This is not done with oncoSimulSample because this function is designed for simulation experiments where you want to examine many different scenarios simultaneously. Thus, we provide additional stopping criteria (max.wall.time.total and max.num.tries.total) to determine whether to continue running the simulations, that bounds the total running time of all the simulations in a call to oncoSimulSample. And, if you are running multiple different scenarios, you might want to make multiple, separate, independent calls (e.g., from different R processes) to oncoSimulSample, instead of relying in mclapply, since this is likely to lead to better usage of multiple cores/CPUs if you are examining a large number of different scenarios.

8 Showing the genealogical relationships of clones

If you run simulations with `keepPhylog = TRUE`, the simulations keep track of when every clone is generated, and that will allow us to see the parent-child relationships between clones. (This is disabled by default).

Let us re-run a previous example:

```
set.seed(15)
tmp <-  oncoSimulIndiv(examplesFitnessEffects[["o3"]],
                       model = "McFL",
                       mu = 5e-5,
                       detectionSize = 1e8,
                       detectionDrivers = 3,
                       sampleEvery = 0.025,
                       max.num.tries = 10,
                       keepEvery = 5,
                       initSize = 2000,
                       finalTime = 20000,
                       onlyCancer = FALSE,
                       extraTime = 1500,
                       keepPhylog = TRUE)
tmp
##
## Individual OncoSimul trajectory with call:
##  oncoSimulIndiv(fp = examplesFitnessEffects[["o3"]], model = "McFL",
##     mu = 5e-05, detectionSize = 1e+08, detectionDrivers = 3,
##     sampleEvery = 0.025, initSize = 2000, keepEvery = 5, extraTime = 1500,
##     finalTime = 20000, onlyCancer = FALSE, keepPhylog = TRUE,
##     max.num.tries = 10)
##
##   NumClones TotalPopSize LargestClone MaxNumDrivers MaxDriversLast
## 1         8         4222         2758             3              3
##   NumDriversLargestPop TotalPresentDrivers FinalTime NumIter
## 1                    3                   3      6259  251948
##   HittedWallTime HittedMaxTries errorMF minDMratio minBMratio
## 1          FALSE          FALSE 0.01442       6161       6667
##   OccurringDrivers
## 1          d, f, m
##
## Final population composition:
##       Genotype    N
## 1            _    0
## 2          d _    0
## 3      d > f _    0
## 4          f _    0
```

```
## 5         m _     0
## 6     m > d _  1464
## 7 m > d > f _  2758
## 8     m > f _     0
```

We can plot the parent-child relationships[13] of every clone ever created (with fitness larger than 0 —clones without viability are never shown):

```
plotClonePhylog(tmp, N = 0)
```

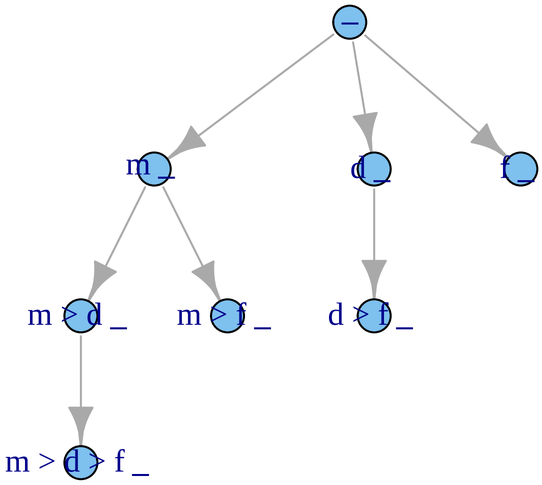

However, we often only want to show clones that exist (have number of cells > 0) at a certain time (while of course showing all of their ancestors, even if those are now extinct —i.e., regardless of their current numbers).

```
plotClonePhylog(tmp, N = 1)
```

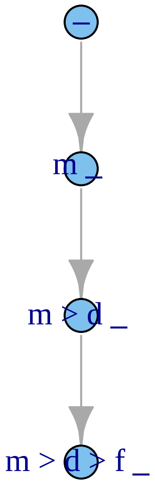

If we set `keepEvents = TRUE` the arrows show how many times each clone appeared:

[13]There are several packages in R devoted to phylogenetic inference and related issues. For instance, *ape*. I have not used that infrastructure because of our very specific needs and circumstances; for instance, internal nodes are observed, we can have networks instead of trees, and we have no uncertainty about when events occurred.

(The next can take a while)

```r
plotClonePhylog(tmp, N = 1, keepEvents = TRUE)
```

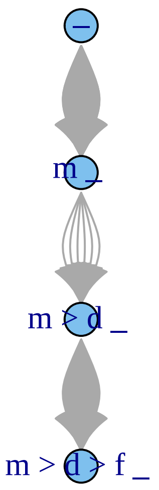

And we can show the plot so that the vertical axis is proportional to time (though you might see overlap of nodes if a child node appeared shortly after the parent):

```r
plotClonePhylog(tmp, N = 1, timeEvents = TRUE)
```

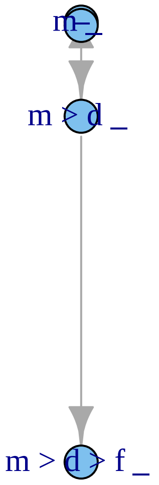

We can obtain the adjacency matrix doing

```r
get.adjacency(plotClonePhylog(tmp, N = 1, returnGraph = TRUE))
## 4 x 4 sparse Matrix of class "dgCMatrix"
##                _  m _  m > d _  m > d > f _
##   _            .  1    .        .
## m _            .  .    1        .
## m > d _        .  .    .        1
## m > d > f _    .  .    .        .
```

We can see another example here:

```r
set.seed(456)
```

```r
mcf1s <-  oncoSimulIndiv(mcf1,
                         model = "McFL",
                         mu = 1e-7,
                         detectionSize = 1e8,
                         detectionDrivers = 100,
                         sampleEvery = 0.025,
                         keepEvery = 2,
                         initSize = 2000,
                         finalTime = 1000,
                         onlyCancer = FALSE,
                         keepPhylog = TRUE)
```

Showing only clones that exist at the end of the simulation (and all their parents):

```r
plotClonePhylog(mcf1s, N = 1)
```

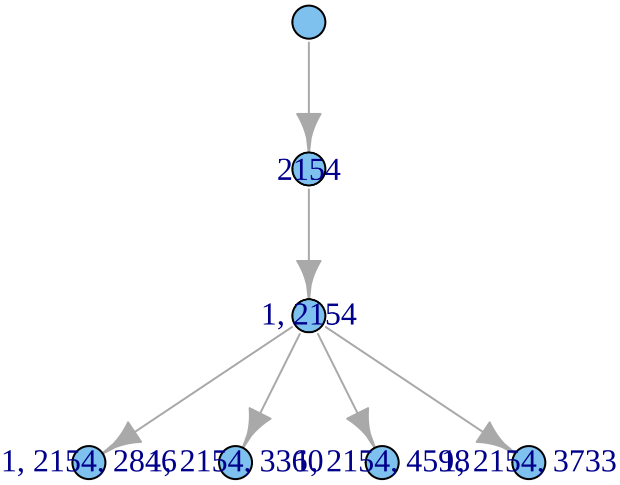

Notice that the labels here do not have a “_“, since there were no order effects in fitness. However, the labels show the genes that are mutated, just as before.

Similar, but with vertical axis proportional to time:

```r
par(cex = 0.7)
plotClonePhylog(mcf1s, N = 1, timeEvents = TRUE)
```

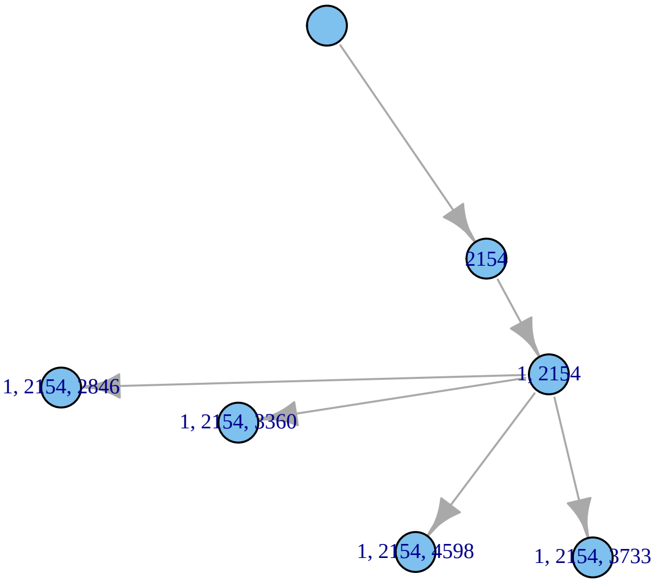

What about those that existed in the last 200 time units?

```r
par(cex = 0.7)
plotClonePhylog(mcf1s, N = 1, t = c(800, 1000))
```

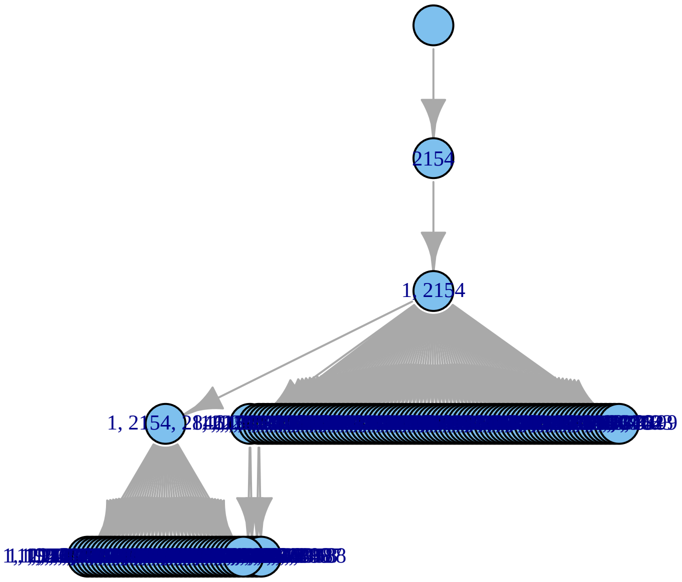

And try now to show also when the clones appeared (we restrict the time to between 900 and 1000, to avoid too much clutter):

```r
par(cex = 0.7)
plotClonePhylog(mcf1s, N = 1, t = c(900, 1000), timeEvents = TRUE)
```

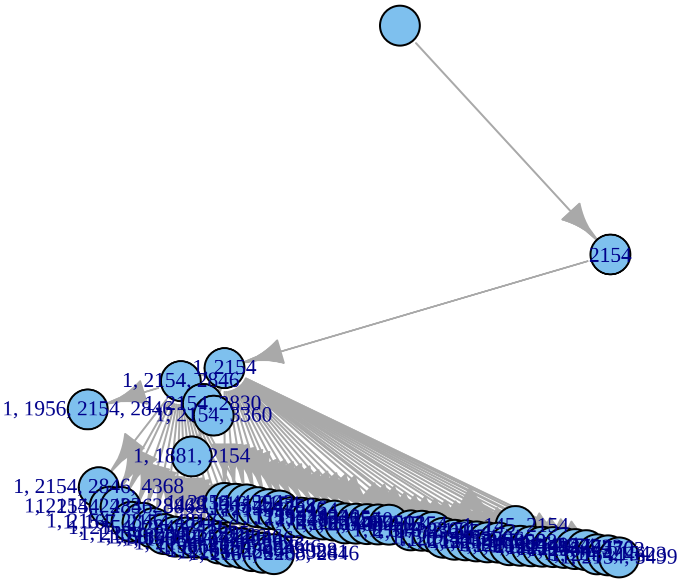

(By playing with `t`, it should be possible to obtain animations of the phylogeny. We will not pursue it here.)

If the previous graph seems cluttered, we can represent it in a different way by calling *igraph* directly after storing the graph and using the default layout:

```
g1 <- plotClonePhylog(mcf1s, N = 1, t = c(900, 1000),
                      returnGraph = TRUE)
```

```
plot(g1)
```

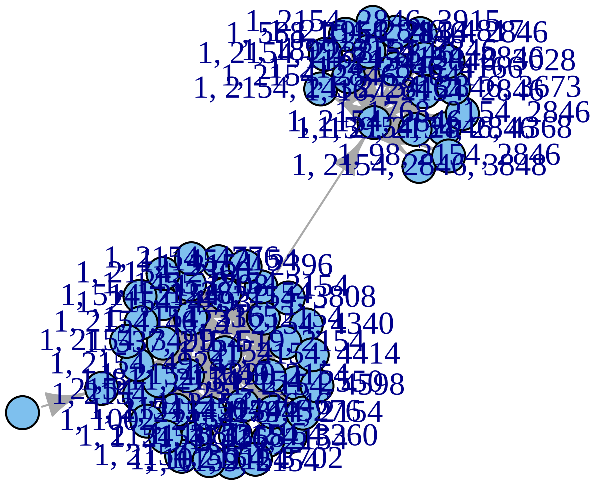

which might be easier to show complex relationships or identify central or key clones.

It is of course quite possible that, especially if we consider few genes, the parent-child relationships will form a network, not a tree, as the same child node can have multiple parents. You can play with this example, modified from one we saw before (section 3.4.6):

```
op <- par(ask = TRUE)
while(TRUE) {
```

```
    tmp <- oncoSimulIndiv(smn1, model = "McFL",
                          mu = 5e-5, finalTime = 500,
                          detectionDrivers = 3,
                          onlyCancer = FALSE,
                          initSize = 1000, keepPhylog = TRUE)
    plotClonePhylog(tmp, N = 0)
}
par(op)
```

8.1 Parent-child relationships from multiple runs

If you use `oncoSimulPop` you can store and plot the “phylogenies" of the different runs:

```
oi <- allFitnessEffects(orderEffects =
               c("F > D" = -0.3, "D > F" = 0.4),
               noIntGenes = rexp(5, 10),
                          geneToModule =
                              c("F" = "f1, f2, f3",
                                "D" = "d1, d2") )
oiI1 <- oncoSimulIndiv(oi, model = "Exp")
oiP1 <- oncoSimulPop(4, oi,
                     keepEvery = 10,
                     mc.cores = 2,
                     keepPhylog = TRUE)
```

We will plot the first two:

```
op <- par(mar = rep(0, 4), mfrow = c(2, 1))
plotClonePhylog(oiP1[[1]])
plotClonePhylog(oiP1[[2]])
```

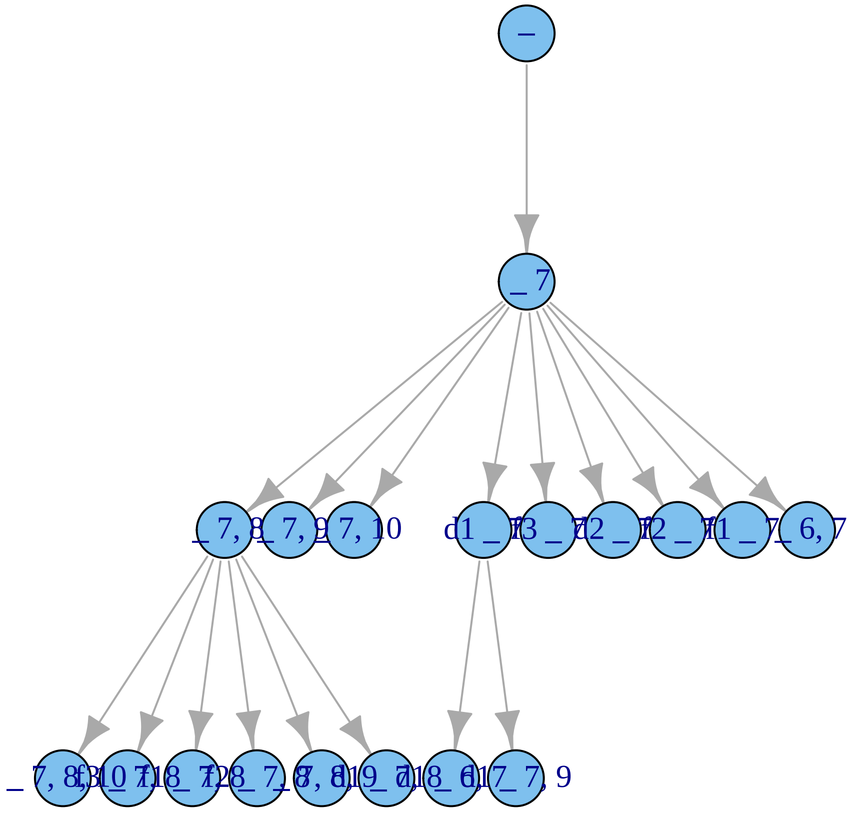

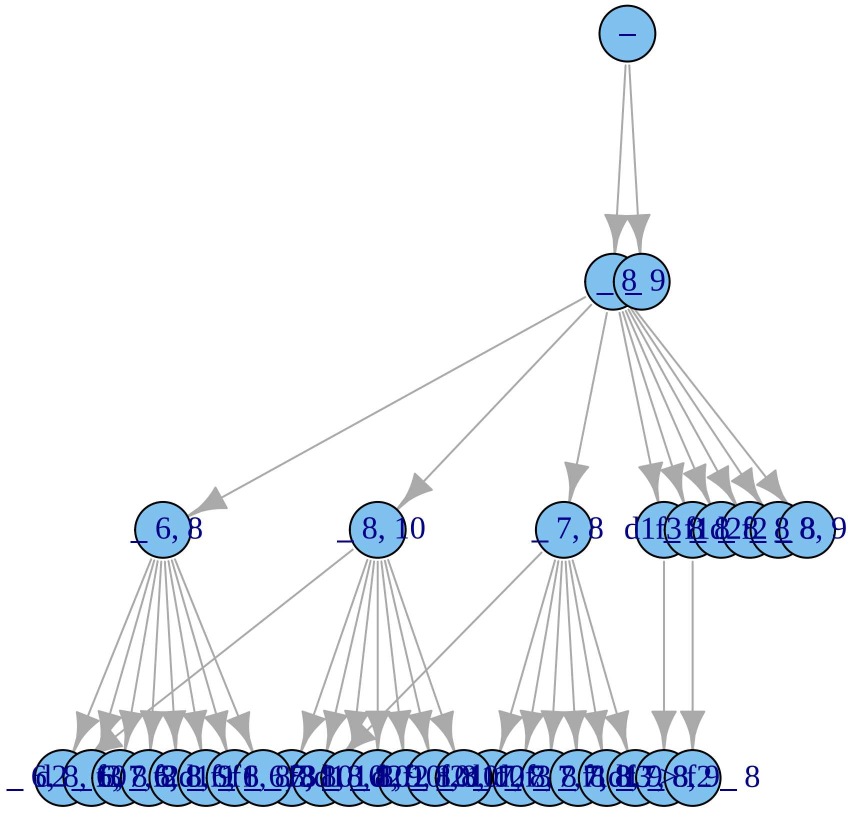


```r
par(op)
```

This is so far disabled in function `oncoSimulSample`, since that function is optimized for other uses. This might change in the future.

9 Generating random fitness landscapes

In most of the examples seen above, we have fully specified the fitness of the different genotypes (either by providing directly the full mapping genotypes to fitness, or by providing that mapping by specifying the effects of the different gene combinations). In some cases, however, we might want to specify a particular model that generates the fitness landscape, and then have fitnesses be random variables obtained under this model. In other words, in this random fitness landscape the fitness of the genotypes is a random variable generated under some specific model. Random fitness landscapes are used extensively, for instance, to understand the evolutionary consequences of different types of epistatic interactions (e.g., Szendro, Schenk, et al. 2013; Franke et al. 2011) and there are especially developed tools for plotting and analyzing random fitness landscapes (e.g., Brouillet et al. 2015).

With OncoSimulR it is possible to generate mappings of genotype to fitness using the function `rfitness` that allows you to use from a pure House of Cards model to a purely additive model. I have followed Szendro, Schenk, et al. (2013) and Franke et al. (2011) and model fitness as

$$f_i = -c\ d(i, reference) + x_i \tag{2}$$

where $d(i, j)$ is the Hamming distance between genotypes i and j (the number of positions that differ), c is the decrease in fitness of a genotype per each unit increase in Hamming distance from the reference genotype, and x_i is a random variable (in this case, a normal deviate of mean 0 and standard deviation sd). You can change the reference genotype to any of the genotypes: for the deterministic part, you make the fittest genotype be the one with all positions mutated by setting `reference = "max"`, or use the wildtype by using a string of 0s, or randomly select a genotype as a reference by using `reference = "random"` or `reference = "random2"`.
And by changing c and sd you can flexibly modify the relative weight of the purely House of Cards vs. additive component. The expression used above is also very similar to the one on Greene and Crona (2014) if you use `rfitness` with the argument `reference = "max"`.

What can you do with these genotype to fitness mappings? You could plot them, you could use them as input for `oncoSimulIndiv` and related functions, or you could export them (`to_Magellan`) and plot them externally (e.g., in MAGELLAN: http://wwwabi.snv.jussieu.fr/public/Magellan/, Brouillet et al. (2015)).

```
## A small example
rfitness(3)
##      A B C Fitness
## [1,] 0 0 0  1.0000
## [2,] 1 0 0  0.7115
## [3,] 0 1 0  0.7406
## [4,] 0 0 1  0.5147
## [5,] 1 1 0  1.2576
```

```
## [6,] 1 0 1  0.7401
## [7,] 0 1 1  0.7631
## [8,] 1 1 1  1.0245
## attr(,"class")
## [1] "matrix"                  "genotype_fitness_matrix"

## A 5-gene example, where the reference genotype is the
## one with all positions mutated, similar to Greene and Crona,
## 2014.  We will plot the landscape and use it for simulations
## We downplay the random component with a sd = 0.5

r1 <- rfitness(5, reference = rep(1, 5), sd = 0.6)
plot(r1)
```

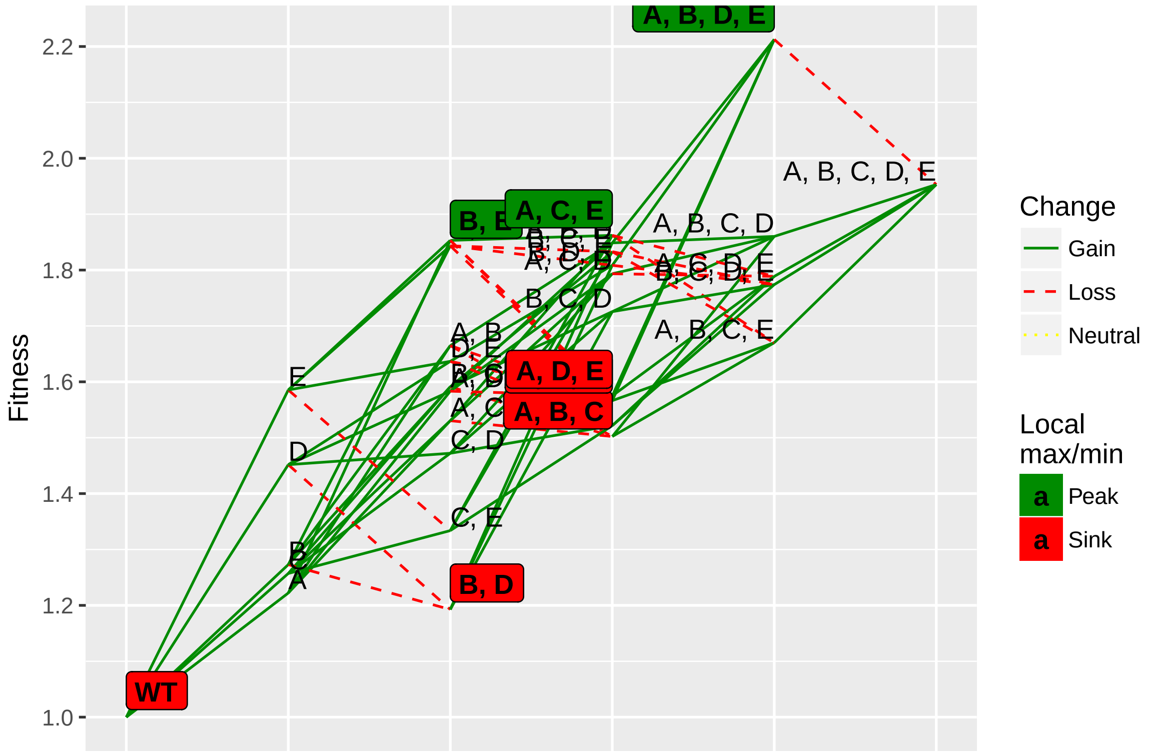


```
oncoSimulIndiv(allFitnessEffects(genotFitness = r1))
##
## Individual OncoSimul trajectory with call:
##  oncoSimulIndiv(fp = allFitnessEffects(genotFitness = r1))
##
##   NumClones TotalPopSize LargestClone MaxNumDrivers MaxDriversLast
## 1         6    156344752    156304634             0              0
##   NumDriversLargestPop TotalPresentDrivers FinalTime NumIter
## 1                    0                   0       766    2521
##   HittedWallTime HittedMaxTries errorMF minDMratio minBMratio
## 1          FALSE          FALSE      NA    157684      2e+05
##   OccurringDrivers
```

```
## 1
##
## Final population composition:
##   Genotype         N
## 1                2412
## 2     A, E      8620
## 3     B, E     25790
## 4     C, E       867
## 5     D, E      2429
## 6        E 156304634
```

10 Measures of evolutionary predictability and genotype diversity

Several measures of evolutionary predictability have been proposed in the literature (see, e.g., Szendro, Franke, et al. (2013) and references therein). We provide two, Lines of Descent (LOD) and Path of the Maximum (POM), following Szendro, Franke, et al. (2013); we also provide a simple measure of diversity of the actual genotypes sampled.

In Szendro, Franke, et al. (2013) "(...) paths defined as the time ordered sets of genotypes that at some time contain the largest subpopulation" are called "Path of the Maximum" (POM) (see their p. 572). In our case, POM are obtained from the `pops.by.time` returned object (i.e., from the genotypes at each of the sampling times) and, thus, the POMs will be affected by how often we sample and keep samples (arguments `sampleEvery` and `keepEvery`), since we are running a continuous time process.

Szendro, Franke, et al. (2013) also define Lines of Descent (LODs) which "(...) represent the lineages that arrive at the most populated genotype at the final time". In that same page (572) they provide the details on how the LODs are obtained. My implementation is not exactly identical to the definition given in p. 572 of Szendro et al. First, in case this might be useful, for each simulation I keep all the paths that "(...) arrive at the most populated genotype at the final time" (first paragraph in p. 572 of Szendro et al.), whereas they only keep one (see second column of p. 572). However, I do provide a single LOD for each run, too. This is the first path to arrive at the genotype that eventually becomes the most populated genotype at the final time (and, in this sense, agrees with the LOD of Szendro et al.). However, in contrast to what is apparently done in Szendro ("A given genotype may undergo several episodes of colonization and extinction that are stored by the algorithm, and the last episode before the colonization of the final state is used to construct the step."), I do not check that this genotype (which is the one that will become the most populated at final time) does not become extinct before the final colonization. So there could be other paths (all in `all_paths`) that are actually the one(s) that are colonizers of the most populated genotype (with no extinction before the final colonization). Obtaining LOD requires that the simulations be run with `keepPhylog = TRUE` (we need the genealogy of clones).

To briefly show some output, we will use again the 5.5 example. Note that using LOD requires running the simulations with `keepPhylog = TRUE`.

```
pancr <- allFitnessEffects(
    data.frame(parent = c("Root", rep("KRAS", 4), "SMAD4", "CDNK2A",
                          "TP53", "TP53", "MLL3"),
               child = c("KRAS","SMAD4", "CDNK2A",
                         "TP53", "MLL3",
                         rep("PXDN", 3), rep("TGFBR2", 2)),
               s = 0.05, sh = -0.3, typeDep = "MN"))
```

```
pancr16 <- oncoSimulPop(16, pancr, model = "Exp", keepPhylog = TRUE,
                        mc.cores = 2)

## Look a the first POM
str(POM(pancr16)[1:3])
## List of 3
##  $ : chr [1:2] "" "KRAS"
##  $ : chr [1:4] "" "KRAS" "" "KRAS"
##  $ : chr [1:2] "" "KRAS"

## Note here that for each simulation there is a "all_paths" and
## a "lod_single", as explained above
LOD(pancr16)[1:2]
## [[1]]
## [[1]]$all_paths
## [[1]]$all_paths[[1]]
## + 2/? vertices, named:
## [1]      KRAS
##
##
## [[1]]$lod_single
## + 2/? vertices, named:
## [1]      KRAS
##
##
## [[2]]
## [[2]]$all_paths
## [[2]]$all_paths[[1]]
## + 2/? vertices, named:
## [1]      KRAS
##
##
## [[2]]$lod_single
## + 2/? vertices, named:
## [1]      KRAS

## The diversity of LOD (lod_single) and POM might or might not
## be identical
diversityPOM(POM(pancr16))
## [1] 0.6886
diversityLOD(LOD(pancr16))
## [1] 0.4634

## Show the genotypes and their diversity (which might, or might
## not, differ from the diversity of LOD and POM)
```

```r
sampledGenotypes(samplePop(pancr16))
##
##  Subjects by Genes matrix of 16 subjects and 7 genes.
##       Genotype Freq
## 1 CDNK2A, KRAS    1
## 2         KRAS   14
## 3  KRAS, SMAD4    1
##
##  Shannon's diversity (entropy) of sampled genotypes: 0.4634
```

11 Generating random DAGs for restrictions

You might want to randomly generate DAGs like those often found in the literature on Oncogenetic trees et al. Function `simOGraph` might help here.

```
## No seed fixed, so reruns will give different DAGs.
(a1 <- simOGraph(10))
##      Root 1 2 3 4 5 6 7 8 9 10
## Root    0 1 1 1 1 0 0 0 0 0  0
## 1       0 0 0 0 0 1 0 0 0 0  0
## 2       0 0 0 0 0 1 0 0 0 0  0
## 3       0 0 0 0 0 0 1 0 0 0  0
## 4       0 0 0 0 0 1 0 0 0 0  0
## 5       0 0 0 0 0 0 1 0 0 0  0
## 6       0 0 0 0 0 0 0 1 1 1  1
## 7       0 0 0 0 0 0 0 0 0 0  0
## 8       0 0 0 0 0 0 0 0 0 0  0
## 9       0 0 0 0 0 0 0 0 0 0  0
## 10      0 0 0 0 0 0 0 0 0 0  0
library(graph) ## for simple plotting
plot(as(a1, "graphNEL"))
```

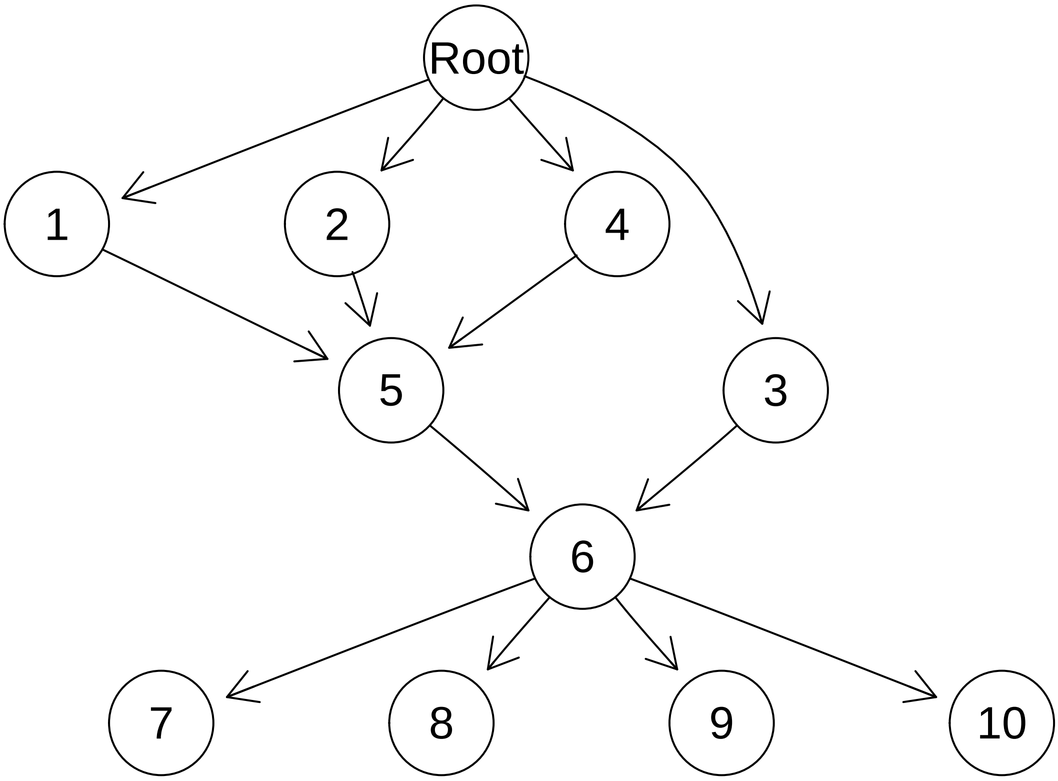

Once you obtain the adjacency matrices, it is for now up to you to convert them into appropriate posets or fitnessEffects objects.

Why this function? I searched for, and could not find any that did what I wanted, in particular bounding the number of parents, being able to specify the approximate

depth[14] of the graph, and optionally being able to have DAGs where no node is connected to another both directly (an edge between the two) and indirectly (there is a path between the two through other nodes). So I wrote my own code. The code is fairly simple to understand (all in file `generate-random-trees.R`). I would not be surprised if this way of generating random graphs has been proposed and named before; please let me know, best if with a reference.

Should we remove direct connections if there are indirect? Or, should we set `removeDirectIndirect = TRUE`? Except for Farahani and Lagergren (2013) and Ramazzotti et al. (2015), none of the DAGs I've seen in the context of CBNs, Oncogenetic trees, etc, include both direct and indirect connections between nodes. If these exist, reasoning about the model can be harder. For example, with CBN (AND or CMPN or monotone relationships) adding a direct connection makes no difference iff we assume that the relationships encoded in the DAG are fully respected (e.g., all $s_h = -\infty$). But it can make a difference if we allow for deviations from the monotonicity, specially if we only check for the satisfaction of the presence of the immediate ancestors. And things get even trickier if we combine XOR with AND. The code for computing fitness, however, should deal with all of this just fine.

[14]Where depth is defined in the usual way to mean smallest number of nodes —or edges— to traverse to get from the bottom to the top of the DAG.

12 FAQ, odds and ends

12.1 What we mean by "clone"; and "I want clones disregarding passengers"

In this vignette we often use "clone" or "genotype" interchangeably. A clone denotes a set of cells that have identical genotypes. So if you are using a fitness specification with four genes (i.e., your genome has only four loci), there can be up to $16 = 2^4$ different genotypes or clones. Any two entities that differ in the genotype are different clones. And this applies regardless of whether or not you declare that some genes (loci) are drivers or not. So if you have four genes, it does not matter whether only the first or all four are regarded as drivers; you will always have at most 16 different clones or 16 different genotypes. Of course you can arrive at the same clone/genotype by different routes. Just think about loci A and B in our four-loci genome, and how you can end up with a cell with both A and B mutated.

Analogously, if you have 100 genes, 10 drivers and 90 passengers, you can have up to 2^{100} different clones or genotypes. Sure, one cell might have driver A mutated and passenger B mutated, and another cell might have driver A mutated and passenger C mutated. So if you only look at drivers you might be tempted to say that they are "the same clone for all practical purposes"; but they really are not the same clone as they differ in their genotype and this makes a lot of difference computationally.

If you want summaries of simulations that collapse over some genes (say, some "passengers", the 90 passengers we just mentioned) look at the help for `samplePop`, argument `geneNames`. This would allow you, for instance, to look at the diversity of clones/genotypes, considering as identical those genotypes that only differ in genes you deem relevant; something similar to defining a "drivers' clone" as the set formed from the union of all sets of cells that have identical genotype with respect to only the drivers (so that in the example of "A, B" and "A, C" just mentioned both cells would be considered "the same clone" as they only differ with respect to passengers). However, this "disregard some genes" only applies to summaries of simulations once we are done simulating data. OncoSimulR will always track clones, as defined above, regardless of whether many of those clones have the same genotype if you were to only focus on driver genes; see also section 12.2.

Labeling something as a "driver", therefore, does not affect what we mean by clone. Yes, labeling something as a driver can affect when you stop simulations if you use `detectionDrivers` as a stopping mechanism (see section 6.3). But, again, this has nothing to do with the definition of "clone".

If this is all obvious to you, ignore it. I am adding it here because I've seen strange misunderstandings that eventually could be traced to the apparently multiple meanings of clone. (And to make the story complete, Mather, Hasty, and Tsimring (2012) use the expression "class" —e.g., Algorithm 4 in the paper, Algorithm 5 in the supplementary material).

12.2 Does OncoSimulR keep track of individuals or of clones? And how can it keep track of such large populations?

OncoSimulR keeps track of clones, where a clone is a set of cells that are genetically identical (note that this means completely identical over the whole set of genes/markers you are using; see section 12.1). We do not need to keep track of individual cells because, for all purposes, and since we do not consider spatial structure, two or more cells that are genetically identical are interchangeable. This means, for instance, that the computational cost of keeping a population of a single clone with 1 individual or with 10^9 individuals is exactly the same: we just keep track of the genotype and the number of cells. (Sure, it is much more likely we will see a mutation soon in a clone with 10^9 cells than in a clone with 1, but that is a different issue.)

Of course, the entities that die, reproduce, and mutate are individual cells. This is of course dealt with by tracking clones (as is clearly shown by Algorithms 4 and 5 in Mather, Hasty, and Tsimring (2012)). Tracking individuals, as individuals, would provide no advantage, but would increase the computational burden by many orders of magnitude.

12.2.1 `sampleEvery`, `keepPhylog`, and pruning

At each sampling time (where `sampleEvery` determines the time units between sampling times) the abundance of all the clones with number of cells > 0 is recorded. This is the structure that at the end of the run is converted into the `pops.by.time` matrix.

Now, some clones might arise from mutation between successive population samples but these clones might be extinct by the time we take a population sample. These clones do not appear in the `pops.by.time` matrix because, as we just said, they have 0 cells at the time of sampling. Of course, some of these clones might appear again later and reach a size larger than 0 at some posterior sampling time; it is at this time when this/these clone(s) will appear in the `pops.by.time` matrix. This pruning of clones with 0 cells can allow considerable savings in computing time (OncoSimulR needs to track the genotype of clones, their population sizes, their birth, death, and mutation rates, their next mutation time and the last time they were updated and thus it is important that we only loop over structures with information that is really needed).

However, we still need to track clones as clones, not simply as classes such as "number of mutated genes". Therefore, very large genomes can represent a problem if they lead to the creation and tracking of many different clones (even if they have the same number of mutated genes), as we have seen, for instance, in section 2.3. In this case, programs that only keep track of numbers of mutated genes or of drivers, not individual clones, can of course achieve better speed.

What about the genealogy? If you ask OncoSimulR to keep track of the complete parent-child relationships (`keepPhylog = TRUE`), you might see in the genealogy clones that are not present in `pops.by.time` if these are clones that never had a population size larger than 0 at any sampling time. To give an example, suppose that we will take population samples at times 0, 1, and 2. Clone A, with a population size larger than 0 at time 1, gives rise at time 1.5 to clone B; clone B then gives rise to clone C at time 1.8. Finally, suppose that at time 2 only clone C is alive. In other words, when we carry out the update of the population with Algorithm 5 from Mather, Hasty, and Tsimring (2012), clones A and B have size 0. Now, at time 1 clones B and C did not yet exist, and clone B is never alive at times 1 or 2. Thus, clone B is not present in `pops.by.time`. But we cannot remove clone B from our genealogy if we want to reflect the complete genealogy of C. Thus, `pops.by.time` will show only clones A and C (not B) but the complete genealogy will show clones A, B, C (and will show that B appeared from A at time 1.5 and C appeared from B at time 1.8). Since function `plotClonePhylog` offers a lot of flexibility with respect to what clones to show depending on their population sizes at different times, you can prevent being shown B, but its existence is there should you need it (see also 2.3.5).

12.3 Dealing with errors in "oncoSimulPop"

When running OncoSimulR under Windows `mclapply` does not use multiple cores, and errors from `oncoSimulPop` are reported directly. For example:

```
## This code will only be evaluated under Windows
if(.Platform$OS.type == "windows")
    try(pancrError <- oncoSimulPop(10, pancr,
                              initSize = 1e-5,
                              detectionSize = 1e7,
                              keepEvery = 10,
                              mc.cores = 2))
```

Under POSIX operating systems (e.g., GNU/Linux or Mac OSX) `oncoSimulPop` can ran parallelized by calling `mclapply`. Now, suppose you did something like

```
## Do not run under Windows
if(.Platform$OS.type != "windows")
    pancrError <- oncoSimulPop(10, pancr,
                              initSize = 1e-5,
                              detectionSize = 1e7,
                              keepEvery = 10,
                              mc.cores = 2)
## Warning in mclapply(seq.int(Nindiv), function(x) oncoSimulIndiv(fp =
## fp, : all scheduled cores encountered errors in user code
```

The warning you are seeing tells you there was an error in the functions called by `mclapply`. If you check the help for `mclpapply` you'll see that it returns a try-error

object, so we can inspect it. For instance, we could do:

```
pancrError[[1]]
```

But the output of this call might be easier to read:

```
pancrError[[1]][1]
```

And from here you could see the error that was returned by `oncoSimulIndiv`: `initSize < 1` (which is indeed true: we pass `initSize = 1e-5`).

12.4 Whole tumor sampling, genotypes, and allele counts: what gives? And what about order?

You are obtaining genotypes, regardless of order. When we use "whole tumor sampling", it is the frequency of the mutations in each gene that counts, not the order. So, for instance, "c, d" and "c, d" both contribute to the counts of "c" and "d". Similarly, when we use single cell sampling, we obtain a genotype defined in terms of mutations, but there might be multiple orders that give this genotype. For example, $d > c$ and $c > d$ both give you a genotype with "c" and "d" mutated, and thus in the output you can have two columns with both genes mutated.

12.5 Doesn't the BNB algorithm require small mutation rates for it to be advantageous?

As discussed in the original paper by Mather, Hasty, and Tsimring (2012) (see also their supplementary material), the BNB algorithm can achieve considerable speed advantages relative to other algorithms especially when mutation events are rare relative to birth and death events; the larger the mutation rate, the smaller the gains compared to other algorithms. As mentioned in their supplementary material (see p.5) "Note that the 'cost' of each step in BNB is somewhat higher than in SSA [SSA is the original Gillespie's Stochastic Simulation Algorithm] since it requires generation of several random numbers as compared to only two uniform random numbers for SSA. However this cost increase is small compared with significant benefits of jumping over birth and death reactions for the case of rare mutations."

Since the earliest versions, OncoSimulR has provided information to assess these issues. The output of function `oncoSimulIndiv` includes a list called *"other"* that itself includes two lists named *"minDMratio"* and *"minBMratio"*, the smallest ratio, over all simulations, of death rate to mutation rate or birth rate to mutation rate, respectively. As explained above, the BNB algorithm thrives when those are large. Note, though, we say "it thrives": these ratios being large is not required for the BNB algorithm to be an exact simulation algorithm; these ratios being large make BNB comparatively much faster than other algorithms.

12.6 Can we use the BNB algorithm with state-dependent birth or death rates?

As discussed in the original paper by Mather, Hasty, and Tsimring (2012) (see sections 2.6 and 3.2 of the paper and section E of the supplementary material), the BNB algorithm can be used as an approximate stochastic simulation algorithm "(...) with non-constant birth, death, and mutation rates by evolving the system with a BNB step restricted to a short duration t." (p. 9 in supplementary material). The justification is that "(...) the propensities for reactions can be considered approximately constant during some short interval." (p. 1234). This is the reason why, when we use McFarland's model, we set a very short `sampleEvery`. In addition, the output of the simulation functions contains the simple summary statistic `errorMF` that can be used to assess the quality of the approximation[15].

Note that, as the authors point out, approximations are common with stochastic simulation algorithms when there is density dependence, but the advantage of the BNB algorithm compared to, say, most tau-leap methods is that clones of different population sizes are treated uniformly. Mather, Hasty, and Tsimring (2012) further present results from simulations comparing the BNB algorithm with the original direct SSA method and the tau-leaps (see their Fig. 5), which shows that the approximation is very accurate as soon as the interval between samples becomes reasonably short.

12.7 Sometimes I get exceptions when running with mutator genes

Yes, sure, the following will cause an exception; this is similar to the example used in 1.3.4 but there is one crucial difference:

```
sd <- 0.1 ## fitness effect of drivers
sm <- 0 ## fitness effect of mutator
nd <- 20 ## number of drivers
nm <- 5  ## number of mutators
mut <- 50 ## mutator effect  THIS IS THE DIFFERENCE
```

[15] Death rates are affected by density dependence and, thus, it is on the death rates where the approximation that they are constant over a short interval plays a role. Thus, we examine how large the difference between successive death rates is. More precisely, let A and C denote two successive sampling periods, with $D_A = log(1 + N_A/K)$ and $D_C = log(1 + N_C/K)$ their death rates. `errorMF_size` stores the largest $abs(D_C - D_A)$ between any two sampling periods ever seen during a simulation. `errorMF` stores the largest $abs(D_C - D_A)/D_A$. Additionally, a simple procedure to use is to run the simulations with different values of `sampleEvery`, say the default value of 0.025 and values that are 10, 20, and 50 times larger or smaller, and assess their effects on the output of the simulations and the `errorMF` statistic itself. You can check that using a `sampleEvery` much smaller than 0.025 rarely makes any difference in `errorMF` or in the simulation output (though it increases computing time significantly). And, just for the fun of it, you can also check that using huge values for `sampleEvery` can lead to trouble and will be manifested too in the simulation output with large and unreasonable jumps in total population sizes and sudden extinctions.

```r
fitnessGenesVector <- c(rep(sd, nd), rep(sm, nm))
names(fitnessGenesVector) <- 1:(nd + nm)
mutatorGenesVector <- rep(mut, nm)
names(mutatorGenesVector) <- (nd + 1):(nd + nm)

ft <- allFitnessEffects(noIntGenes = fitnessGenesVector,
                        drvNames = 1:nd)
mt <- allMutatorEffects(noIntGenes = mutatorGenesVector)
```

Now, simulate using the fitness and mutator specification. We fix the number of drivers to cancer, and we stop when those numbers of drivers are reached. Since we only care about the time it takes to reach cancer, not the actual trajectories, we set `keepEvery = NA`:

```r
ddr <- 4
set.seed(2)
RNGkind("L'Ecuyer-CMRG")
st <- oncoSimulPop(4, ft, muEF = mt,
                   detectionDrivers = ddr,
                   finalTime = NA,
                   detectionSize = NA,
                   detectionProb = NA,
                   onlyCancer = TRUE,
                   keepEvery = NA,
                   mc.cores = 2, ## adapt to your hardware
                   seed = NULL) ## for reproducibility
set.seed(NULL) ## return things to their "usual state"
```

What happened? That you are using five mutator genes, each with an effect of multiplying by 50 the mutation rate. So the genotype with all those five genes mutated will have an increased mutation rate of $50^5 = 312500000$. If you set the mutation rate to the default of $1e-6$ you have a mutation rate of 312 which makes no sense (and leads to all sorts of numerical issues down the road and an early warning).

Oh, but you want to accumulate mutator effects and have some, or the early ones, have a large effects and the rest progressively smaller effects? You can do that using epistatic effects for mutator effects.

12.8 What are good values of `sampleEvery`?

First, we need to differentiate between the McFarland and the exponential models. If you use the McFarland model, you should read section 12.6 but, briefly, the small default is probably a good choice.

With the exponential model, however, simulations can often be much faster if `sampleEvery` is large. How large? As large as you can make it. `sampleEvery` should not be larger than your desired `keepEvery`, where `keepEvery` determines the

resolution or granularity of your samples (i.e., how often you take a snapshot of the population). If you only care about the final state, then set `keepEvery = NA`.

The other factors that affects choosing a reasonable `sampleEvery` are mutation rate and population size. If population growth is very fast or mutation rate very large, you need to sample frequently to avoid the "Recoverable exception ti set to DBL_MIN. Rerunning." issue (see discussion in section 2.4).

12.9 What can you do with the simulations?

This is up to you. In section 13.2 we show an example where we infer an Oncogenetic tree from simulated data and in section 1.3 we go over a varied set of scientific questions where OncoSimulR could help.

13 Using v.1 posets and simulations

It is strongly recommended that you use the new (v.2) procedures for specifying fitness effects. However, the former v.1 procedures are still available, with only very minor changes to function calls. What follows below is the former vignette. You might want to use v.1 because for certain models (e.g., small number of genes, with restrictions as specified by a simple poset) simulations might be faster with v.1 (fitness evaluation is much simpler —we are working on further improving speed).

13.1 Specifying restrictions: posets

How to specify the restrictions is shown in the help for `poset`. It is often useful, to make sure you did not make any mistakes, to plot the poset. This is from the examples (we use an "L" after a number so that the numbers are integers, not doubles; we could alternatively have modified `storage.mode`).

```
## Node 2 and 3 depend on 1, and 4 depends on no one
p1 <- cbind(c(1L, 1L, 0L), c(2L, 3L, 4L))
plotPoset(p1, addroot = TRUE)
```

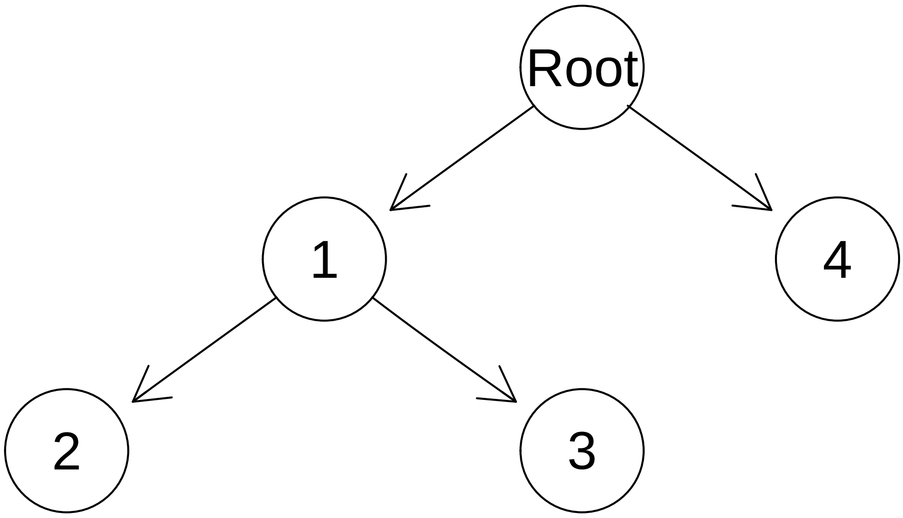


```
## A simple way to create a poset where no gene (in a set of 15)
## depends on any other.
p4 <- cbind(0L, 15L)
plotPoset(p4, addroot = TRUE)
```

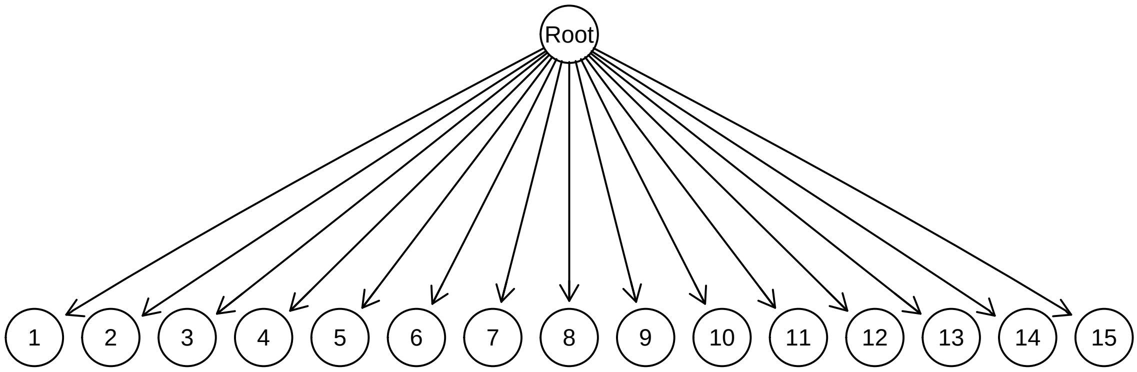

Specifying posets is actually straightforward. For instance, we can specify the pancreatic cancer poset in Gerstung et al. (2011) (their figure 2B, left). We specify the poset using numbers, but for nicer plotting we will use names (KRAS is 1, SMAD4 is 2, etc). This example is also in the help for `poset`:

```
pancreaticCancerPoset <- cbind(c(1, 1, 1, 1, 2, 3, 4, 4, 5),
                               c(2, 3, 4, 5, 6, 6, 6, 7, 7))
storage.mode(pancreaticCancerPoset) <- "integer"
plotPoset(pancreaticCancerPoset,
          names = c("KRAS", "SMAD4", "CDNK2A", "TP53",
                    "MLL3","PXDN", "TGFBR2"))
```

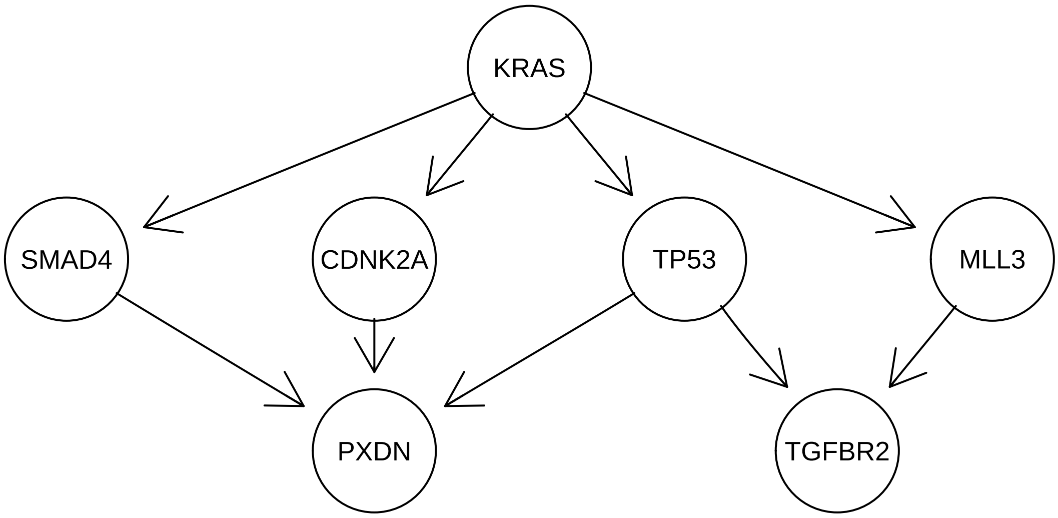

##

Simulating cancer progression {#simul1}

We can simulate the progression in a single subject. Using an example very similar to the one in the help:

```
## use poset p1101
data(examplePosets)
p1101 <- examplePosets[["p1101"]]

## Bozic Model
b1 <- oncoSimulIndiv(p1101, keepEvery = 15)
summary(b1)
```

```
##   NumClones TotalPopSize LargestClone MaxNumDrivers
## 1        16     50694307     16073302             4
##   MaxDriversLast NumDriversLargestPop TotalPresentDrivers
## 1              4                    1                   9
##   FinalTime NumIter HittedWallTime HittedMaxTries errorMF
## 1       556    3329          FALSE          FALSE      NA
##   minDMratio minBMratio          OccurringDrivers
## 1      90909      90909 1, 2, 3, 4, 5, 6, 7, 8, 9
```

The first thing we do is make it simpler (for future examples) to use a set of restrictions. In this case, those encoded in poset p1101. Then, we run the simulations and look at a simple summary and a plot.

If you want to plot the trajectories, it is better to keep more frequent samples, so you can see when clones appear:

```
b2 <- oncoSimulIndiv(p1101, keepEvery = 1)
summary(b2)
##   NumClones TotalPopSize LargestClone MaxNumDrivers
## 1        12     17151109      7246808             4
##   MaxDriversLast NumDriversLargestPop TotalPresentDrivers
## 1              4                    2                   6
##   FinalTime NumIter HittedWallTime HittedMaxTries errorMF
## 1      1852    2992          FALSE          FALSE      NA
##   minDMratio minBMratio OccurringDrivers
## 1      90909      90909 1, 2, 3, 7, 8, 9
plot(b2)
```

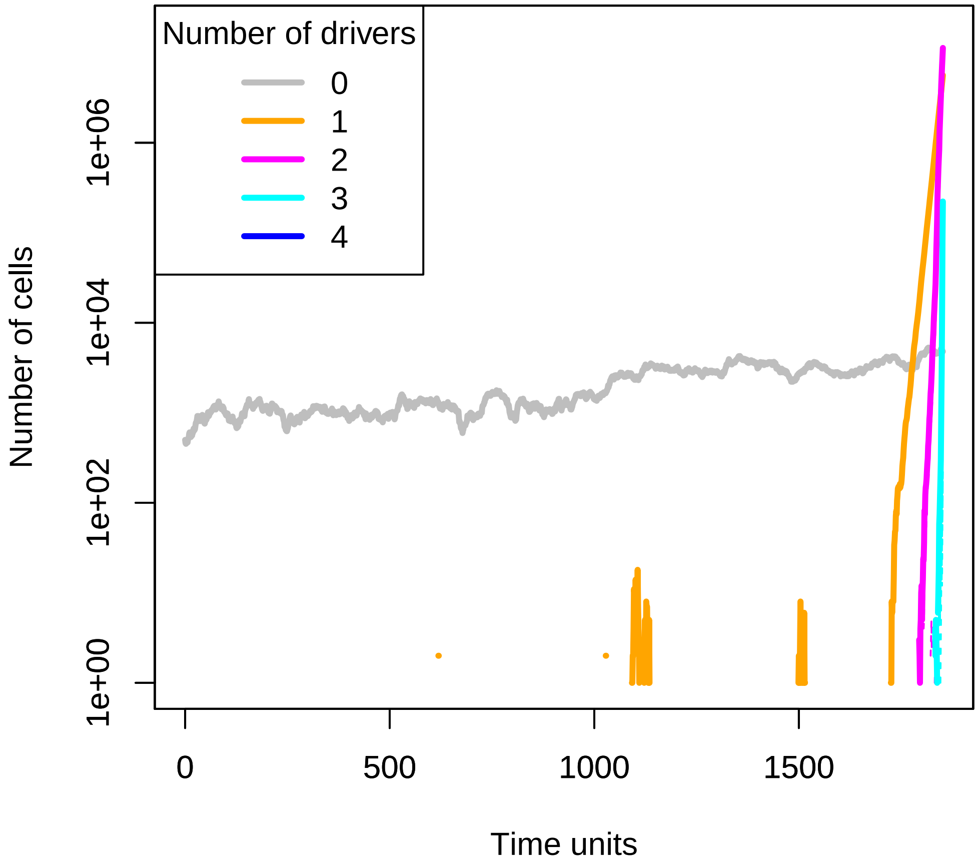

As we have seen before, the stacked plot here is less useful and that is why I do not evaluate that code for this vignette.

```
plot(b2, type = "stacked")
```

The following is an example where we do not care about passengers, but we want to use a different graph, and we want a few more drivers before considering cancer has been reached. And we allow it to run for longer. Note that in the McFL model `detectionSize` really plays no role. Note also how we pass the poset: it is the same as before, but now we directly access the poset in the list of posets.

```
m2 <- oncoSimulIndiv(examplePosets[["p1101"]], model = "McFL",
                     numPassengers = 0, detectionDrivers = 8,
                     mu = 5e-7, initSize = 4000,
                     sampleEvery = 0.025,
                     finalTime = 25000, keepEvery = 5,
                     detectionSize = 1e6)
```

(Very rarely the above run will fail to reach cancer. If that happens, execute it again.)

As usual, we will plot using both a line and a stacked plot:

```
par(mfrow = c(2, 1))
plot(m2, addtot = TRUE, log = "",
     thinData = TRUE, thinData.keep = 0.5)
```

```r
plot(m2, type = "stacked",
     thinData = TRUE, thinData.keep = 0.5)
```

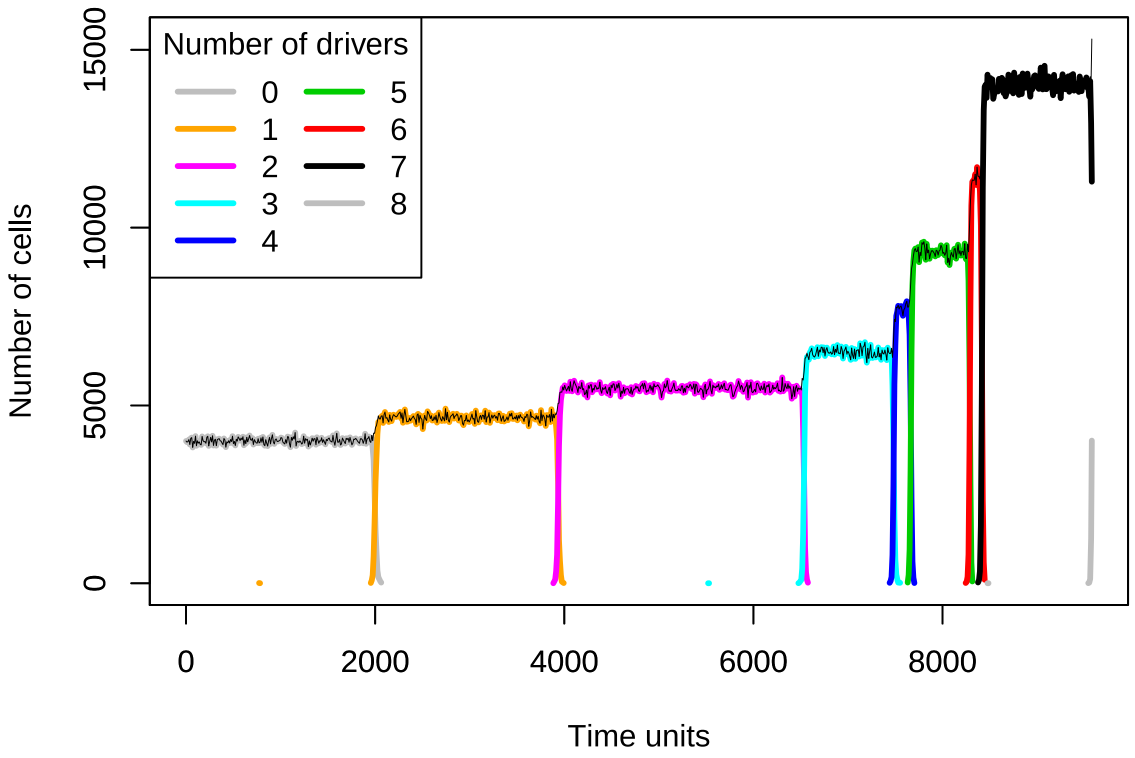

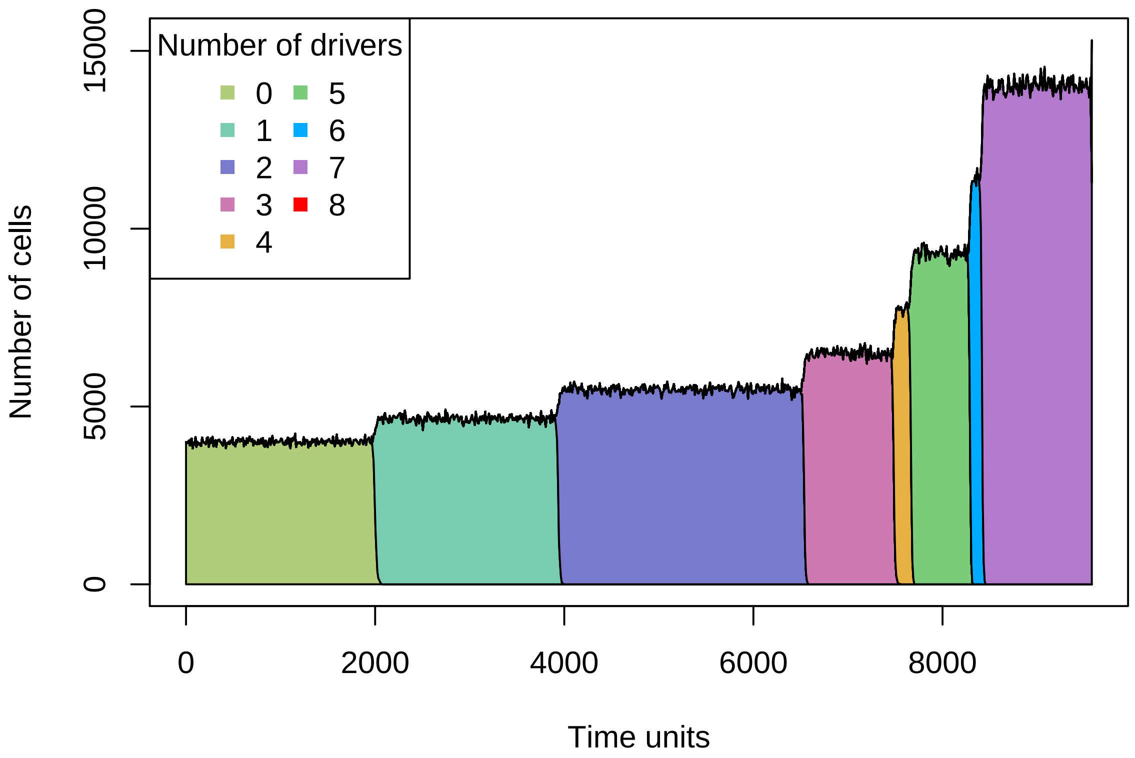

The default is to simulate progression until a simulation reaches cancer (i.e., only simulations that satisfy the detectionDrivers or the detectionSize will be returned).

If you use the McFL model with large enough `initSize` this will often be the case but not if you use very small `initSize`. Likewise, most of the Bozic runs do not reach cancer. Lets try a few:

```
b3 <- oncoSimulIndiv(p1101, onlyCancer = FALSE)
summary(b3)
##   NumClones TotalPopSize LargestClone MaxNumDrivers
## 1         1            0            0             0
##   MaxDriversLast NumDriversLargestPop TotalPresentDrivers
## 1              0                    0                   0
##   FinalTime NumIter HittedWallTime HittedMaxTries errorMF
## 1       751     753          FALSE          FALSE      NA
##   minDMratio minBMratio OccurringDrivers
## 1      90909      90909               NA

b4 <- oncoSimulIndiv(p1101, onlyCancer = FALSE)
summary(b4)
##   NumClones TotalPopSize LargestClone MaxNumDrivers
## 1         1            0            0             0
##   MaxDriversLast NumDriversLargestPop TotalPresentDrivers
## 1              0                    0                   0
##   FinalTime NumIter HittedWallTime HittedMaxTries errorMF
## 1       104     104          FALSE          FALSE      NA
##   minDMratio minBMratio OccurringDrivers
## 1      90909      90909               NA
```

Plot those runs:

```
par(mfrow = c(1, 2))
par(cex = 0.8) ## smaller font
plot(b3)
plot(b4)
```

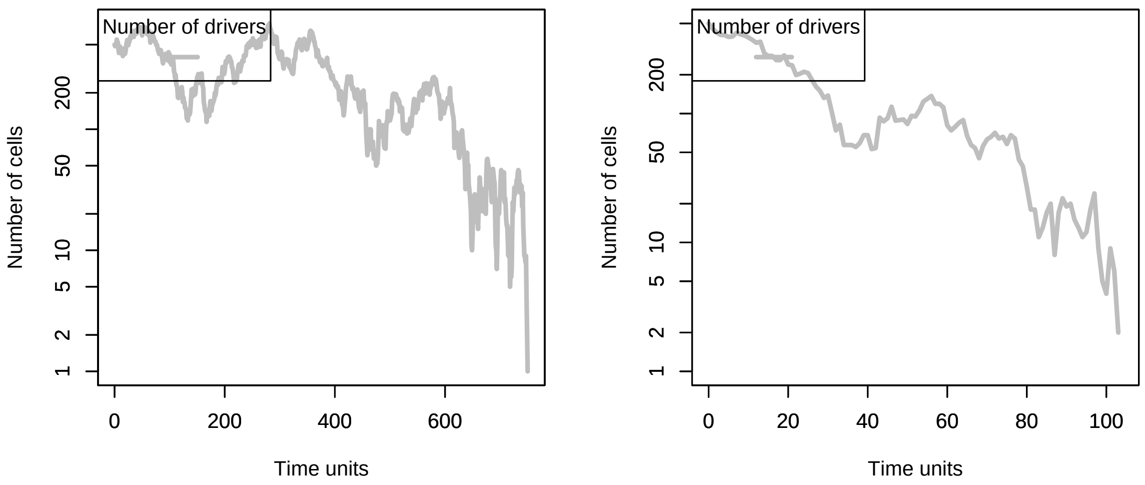

13.1.1 Simulating progression in several subjects

To simulate the progression in a bunch of subjects (we will use only four, so as not to fill the vignette with plots) we can do, with the same settings as above:

```
p1 <- oncoSimulPop(4, p1101, mc.cores = 2)
par(mfrow = c(2, 2))
plot(p1, ask = FALSE)
```

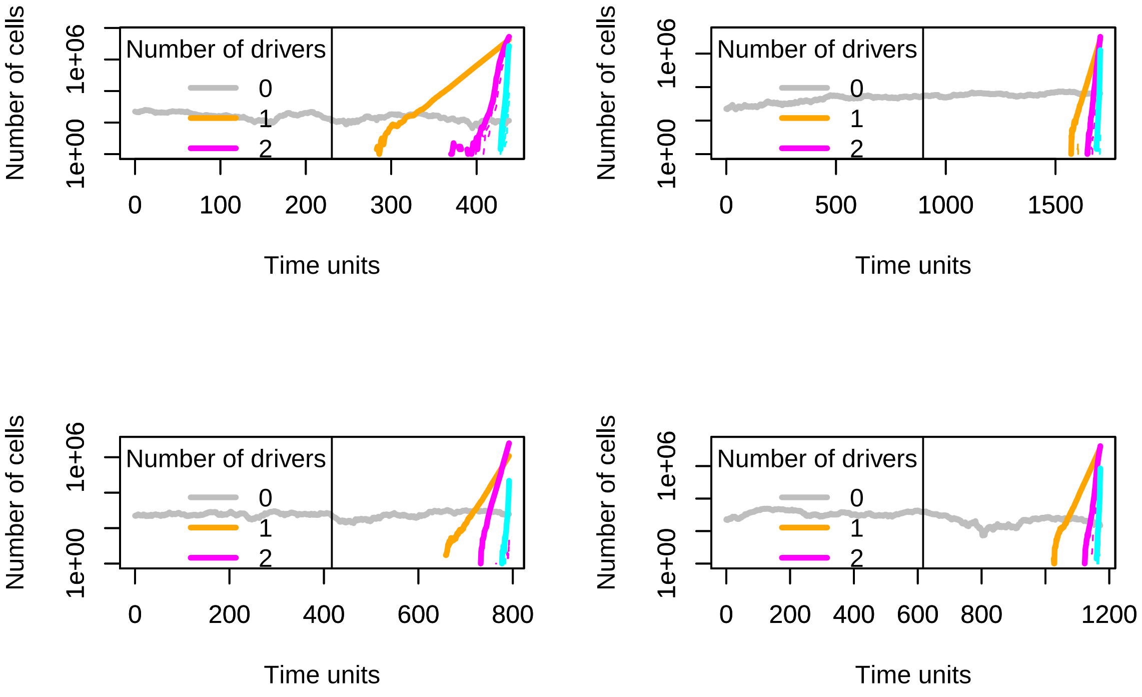

We can also use stream and stacked plots, though they might not be as useful in this case. For the sake of keeping the vignette small, these are commented out.

```
par(mfrow = c(2, 2))
plot(p1, type = "stream", ask = FALSE)
```

```
par(mfrow = c(2, 2))
plot(p1, type = "stacked", ask = FALSE)
```

13.2 Sampling from a set of simulated subjects

You will often want to do something with the simulated data. For instance, sample the simulated data. Here we will obtain the trajectories for 100 subjects in a scenario without passengers. Then we will sample with the default options and store that as a vector of genotypes (or a matrix of subjects by genes):

```
m1 <- oncoSimulPop(100, examplePosets[["p1101"]],
                   numPassengers = 0, mc.cores = 2)
```

The function samplePop samples that object, and also gives you some information about the output:

```
genotypes <- samplePop(m1)
##
##  Subjects by Genes matrix of 100 subjects and 11 genes.
```

What can you do with it? That is up to you. As an example, let us try to infer an Oncogenetic tree (and plot it) using the *Oncotree* package Szabo and Pappas (2013) after getting a quick look at the marginal frequencies of events:

```
colSums(genotypes)/nrow(genotypes)
##    1    2    3    4    5    6    7    8    9   10   11
## 0.49 0.04 0.02 0.00 0.00 0.00 0.54 0.03 0.04 0.00 0.00

require(Oncotree)
## Loading required package: Oncotree
## Loading required package: boot
ot1 <- oncotree.fit(genotypes)
## The following events had no observed occurances,so they will not be included in
## 4 5 6 10 11
plot(ot1)
```

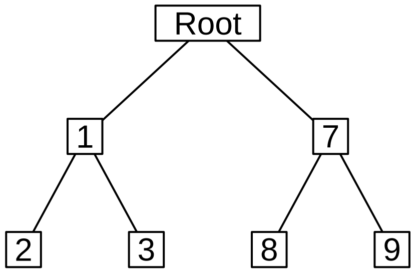

Your run will likely differ from mine, but with the defaults (detection size of 10^8) it is likely that events down the tree will never appear. You can set detectionSize = 1e9 and you will see that events down the tree are now found in the cross-sectional sample.

Alternatively, you can use single cell sampling and that, sometimes, recovers one or a couple more events.

```
genotypesSC <- samplePop(m1, typeSample = "single")
##
##  Subjects by Genes matrix of 100 subjects and 11 genes.
colSums(genotypesSC)/nrow(genotypesSC)
##    1    2    3    4    5    6    7    8    9   10   11
## 0.59 0.06 0.12 0.00 0.00 0.00 0.65 0.08 0.14 0.00 0.00

ot2 <- oncotree.fit(genotypesSC)
## The following events had no observed occurances,so they will not be included in
## 4 5 6 10 11
```

```r
plot(ot2)
```

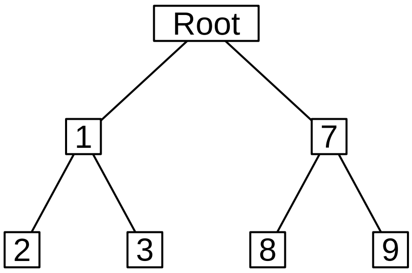

You can of course rename the columns of the output matrix to something else if you want so the names of the nodes will reflect those potentially more meaningful names.

14 Session info and packages used

This is the information about the version of R and packages used:

```
sessionInfo()
## R Under development (unstable) (2016-12-19 r71815)
## Platform: x86_64-pc-linux-gnu (64-bit)
## Running under: Debian GNU/Linux stretch/sid
##
## locale:
##  [1] LC_CTYPE=en_GB.utf8       LC_NUMERIC=C
##  [3] LC_TIME=en_GB.utf8        LC_COLLATE=en_GB.utf8
##  [5] LC_MONETARY=en_GB.utf8    LC_MESSAGES=en_GB.utf8
##  [7] LC_PAPER=en_GB.utf8       LC_NAME=C
##  [9] LC_ADDRESS=C              LC_TELEPHONE=C
## [11] LC_MEASUREMENT=en_GB.utf8 LC_IDENTIFICATION=C
##
## attached base packages:
## [1] parallel  methods   stats     graphics  grDevices
## [6] utils     datasets  base
##
## other attached packages:
##  [1] Oncotree_0.3.3      boot_1.3-18
##  [3] igraph_1.0.1        graph_1.53.0
##  [5] BiocGenerics_0.21.1 pander_0.6.0
##  [7] bookdown_0.3        BiocStyle_2.3.26
##  [9] rmarkdown_1.2       testthat_1.0.2
## [11] OncoSimulR_2.5.9
##
## loaded via a namespace (and not attached):
##  [1] gtools_3.5.0       splines_3.4.0
##  [3] lattice_0.20-34    colorspace_1.3-2
##  [5] htmltools_0.3.5    stats4_3.4.0
##  [7] yaml_2.1.14        mgcv_1.8-16
##  [9] nloptr_1.0.4       DBI_0.5-1
## [11] Rgraphviz_2.19.0   RColorBrewer_1.1-2
## [13] plyr_1.8.4         stringr_1.1.0
## [15] MatrixModels_0.4-1 munsell_0.4.3
## [17] gtable_0.2.0       evaluate_0.10
## [19] knitr_1.15.1       SparseM_1.74
## [21] quantreg_5.29      pbkrtest_0.4-6
## [23] highr_0.6          Rcpp_0.12.8
## [25] scales_0.4.1       backports_1.0.4
## [27] lme4_1.1-12        smatr_3.4-3
## [29] ggplot2_2.2.0      digest_0.6.10
## [31] stringi_1.1.2      dplyr_0.5.0
```

```
## [33] ggrepel_0.6.5      grid_3.4.0
## [35] rprojroot_1.1      tools_3.4.0
## [37] magrittr_1.5       lazyeval_0.2.0
## [39] tibble_1.2         crayon_1.3.2
## [41] car_2.1-4          MASS_7.3-45
## [43] Matrix_1.2-7.1     data.table_1.10.0
## [45] assertthat_0.1     minqa_1.2.4
## [47] R6_2.2.0           nnet_7.3-12
## [49] nlme_3.1-128       compiler_3.4.0
```

15 Funding

Supported by BFU2015-67302-R (MINECO/FEDER, EU).

16 References

Ashworth, Alan, Christopher J Lord, and Jorge S Reis-Filho. 2011. "Genetic interactions in cancer progression and treatment." *Cell* 145 (1). Elsevier Inc.: 30–38. doi:10.1016/j.cell.2011.03.020.

Bauer, Benedikt, Reiner Siebert, and Arne Traulsen. 2014. "Cancer initiation with epistatic interactions between driver and passenger mutations." *Journal of Theoretical Biology* 358. Elsevier: 52–60. doi:10.1016/j.jtbi.2014.05.018.

Beerenwinkel, Niko, Tibor Antal, David Dingli, Arne Traulsen, Kenneth W Kinzler, Victor E Velculescu, Bert Vogelstein, and Martin A Nowak. 2007. "Genetic progression and the waiting time to cancer." *PLoS Computational Biology* 3 (11): e225. doi:10.1371/journal.pcbi.0030225.

Beerenwinkel, Niko, Nicholas Eriksson, and Bernd Sturmfels. 2007. "Conjunctive Bayesian networks." *Bernoulli* 13 (4): 893–909. doi:10.3150/07-BEJ6133.

Bozic, Ivana, Tibor Antal, Hisashi Ohtsuki, Hannah Carter, Dewey Kim, Sining Chen, Rachel Karchin, Kenneth W Kinzler, Bert Vogelstein, and Martin A Nowak. 2010. "Accumulation of driver and passenger mutations during tumor progression." *Proceedings of the National Academy of Sciences of the United States of America* 107 (September): 18545–50. doi:10.1073/pnas.1010978107.

Brouillet, Sophie, Harry Annoni, Luca Ferretti, and Guillaume Achaz. 2015. "MAGELLAN: A Tool to Explore Small Fitness Landscapes." *BioRxiv*, November, 031583. doi:10.1101/031583.

Carvajal-Rodriguez, Antonio. 2010. "Simulation of Genes and Genomes Forward in Time." *Current Genomics* 11 (1): 58–61. doi:10.2174/138920210790218007.

Datta, Ruchira S, Alice Gutteridge, Charles Swanton, Carlo C Maley, and Trevor

A Graham. 2013. "Modelling the evolution of genetic instability during tumour progression." *Evolutionary Applications* 6 (1): 20–33. doi:10.1111/eva.12024.

Desper, R, F Jiang, O P Kallioniemi, H Moch, C H Papadimitriou, and A A Schäffer. 1999. "Inferring tree models for oncogenesis from comparative genome hybridization data." *J Comput Biol* 6 (1): 37–51. http://view.ncbi.nlm.nih.gov/pubmed/10223663.

Diaz-Uriarte, R. 2015. "Identifying restrictions in the order of accumulation of mutations during tumor progression: effects of passengers, evolutionary models, and sampling." *BMC Bioinformatics* 16 (41): 0–36. doi:doi:10.1186/s12859-015-0466-7.

Farahani, H, and J Lagergren. 2013. "Learning oncogenetic networks by reducing to mixed integer linear programming." *PloS One* 8 (6): e65773. doi:10.1371/journal.pone.0065773.

Franke, Jasper, Alexander Klözer, J. Arjan G. M. de Visser, and Joachim Krug. 2011. "Evolutionary Accessibility of Mutational Pathways." *PLoS Comput Biol* 7 (8): e1002134. doi:10.1371/journal.pcbi.1002134.

Gerrish, Philip J., Alexandre Colato, Alan S. Perelson, and Paul D. Sniegowski. 2007. "Complete Genetic Linkage Can Subvert Natural Selection." *Proceedings of the National Academy of Sciences of the United States of America* 104 (15): 6266–71. doi:10.1073/pnas.0607280104.

Gerstung, Moritz, Michael Baudis, Holger Moch, and Niko Beerenwinkel. 2009. "Quantifying cancer progression with conjunctive Bayesian networks." *Bioinformatics (Oxford, England)* 25 (21): 2809–15. doi:10.1093/bioinformatics/btp505.

Gerstung, Moritz, Nicholas Eriksson, Jimmy Lin, Bert Vogelstein, and Niko Beerenwinkel. 2011. "The Temporal Order of Genetic and Pathway Alterations in Tumorigenesis." *PLoS ONE* 6 (11): e27136. doi:10.1371/journal.pone.0027136.

Gillespie, J. H. 1993. "Substitution processes in molecular evolution. I. Uniform and clustered substitutions in a haploid model." *Genetics* 134 (3): 971–81.

Greene, Devin, and Kristina Crona. 2014. "The Changing Geometry of a Fitness Landscape Along an Adaptive Walk." *PLoS Computational Biology* 10 (5): e1003520. doi:10.1371/journal.pcbi.1003520.

Hartman, J L, B Garvik, and L Hartwell. 2001. "Principles for the buffering of genetic variation." *Science (New York, N.Y.)* 291 (5506): 1001–4. doi:10.1126/science.291.5506.1001.

Hjelm, M, M Höglund, and J Lagergren. 2006. "New probabilistic network models and algorithms for oncogenesis." *J Comput Biol* 13 (4). SBC; Dept. of Numerical Analysis; Computer Science, KTH, Stockholm, Sweden.: 853–65. doi:10.1089/cmb.2006.13.853.

Hoban, Sean, Giorgio Bertorelle, and Oscar E Gaggiotti. 2011. "Computer Simulations: Tools for Population and Evolutionary Genetics." *Nature Reviews. Genetics* 13 (2): 110–22. doi:10.1038/nrg3130.

Hothorn, Torsten, Kurt Hornik, Mark A. van de Wiel, and Achim Zeileis. 2006. "A

Lego System for Conditional Inference." *The American Statistician* 60 (3): 257–63.

———. 2008. "Implementing a Class of Permutation Tests: The coin Package." *Journal of Statistical Software* 28 (8): 1–23. http://www.jstatsoft.org/v28/i08/.

Korsunsky, Ilya, Daniele Ramazzotti, Giulio Caravagna, and Bud Mishra. 2014. "Inference of Cancer Progression Models with Biological Noise," August, 1–29. http://arxiv.org/abs/1408.6032v1 http://biorxiv.org/content/early/2014/08/25/00832.

Mather, William H, Jeff Hasty, and Lev S Tsimring. 2012. "Fast stochastic algorithm for simulating evolutionary population dynamics." *Bioinformatics (Oxford, England)* 28 (9): 1230–8. doi:10.1093/bioinformatics/bts130.

McFarland, CD. 2014. "The role of deleterious passengers in cancer." PhD thesis, Harvard University. http://nrs.harvard.edu/urn-3:HUL.InstRepos:13070047.

McFarland, Christopher D, Kirill S Korolev, Gregory V Kryukov, Shamil R Sunyaev, and Leonid A Mirny. 2013. "Impact of deleterious passenger mutations on cancer progression." *Proceedings of the National Academy of Sciences of the United States of America* 110 (8): 2910–5. doi:10.1073/pnas.1213968110.

McFarland, Christopher, Leonid Mirny, and Kirill S. Korolev. 2014. "A Tug-of-War Between Driver and Passenger Mutations in Cancer and Other Adaptive Processes." *Proc Natl Acad Sci U S A* 111 (42): 15138–43. doi:10.1101/003053.

Misra, Navodit, Ewa Szczurek, and Martin Vingron. 2014. "Inferring the paths of somatic evolution in cancer." *Bioinformatics (Oxford, England)* 30 (17): 2456–63. doi:10.1093/bioinformatics/btu319.

Nowak, M. A. 2006. *Evolutionary Dynamics: Exploring the Equations of Life*. Cambridge, Mass.: Belknap Press of Harvard University Press.

Ochs, Ian E, and Michael M Desai. 2015. "The competition between simple and complex evolutionary trajectories in asexual populations." *BMC Evolutionary Biology* 15 (1): 1–9. doi:10.1186/s12862-015-0334-0.

Ortmann, Christina a., David G. Kent, Jyoti Nangalia, Yvonne Silber, David C. Wedge, Jacob Grinfeld, E. Joanna Baxter, et al. 2015. "Effect of Mutation Order on Myeloproliferative Neoplasms." *New England Journal of Medicine* 372: 601–12. doi:10.1056/NEJMoa1412098.

Ramazzotti, Daniele, Giulio Caravagna, Loes Olde Loohuis, Alex Graudenzi, Ilya Korsunsky, Giancarlo Mauri, Marco Antoniotti, and Bud Mishra. 2015. "CAPRI: Efficient Inference of Cancer Progression Models from Cross-Sectional Data." *Bioinformatics (Oxford, England)* 31 (18): 3016–26. doi:10.1093/bioinformatics/btv296.

Raphael, Benjamin J, and Fabio Vandin. 2015. "Simultaneous Inference of Cancer Pathways and Tumor Progression from Cross-Sectional Mutation Data." *Journal of Computational Biology* 22 (00): 250–64. doi:10.1089/cmb.2014.0161.

Reiter, JG, Ivana Bozic, Krishnendu Chatterjee, and MA Nowak. 2013. "TTP: tool for tumor progression." In *Computer Aided Verification, Lecture Notes in Computer*

Science, edited by Natasha Sharygina and Helmut Veith, 101–6. Berlin, Heidelberg: Springer-Verlag. http://link.springer.com/chapter/10.1007/ 978-3-642-39799-8_6 http://dx.doi.org/10.1007/ 978-3-642-39799-8_6 http://pub.ist.ac.at/ttp/.

Szabo, A, and Kenneth M Boucher. 2008. "Oncogenetic trees." In *Handbook of Cancer Models with Applications*, edited by W-Y Tan and L Hanin, 1–24. World Scientific. http://www.worldscibooks.com/lifesci/6677.html.

Szabo, Aniko, and Lisa Pappas. 2013. "Oncotree: Estimating oncogenetic trees." http://cran.r-project.org/package=Oncotree.

Szendro, Ivan G., Jasper Franke, J. Arjan G. M. de Visser, and Joachim Krug. 2013. "Predictability of Evolution Depends Nonmonotonically on Population Size." *Proceedings of the National Academy of Sciences* 110 (2): 571–76. doi:10.1073/pnas.1213613110.

Szendro, Ivan G., Martijn F. Schenk, Jasper Franke, Joachim Krug, and J. Arjan G. M. de Visser. 2013. "Quantitative Analyses of Empirical Fitness Landscapes." *Journal of Statistical Mechanics: Theory and Experiment* 2013 (01): P01005. doi:10.1088/1742-5468/2013/01/P01005.

Tomlinson, I. P., M. R. Novelli, and W. F. Bodmer. 1996. "The Mutation Rate and Cancer." *Proceedings of the National Academy of Sciences of the United States of America* 93 (25): 14800–14803.

Weissman, Daniel B., Michael M. Desai, Daniel S. Fisher, and Marcus W. Feldman. 2009. "The rate at which asexual populations cross fitness valleys." *Theoretical Population Biology* 75 (4). Elsevier Inc.: 286–300. doi:10.1016/j.tpb.2009.02.006.

Yuan, Xiguo, David J. Miller, Junying Zhang, David Herrington, and Yue Wang. 2012. "An Overview of Population Genetic Data Simulation." *Journal of Computational Biology* 19 (1): 42–54. doi:10.1089/cmb.2010.0188.

Zanini, Fabio, and Richard a. Neher. 2012. "FFPopSim: An efficient forward simulation package for the evolution of large populations." *Bioinformatics* 28 (24): 3332–3. doi:10.1093/bioinformatics/bts633.
